# Variations of follicular fluid extracellular vesicles miRNAs content in relation to development stage and season in buffalo

**DOI:** 10.1038/s41598-022-18438-8

**Published:** 2022-09-01

**Authors:** Emanuele Capra, Michal Andrzej Kosior, Natascia Cocchia, Barbara Lazzari, Chiara Del Prete, Valentina Longobardi, Flavia Pizzi, Alessandra Stella, Roberto Frigerio, Marina Cretich, Anna Lange Consiglio, Bianca Gasparrini

**Affiliations:** 1 Istituto di Biologia e Biotecnologia Agraria, Consiglio Nazionale delle Ricerche IBBA CNR, Via Einstein 1, 26900 Lodi, Italy; 2 Dipartimento di Medicina Veterinaria e Produzioni Animali (DMVPA), Università degli Studi di Napoli Federico II, Via F. Delpino 1, 80137 Napoli, Italy; 3 Istituto di Scienze e Tecnologie Chimiche “Giulio Natta”, Consiglio Nazionale delle Ricerche SCITEC-CNR, Milano, Italy; 4 Dipartimento di Medicina Veterinaria e Scienze Animali (DIVAS), Università degli Studi di Milano, Via Celoria, 10, 20133 Lodi, Milano Italy

**Keywords:** Cell biology, Molecular biology

## Abstract

In buffalo (*Bubalus bubalis*) reproductive seasonality, causing cycles of milk production, is one of the major factors affecting farming profitability. Follicular fluid (FF) contains extracellular vesicles (EVs) playing an important role in modulating oocyte developmental competence and carrying microRNAs (miRNAs) essential for in vitro fertilization outcomes. The aim of this work was to characterize the FF-EVs-miRNA cargo of antral (An) and preovulatory (pO) follicles collected in the breeding (BS) and non-breeding (NBS) seasons, to unravel the molecular causes of the reduced oocyte competence recorded in buffalo during the NBS. In total, 1335 miRNAs (538 known Bos taurus miRNAs, 324 homologous to known miRNAs from other species and 473 new candidate miRNAs) were found. We identified 413 differentially expressed miRNAs (DE-miRNAs) (FDR < 0.05) between An and pO groups. A subset of the most significant DE-miRNAs between An and pO groups targets genes which function is related to the lipid and steroid metabolism, response to glucocorticoid and oestradiol stimulus. Comparison between BS and NBS showed 14 and 12 DE-miRNAs in An-FF-EVs and pO-FF-EVs, which regulate IL6 release and cellular adhesion, respectively. In conclusion, these results demonstrated that the miRNA cargo of buffalo FF-EVs varies in relation to both follicular development and season.

## Introduction

The importance of buffalo (*Bubalus bubalis*) breeding is clearly indicated by the positive growth trend all over the world (Faostat.fao.org/faostat), due to specific features making this species a valuable protein source, particularly for tropical countries. A peculiar situation is described in Italy, where the success of buffalo breeding is closely related to the production of mozzarella cheese, highly requested around the world. A major limiting factor is reproductive seasonality, impeding continuity of milk production throughout the year, and hence not allowing to meet the market demand. Buffalo is a short-day breeder, with an increased reproductive activity observed during decreasing day length months^1,2^. In Italy, where the Italian Mediterranean buffalo breed has been selected, the seasonality pattern shows an opposite trend to the market request. Therefore, the out of breeding mating strategy (OBMS), has been efficiently applied to distribute calving more evenly during the year^2^. However, forcing buffalo cows to conceive during the non-breeding season (NBS) may lead to extended post-partum anestrus, higher rates of embryonic mortality and overall reduced fertility^1,3,4^. In previous studies it was demonstrated that embryonic mortality is in part caused by impaired luteal function, and consequently reduced progesterone secretion^5^. This in turn interferes with embryo growth that is accompanied by transcriptomic and proteomic changes at the level of embryos and chorioamnios/caruncles^6,7^, definitively hampering embryo attachment. Embryonic mortality during the NBS is also in part due to reduced oocyte developmental competence, as shown by the decreased cleavage and blastocyst rates obtained after in vitro fertilization during increasing daylight months in Italian Mediterranean buffaloes^8,9^. A seasonal effect on follicular population and embryo outcomes was also reported by other authors^10,11^. A poorer oocyte quality was observed in Murrah buffalo heifers during long day months, that was associated to lower intrafollicular levels of estradiol and IGF-1^12^, known to influence oocyte development^13–15^. However, the underlining biological causes of reduced oocyte competence in the NBS have not yet been unraveled in buffalo.

It is known that oocyte developmental competence is acquired during the last phase of oocyte growth that is strictly coordinated with follicular development^16^. Folliculogenesis involves complex paracrine interactions within ovaries and the bidirectional communication between oocytes and surrounding somatic cells is essential for oocyte maturation and acquisition of developmental competence, i.e. the capability of the oocyte to undergo fertilization and embryogenesis. The follicular fluid composition is dynamic, as in part reflects that of plasma, due to transudation, in part results from the secretion of theca and granulosa cells^17^. The follicular fluid protects the oocyte during development and ensures the bidirectional communication between somatic and germinal compartments of the ovarian follicle, fundamental for oocyte developmental competence^18^. The analysis of follicular fluid is hence very important to identify markers associated to oocyte competence^19^.

The acquisition of developmental competence is a gradual process requiring a precisely regulated spatio-temporal expression of various genes^16,20^. In multicellular organisms, microRNAs (miRNAs) comprise an abundant class of gene regulatory molecules that regulate the expression of complementary messenger RNAs. MiRNAs are phylogenetically conserved in different animals and have a fundamental role in cell development, proliferation and death^21^. MiRNAs play a significant role in mammalian follicular and oocyte development^22^. Many miRNAs were observed to change their expression across ovarian developmental stages, and specific miRNAs were differentially expressed during follicular–luteal transition^23^. Equine follicular fluid contains different miRNAs, which expression changes between ovulatory and anovulatory follicles, in part reflecting changes observed in granulosa cells^24^. Follicular fluid hormones, metabolites and miRNA content is correlated with the follicular developmental stage^25,26^. Changes in follicular miRNA levels during folliculogenesis have been reported in cattle, suggesting a regulatory role in oocyte growth^27^. Furthermore, in the same species it was recently demonstrated that differences in developmental competence are reflected in changes of miRNAs profile in follicular fluid and oocytes^28^.

Follicular fluid contains extracellular vesicles (EVs), whose miRNAs cargo has been suggested to be implicated in cell communication in different species^27,29,30^. EVs are membrane-enclosed vesicles of cellular origin that can carry a large array of active molecules (lipids, proteins, and nucleic acids); they derive from the endosomal compartment (exosomes) or are released by budding or fission of the plasma membrane (microvesicles)^31,32^.

Co-incubation of bovine follicular EVs with cumulus-oocyte complexes (COC) was reported to promote cumulus expansion and increase the expression of key genes^33^. It was also demonstrated that EVs isolated from bovine follicular fluid enhance in vitro oocyte maturation and embryo development and influence miRNAs profile and developmental related genes in embryos^34^.

In sheep, another short-day breeder, seasonal effects were demonstrated on ovarian transcriptome and miRNAs profile between anestrus and breeding season^35,36^. Furthermore, seasonal-related variations in expression levels of miRNAs involved in hormone regulation, follicular growth and angiogenesis were also observed in estrous sheep^37^. Recently, we demonstrated that microRNAs (miRNAs) content and transcriptomic profile are altered by season both in buffalo oocytes and follicular cells^38^. However, the follicular fluid, composed by serum-derived and locally produced factors, providing a specialized environment for oocyte growth and maturation, has not been characterized, yet. The hypothesis underlying this work was that miRNAs content of EVs contained in buffalo follicular fluid might undergo seasonal variations that may account for the reduced oocyte developmental competence during the NBS. In order to test this hypothesis, we first investigated the miRNA cargo of EVs isolated from follicular fluid collected from antral and pre-ovulatory follicles to identify those that are implicated in folliculogenesis regulation. Then, the seasonal variations in EVs’ miRNA content were assessed by characterizing follicular fluid from antral and pre-ovulatory follicles during the breeding and non-breeding seasons.

## Results

### Follicular parameters and oocyte developmental competence

With regard to pre-ovulatory follicles no differences in size were recorded between BS and NBS (1.0 vs. 1.2 cm, respectively). With the limitation of the low numbers, it was noted that during the BS 100% pre-ovulatory follicles (5/5) contained a COC with a nice expansion of cumulus cells, while during the NBS only 2 out of 5 (40%) did.

Oocyte competence was assessed by evaluating cleavage and blastocyst rates of Grade A and B COCs recovered from antral follicles. As shown in Table 1, cleavage rate decreased (*P* < 0.05) during the NBS compared to the BS. Likewise, a reduction (*P* < 0.05) of blastocyst yields was recorded in the NBS compared to the BS, both in relation to total COCs (*P* < 0.01) and cleaved oocytes (*P* < 0.05).

**Table 1:** Cleavage and blastocyst rates after in vitro fertilization of abattoir-derived buffalo oocytes in relation to season.

	COCs	Cleavage rate	Blastocyst rate(out of COCs)	Blastocyst rate (out of cleaved)
	*n*	*n* (%)	*n* (%)	*n* (%)
Breeding season	140	103 (73.6)^a^	46 (32.9)^A^	46 (44.7)^a^
Non-breeding seasons	138	85 (61.6)^b^	27 (19.6)^B^	27 (31.8)^b^

^a, b^Values with different superscripts are significantly different; *P* < 0.05.

^A, B^Values with different superscripts are significantly different; *P* < 0.01.

### EVs isolation, characterization and miRNA profiling

**Figure 1:**
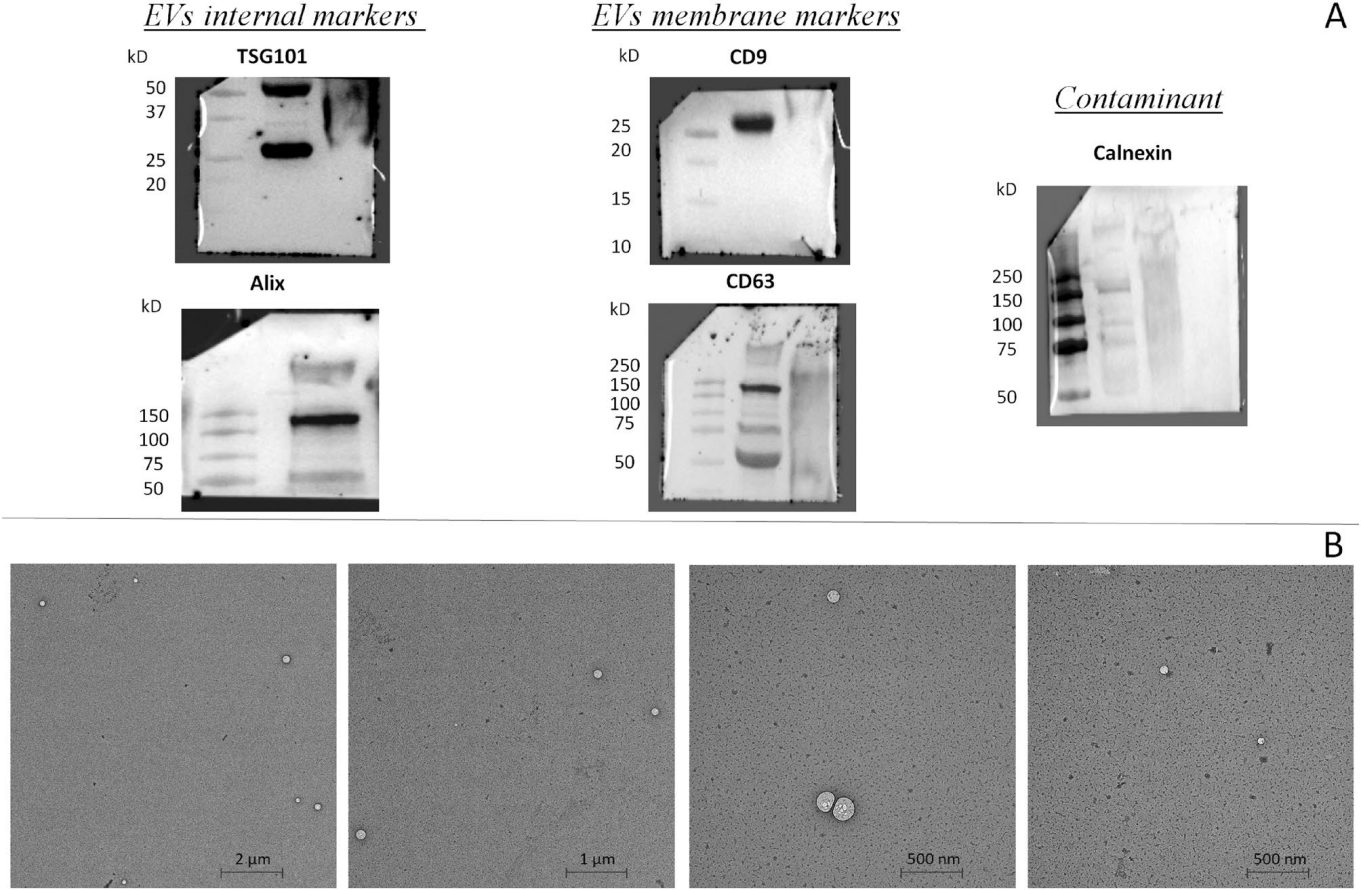
EVs isolated (pool of FF collected in An and pO follicles in both seasons) were characterized by: (**A**) Western Blot for EVs internal markers (TSG101 and Alix), membrane markers (CD9 and CD63); and Calnexin as marker of cell contamination in EVs preparation. (**B**) Transmission Electron Microscopy that revealed typical morphologies characteristic of vesicle (scale bar: from 500 nm to 2 µm).

EVs were isolated as described in the Materials and Methods section from An and pO follicles in the two breeding seasons and analyzed by Nanoparticle Tracking Analysis (NTA) to characterize their size and concentration. EVs were characterized according to MISEV2018 guidelines, by Nanoparticle Tracking Analysis (NTA), Transmission Electron Microscopy (TEM) and Western blotting (WB)^39^. EVs showed similar size distribution (mean size from 160 to 212 nm), but particle concentration was higher in antral follicles samples (An: 2.17e + 11 particles/ml in BS and 1.01e + 11 particles/ml in NBS; pO: 2.51e + 10 particles/ml in BS and 5.40e + 10 particles/ml in NBS), as shown in Supplementary file S1. WB showed the presence of specific EVs marker such as CD9, CD63, Alix and TSG101 and a negligible contamination of Calnexin (Fig. 1A). Observation by electron microscope revealed that preparations contained EVs (Fig. 1B).

**Figure 2:**
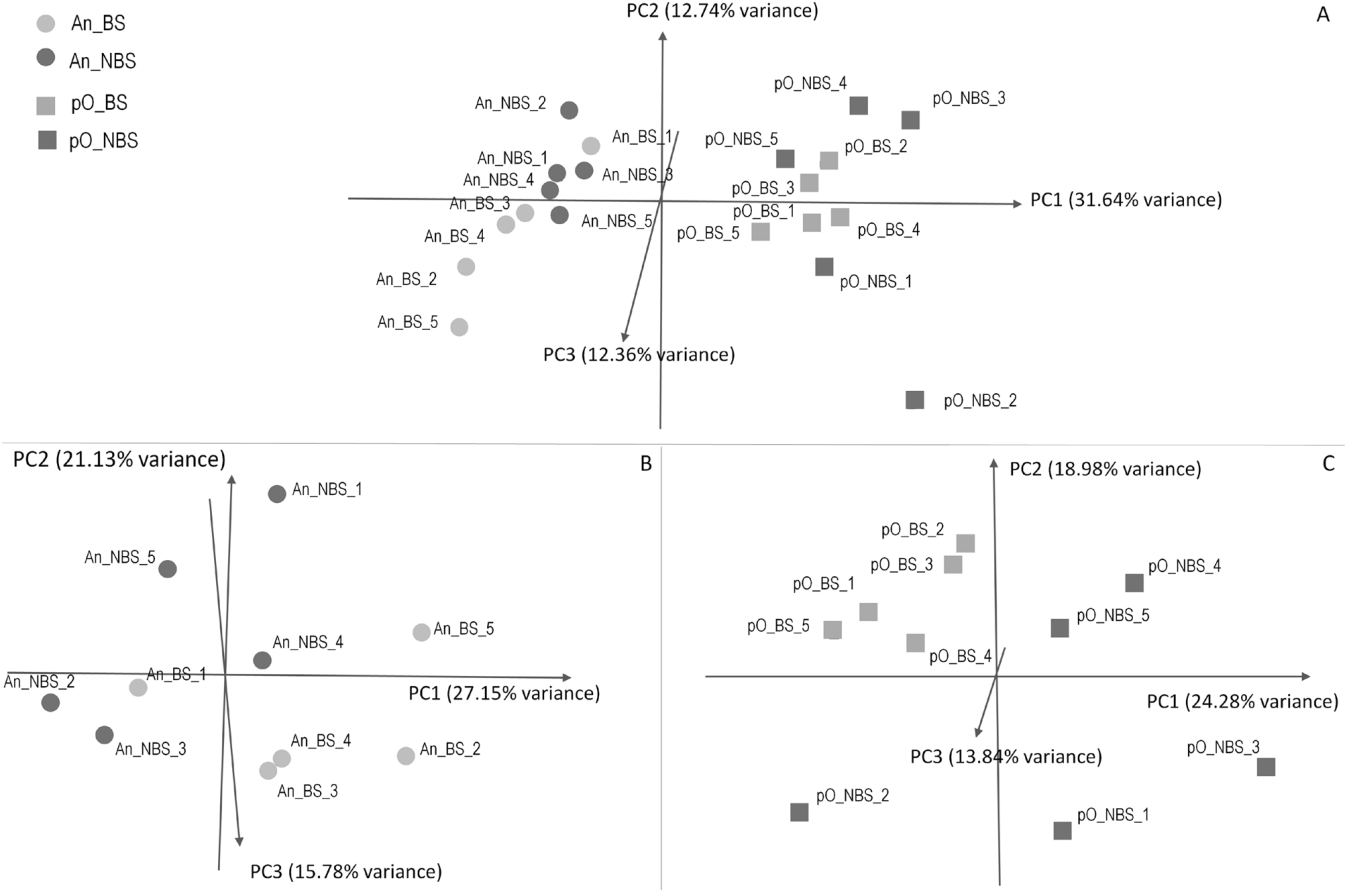
Principal Component Analysis (PCA) for: (**A**) 317 miRNAs counted in all Antral (An) and preovulatory (pO) samples, B) 467 miRNAs counted in all An samples and (**C**) 322 miRNAs counted in all pO samples.

About 37.7 and 23.8 million reads were sequenced for EVs isolated from An and pO follicles, 4.8% and 1.6% of which were assigned to miRNAs in An and pO FFs, respectively (Supplementary file S2). EVs isolated from An and pO FFs showed a total of 1335 miRNAs (538 known Bos taurus miRNAs, 324 homologous to known miRNAs from other species and 473 new candidate miRNAs). Principal component analysis of the 317 miRNAs, counted at least once in all 20 samples, clearly separated An and pO samples but only partially NBS and BS (Fig. 2A).

However, within each developmental stage (An and pO), the analysis of 467 and 322 miRNAs respectively present in all An and pO samples showed a clear separation between BS and NBS, especially for pO (Fig. 2B and C). FF isolated from follicles at different developmental stages contained EVs that showed a specific miRNA cargo with 413 differentially expressed miRNAs (DE-miRNAs) (False Discovery Rate FDR < 0.05) between An and pO (Supplementary file S3).

Considering only the most significant DE-bta-miRNA (FDR < 10exp-6, LogFC >|2|, *n* = 18), 14 miRNAs (LogFC > 2: bta-miR-132-3p, bta-miR-194, bta-miR-215, bta-miR-708, bta-miR-129-5p, bta-miR-193b, bta-miR-191; LogFC < 2: bta-miR-101, bta-miR-130a, bta-miR-29c, bta-miR-378; bta-miR-148d, bta-miR-1246, bta-miR-335), homologous with human miRNAs, target 3’UTR of 273 genes with high confidence (*P* value < 0.001), (Supplementary file S4). Gene Ontology analysis of target genes identified pathways mainly related to glucuronidation, lipid and steroid metabolic process, response to steroid and oestradiol stimulus (Table 2).

**Table 2:** Gene Ontology (GO) analysis results of target genes for the most significant differentially expressed miRNAs (DE-bta-miRNAs) between An and pO (FDR < 10exp-6, LogFC >|2|, *n* = 18).

GOID	Associated genes found	GOTerm	Term *P* value*
GO:0,052,696	[UGT1A1. UGT1A10. UGT1A3. UGT1A4. UGT1A6. UGT1A7. UGT1A8. UGT1A9]	Flavonoid glucuronidation	3.98E-14
GO:0,052,697	[UGT1A1. UGT1A10. UGT1A3. UGT1A4. UGT1A6. UGT1A7. UGT1A8. UGT1A9]	Xenobiotic glucuronidation	6.19E-12
GO:0,006,789	[UGT1A1. UGT1A10. UGT1A4. UGT1A6. UGT1A7. UGT1A8]	Bilirubin conjugation	2.18E-10
GO:0,070,980	[UGT1A1. UGT1A10. UGT1A4. UGT1A6. UGT1A7. UGT1A8]	Biphenyl catabolic process	2.18E-10
GO:0,033,013	[ABCB6. CPOX. TCN1. UGT1A1. UGT1A10. UGT1A4. UGT1A6. UGT1A7. UGT1A8]	Tetrapyrrole metabolic process	1.73E-05
GO:0,045,833	[CRTC3. NCOR1. SIRT4. SORL1. UGT1A1. UGT1A10. UGT1A4. UGT1A6. UGT1A7. UGT1A8]	Negative regulation of lipid metabolic process	4.38E-05
GO:0,045,939	[UGT1A1. UGT1A10. UGT1A4. UGT1A6. UGT1A7. UGT1A8]	Negative regulation of steroid metabolic process	1.36E-04
GO:0,045,471	[CHRNB2. GNRH1. HPGD. POLG2. TUFM. UGT1A1. UGT1A10. UGT1A4. UGT1A6. UGT1A7. UGT1A8]	Response to ethanol	2.14E-04
GO:0,071,392	[UGT1A1. UGT1A10. UGT1A4. UGT1A6. UGT1A7. UGT1A8]	Cellular response to estradiol stimulus	6.41E-04
GO:0,006,953	[CD163. UGT1A1. UGT1A10. UGT1A4. UGT1A6. UGT1A7. UGT1A8]	Acute-phase response	6.53E-04
GO:0,008,210	[UGT1A1. UGT1A10. UGT1A4. UGT1A6. UGT1A7. UGT1A8]	Estrogen metabolic process	7.14E-04
GO:0,071,385	[DDIT4. UGT1A1. UGT1A10. UGT1A4. UGT1A6. UGT1A7. UGT1A8]	Cellular response to glucocorticoid stimulus	1.09E-03
GO:0,001,523	[KDM5A. UGT1A1. UGT1A10. UGT1A3. UGT1A4. UGT1A6. UGT1A7. UGT1A8. UGT1A9]	Retinoid metabolic process	1.15E-03
GO:0,032,355	[HPGD. KCNJ11. MBD1. MYCBP2. UGT1A1. UGT1A10. UGT1A4. UGT1A6. UGT1A7. UGT1A8]	Response to estradiol	1.65E-03
GO:0,032,466	[CHMP4C. E2F8. TEX14]	Negative regulation of cytokinesis	2.22E-03
GO:0,051,187	[AHCY. UGT1A1. UGT1A10. UGT1A4. UGT1A6. UGT1A7. UGT1A8]	Cofactor catabolic process	3.97E-03
GO:0,031,960	[CPNE1. DDIT4. GNRH1. MYCBP2. UGT1A1. UGT1A10. UGT1A4. UGT1A6. UGT1A7. UGT1A8]	Response to corticosteroid	7.33E-03
GO:0,021,952	[CDH11. CHRNB2. DCC. MYCBP2]	Central nervous system projection neuron axonogenesis	9.26E-03
GO:0,016,999	[CBR4. MTHFD1L. UGT1A1. UGT1A10. UGT1A4. UGT1A6. UGT1A7. UGT1A8]	Antibiotic metabolic process	1.34E-02
GO:0,006,474	[NAA25. NAA30. NAA50]	N-terminal protein amino acid acetylation	3.17E-02
GO:0,032,465	[BIRC6. CALM2. CHMP4C. E2F8. SPAST. TEX14]	Regulation of cytokinesis	3.26E-02
GO:0,071,466	[DDIT4. UGT1A1. UGT1A10. UGT1A3. UGT1A4. UGT1A6. UGT1A7. UGT1A8. UGT1A9]	Cellular response to xenobiotic stimulus	3.28E-02

Gene ontology IDs (GO-ID), gene ontology terms (GO-term), associated genes found and corrected p-values as determined by ClueGO (http://apps.cytoscape.org/apps/cluego) are indicated.

*Corrected with Bonferroni step down.

Although a subset of the most significant DE-miRNAs between pO and An targets genes which function is directly related to oestrogen response, these miRNAs did not show a common variation in both development stages in relation to breeding seasons (Supplementary file S5). Comparison of miRNA cargo of EVs isolated in the BS and NBS in both developmental stages did not show any DE-miRNAs, probably due to the high heterogeneity of miRNA expression between An and pO follicles. However, when An or pO developmental stages were considered separately, an alteration in miRNA expression between seasons was found, with 14 and 12 DEmiRNAs between NBS and BS for An and pO, respectively (Supplementary file S3 and Table 3).

**Table 3:** Differentially expressed miRNAs (DE-miRNAs) between non-breeding (NBS) and breeding season (BS) in antral (An) and preovulatory (pO) follicles.

An (NBS vs. BS)				pO (NBS vs. BS)			
miRNA	logFC	FDR	Homologous human miRNA (miRBase)	MiRNA	logFC	FDR	Homologous human miRNA (miRBase)
Novel:chi-miR-24-5p	1.8	0.001	hsa-miR-24–1-5p	Novel:NC_037560.1_35403	5.3	0.002	None
Novel:hsa-miR-4783-5p	− 2.3	0.006	hsa-miR-4783-5p	Novel:NC_037567.1_44155	− 5.2	0.016	None
Novel:NC_037560.1_35933	2.3	0.011	None	bta-let-7f.	− 1.4	0.016	hsa-let-7f.-5p
bta-miR-2285bf	− 5.7	0.012	None	Novel:NC_037564.1_40326	− 1.9	0.016	None
bta-let-7e	2.0	0.012	hsa-let-7e-5p	bta-miR-381	− 1.9	0.016	hsa-miR-381-3p
Novel:hsa-miR-3185	2.2	0.012	hsa-miR-3185	Novel:hsa-miR-3714	− 2.1	0.043	hsa-miR-3714
Novel:NC_037560.1_35256	− 5.0	0.019	None	Novel:hsa-miR-6077	− 3.9	0.043	hsa-miR-6077
Novel:hsa-miR-3689d	1.5	0.024	hsa-miR-3689d	Novel:hsa-miR-4515	1.9	0.043	hsa-miR-4515
bta-miR-669	− 1.3	0.027	hsa-miR-574-3p	Novel:NC_037552.1_22617	− 2.6	0.043	None
Novel:hsa-miR-766-5p	− 3.3	0.033	hsa-miR-766-5p	Novel:NC_037569.1_46660	− 2.1	0.043	None
Novel:NC_037556.1_29844	2.2	0.034	None	bta-miR-487b	− 4.6	0.043	hsa-miR-487b-3p
Novel:NC_037547.1_8396	1.8	0.037	None	Novel:hsa-miR-4428	− 2.2	0.048	hsa-miR-4428
bta-miR-193a	− 1.6	0.045	hsa-miR-193a-5p				
Novel:hsa-miR-6741-5p	− 1.0	0.045	hsa-miR-6741-5p				

For each DEmiRNAs, logFC = log Fold Change, False Discovery Rate FDR < 0.05 and homologous human miRNA were reported.

Seven out of the 14 De-miRNAs in An follicles and 10 of the 12 DE-miRNAs in pO follicles were reduced in NBS.

**Figure 3:**
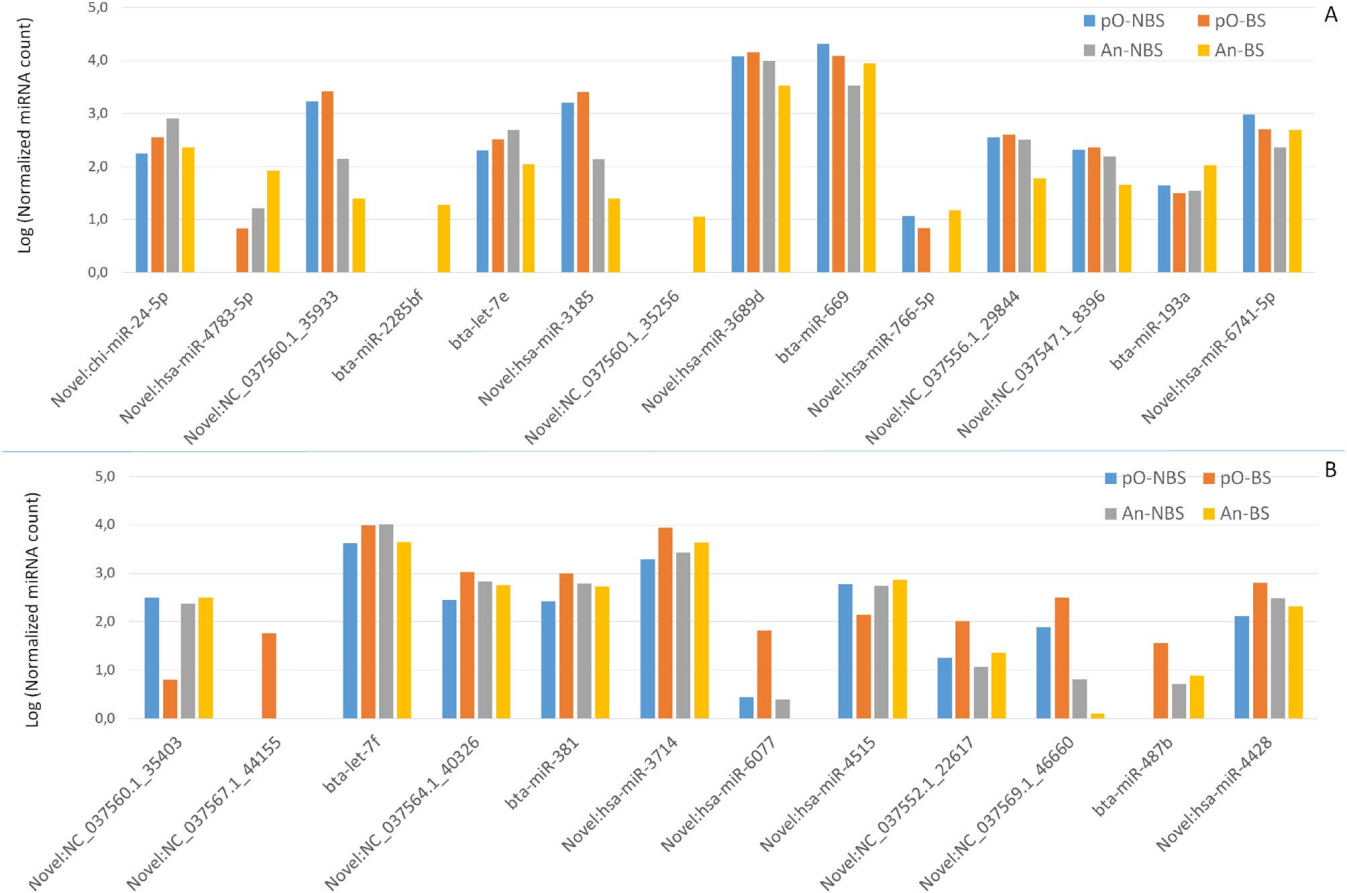
Average expression of differentially expressed miRNAs (DE-miRNAs) differing between non-breeding season (NBS) and breeding season (BS) in (**A**) antral follicles and (**B**) pO follicles. For each combination An and pO follicles in NBS and BS, the Log normalized miRNAs count was reported.

Surprisingly, the variation of these DE-miRNAs in EVs in the two breeding seasons showed mainly an opposite direction in An and pO follicles, i.e. when a specific miRNA increases from NBS to BS in An follicle the same miRNA decreases from NBS to BS in pO follicle and viceversa (Fig. 3).

Finally, GO analysis of target genes for human homologous DE-miRNAs between NBS and BS (*P* value < 0.001), identified pathways related to interleukin-6 production and secretion in An follicles and synaptic transmission and cell adhesion in pO follicles, respectively (Table 4).

**Table 4:** Gene Ontology (GO) analysis results of target genes for the differentially expressed miRNAs (DE-bta-miRNAs) between non-breeding (NBS) and breeding season (BS) in antral (An) and preovulatory (pO) follicles.

GOID	Associated genes found	GOTerm	Term *P* value*
**An (NBS vs. BS)**			
GO:0,072,604	[C1QTNF3, C1QTNF4, DDX58, SYT11, TLR8]	Interleukin-6 secretion	0.011
GO:0,001,755	[NRTN, PHACTR4, SEMA3A, SEMA4C, SEMA4G]	Neural crest cell migration	0.012
GO:0,150,079	[SYT11, TAFA3, TNFRSF1B]	Negative regulation of neuroinflammatory response	0.012
GO:0,032,675	[C1QTNF3, C1QTNF4, DDX58, MYD88, SYT11, TLR1, TLR8]	Regulation of interleukin-6 production	0.029
GO:0,050,710	[C1QTNF3, SYT11, TLR8]	Negative regulation of cytokine secretion	0.033
GO:0,061,900	[SYT11, TAFA3, TLR8]	Glial cell activation	0.04
GO:0,032,635	[C1QTNF3, C1QTNF4, DDX58, MYD88, SYT11, TLR1, TLR8]	Interleukin-6 production	0.045
GO:0,015,459	[KCNAB2, KCNE5, SYNGAP1]	Potassium channel regulator activity	0.047
**pO (NBS vs. BS)**			
GO:0,051,966	[ADORA1, DISC1, DRD2]	Regulation of synaptic transmission, glutamatergic	0.033
GO:0,051,895	[BCAS3, DUSP22, LRP1]	Negative regulation of focal adhesion assembly	0.049
GO:0,150,118	[BCAS3, DUSP22, LRP1]	Negative regulation of cell-substrate junction organization	0.049

Gene ontology IDs (GO-ID), gene ontology terms (GO-term), associated genes found and corrected *p*-values as determined by ClueGO (http://apps.cytoscape.org/apps/cluego) are indicated.

*Corrected with Bonferroni step down.

## Discussion

The present study aimed to investigate whether season might influence the EVs miRNA profile in FF of both antral and pre-ovulatory follicles in buffalo. A further objective was to investigate the FF-EVs-miRNA cargo of An and pO buffalo follicles, in order to understand possible changes occurring at different developmental stages and their potential role in modulating follicle development. This is the first report on the characterization of miRNAs contained in EVs of FF in this species, demonstrating that specific miRNAs may be involved in regulating follicular development and modulating seasonal effects on oocyte competence.

To achieve these goals, we isolated the EVs using a previously published method based on polymer precipitation of EVs from bovine FF that showed a high degree of purity in EVs^26^. In accordance with earlier findings in bovine^34,40^, a similar size distribution in buffalo FF-EVs was observed. An follicles showed a higher EVs concentration compared to pO follicles, that was in accordance with a previous study that reported a higher EVs concentration in FF isolated from small versus large bovine follicle^40^. A high similarity of buffalo and bovine FF-EVs-miRNA cargo has also been observed with several shared miRNAs whose expression varies similarly during different stages of follicular development^40^. GO analysis of the target genes of miRNAs showing the most significant variation in EVs isolated from An e pO follicles revealed alteration in genes which function is related to hormone regulation, such as response to oestradiol and oestrogen metabolism. It is known that oestradiol supports follicular and oocyte growth, antrum formation, and follicular function^41^. Interestingly, miR-132-3p and miR193-b, which are highly enriched in EVs from pO follicles, were observed to be highly abundant in human follicular fluids, and to regulate oestradiol and progesterone concentration, respectively, in a steroidogenic human granulosa-like tumour cell line^30^. In addition, other three miRNAs (miR191, miR-29c and miR378) whose expression varies consistently between An and pO follicles, were differentially expressed in the follicular fluid of women with endometriosis compared to healthy patients^42^, and other three miRNAs (miR132-3p, miR-708, and miR-335) were differentially expressed between the follicular fluid of preovulatory dominant and subordinate follicles^43^, indicating a potential role in the selection of follicles. Interestingly, the expression of these miRNAs varies similarly also in granulosa cells and theca cells^44^.

The present study also characterizes miRNAs content of EVs isolated from FF in An and pO follicles in the BS and NBS in order to evaluate if a seasonal effect in FF-EVs-miRNA content exists. Indeed, EVs isolated from the FF of An and pO follicles were enriched in specific miRNAs in the two breeding seasons; in particular, 14 and 12 DE-miRNAs were identified in An follicles and pO follicles, respectively. The evidence of seasonal differences in miRNA profile of FF-EVs suggests that these changes may account for the reduced oocyte developmental competence recorded during NBS. Indeed, both cleavage and blastocyst rates significantly decreased during the NBS in the present study, confirming previous observations^8,9^. Furthermore, despite the limitations due to the low numbers observed, it is intriguing that cumulus expansion was impaired in pre-ovulatory follicles during NBS.

Among the DE-miRNA between seasons found in the present work there are let-7e and let7f. that showed different expression respectively in An and pO follicles. Interestingly, few members of the let7 family were also identified among DE-miRNAs in the sheep ovary in relation to season^35^. Furthermore, a role of the let-7 miRNA family in granulosa cell programmed death and follicular atresia was demonstrated in swine^44^ and miR-let-7f. was found to modulate the expression of CYP19AI transcript in cultured buffalo granulosa cells^45^.

Interestingly, the GO analysis revealed that seasonal variation in An follicles involves miRNAs targeting genes which function is related to interleukin-6 (IL-6) production and secretionThis is very interesting as IL-6 is a cytokine characterized by both pro-inflammatory and anti-inflammatory activities, that modulates intraovarian functions at different levels, by regulating steroidogenesis, angiogenesis, as well as granulosa cell function^46,47^ and follicular development^48^. Interestingly, IL-6 in follicular fluid was observed to reduce embryo fragmentation and to improve the rates of clinical pregnancy in human^49^. Furthermore, a regulatory role of IL-6 on cumulus expansion and oocyte developmental competence was demonstrated in the mouse^50^.

In pO follicles, GO analysis of target genes for DE-miRNAs showed involvement in the regulation of focal adhesion assembly and cell-substrate junction organization. These pathways are important to ensure the establishment of a correct bidirectional communication between the oocyte and surrounding somatic cells, essential for proper oocyte growth and maturation^51–53^. This is possible through paracrine factors and direct cell–cell communication via gap junctions located at the sites of cell contact. It was demonstrated that a focal adhesion kinase is involved in regulating the adherens junction formation between oocyte and somatic cells in the mouse, hence playing a pivotal role on oocyte-follicle communication^54^.

Finally, it is worth noting that the analysis of the DE-miRNAs expression level between NBS and BS has shown an opposite pattern in An and pO follicles. Some DE-miRNAs were observed to increase in An while decreasing in pO from NBS to BS and viceversa. In addition, some of these miRNAs (miR487 and miR669) were previously observed to change during folliculogenesis, in response to cytokines or in atretic follicles^55,56^. Taking into account that a cyclic temporal miRNA expression has been observed during follicle development in bovine, with several miRNAs increasing in expression until the mid-luteal phase, and decreasing in the late follicular phase^22^, the alteration observed in NBS and BS for An and pO follicles could probably be related to an acceleration or delay of follicular development in the two breeding seasons.

## Conclusion

In conclusion, it was demonstrated that buffalo follicular fluid contains EVs which miRNA cargo is strictly related to the follicular developmental stage. Passing from An to pO different miRNAs could modulate the expression of genes which function was mainly associated to estradiol and steroid metabolism. It was also shown that season influences the miRNA content of EVs isolated from both An and pO follicles. In particular, EVs isolated from An follicles showed also misregulation of miRNAs that potentially influence IL-6 expression and secretion in FF, while those isolated from pO follicles contained miRNAs targeting genes involved in regulation of focal adhesion assembly and cell-substrate junction organization. This is the first report on the characterization of miRNAs contained in EVs isolated from buffalo follicular fluid and opens the way to future studies to develop in vitro corrective strategies for improving oocyte developmental competence during NBS in this species. In fact, knowing that the miRNA cargo of FF-EVs undergoes seasonal variations lays the basis for future studies aimed at evaluating whether enriching the in vitro maturation medium with either FF-EVs from the BS or specific DE miRNAs would improve oocyte competence during the NBS.

## Materials and methods

### Ethics

The experimental design and animal treatments were approved by the Ethical Animal Care and Use Committee of the University of Naples Federico II, Italy (PG/2029/007,004 of 2 July 2019). The biological samples were collected from animals slaughtered after mechanical stunning by captive bolt to ensure a human painless end. All methods were performed in accordance with the relevant guidelines and regulations and the study is reported in accordance with ARRIVE guidelines.

### Collection of follicular fluid

The study was carried out in Campania region, Southern Italy (latitude 40.5°–41.5° N and longitude 13.5–15.5) in October, i.e. autumn (BS) and January, i.e. mid-winter (NBS). Follicular fluid (FF) of antral follicles (< 0.5 cm) and pre-ovulatory follicles (≥ 1 cm diameter) was collected from buffalo ovaries recovered at a local abattoir (Real Beef s.r.l., Flumeri (AV), Italy) from animals slaughtered under national food hygiene regulations, and transported to the laboratory in physiological saline buffer supplemented with 150 mg/L kanamycin at 30–35 °C within 4 h after slaughter. In particular, in order to collect FF from antral follicles and cumulus-oocyte-complexes for in vitro embryo production, 76 cyclic multiparous Italian Mediterranean Buffalo cows with a mean weight and age of 546.3 ± 11.8 kg and 5.5 ± 0.5 years, over a total of 10 replicates (5/season) were used. Cyclic ovarian activity was assessed by two clinical examinations carried out 12 days apart before slaughter, to detect the presence of a follicle greater than 1 cm and/or corpus luteum on the ovary. In order to collect FF from pre-ovulatory follicles, animals (*n* = 7 and 9, respectively in BS and NBS) with a mean weight and age of 555.2 ± 13.2 kg and 5.5 ± 0.3 years were synchronized by Ovsynch^57^, consisting in GnRH administration of 0.012 mg buserelin acetate (Receptal, Intervet, Milan, Italy) im on Day 0, followed by 0.524 mg of synthetic prostaglandin (Cloprostenol, Estrumate, Schering-Plough Animal Health, Milan, Italy) on Day 7 and an additional 12 mg buserelin acetate on Day 9. Animals that ovulated after the first GnRH (*n* = 5/season) were slaughtered 18 h after the last GnRH, i.e. in proximity of ovulation. The follicle size was calculated as the mean of two perpendicular diameters of each pre-ovulatory follicle.

The FF of individual preovulatory follicles and of 2–8 mm antral follicles (pool of 20) was aspirated by a syringe with a 21 G gauge needle and poured into a petri dish for a quick search and assessment of the COC. Afterwards FF was transferred into a vial and centrifuged at 300 × *g* for 10 min at 4 °C to separate the follicular fluid and the follicular cells. The FF was centrifuged again at 2000 g for 10 min and at 16500 g × 30 min and supernatant was stored at − 80 °C until RNA isolation.

After morphological assessment^8^, Grade A and B COCs from antral follicles, considered suitable for in vitro embryo production (IVEP), were in vitro matured, fertilized and cultured up to the blastocyst stage (*n* = 140 and 138, respectively in the BS and NBS), in order to assess developmental competence.

### Extracellular vesicle (EVs) isolation from Follicular Fluids

EVs were isolated from FF through Exoquick precipitation. In a previous study in cattle, this method was proven as efficient as ultracentrifugation in separating EVs and non-EVs fraction from FF^26^. About 200 µl of FF from five biological replicates, collected from An (*n* = 20) and pO (*n* = 1) follicles, in the breeding (BS) and non-breeding (NBS) seasons, were used for EVs isolation.

### Nanoparticle tracking analysis (NTA)

Number, size and concentration of isolated EVs were determined by Nanoparticle tracking analysis (NTA) performed according to manufacturer’s instructions using a NanoSight NS300 system (Malvern Technologies, Malvern, UK) configured with 532 nm laser. All samples were diluted in filtered PBS to a final volume of 1 ml. Ideal measurement concentrations were found by pre-testing the ideal particle per frame value (20–100 particles/frame). Following settings were set according to the manufacturer’s software manual. A syringe pump with constant flow injection was used and three videos of 60 s were captured and analysed with NTA software version 3.2. From each video, the mean, mode, and median EVs size was used to calculate samples concentration expressed in nanoparticles/mL.

### Western blotting

EVs isolated (pool of FF isolated in An and pO follicles in both seasons) were solubilized in Laemmli buffer for 5 min at 95 °C; 8uL of reduction buffer were added at 32uL of EVs. Sample was separated by SDS-PAGE (4–20%, Mini-Protean TGX Precast protein gel, Bio-Rad) and transferred onto a nitrocellulose membrane (BioRad, Trans-Blot Turbo). Blocking step was performed to saturate nonspecific sites, 1 h with 5% (w/v) BSA in T-TBS (tris-buffered saline: 150 mM NaCl, 20 mM TrisHCl, pH 7.4, and 0.5% Tween 20). Membranes were incubated overnight at 4 °C with anti-CD9 (1:1000, BD Pharmingen), anti-CD63 (1:1000; BD Pharmingen, San Jose, CA, USA), anti-Alix (1:1000, Santa Cruz, CA, USA), anti-TSG101 (1:1000, Novus Bio, Centennial, CO, USA) and anti-Calnexin (1:1000, Santa Cruz). After washing with T-TBS, membranes were incubated with the horseradish peroxidase-conjugated (Jackson ImmunoResearch, Tucker, GA, USA) secondary antibodies diluted 1:3000 for 1 h. After washing, the signal was detected using Bio-Rad Clarity Western ECL Substrate (Bio-Rad) and imaged using a Chemidoc XRS + (BioRad).

### Transmission electron microscopy

EVs isolated (pool of FF isolated in An and pO follicles in both seasons) were fixed in a mixture of 2% paraformaldehyde and 2.5% glutaraldehyde in 0.1 M sodium cacodylate buffered solution at pH 7.4 for 1 h at room temperature. After washing in the same buffer, samples were post-fixed in 1% OsO4, 1.5% potassium ferrocyanide in 0.1 M cacodylate for 1 h in dark condition on ice. After several washings in distilled water, samples were stained with 0.5% uranyl acetate in water overnight at 4 °C and, finally, were dehydrated in a graded ethanol series (30, 50, 70, 80, 90, 96% for 5 min each and washed three times with absolute ethanol for 10 min each). The samples were infiltrated with ethanol and resin (Araldite-Epon) at volumetric proportions of 1:1 for 2 h, and then in 100% Epon twice for 1 h each and, finally, polymerized at 60 °C for 48 h. Sectioning was performed using an Ultracut E microtome (Reichert, Austria). Sections of 70 nm were collected on 300-mesh uncoated copper grid and observed with a Zeiss LEO 912ab Energy Filtering TEM operating at 120 kV. Digital images were acquired using a CCD-BM/1 K system operating with the iTEM (Olympus Soft Imaging Solutions).

### RNA isolation

Total RNA was extracted from isolated EVs. Total RNA was isolated by NucleoSpin miRNA kit (Macherey–Nagel, Germany), following the protocol in combination with TRIzol (Invitrogen, Carlsbad, CA, USA) lysis with small and large RNA in one fraction (total RNA). Concentration and quality of RNA were determined by Agilent 2100 (Santa Clara, CA, USA). The isolated RNAs were stored at − 80 °C until use.

### Library preparation and sequencing

In total, 20 libraries of small RNA were obtained from five isolated EVs per group (*n* = 5) of two developmental stagse (An and pO) in both seasons (BS and NBS). Small RNA libraries were prepared using TruSeq Small RNA Library Preparation kit, according to manufacturer’s instructions (Illumina). Small RNA (sRNAs) libraries were pooled together and purified with Agencourt AMPure XP (Beckman, Coulter, Brea, CA) (1 Vol. sample: 1.8 Vol. beads) twice. Concentration and profile of libraries were determined by Agilent 2100 Bioanalyzer before library sequencing on a single lane of Illumina Novaseq 6000 (San Diego, CA, USA).

### Data analysis

Illumina raw sequences were quality checked with FastQC (http://www.bioinformatics.babraham.ac.uk/projects/fastqc/) and trimmed with Trimmomatic (version 0.32)^58^, then miRDeep2 (miRDeep2 (version 2.0.0.5)^59^ was used for miRNA detection and discovery. Known miRNAs available at MirBase (http://www.mirbase.org/) were used to support miRNA identification. In particular, Bos taurus miRNAs were input to support known miRNA detection and miRNAs from related species (sheep, goat and human) were input to support novel miRNA identification. All the identified miRNAs were quantified using the miRDeep2 quantifier module. The Bioconductor edgeR package (version 2.4) was used to identify statistically significant differential expression between groups of samples (false discovery rate [FDR] < 0.05)^60^. Predicted miRNA gene targeting of differentially expressed Bos taurus miRNAs (DE-miRNAs) was performed with miRWalk2.0^61^, using homologous human miRNAs as input identifiers.

Target genes were submitted to GO analysis. GO classification of the DEGs was performed according to canonical GO categories, using the Cytoscape (version.3.2.1) plug-in ClueGO (version 2.3.5) which integrates GO and enhances biological interpretation of large lists of genes^62^. MicroRNA cluster analysis was performed with Genesis (version1.8.1)^63^.

### In vitro embryo production

Reagents were acquired from Sigma Chemical Company (Milano, Italy) unless differently specified. Grade A and B COCs retrieved by follicular aspiration were washed in HEPES-buffered TCM199 supplemented with 10% fetal calf serum (FCS) and in vitro matured, fertilized and cultured to the blastocyst stage. In vitro maturation (IVM) methods briefly reported below were in part reproduced from Gasparrini et al.^64^. Briefly, COCs were allocated to 50 µL drops (10 per drop) of IVM medium, i.e. in TCM199 buffered with 25 mM sodium bicarbonate and supplemented with 10% FCS, 0.2 mM sodium pyruvate, 0.5 µg/mL FSH, 5 µg/mL LH, 1 µg/mL 17 β-oestradiol and 50 µg/mL kanamycin, and incubated at 38.5 °C for 21 h in a controlled gas atmosphere of 5% CO2 in humidified air. In vitro fertilization (IVF) and culture (IVC) methods were reproduced from Di Francesco et al.^9^. Frozen straw from an IVF tested bull were thawed at 37 °C for 40 s and sperm were selected by centrifugation (25 min at 300 g) on a discontinuous Percoll gradient (45 and 80%). The sperm pellet was re-suspended to a final concentration of 2 × 106 mL-1 in the IVF medium, consisting of Tyrode albumin lactate pyruvate supplemented with 0.2 mM penicillamine, 0.1 mM hypotaurine and 0.01 mM heparin. Insemination was performed in 50 µL drops of IVF medium under mineral oil (5 oocytes per drop) at 38.5 °C under humidified 5% CO2 in air. Twenty hours after IVF, putative zygotes were denuded of cumulus cells by gentle pipetting and transferred to 20 µL drops of IVC medium, i.e. synthetic oviduct fluid (SOF) including essential and non-essential amino acids and 8 mg/mL bovine serum albumin^65^. Culture was carried out under humidified air with 5% CO2, 7% O2 and 88% N2 at 38.5 °C. On day 5 and 7 post-insemination the cleavage and blastocyst rates were assessed.

## Supplementary Information

Supplementary Information.

## Data Availability

Small-RNA-Seq data are available in the Sequence Reads Archive (SRA), BioProject accession number, PRJNA771497. Novel miRNA precursors and novel miRNA mature sequences are reported in Supplementary file S5 and S6.
