# Supplementary material for: Variations of follicular fluid extracellular vesicles miRNAs content in relation to development stage and season in buffalo
Source: Sci Rep. 2022 Sep 1;12:14886. doi: 10.1038/s41598-022-18438-8 (PMC9437019; doi:10.1038/s41598-022-18438-8)
Supplement: Supplementary file 1 — Supplementary Information. [file 41598_2022_18438_MOESM1_ESM.pdf]

Variations of follicular fluid extracellular vesicles miRNAs content in relation to development stage and season in buffalo

Emanuele Capra, Michal Andrzej Kosior, Natascia Cocchia, Barbara Lazzari, Chiara Del Prete, Valentina Longobardi, Flavia Pizzi, Alessandra Stella, Roberto Frigerio, Marina Cretich, Anna Lange Consiglio, Bianca Gasparrini

Supplemental file S1.

Measurement of size and concentration with NanoSight instrument for extracellular vesicle (EVs) isolated from follicular fluid (FF) from antral (An) and preovulatory (pO) follicles collected in the breeding (BS) and non-breeding (NBS) season. Analysis was performed on FF pool of five replicate for each condition.

sample name	FF1-5 (pool FF1-FF5)	FF6-10 (pool FF6-FF10)	FF11-15 (pool FF11-FF15)	FF16-20 (pool FF16-FF20)
Type	An	An	oP	oP
Season	NBS	BS	BS	NBS
Mean Size (nM)	187.0	212.1	159.7	171.6
Mode Size (nM)	148.7	170.5	142.6	126.0
Concentration	1.01exp 11	2.17 exp 11	2.51 exp 10	5.40 exp 10

Supplemental file S2.

Sequencing Statistics for miRNA cargo of extracellular vesicle (EVs) isolated from follicular fluid (FF) collected from antral (An) and preovulatory (pO) follicles in the breeding (BS) and non-breeding (NBS) season.

Sample name	Follicle Type	Season	Reads sequenced	Reads assigned to miRNAs	%
An_NBS_1	An	NBS	28341180	1846840	6.516454
An_NBS_2	An	NBS	30720758	2462608	8.016104
An_NBS_3	An	NBS	27697910	772780	2.79003
An_NBS_4	An	NBS	45848478	1857158	4.050643
An_NBS_5	An	NBS	57126912	856959	1.500097
An_BS_1	An	BS	37177220	2605528	7.008399
An_BS_2	An	BS	42897940	1698537	3.959484
An_BS_3	An	BS	28752136	1565074	5.443331
An_BS_4	An	BS	25428560	1161772	4.568768
An_BS_5	An	BS	53465444	2366966	4.427095
pO_NBS_1	pO	NBS	11841854	171287	1.446454
pO_NBS_2	pO	NBS	27921474	374102	1.339836
pO_NBS_3	pO	NBS	20072060	206734	1.029959
pO_NBS_4	pO	NBS	24551838	358812	1.461447
pO_NBS_5	pO	NBS	29442156	735623	2.498536
pO_BS_1	pO	BS	34901768	432487	1.239155
pO_BS_2	pO	BS	22521000	441941	1.962351
pO_BS_3	pO	BS	15970424	362244	2.268218
pO_BS_4	pO	BS	30375546	612867	2.017633
pO_BS_5	pO	BS	20694302	201544	0.973911

Supplemental file S3.
List of differentially expressed miRNAs (DE-miRNAs) between: Sheet1) extracellular vesicle (EVs) isolated from follicular fluid (FF) collected from antral (An) and preovulatory (pO) follicles; Sheet2) EVs isolated form FF collected in the breeding (BS) and non-breeding (NBS) season in both An and preovulatory pO follicles; Sheet3) EVs isolated form FF collected in BS and non-breeding NBS season in An follicles; Sheet1) EVs isolated form FF collected in BS and non-breeding NBS season in pO follicles. For each DE-miRNA, the Log Fold Change (LogFC), P-value and the False Discovery Rate (FDR) were reported.

An_vs_pO	logFC	logCPM	LR	PValue	FDR
Novel:NC_037547.1_9757	-6.63668	8.358043	318.723	2.75E-71	2.80E-68
Novel:hsa-miR-591	-5.75721	8.611969	274.394	1.25E-61	6.37E-59
bta-miR-132	-5.93113	9.120079	267.7784	3.46E-60	1.18E-57
bta-miR-215	-5.08713	10.94187	183.9321	6.71E-42	1.71E-39
Novel:NC_037549.1_16300	-4.02009	11.26963	145.6926	1.52E-33	3.09E-31
Novel:hsa-miR-5190	-4.44314	5.299063	109.3535	1.36E-25	2.31E-23
Novel:hsa-miR-4800-3p	-4.47535	5.272464	107.7999	2.97E-25	4.33E-23
bta-miR-194	-5.92741	5.893439	105.4563	9.70E-25	1.24E-22
bta-miR-148d	2.779767	10.46926	103.3274	2.84E-24	3.22E-22
bta-miR-130a	2.136558	12.86246	101.4517	7.32E-24	7.46E-22
Novel:hsa-miR-1237-3p	2.331861	13.2522	89.59293	2.93E-21	2.71E-19
bta-miR-378	2.3243	13.21874	88.62942	4.76E-21	4.04E-19
Novel:NC_037564.1_41171	4.093478	7.382011	85.61878	2.18E-20	1.71E-18
Novel:NC_037569.1_46549	1.964079	11.78582	82.09888	1.29E-19	9.42E-18
Novel:hsa-miR-6777-5p	3.813754	9.587864	81.88004	1.45E-19	9.74E-18
bta-miR-660	1.959332	11.78546	81.76959	1.53E-19	9.74E-18
bta-miR-92a	1.653854	14.06721	71.90297	2.26E-17	1.35E-15
Novel:hsa-miR-4720-5p	3.86597	4.705886	69.24175	8.71E-17	4.93E-15
bta-miR-129	-3.11419	6.640127	67.22718	2.42E-16	1.29E-14
bta-miR-129-5p	-3.11418	6.640127	67.13959	2.53E-16	1.29E-14
Novel:NC_037558.1_32026	1.886928	10.0255	66.74961	3.08E-16	1.50E-14
bta-miR-378b	2.278083	6.593468	62.80939	2.28E-15	1.05E-13
Novel:NC_037560.1_35933	-4.24115	7.547813	61.15915	5.26E-15	2.28E-13
bta-miR-335	3.69314	4.938104	61.12006	5.37E-15	2.28E-13
Novel:hsa-miR-3185	-4.21282	7.489153	59.9663	9.65E-15	3.93E-13
bta-miR-30d	-1.94392	10.2946	59.71741	1.10E-14	4.29E-13
Novel:hsa-miR-3908	2.918598	6.883669	59.0849	1.51E-14	5.70E-13
Novel:NC_037560.1_34765	-3.10931	5.719019	58.62146	1.91E-14	6.70E-13
Novel:NC_037552.1_22370	-3.10931	5.719019	58.58522	1.95E-14	6.70E-13
Novel:NC_037569.1_46660	-4.90255	4.052275	58.55888	1.97E-14	6.70E-13
bta-miR-103	1.807326	13.67239	57.42472	3.51E-14	1.15E-12
Novel:NC_037552.1_22398	3.608217	4.919149	56.60095	5.34E-14	1.70E-12
Novel:NC_037560.1_35426	-3.10926	9.593978	55.5381	9.17E-14	2.83E-12
bta-miR-191	-2.02952	12.03118	54.46129	1.59E-13	4.75E-12
bta-miR-107	1.809016	12.66837	53.08732	3.19E-13	9.29E-12
Novel:NC_037545.1_824	1.753239	9.346425	52.57454	4.14E-13	1.17E-11
Novel:NC_037545.1_498	1.608249	8.497758	52.27759	4.82E-13	1.33E-11
bta-miR-296-3p	1.668767	10.25456	51.91792	5.79E-13	1.55E-11
bta-miR-1246	2.861689	10.87101	49.52675	1.96E-12	5.11E-11

Novel:NC_037545.1_434	-2.90259	3.683947	48.71763	2.96E-12	7.53E-11
bta-miR-10b	1.192325	18.76372	48.37968	3.51E-12	8.73E-11
bta-miR-29c	2.180603	6.013244	47.44042	5.67E-12	1.37E-10
bta-miR-101	2.08718	9.745675	47.40377	5.78E-12	1.37E-10
Novel:NC_037546.1_2960	4.302524	3.116534	46.95845	7.25E-12	1.68E-10
Novel:hsa-miR-6839-3p	3.532144	9.861999	45.84845	1.28E-11	2.89E-10
Novel:NC_037545.1_2499	-2.48409	11.53725	44.93416	2.04E-11	4.51E-10
Novel:hsa-miR-4447	-2.95361	3.288772	43.9836	3.31E-11	7.18E-10
Novel:hsa-miR-7113-3p	3.231405	10.99723	43.58212	4.07E-11	8.63E-10
bta-miR-193b	-2.11974	10.34292	43.19068	4.97E-11	1.03E-09
Novel:NC_037569.1_46587	-3.06034	4.378462	42.85644	5.89E-11	1.19E-09
Novel:hsa-miR-6068	2.345595	6.749086	42.83347	5.96E-11	1.19E-09
Novel:hsa-miR-6849-3p	-3.65755	3.261915	42.54021	6.92E-11	1.36E-09
Novel:NC_037568.1_45248	-2.20102	9.939781	41.81356	1.00E-10	1.93E-09
Novel:NC_037569.1_47154	2.28833	6.899375	39.75666	2.88E-10	5.43E-09
Novel:NC_037548.1_12850	4.015961	8.779627	39.38871	3.47E-10	6.43E-09
bta-miR-378c	1.646888	6.785008	39.08862	4.05E-10	7.37E-09
Novel:NC_037563.1_39878	2.14221	14.40013	38.43961	5.65E-10	1.01E-08
Novel:NC_037550.1_18158	2.145458	7.851575	37.71233	8.20E-10	1.44E-08
bta-miR-2440	3.604011	5.382454	37.61787	8.61E-10	1.49E-08
Novel:NC_037546.1_4370	2.43561	10.3353	37.13035	1.10E-09	1.88E-08
Novel:NC_037563.1_39396	2.050284	14.43261	36.47246	1.55E-09	2.55E-08
Novel:NC_037566.1_43625	2.119847	6.267013	36.47223	1.55E-09	2.55E-08
Novel:NC_037567.1_44092	2.422311	10.19856	36.05919	1.91E-09	3.10E-08
Novel:NC_037561.1_36931	1.478643	7.136286	35.79747	2.19E-09	3.49E-08
Novel:NC_037547.1_8540	1.291013	13.46986	35.33309	2.78E-09	4.36E-08
Novel:NC_037566.1_43137	2.059353	6.250631	34.75978	3.73E-09	5.76E-08
Novel:chi-miR-145-3p	2.282626	5.590849	34.64626	3.95E-09	5.96E-08
bta-miR-3600	-1.25259	16.21492	34.63591	3.98E-09	5.96E-08
bta-miR-22-3p	-1.25125	16.18844	34.42754	4.42E-09	6.53E-08
Novel:NC_037555.1_27844	2.429389	5.246183	34.33763	4.63E-09	6.74E-08
Novel:NC_037557.1_30640	1.982772	9.616001	33.94761	5.66E-09	8.13E-08
Novel:NC_037562.1_38397	-5.66176	2.643332	33.7644	6.22E-09	8.80E-08
bta-miR-10a	1.914202	10.85694	33.36437	7.64E-09	1.07E-07
Novel:NC_037557.1_30644	1.989552	6.77648	32.53063	1.17E-08	1.62E-07
bta-miR-2285ba	2.458981	3.330188	32.48167	1.20E-08	1.63E-07
bta-miR-301a	-1.70035	6.192571	32.05884	1.50E-08	2.01E-07
bta-miR-151-3p	-1.21258	11.3679	30.71827	2.98E-08	3.95E-07
Novel:NC_037547.1_9951	-1.67905	6.201535	30.0768	4.15E-08	5.43E-07
bta-miR-145	1.888525	7.021458	29.79913	4.79E-08	6.18E-07
bta-miR-708	-3.92347	3.838367	29.62848	5.23E-08	6.67E-07
bta-miR-221	1.41714	9.821362	29.42948	5.80E-08	7.21E-07
bta-miR-423-3p	1.160018	12.04404	29.42755	5.80E-08	7.21E-07
bta-miR-19b	1.763333	9.826208	29.30946	6.17E-08	7.54E-07
bta-miR-361	1.877023	8.067337	29.2945	6.22E-08	7.54E-07
Novel:hsa-miR-7108-3p	1.698869	11.40746	29.23571	6.41E-08	7.68E-07
Novel:NC_037558.1_32071	2.536738	4.801391	28.54043	9.18E-08	1.09E-06
Novel:NC_037569.1_46466	1.416031	9.786759	28.24897	1.07E-07	1.25E-06
Novel:chi-miR-3958-5p	-2.337	5.076879	28.06789	1.17E-07	1.36E-06

bta-miR-1388-3p	1.499351	6.055438	27.97889	1.23E-07	1.40E-06
bta-miR-677	2.377757	5.216157	27.81016	1.34E-07	1.52E-06
Novel:chi-miR-1307-3p	1.536831	6.605067	27.69857	1.42E-07	1.59E-06
Novel:hsa-miR-5589-5p	1.852293	5.046655	27.51757	1.56E-07	1.72E-06
bta-miR-25	0.844593	14.57391	26.72639	2.34E-07	2.57E-06
bta-miR-6119-5p	2.284441	5.058327	26.65569	2.43E-07	2.64E-06
bta-miR-1388-5p	1.307383	7.963253	26.22948	3.03E-07	3.25E-06
bta-miR-320a	1.092762	13.60962	26.20599	3.07E-07	3.26E-06
Novel:NC_037569.1_47107	2.353689	5.065339	25.88265	3.63E-07	3.81E-06
Novel:hsa-miR-4308	1.713866	4.69249	25.85603	3.68E-07	3.82E-06
Novel:hsa-miR-4783-5p	4.03102	3.294775	25.7421	3.90E-07	4.02E-06
Novel:hsa-miR-6814-5p	3.303938	3.255067	25.3228	4.85E-07	4.94E-06
Novel:NC_037549.1_15077	1.078469	7.971634	25.18815	5.20E-07	5.25E-06
bta-miR-210	1.492623	9.376248	24.79978	6.36E-07	6.35E-06
Novel:NC_037551.1_19861	-2.88821	2.945322	24.51328	7.38E-07	7.30E-06
Novel:chi-miR-323b	2.20868	5.530422	24.39742	7.84E-07	7.68E-06
bta-miR-148a	0.862609	13.32269	24.20205	8.67E-07	8.41E-06
Novel:NC_037557.1_31142	1.431357	5.892889	24.17175	8.81E-07	8.41E-06
Novel:NC_037564.1_41452	1.43135	5.892889	24.16856	8.83E-07	8.41E-06
Novel:NC_037569.1_46961	1.431036	5.892889	24.0159	9.55E-07	9.01E-06
bta-miR-19a	1.384603	7.212439	23.90887	1.01E-06	9.44E-06
Novel:NC_037559.1_33968	-1.11423	10.31716	23.80306	1.07E-06	9.89E-06
Novel:hsa-miR-372-5p	-2.86949	2.985965	23.67634	1.14E-06	1.05E-05
Novel:chi-miR-103-3p	1.982387	3.617945	23.50031	1.25E-06	1.14E-05
Novel:NC_037569.1_47983	1.398942	5.9909	23.47107	1.27E-06	1.14E-05
Novel:NC_037557.1_30428	1.396867	5.989433	23.41164	1.31E-06	1.16E-05
Novel:NC_037564.1_40744	1.396864	5.989433	23.41018	1.31E-06	1.16E-05
bta-miR-199a-5p	1.840066	4.520849	23.37555	1.33E-06	1.17E-05
Novel:NC_037545.1_1494	3.365135	2.500571	23.14604	1.50E-06	1.31E-05
bta-miR-99a-3p	2.097037	4.681966	22.96777	1.65E-06	1.42E-05
Novel:NC_037567.1_44260	1.247746	5.977627	22.815	1.78E-06	1.53E-05
Novel:hsa-miR-10399-3p	-4.37118	1.884984	22.46072	2.14E-06	1.82E-05
Novel:hsa-miR-548a-3p	1.435511	5.967269	22.00957	2.71E-06	2.28E-05
Novel:NC_037558.1_32412	1.381831	6.140114	21.51202	3.52E-06	2.94E-05
Novel:NC_037546.1_3432	4.770708	2.11667	21.45632	3.62E-06	3.00E-05
bta-miR-148c	1.294475	8.60482	21.25413	4.02E-06	3.31E-05
Novel:NC_037548.1_12074	1.66766	9.081819	21.02121	4.54E-06	3.70E-05
Novel:chi-miR-127-3p	4.21309	2.612103	20.80848	5.08E-06	4.10E-05
Novel:NC_037549.1_14266	5.077458	2.276403	20.73631	5.27E-06	4.23E-05
Novel:NC_037567.1_44693	1.229522	5.903541	20.71477	5.33E-06	4.24E-05
Novel:NC_037567.1_44155	-5.21103	1.9895	20.58696	5.70E-06	4.50E-05
bta-miR-2284z	4.187483	2.568666	20.16661	7.10E-06	5.56E-05
Novel:NC_037546.1_3986	-4.52285	1.722475	19.81734	8.52E-06	6.63E-05
Novel:NC_037564.1_40573	-4.8464	1.826845	19.68185	9.15E-06	7.06E-05
bta-miR-98	2.067118	5.413172	19.63612	9.37E-06	7.17E-05
bta-miR-2890	-1.39069	5.183454	19.62352	9.43E-06	7.17E-05
Novel:NC_037545.1_431	0.943957	8.012423	19.54523	9.82E-06	7.42E-05
Novel:NC_037547.1_8573	0.943955	8.012423	19.52575	9.93E-06	7.44E-05
bta-miR-126-3p	2.613075	3.782559	19.50999	1.00E-05	7.44E-05

Novel:NC_037545.1_1554	1.876525	3.552461	19.47835	1.02E-05	7.51E-05
Novel:NC_037558.1_32847	-1.66263	6.111542	19.33254	1.10E-05	8.05E-05
bta-miR-29a	1.164038	9.714093	19.28039	1.13E-05	8.21E-05
Novel:chi-miR-378-3p	2.748158	2.660223	19.26019	1.14E-05	8.24E-05
bta-miR-152	1.171206	8.354696	19.22639	1.16E-05	8.33E-05
bta-miR-143	1.413844	14.11466	19.0307	1.29E-05	9.17E-05
bta-miR-375	-1.41294	7.202781	18.64664	1.57E-05	0.000111
Novel:hsa-miR-185-3p	1.982001	3.559248	18.50851	1.69E-05	0.000119
bta-miR-2285e	1.533451	3.858602	18.49715	1.70E-05	0.000119
bta-miR-12030	2.039507	3.567166	18.34646	1.84E-05	0.000128
bta-miR-2284y	1.566224	4.879417	18.30726	1.88E-05	0.000129
bta-miR-199c	2.203299	2.93101	18.1593	2.03E-05	0.000139
bta-miR-205	-1.86181	5.159929	18.01358	2.19E-05	0.000149
Novel:NC_037558.1_31888	-3.59619	1.791055	17.79076	2.47E-05	0.000166
Novel:hsa-miR-2115-5p	-0.83981	13.02766	17.67818	2.62E-05	0.000175
bta-miR-92b	-1.08827	12.03627	17.58481	2.75E-05	0.000183
bta-let-7c	1.132887	9.077588	17.55495	2.79E-05	0.000184
Novel:NC_037564.1_40729	0.976859	8.723562	17.55125	2.80E-05	0.000184
bta-miR-9851	4.695285	2.091011	17.50988	2.86E-05	0.000186
Novel:NC_037564.1_41155	-1.31665	4.850642	17.50149	2.87E-05	0.000186
Novel:oar-miR-10b	1.808274	4.007948	17.41159	3.01E-05	0.000194
Novel:NC_037545.1_2589	4.813652	2.117394	17.13921	3.47E-05	0.000223
Novel:hsa-miR-4491	4.425708	1.954884	17.05911	3.62E-05	0.000231
bta-miR-130b	0.936808	7.882974	17.03202	3.68E-05	0.000232
bta-miR-2284x	0.921772	10.54743	17.02327	3.69E-05	0.000232
Novel:NC_037550.1_17666	1.351774	4.834404	16.84948	4.05E-05	0.000253
Novel:NC_037557.1_30688	-4.03135	1.750699	16.83054	4.09E-05	0.000254
bta-miR-411a	1.873177	5.339844	16.80948	4.13E-05	0.000255
Novel:chi-miR-24-5p	1.524926	6.450059	16.80041	4.15E-05	0.000255
Novel:NC_037562.1_38928	-4.3848	1.644332	16.70338	4.37E-05	0.000265
bta-miR-449a	3.488549	2.562118	16.70152	4.37E-05	0.000265
bta-miR-2285b	3.946897	2.458557	16.65274	4.49E-05	0.000271
bta-miR-2285cj	1.896978	3.479509	16.61699	4.57E-05	0.000274
bta-miR-11972	-2.0704	3.074774	16.46491	4.96E-05	0.000295
Novel:NC_037564.1_40393	-4.36066	1.616527	16.31986	5.35E-05	0.000317
bta-miR-379	4.609735	2.030385	16.28169	5.46E-05	0.000322
Novel:NC_037547.1_9353	1.793668	5.206497	16.26114	5.52E-05	0.000323
bta-miR-10164-3p	-1.61179	4.51517	16.22065	5.64E-05	0.000328
bta-miR-2285u	0.831947	7.134923	16.07436	6.09E-05	0.000353
Novel:NC_037563.1_39679	1.293281	6.315131	15.95025	6.50E-05	0.000374
Novel:NC_037546.1_4402	4.691846	2.058045	15.91618	6.62E-05	0.000379
Novel:NC_037569.1_47935	-1.24069	4.792874	15.75938	7.19E-05	0.000409
Novel:hsa-miR-629-3p	-3.84308	1.729879	15.61631	7.76E-05	0.000439
Novel:hsa-miR-4515	1.549402	6.798042	15.53533	8.10E-05	0.000456
bta-miR-199a-3p	1.149406	7.310694	15.49635	8.27E-05	0.000461
bta-miR-182	-1.37602	3.587991	15.49506	8.27E-05	0.000461
bta-miR-2285o	4.382282	1.934651	15.3994	8.70E-05	0.000482
Novel:NC_037552.1_22185	3.864585	2.429666	15.36893	8.84E-05	0.000487
Novel:hsa-miR-5195-5p	-1.05076	8.260993	15.34589	8.95E-05	0.00049

Novel:NC_037552.1_21299	3.855227	2.431463	15.22557	9.54E-05	0.00052
bta-miR-769	-0.91979	6.582947	15.19054	9.72E-05	0.000527
Novel:hsa-miR-1265	-1.26774	11.04787	15.17007	9.82E-05	0.00053
Novel:hsa-miR-3153	-3.06423	2.140379	15.08052	0.000103	0.000553
bta-miR-3601	-1.59224	3.451556	14.93556	0.000111	0.000594
bta-miR-3604	1.123306	7.961154	14.88985	0.000114	0.000605
bta-miR-2285aa	2.276716	2.784527	14.8603	0.000116	0.000611
Novel:NC_037564.1_40345	1.775477	5.426706	14.85079	0.000116	0.000611
bta-miR-542-5p	-1.613	3.374212	14.81783	0.000118	0.000619
Novel:NC_037562.1_38773	0.845002	12.54993	14.75118	0.000123	0.000638
bta-miR-192	-1.00007	11.15138	14.70701	0.000126	0.000648
Novel:chi-miR-151-5p	4.336399	1.918618	14.7011	0.000126	0.000648
bta-miR-218	2.228998	2.915438	14.62247	0.000131	0.000673
bta-miR-153	4.419153	1.940425	14.50526	0.00014	0.000712
Novel:NC_037560.1_34839	2.920671	2.277454	14.44758	0.000144	0.000725
bta-miR-2285bw	1.460382	3.981088	14.44532	0.000144	0.000725
Novel:NC_037553.1_23542	1.423306	4.658063	14.43631	0.000145	0.000725
Novel:hsa-miR-203a-5p	-3.87941	2.235836	14.42469	0.000146	0.000725
Novel:NC_037553.1_24652	1.398493	4.606136	14.41667	0.000147	0.000725
bta-miR-155	-1.024	8.19912	14.41586	0.000147	0.000725
Novel:hsa-miR-3974	4.970244	2.198793	14.3267	0.000154	0.000756
Novel:NC_037553.1_22994	1.247676	13.70481	14.31446	0.000155	0.000758
bta-miR-140	0.835678	12.45059	14.29892	0.000156	0.00076
Novel:hsa-miR-4276	3.559084	2.236152	14.22345	0.000162	0.000788
bta-miR-2284aa	4.287179	1.897747	14.17821	0.000166	0.000803
Novel:NC_037564.1_40877	3.553318	2.234126	13.86554	0.000196	0.000944
Novel:NC_037550.1_17420	2.236696	3.204808	13.79651	0.000204	0.000975
Novel:hsa-miR-6077	-3.36851	2.165059	13.66758	0.000218	0.001039
bta-miR-212	-3.9501	1.687778	13.65852	0.000219	0.001039
Novel:hsa-miR-548v	1.325714	6.578466	13.46544	0.000243	0.001146
Novel:NC_037549.1_16477	-1.63911	5.935281	13.40089	0.000252	0.001181
Novel:hsa-miR-4520-3p	1.470545	5.757401	13.34465	0.000259	0.001211
Novel:NC_037547.1_9101	-1.45515	4.456233	13.32271	0.000262	0.00122
bta-miR-1839	0.838475	8.846108	13.3087	0.000264	0.001224
Novel:NC_037553.1_22953	1.648468	3.089603	13.29262	0.000266	0.001229
bta-miR-1307	0.916116	6.149816	13.26598	0.00027	0.001241
Novel:hsa-miR-1301-3p	-4.40837	1.628096	13.24301	0.000274	0.00125
Novel:NC_037569.1_47783	-2.60261	1.919298	12.98301	0.000314	0.00143
Novel:NC_037564.1_40358	0.972582	6.210367	12.87406	0.000333	0.001509
Novel:NC_037557.1_31699	-1.50585	6.419306	12.83789	0.00034	0.001532
bta-miR-29d-3p	2.413987	2.29301	12.82718	0.000342	0.001534
Novel:NC_037565.1_42130	-3.59993	1.753756	12.80109	0.000346	0.001548
Novel:hsa-miR-548u	-4.19751	1.576437	12.76101	0.000354	0.001575
Novel:NC_037547.1_8583	1.272292	5.155344	12.63919	0.000378	0.001674
Novel:NC_037545.1_169	4.434116	1.971564	12.49359	0.000408	0.001801
bta-miR-204	-1.43972	5.464742	12.4641	0.000415	0.001822
Novel:hsa-miR-3150b-3p	-2.51266	2.386962	12.4352	0.000421	0.001843
Novel:NC_037546.1_4816	4.294327	1.87114	12.3856	0.000433	0.001884
Novel:oar-miR-134-3p	4.436598	1.953319	12.33888	0.000444	0.001924

Novel:hsa-miR-185-5p	-3.76603	1.616545	12.24898	0.000466	0.00201
bta-miR-30b-5p	-1.13426	4.674374	12.21779	0.000473	0.002035
bta-miR-142-5p	1.990192	3.542727	12.12471	0.000498	0.00213
bta-miR-505	-1.13535	4.80956	12.00126	0.000532	0.002267
bta-miR-2285av	2.145836	2.510096	11.93828	0.00055	0.002335
bta-miR-2285y	2.432402	2.479721	11.9223	0.000555	0.002341
Novel:NC_037554.1_24998	2.303989	3.295758	11.91819	0.000556	0.002341
Novel:NC_037548.1_13397	0.955784	4.597736	11.73713	0.000613	0.002569
bta-miR-1224	1.855954	3.588393	11.67517	0.000633	0.002645
Novel:hsa-miR-4743-5p	-1.45844	3.793662	11.667	0.000636	0.002646
Novel:NC_037546.1_4218	-1.40216	3.253065	11.62573	0.00065	0.002694
bta-miR-378d	1.922768	2.685714	11.56127	0.000673	0.002778
bta-miR-541	-1.50016	3.731261	11.53776	0.000682	0.0028
Novel:hsa-miR-4475	-2.10874	3.846452	11.53146	0.000684	0.0028
bta-miR-2899	2.871594	2.248506	11.46257	0.00071	0.002894
bta-let-7i	0.826241	10.80401	11.23064	0.000805	0.003266
bta-miR-2284m	4.200051	1.866581	11.03841	0.000892	0.003609
bta-miR-2285au	4.324372	1.925221	10.88594	0.000969	0.003899
Novel:NC_037545.1_548	-2.50021	2.05425	10.88049	0.000972	0.003899
bta-miR-2285co	0.704567	6.952334	10.82318	0.001002	0.004006
Novel:hsa-miR-4482-3p	-1.3719	3.781068	10.79043	0.00102	0.004061
bta-miR-26a	-0.80085	11.05742	10.64757	0.001102	0.00437
Novel:NC_037549.1_14328	3.928111	1.753525	10.60557	0.001127	0.004453
bta-miR-2285aj-5p	0.849879	8.99209	10.56153	0.001155	0.004543
Novel:hsa-miR-4632-3p	-3.47513	1.964895	10.51	0.001187	0.004653
Novel:hsa-miR-1205	2.204055	2.372954	10.49508	0.001197	0.004673
Novel:NC_037555.1_26499	3.975411	1.765706	10.43913	0.001234	0.004798
bta-miR-2284f	3.752268	1.686793	10.42069	0.001246	0.004828
bta-miR-2285cv	3.818267	1.712942	10.41242	0.001252	0.004831
Novel:hsa-miR-1915-3p	4.3596	1.873259	10.40478	0.001257	0.004833
Novel:chi-miR-125a-5p	-0.97546	5.336388	10.39644	0.001263	0.004837
bta-miR-26c	-0.79045	11.17691	10.37321	0.001279	0.00488
Novel:hsa-miR-3909	1.490595	3.535132	10.28038	0.001345	0.005112
Novel:hsa-miR-339-5p	1.187274	4.310873	10.27092	0.001351	0.005119
Novel:hsa-miR-4659a-3p	-3.72799	2.103619	10.12849	0.00146	0.00551
Novel:NC_037569.1_48167	-2.324	2.102307	10.07412	0.001504	0.005654
bta-miR-2285dd	2.703051	1.833204	9.991737	0.001572	0.005891
bta-miR-30a-5p	-0.6605	12.86624	9.980136	0.001582	0.005906
bta-miR-409b	-1.01866	7.845368	9.943205	0.001614	0.006004
bta-miR-2285bl	0.924345	4.477735	9.766608	0.001777	0.006585
bta-miR-136	2.548274	2.35379	9.704452	0.001838	0.006787
Novel:NC_037560.1_35631	-3.7359	1.676169	9.67743	0.001865	0.006862
bta-miR-411c-3p	-3.84228	1.827934	9.667067	0.001876	0.006876
bta-miR-196b	2.989022	1.943463	9.609618	0.001936	0.007069
Novel:NC_037561.1_36258	-1.63807	2.81917	9.599156	0.001947	0.007084
Novel:hsa-miR-5193	2.163481	2.361108	9.502593	0.002052	0.007441
bta-miR-1247-5p	1.504447	3.774701	9.292188	0.002301	0.008316
Novel:NC_037554.1_25895	-3.67314	1.471838	9.271438	0.002328	0.008381
bta-miR-21-5p	0.80953	11.5359	9.171984	0.002457	0.008811

Novel:NC_037569.1_46468	1.246732	5.706849	9.166873	0.002464	0.008811
Novel:hsa-miR-1252-3p	2.172958	2.516608	9.129755	0.002515	0.00896
Novel:NC_037565.1_41975	-3.80609	1.487423	9.101659	0.002554	0.009067
Novel:NC_037557.1_30447	2.184329	2.401154	9.089229	0.002571	0.009097
bta-miR-451	2.572886	3.324871	8.950301	0.002774	0.009782
Novel:hsa-miR-663a	-1.1889	9.508152	8.904346	0.002845	0.009996
Novel:hsa-miR-5587-3p	3.742883	1.691443	8.804192	0.003005	0.010524
Novel:NC_037557.1_31157	2.193206	2.408362	8.794104	0.003022	0.010546
Novel:hsa-miR-6868-3p	2.642617	2.416248	8.780211	0.003045	0.010591
bta-miR-502a	1.344872	3.381452	8.755526	0.003087	0.01067
bta-miR-199b	1.9482	2.892482	8.7542	0.003089	0.01067
bta-miR-129-3p	-2.71399	1.688397	8.695504	0.00319	0.010945
bta-miR-1260b	-3.08128	1.837605	8.695393	0.00319	0.010945
Novel:hsa-miR-3157-5p	2.090831	2.575054	8.681449	0.003215	0.010961
Novel:NC_037556.1_28345	3.709274	1.668478	8.675168	0.003226	0.010961
Novel:hsa-miR-4687-3p	-3.9966	1.480452	8.669179	0.003236	0.010961
Novel:NC_037556.1_28346	3.709301	1.668478	8.668487	0.003238	0.010961
Novel:NC_037557.1_31285	-3.88269	1.492629	8.617481	0.00333	0.011234
Novel:hsa-miR-744-5p	3.869015	1.707026	8.60635	0.00335	0.011266
Novel:NC_037568.1_44853	3.835957	1.698386	8.516641	0.003519	0.011796
Novel:NC_037549.1_15080	2.016975	5.480524	8.357678	0.003841	0.012831
bta-miR-2285ak-5p	3.404136	1.572332	8.332002	0.003895	0.012972
Novel:NC_037569.1_47143	-2.1502	2.087112	8.318812	0.003924	0.013023
bta-miR-34c	2.431897	2.588998	8.271422	0.004027	0.013324
bta-miR-497	0.755906	6.969268	8.229878	0.004121	0.013589
bta-miR-195	0.900534	4.780768	8.11051	0.004401	0.014466
bta-miR-2285j	3.593798	1.648322	8.090166	0.004451	0.014583
Novel:NC_037558.1_32790	-3.3526	1.394508	8.003352	0.004669	0.015249
Novel:NC_037555.1_28194	3.440562	1.591504	7.977037	0.004737	0.015423
bta-miR-10182-5p	-3.24021	1.362835	7.771743	0.005307	0.017185
bta-miR-222	1.107652	5.797248	7.769875	0.005312	0.017185
bta-miR-99a-5p	1.084764	8.970836	7.64821	0.005683	0.018325
Novel:NC_037564.1_40897	3.475651	1.604643	7.555105	0.005984	0.019227
bta-miR-2285da	2.21955	1.98348	7.545103	0.006017	0.019227
Novel:NC_037568.1_44803	1.249042	4.851546	7.544555	0.006019	0.019227
bta-miR-2285x	3.543044	1.610386	7.52488	0.006085	0.019378
Novel:NC_037547.1_7828	1.217311	3.42839	7.510634	0.006134	0.019424
Novel:NC_037551.1_19947	1.899092	2.340871	7.509383	0.006138	0.019424
Novel:NC_037563.1_39275	1.899217	2.340871	7.495935	0.006184	0.019509
bta-miR-484	-0.67307	7.623101	7.467695	0.006282	0.019756
Novel:chi-miR-214-3p	-3.3119	1.852499	7.443775	0.006366	0.019902
Novel:NC_037545.1_503	1.123753	8.720566	7.44335	0.006367	0.019902
bta-miR-6528	-3.1083	1.817711	7.432022	0.006407	0.019912
bta-miR-193a-5p	1.174877	4.048057	7.431417	0.006409	0.019912
bta-miR-2285bo	3.762986	1.71629	7.399752	0.006523	0.020204
Novel:hsa-miR-6715a-3p	0.743857	7.040934	7.392465	0.00655	0.020225
bta-miR-342	-0.74673	7.138332	7.367262	0.006642	0.020448
bta-miR-2285bf	4.217823	1.887701	7.359793	0.00667	0.020472
Novel:NC_037564.1_40415	2.686179	2.121144	7.345922	0.006722	0.020568

Novel:NC_037548.1_11583	3.922619	1.774449	7.323247	0.006807	0.020767
Novel:NC_037553.1_24045	3.047446	1.463197	7.282457	0.006963	0.02115
bta-miR-2892	1.041379	5.254649	7.279638	0.006974	0.02115
bta-miR-11988	3.531721	1.618597	7.265271	0.00703	0.021257
Novel:NC_037547.1_7586	3.698814	1.648387	7.230103	0.007169	0.021603
Novel:chi-miR-221-5p	-2.25779	2.067137	7.225699	0.007187	0.021603
Novel:NC_037546.1_4132	3.340226	1.559432	7.205636	0.007267	0.021781
bta-let-7a-5p	0.962839	10.1121	7.187279	0.007342	0.021941
Novel:NC_037546.1_6204	3.392045	1.577287	7.176779	0.007385	0.022005
bta-miR-6518	-0.80924	6.301559	7.157698	0.007464	0.022175
Novel:hsa-miR-6834-5p	1.466094	3.000097	7.147342	0.007508	0.022229
bta-miR-421	-0.83356	5.585144	7.142949	0.007526	0.022229
Novel:NC_037558.1_31842	-1.75149	2.398247	7.117754	0.007632	0.022478
Novel:NC_037561.1_36221	1.787863	2.139022	7.092812	0.007739	0.022727
Novel:NC_037547.1_7786	-2.96908	1.811948	7.086276	0.007768	0.022745
bta-miR-2408	2.24821	1.965422	6.994217	0.008177	0.023876
bta-miR-2483-5p	-1.6427	2.686172	6.969838	0.008289	0.024134
Novel:hsa-miR-219a-2-3p	2.053424	3.415011	6.936044	0.008448	0.024524
Novel:hsa-miR-4501	4.330356	1.861445	6.930411	0.008474	0.024532
bta-miR-3431	1.091843	3.346864	6.890536	0.008665	0.025014
Novel:hsa-miR-3173-3p	-2.80676	1.994541	6.875716	0.008737	0.02515
Novel:NC_037549.1_16021	-1.52568	3.433498	6.870764	0.008762	0.02515
Novel:NC_037558.1_33220	-3.18151	1.495175	6.823651	0.008996	0.025701
Novel:NC_037558.1_32366	-3.1815	1.495175	6.822006	0.009004	0.025701
Novel:NC_037564.1_41137	2.556682	2.070241	6.810611	0.009062	0.025793
Novel:NC_037548.1_11028	-3.11243	1.74809	6.750772	0.009371	0.026598
Novel:NC_037550.1_18832	-1.94778	2.160908	6.718938	0.009539	0.027002
bta-miR-6123	-0.90128	4.651462	6.705099	0.009614	0.027108
bta-miR-181d	-1.04812	4.544557	6.702075	0.00963	0.027108
Novel:NC_037550.1_18908	-0.69097	9.506108	6.682886	0.009734	0.027326
Novel:hsa-miR-548c-3p	2.079884	1.883438	6.675693	0.009774	0.027361
bta-miR-6520	1.23031	4.529476	6.631475	0.010019	0.027971
Novel:hsa-miR-3917	3.73243	1.646482	6.622516	0.01007	0.028036
Novel:chi-miR-500-5p	1.110315	3.078505	6.602295	0.010185	0.028279
Novel:hsa-miR-6776-5p	3.567498	1.618168	6.596175	0.01022	0.028299
bta-miR-197	-1.02556	5.121527	6.558008	0.010441	0.028834
bta-miR-669	-0.82028	11.02601	6.542163	0.010535	0.029013
bta-miR-125b	1.235241	7.167017	6.512649	0.010711	0.029419
bta-miR-141	-1.06663	4.148658	6.480374	0.010907	0.029878
Novel:NC_037557.1_30571	-3.69037	1.555132	6.46633	0.010994	0.030034
bta-miR-186	-0.6836	9.529095	6.453474	0.011074	0.030171
Novel:NC_037550.1_19123	2.99471	1.457216	6.416073	0.011309	0.030731
Novel:NC_037560.1_35256	3.555157	1.622831	6.393475	0.011454	0.031042
Novel:NC_037545.1_1689	2.207348	2.112103	6.375482	0.011571	0.031275
Novel:NC_037566.1_43276	3.753913	1.623389	6.356663	0.011694	0.031525
Novel:NC_037558.1_33041	3.278014	1.545285	6.302647	0.012056	0.032414
Novel:hsa-miR-6770-5p	-3.00721	1.798173	6.272049	0.012266	0.032892
bta-miR-665	-1.02249	3.887364	6.229241	0.012566	0.033608
Novel:NC_037547.1_10839	-2.95682	1.566468	6.192192	0.012832	0.034229

Novel:hsa-miR-6784-3p	-1.96923	2.145736	6.18663	0.012872	0.034247
Novel:NC_037547.1_8898	3.221996	1.512076	6.114597	0.013407	0.035577
bta-miR-455-3p	2.752086	1.833511	6.040272	0.013983	0.03701
Novel:hsa-miR-4695-5p	3.265385	1.518632	6.003998	0.014273	0.037572
Novel:NC_037547.1_10635	-0.53403	13.59165	6.003581	0.014277	0.037572
Novel:hsa-miR-4434	-2.81594	1.528389	5.999949	0.014306	0.037572
Novel:NC_037550.1_19237	-2.86027	1.521505	5.985377	0.014425	0.037787
bta-miR-2435	0.769012	5.508119	5.974891	0.014511	0.037915
bta-miR-193a	1.24099	3.915333	5.935166	0.014842	0.038679
bta-miR-12006	2.269789	1.934736	5.905398	0.015095	0.039238
Novel:hsa-miR-208a-5p	-1.65249	3.12562	5.894826	0.015185	0.039374
Novel:hsa-miR-6771-5p	-1.08075	5.295686	5.874072	0.015365	0.03974
bta-miR-424-5p	0.867123	5.993412	5.857087	0.015514	0.040023
Novel:hsa-miR-1184	3.605466	1.574764	5.849186	0.015584	0.040102
Novel:NC_037548.1_12259	0.803459	5.234981	5.834304	0.015717	0.040341
Novel:hsa-miR-4767	3.408232	1.603294	5.808867	0.015946	0.040692
Novel:NC_037545.1_315	2.169975	2.090469	5.806378	0.015968	0.040692
Novel:hsa-miR-486-3p	3.166692	1.491681	5.805821	0.015973	0.040692
Novel:hsa-miR-149-3p	3.354937	1.552945	5.731118	0.016667	0.042353
Novel:chi-miR-24-3p	-1.70731	2.958852	5.713548	0.016834	0.042672
bta-miR-2284j	1.633598	2.111724	5.708168	0.016886	0.042697
bta-miR-106b	-0.70725	5.117987	5.684981	0.017111	0.043158
bta-miR-2389	-1.42872	2.480131	5.641283	0.017542	0.044137
bta-miR-1296	0.785491	5.245358	5.602113	0.017939	0.045024
Novel:NC_037564.1_40236	-1.18072	3.426479	5.586577	0.018099	0.045313
Novel:hsa-miR-1908-3p	-1.57629	3.468873	5.572402	0.018246	0.04557
Novel:NC_037547.1_9455	-1.58328	2.672723	5.567886	0.018293	0.045576
Novel:hsa-miR-203a-3p	-1.17698	3.345902	5.492066	0.019103	0.047392
bta-miR-592	1.894453	2.222739	5.49097	0.019115	0.047392
Novel:NC_037565.1_42675	1.983604	2.570205	5.44641	0.019609	0.048491
Novel:NC_037545.1_583	3.048153	1.488061	5.442434	0.019653	0.048491
Novel:hsa-miR-3683	3.789265	1.640504	5.380787	0.02036	0.050106
bta-miR-18a	-1.51336	2.065128	5.37681	0.020406	0.050106
bta-miR-11971	-0.9088	4.568423	5.370377	0.020482	0.05017
Novel:NC_037548.1_12213	2.121308	1.640838	5.355697	0.020655	0.050411
Novel:NC_037554.1_25983	1.902777	1.796546	5.350846	0.020712	0.050411
Novel:NC_037569.1_47087	0.821405	6.113148	5.349493	0.020728	0.050411
Novel:NC_037556.1_29821	3.379954	1.528561	5.32045	0.021077	0.051136
bta-miR-17-3p	2.58616	1.762606	5.301671	0.021305	0.051567
bta-miR-493	-1.38988	2.64854	5.284014	0.021522	0.051969
Novel:hsa-miR-6818-3p	-2.34007	1.741309	5.270828	0.021686	0.05224
Novel:hsa-miR-1199-3p	0.973018	5.062874	5.169366	0.022989	0.055249
bta-miR-151-5p	0.591127	6.912468	5.125794	0.023573	0.056519
Novel:NC_037557.1_30978	1.475999	2.031097	5.113512	0.02374	0.056787
bta-miR-26b	0.528955	7.281389	5.079857	0.024205	0.057676
bta-miR-2299-3p	3.175657	1.499866	5.078462	0.024225	0.057676
bta-miR-12034	0.74593	5.693699	5.066847	0.024388	0.057928
Novel:NC_037564.1_40367	-0.92151	3.802399	5.005722	0.025264	0.059869
Novel:hsa-miR-6885-3p	3.34122	1.541519	4.996082	0.025405	0.060064

Novel:chi-miR-320-3p	2.313879	1.699907	4.955482	0.026008	0.061348
bta-miR-2285dk	-2.58349	1.465838	4.930517	0.026386	0.062096
Novel:hsa-miR-5002-5p	1.452827	2.309882	4.910521	0.026694	0.062675
bta-miR-11987	1.009996	5.270329	4.898006	0.026888	0.062985
bta-miR-2331-3p	2.156347	1.664081	4.887477	0.027052	0.063225
Novel:NC_037564.1_41300	2.181329	1.935592	4.855295	0.027561	0.064268
Novel:chi-miR-34a	3.146686	1.507572	4.832029	0.027936	0.064992
bta-miR-486	1.047185	11.54257	4.816154	0.028194	0.065444
Novel:hsa-miR-12117	3.134647	1.519411	4.74497	0.029384	0.068051
bta-miR-2285cs	1.2691	2.093282	4.73725	0.029516	0.068202
bta-miR-28	-0.85345	3.947604	4.710153	0.029985	0.069128
Novel:NC_037562.1_38088	3.078983	1.507202	4.689562	0.030346	0.069804
Novel:hsa-miR-5088-5p	3.329251	1.535475	4.678535	0.030542	0.070095
Novel:NC_037547.1_10260	-1.97811	3.634757	4.660705	0.030861	0.070667
Novel:NC_037545.1_1965	2.998568	1.48337	4.656049	0.030944	0.0707
Novel:NC_037556.1_29371	-1.63782	1.908975	4.644375	0.031155	0.071023
bta-miR-1343-3p	-0.72895	5.033847	4.634289	0.031339	0.071283
Novel:oar-miR-299-3p	3.469047	1.515066	4.603114	0.031914	0.072428
Novel:hsa-miR-10392-3p	-1.47089	2.426601	4.574228	0.032456	0.073496
bta-miR-133a	1.236466	3.232586	4.55301	0.032861	0.074231
bta-let-7g	-0.66203	8.527694	4.549581	0.032927	0.074231
Novel:NC_037556.1_29299	-1.62604	2.270234	4.541447	0.033084	0.07442
Novel:NC_037558.1_32842	0.971619	4.191776	4.498439	0.033926	0.076146
bta-miR-2284c	2.467177	1.794845	4.494531	0.034003	0.076153
Novel:hsa-miR-759	1.16179	2.691776	4.471833	0.034458	0.077002
Novel:hsa-miR-4800-5p	-0.54852	7.319868	4.459495	0.034708	0.077256
Novel:chi-miR-103-5p	2.156382	1.674635	4.458717	0.034724	0.077256
Novel:NC_037564.1_40695	2.624099	1.378179	4.441201	0.035082	0.077883
bta-miR-99b	0.721601	12.01029	4.436296	0.035183	0.077937
Novel:hsa-miR-3122	2.618451	1.376561	4.401143	0.035915	0.079387
Novel:hsa-miR-6888-5p	-2.25292	1.678685	4.384547	0.036266	0.07999
bta-miR-21-3p	-0.59865	10.42962	4.380061	0.036362	0.080027
bta-miR-3956	-2.22255	1.300898	4.364923	0.036686	0.080567
Novel:NC_037552.1_22617	-1.23461	3.113449	4.353615	0.03693	0.080929
bta-miR-671	-0.87835	3.3512	4.34013	0.037224	0.081398
Novel:NC_037556.1_30323	-1.61107	2.304906	4.328399	0.037481	0.081615
bta-let-7f	0.672139	10.52281	4.327536	0.0375	0.081615
Novel:NC_037559.1_34015	2.278719	1.932466	4.324674	0.037564	0.081615
Novel:hsa-miR-6832-3p	2.280343	1.659467	4.319951	0.037668	0.081667
Novel:NC_037546.1_3761	2.044474	2.346788	4.308653	0.037919	0.081946
Novel:NC_037549.1_15024	1.347344	2.341508	4.306929	0.037957	0.081946
Novel:hsa-miR-6727-5p	1.011293	10.86949	4.274488	0.038689	0.083348
Novel:hsa-miR-4514	-1.04048	3.7561	4.263119	0.038948	0.083661
Novel:NC_037546.1_5554	1.271647	2.43772	4.260949	0.038998	0.083661
Novel:NC_037553.1_23290	-1.05981	2.622155	4.244334	0.039382	0.084218
bta-miR-339b	0.518104	7.372615	4.242539	0.039423	0.084218
bta-miR-208a	2.609009	1.376609	4.210588	0.040172	0.08564
Novel:hsa-miR-146b-3p	1.424572	2.734996	4.151931	0.041587	0.08847
Novel:NC_037564.1_41423	1.125729	2.502913	4.132648	0.042063	0.089297

Novel:hsa-miR-6734-3p	1.008066	7.95409	4.124842	0.042258	0.089524
bta-miR-3432a	2.885659	1.430096	4.106772	0.042712	0.090106
bta-miR-181a	-0.36384	12.30758	4.106439	0.04272	0.090106
bta-miR-2881	-2.26869	1.317191	4.103351	0.042798	0.090106
bta-miR-330	-1.539	2.466464	4.055462	0.044029	0.092506
Novel:NC_037565.1_42672	-0.61998	8.590212	4.043289	0.044347	0.092983
Novel:hsa-miR-3169	2.990726	1.418222	3.951875	0.046819	0.097965
bta-miR-431	-2.03876	1.442885	3.942533	0.04708	0.098308
Novel:NC_037549.1_15917	-0.81352	4.592593	3.912851	0.047918	0.099854
Novel:hsa-miR-8057	0.508734	8.375805	3.900124	0.048283	0.100408
Novel:hsa-miR-136-3p	1.5379	2.213474	3.876965	0.048953	0.101595
Novel:NC_037558.1_32697	2.026551	1.597211	3.839798	0.05005	0.103646
Novel:NC_037548.1_11079	-0.71773	8.691761	3.834319	0.050213	0.103646
Novel:NC_037548.1_11059	2.960856	1.441166	3.833218	0.050246	0.103646
Novel:NC_037548.1_13083	-0.5717	5.433777	3.809968	0.050948	0.104882
Novel:chi-miR-345-5p	2.277302	1.665726	3.801982	0.051192	0.105171
bta-miR-873	-1.12351	4.993447	3.769096	0.052208	0.106819
Novel:hsa-miR-5580-5p	0.783945	5.047405	3.767048	0.052272	0.106819
Novel:NC_037553.1_24444	-2.55848	1.647557	3.76264	0.05241	0.106819
Novel:NC_037552.1_21699	2.669304	1.392324	3.762516	0.052414	0.106819
Novel:hsa-miR-4795-5p	-2.4345	1.481397	3.754248	0.052673	0.107134
bta-miR-4449	-2.28535	1.548301	3.713883	0.053962	0.109536
Novel:chi-miR-202-3p	-0.45246	11.14759	3.70114	0.054375	0.110156
Novel:hsa-miR-3919	1.243962	2.607347	3.696174	0.054537	0.110265
Novel:NC_037568.1_45446	-1.94711	1.723557	3.57537	0.058642	0.11832
Novel:NC_037564.1_40932	-2.16697	1.661061	3.571417	0.058782	0.11832
bta-miR-369-3p	0.993253	2.946535	3.568942	0.05887	0.11832
Novel:NC_037558.1_32328	3.219752	1.449788	3.541991	0.059834	0.120021
Novel:NC_037553.1_24374	0.705913	4.498713	3.523168	0.060517	0.121152
Novel:NC_037555.1_26458	2.44356	1.346259	3.508437	0.061057	0.121994
Novel:NC_037562.1_38320	2.470133	1.357791	3.49771	0.061454	0.122547
bta-miR-2889	0.773611	6.50004	3.493389	0.061614	0.122627
Novel:NC_037561.1_36492	0.894567	2.885259	3.489349	0.061765	0.122687
Novel:NC_037555.1_27478	2.450505	1.347565	3.479623	0.062129	0.12317
Novel:NC_037545.1_1192	0.686764	3.250631	3.472552	0.062395	0.123457
Novel:NC_037549.1_15660	-0.35595	11.30692	3.466742	0.062615	0.123652
Novel:NC_037556.1_30106	-0.35595	11.30692	3.458474	0.062928	0.124031
Novel:chi-miR-493-3p	2.475363	1.678373	3.431193	0.063976	0.125852
Novel:NC_037569.1_46554	0.974143	3.354398	3.41222	0.064715	0.127061
bta-miR-2285ae	1.795391	1.552078	3.404681	0.065012	0.127196
Novel:NC_037563.1_39389	-2.66835	1.592004	3.404128	0.065033	0.127196
Novel:NC_037546.1_3187	2.630622	1.399594	3.399263	0.065226	0.127327
Novel:NC_037568.1_45118	1.796131	2.257136	3.392173	0.065507	0.127631
Novel:NC_037555.1_27170	1.887688	1.806839	3.350181	0.067198	0.130676
bta-miR-15b	0.842349	3.113987	3.346662	0.067341	0.130707
Novel:NC_037561.1_37534	0.674699	5.227825	3.331559	0.067962	0.131661
bta-let-7b	-0.42124	10.0414	3.321806	0.068367	0.132193
Novel:hsa-miR-6748-3p	1.437942	2.275415	3.304986	0.06907	0.1333
Novel:NC_037549.1_16548	1.165663	2.260933	3.269505	0.070579	0.135955

Novel:NC_037564.1_40643	1.388344	2.022146	3.245601	0.071615	0.137691
Novel:NC_037547.1_8560	0.438278	6.489157	3.241444	0.071797	0.137781
Novel:hsa-miR-4443	2.417509	1.341212	3.235939	0.072039	0.137984
Novel:NC_037558.1_33194	3.017153	1.411069	3.219959	0.072745	0.139076
bta-let-7e	0.776252	5.938148	3.195675	0.073833	0.140892
Novel:NC_037547.1_8021	2.562173	1.38966	3.163133	0.075319	0.143457
bta-miR-345-5p	-2.37403	1.502983	3.156021	0.075647	0.143815
bta-miR-2285af	2.646881	1.382928	3.135694	0.076596	0.145132
Novel:hsa-miR-4746-5p	0.80325	3.935307	3.13483	0.076636	0.145132
bta-miR-483	-1.59307	1.667441	3.132045	0.076767	0.145132
Novel:NC_037564.1_40307	0.778214	4.101539	3.103685	0.078115	0.147406
Novel:NC_037567.1_43945	-2.07657	1.688593	3.08942	0.078803	0.148429
Novel:chi-miR-9-5p	2.037155	1.811226	3.069213	0.079788	0.150008
bta-miR-1343-5p	1.419998	2.162148	3.049762	0.080749	0.151534
Novel:NC_037547.1_8236	2.386848	1.858929	3.043316	0.08107	0.151857
Novel:NC_037545.1_1896	2.022102	1.602682	3.036375	0.081418	0.152228
bta-miR-503-5p	-1.7841	1.479371	2.995109	0.083516	0.155866
Novel:NC_037547.1_8809	1.613596	1.742979	2.987113	0.08393	0.156352
Novel:NC_037546.1_2906	1.620222	2.560934	2.980668	0.084264	0.156689
Novel:NC_037564.1_40946	2.229383	1.616771	2.969952	0.084824	0.157443
Novel:hsa-miR-3179	-2.89311	1.513818	2.965174	0.085075	0.157621
Novel:NC_037561.1_37162	0.82337	2.849062	2.935659	0.086643	0.160235
Novel:hsa-miR-3126-5p	2.55543	1.374119	2.921875	0.087386	0.161316
bta-miR-27b	-0.33896	13.93007	2.88595	0.089355	0.164653
Novel:NC_037555.1_27093	1.755382	1.736116	2.830789	0.092473	0.17009
Novel:NC_037552.1_21735	2.504977	1.343465	2.826677	0.09271	0.170219
Novel:NC_037547.1_10537	0.422254	6.487384	2.812508	0.093532	0.171419
bta-miR-2468	1.083911	2.618375	2.779166	0.095498	0.174708
Novel:NC_037567.1_44073	0.43404	9.585821	2.759745	0.096664	0.176331
Novel:hsa-miR-6131	-0.43929	6.618665	2.758624	0.096731	0.176331
Novel:NC_037554.1_24951	0.426565	6.411119	2.752026	0.097131	0.176744
Novel:hsa-miR-149-5p	0.959409	2.202583	2.74418	0.097609	0.177297
bta-miR-188	0.682079	4.138516	2.731488	0.098388	0.178198
Novel:NC_037558.1_32528	1.972395	1.807592	2.730393	0.098455	0.178198
Novel:NC_037547.1_7667	1.772751	1.740783	2.71977	0.099112	0.179059
bta-miR-504	0.908542	2.685769	2.715158	0.099399	0.179059
bta-miR-11980	1.43649	2.105497	2.714218	0.099458	0.179059
bta-miR-2411-5p	2.379422	1.315643	2.690645	0.100939	0.181405
bta-miR-126-5p	0.635148	7.547563	2.668439	0.102356	0.183416
bta-miR-1247-3p	0.81813	3.425875	2.667481	0.102418	0.183416
Novel:NC_037546.1_5605	-1.87295	1.524337	2.648853	0.103625	0.185252
Novel:chi-let-7i-3p	-1.88752	2.016043	2.612524	0.106023	0.189207
Novel:NC_037569.1_46557	0.656327	4.221164	2.604942	0.106531	0.189494
bta-miR-2285cm	1.282948	2.122112	2.604575	0.106556	0.189494
bta-miR-362-3p	0.891832	3.325013	2.599314	0.10691	0.189793
bta-miR-2285f	0.583491	3.47853	2.590378	0.107515	0.190535
Novel:hsa-miR-6125	-0.75411	4.499761	2.578341	0.108335	0.191655
bta-miR-2478	-1.12293	2.582596	2.539237	0.111048	0.196115
Novel:NC_037567.1_44728	0.885738	3.10722	2.515352	0.112742	0.198762

Novel:hsa-miR-4683	-2.70606	1.438878	2.508527	0.113232	0.199127
Novel:NC_037547.1_10102	1.141494	2.552621	2.507015	0.11334	0.199127
bta-miR-11977	0.525052	4.873319	2.48273	0.115102	0.201875
bta-miR-2338	2.122905	1.285826	2.4755	0.115633	0.202456
bta-miR-219	-1.41205	2.002062	2.45786	0.116938	0.204391
Novel:NC_037556.1_30288	0.61342	7.600591	2.45418	0.117212	0.204519
Novel:NC_037550.1_18976	1.568017	1.48472	2.440882	0.11821	0.205769
bta-miR-424-3p	-0.48938	5.12753	2.439257	0.118332	0.205769
bta-miR-2904	-0.73188	5.046837	2.433342	0.118779	0.206195
bta-miR-455-5p	-0.6228	3.969401	2.399442	0.121379	0.210348
Novel:NC_037547.1_10214	-0.39633	8.163206	2.394497	0.121763	0.210656
Novel:NC_037555.1_26610	1.520553	1.92611	2.385289	0.122482	0.211541
Novel:hsa-miR-8077	-0.46957	5.021965	2.366772	0.123943	0.213508
Novel:hsa-miR-3064-5p	1.658225	1.540791	2.365554	0.12404	0.213508
Novel:NC_037553.1_23923	1.338093	1.829878	2.355152	0.12487	0.214574
bta-miR-2446	-1.04729	2.258141	2.313039	0.128293	0.220085
bta-miR-16a	-0.62472	4.919493	2.300009	0.129373	0.221565
Novel:NC_037560.1_35881	1.079713	1.883612	2.293266	0.129936	0.222156
Novel:NC_037549.1_14559	-1.9019	1.542833	2.251188	0.133512	0.227887
bta-miR-12023	-1.1736	1.791724	2.23202	0.135177	0.230343
bta-miR-423-5p	-0.33513	14.18469	2.22818	0.135513	0.230531
Novel:NC_037567.1_44300	0.832307	3.137013	2.219811	0.13625	0.231382
bta-miR-193a-3p	0.636931	4.904254	2.217342	0.136468	0.231382
Novel:hsa-miR-4524a-3p	1.923383	1.730898	2.210839	0.137044	0.231847
Novel:NC_037550.1_17628	0.378769	7.792666	2.209121	0.137197	0.231847
bta-miR-760-3p	0.374216	7.631615	2.169872	0.140738	0.237437
Novel:NC_037555.1_27452	1.146713	2.232166	2.161445	0.141512	0.238348
Novel:chi-miR-491-5p	0.619729	3.079856	2.134988	0.143972	0.242091
Novel:NC_037549.1_14209	1.560075	1.668238	2.110847	0.146258	0.245047
Novel:NC_037552.1_21136	1.467427	1.811001	2.110148	0.146325	0.245047
Novel:NC_037552.1_22028	1.467668	1.811001	2.108832	0.146451	0.245047
Novel:hsa-miR-4450	-1.56007	1.657375	2.092831	0.147992	0.246884
Novel:hsa-miR-765	-1.56152	1.604977	2.091585	0.148112	0.246884
Novel:NC_037569.1_46559	0.334682	9.274366	2.0899	0.148276	0.246884
Novel:hsa-miR-7704	1.857332	1.56874	2.066197	0.150597	0.250339
Novel:NC_037552.1_22436	1.33645	2.468878	2.036991	0.153513	0.254772
bta-miR-6535	1.622807	1.507389	2.029744	0.154247	0.255573
Novel:NC_037553.1_23200	-0.60295	3.477013	2.018545	0.155388	0.257046
bta-miR-425-3p	0.442889	4.510771	2.003751	0.156911	0.259144
bta-miR-380-3p	0.525235	4.742056	1.995844	0.157731	0.260078
bta-miR-532	0.324778	9.202959	1.987872	0.158564	0.261028
bta-miR-454	-1.84741	1.903918	1.937007	0.163994	0.269532
Novel:hsa-miR-4442	-1.55112	1.514101	1.921058	0.16574	0.271963
Novel:hsa-miR-760	-1.74074	1.856865	1.915828	0.166317	0.272472
bta-miR-31	-0.3481	8.006829	1.907674	0.167222	0.273514
Novel:NC_037552.1_21563	1.290449	2.446341	1.885205	0.169744	0.277194
Novel:NC_037566.1_43265	0.727968	2.598162	1.879019	0.170446	0.277895
Novel:NC_037548.1_12436	-1.85892	1.564663	1.864231	0.172137	0.280204
bta-miR-409a	-1.94417	1.814905	1.861675	0.172432	0.280236

Novel:NC_037546.1_4277	1.401031	1.967407	1.858214	0.172831	0.280438
Novel:chi-miR-411b-5p	0.617676	3.230875	1.847067	0.174125	0.282088
bta-miR-331-3p	0.880984	2.288743	1.83351	0.175713	0.28421
Novel:hsa-miR-1199-5p	1.571961	1.525096	1.825302	0.176683	0.285326
Novel:NC_037560.1_35403	1.202358	5.696217	1.813126	0.178134	0.287212
bta-miR-1306	-0.86545	2.516364	1.808653	0.17867	0.287622
bta-miR-194b	-1.67787	1.733681	1.797441	0.180022	0.289342
Novel:NC_037565.1_41728	1.720232	1.476839	1.77515	0.182746	0.293257
bta-miR-328	-0.65505	4.639757	1.772624	0.183058	0.293295
bta-miR-185	1.187318	2.217683	1.741428	0.186958	0.299074
bta-miR-432	-0.48662	5.254351	1.730566	0.188339	0.30081
bta-miR-30f	-0.62215	3.198713	1.727332	0.188752	0.300999
bta-miR-146a	1.361799	2.155838	1.718436	0.189895	0.302348
Novel:NC_037546.1_4225	0.903814	2.917408	1.679185	0.195033	0.310044
Novel:hsa-miR-4778-3p	1.592933	1.498543	1.664816	0.196954	0.312162
Novel:NC_037546.1_6567	-1.87292	1.711288	1.664644	0.196977	0.312162
bta-miR-2312	1.248801	1.652199	1.659409	0.197683	0.312794
bta-miR-410	0.364607	7.526573	1.653603	0.198469	0.31343
Novel:NC_037549.1_15945	-1.60956	1.687103	1.6519	0.198701	0.31343
Novel:hsa-miR-6741-5p	-0.39074	6.72832	1.640758	0.200221	0.315341
bta-miR-125a	0.637915	7.52891	1.636764	0.20077	0.315717
Novel:hsa-miR-635	1.43254	1.710091	1.634512	0.20108	0.315717
Novel:NC_037549.1_14540	1.642733	2.107917	1.613861	0.20395	0.319687
Novel:NC_037550.1_18131	-1.50197	1.513583	1.611823	0.204236	0.319687
Novel:NC_037552.1_21521	-2.05524	1.717519	1.603748	0.205373	0.320556
Novel:hsa-miR-1252-5p	1.194005	1.766411	1.603413	0.20542	0.320556
bta-miR-574	0.3256	10.50774	1.594088	0.206743	0.322127
Novel:hsa-miR-4277	-0.82903	1.896485	1.589244	0.207434	0.322711
Novel:NC_037548.1_13020	-1.56066	1.435442	1.577213	0.209163	0.324904
Novel:NC_037567.1_44673	1.88266	1.541969	1.572802	0.209801	0.325398
bta-miR-654	-1.2552	1.738886	1.559647	0.211717	0.327872
Novel:NC_037546.1_3818	1.338166	1.653387	1.528696	0.216308	0.334474
bta-miR-339a	0.315192	7.62451	1.503262	0.22017	0.339683
Novel:NC_037553.1_23622	1.246274	1.683655	1.502131	0.220344	0.339683
bta-miR-29b	0.951943	2.70531	1.495532	0.22136	0.340719
Novel:NC_037564.1_41393	-1.03747	2.193261	1.493434	0.221684	0.340719
Novel:NC_037564.1_40326	0.424902	7.080237	1.485175	0.222967	0.342173
Novel:hsa-miR-10522-5p	-1.96511	1.686755	1.478359	0.224031	0.34329
Novel:hsa-miR-619-3p	1.759235	1.557949	1.469706	0.225392	0.344857
bta-miR-500	-1.43824	1.427899	1.465141	0.226114	0.345385
Novel:chi-miR-7-5p	1.05996	2.026739	1.463238	0.226416	0.345385
bta-miR-27a-5p	1.536759	1.752797	1.450749	0.228408	0.347904
Novel:NC_037549.1_15915	1.591513	1.667372	1.426944	0.232264	0.353249
bta-miR-128	0.298852	8.761105	1.418275	0.233688	0.354885
Novel:NC_037548.1_12165	-0.62017	2.851068	1.413971	0.234398	0.355434
bta-miR-381	0.407995	6.993143	1.360121	0.243516	0.368712
bta-miR-24-3p	0.299017	9.972739	1.350932	0.245115	0.370582
Novel:NC_037560.1_35724	-0.75979	2.148924	1.34129	0.246807	0.372587
Novel:NC_037553.1_23265	-0.66263	3.492746	1.321516	0.25032	0.376653

bta-miR-323	0.853711	2.321409	1.320096	0.250575	0.376653
Novel:hsa-miR-6867-5p	0.392783	4.879521	1.319903	0.250609	0.376653
bta-miR-30c	-0.36846	5.435311	1.292272	0.25563	0.383633
Novel:NC_037545.1_698	-1.27739	1.666948	1.287749	0.256463	0.384318
bta-miR-425-5p	0.344166	5.769016	1.285294	0.256917	0.384432
Novel:NC_037569.1_46992	-0.34748	5.771867	1.275492	0.258739	0.386591
Novel:NC_037553.1_24733	1.411031	1.685415	1.265413	0.260629	0.388845
bta-miR-2284ab	0.340068	4.92297	1.248789	0.263784	0.392976
bta-miR-2285k	0.832753	1.78724	1.228372	0.267724	0.398009
Novel:NC_037546.1_4112	0.437211	3.010354	1.22537	0.268309	0.398009
Novel:NC_037547.1_7372	0.437219	3.010354	1.225244	0.268334	0.398009
Novel:hsa-miR-1304-3p	1.620618	1.522721	1.221647	0.269038	0.398473
bta-miR-22-5p	0.406321	5.234732	1.218301	0.269694	0.398866
bta-miR-138	1.310968	1.649468	1.209585	0.271414	0.400828
Novel:hsa-miR-652-5p	0.38426	5.487295	1.188504	0.275631	0.406467
Novel:NC_037565.1_42254	0.568108	3.402071	1.185121	0.276316	0.406729
bta-miR-1271	-0.635	3.487239	1.182647	0.276818	0.406729
Novel:hsa-miR-3620-5p	0.993986	1.722003	1.179963	0.277363	0.406729
Novel:NC_037552.1_21762	1.569744	1.416459	1.179756	0.277406	0.406729
Novel:chi-miR-326-3p	1.00645	1.726182	1.172793	0.278828	0.407899
Novel:NC_037556.1_28412	0.440121	6.378204	1.169583	0.279487	0.407899
Novel:NC_037547.1_8396	-0.54052	4.98544	1.168091	0.279794	0.407899
Novel:NC_037553.1_22911	0.463919	4.43429	1.168025	0.279807	0.407899
bta-miR-2285q	0.453483	3.341221	1.166094	0.280205	0.407899
bta-miR-450a	-0.89541	2.208435	1.157885	0.281905	0.409787
Novel:hsa-miR-198	0.694804	4.191775	1.151354	0.283266	0.41118
Novel:NC_037551.1_20645	1.103765	1.726629	1.121605	0.289573	0.419737
Novel:hsa-miR-3689d	-0.43872	10.87214	1.119074	0.290118	0.419929
bta-miR-11986b	0.911177	1.693458	1.107395	0.292649	0.422992
bta-miR-33b	0.92843	2.078368	1.082893	0.298051	0.43019
bta-miR-487b	-0.94459	2.133726	1.076973	0.299376	0.43149
Novel:hsa-miR-572	-1.27513	1.636425	1.074268	0.299983	0.431756
Novel:NC_037561.1_36340	1.20183	1.720642	1.070855	0.300752	0.432252
Novel:NC_037545.1_1619	-1.54167	1.43528	1.063567	0.302403	0.434012
Novel:NC_037545.1_137	0.846953	1.837749	1.052357	0.304965	0.437074
bta-miR-149-5p	0.387117	4.632013	1.041039	0.30758	0.439767
bta-miR-139	1.257464	1.739656	1.04049	0.307707	0.439767
bta-miR-376b	-1.43903	1.299621	1.034517	0.3091	0.441139
bta-miR-2285p	1.28066	1.412905	1.026624	0.310953	0.443162
bta-miR-2387	1.518552	1.483673	1.006138	0.31583	0.44929
Novel:hsa-miR-505-3p	-1.29468	1.447944	1.004872	0.316134	0.44929
bta-miR-1291	0.962743	1.520876	0.991355	0.319411	0.453315
Novel:NC_037553.1_24410	-0.75684	1.707308	0.957187	0.327897	0.46471
Novel:NC_037551.1_19470	-1.14272	1.850316	0.942056	0.33175	0.469518
Novel:hsa-miR-6808-3p	0.966737	1.655409	0.921807	0.337001	0.476289
Novel:hsa-miR-3074-5p	0.259056	9.86462	0.914402	0.338949	0.478045
Novel:hsa-miR-6846-5p	1.113432	1.542855	0.913521	0.339182	0.478045
Novel:hsa-miR-4689	-0.48538	3.656043	0.909424	0.340267	0.478912
Novel:NC_037569.1_46653	0.810605	2.166808	0.902408	0.342137	0.480879

Novel:hsa-miR-1251-3p	-0.40723	9.568614	0.894494	0.344262	0.4832
bta-miR-27a-3p	0.196786	8.170203	0.886837	0.346336	0.485086
bta-miR-499	0.906174	1.835727	0.88451	0.346969	0.485086
bta-miR-224	-0.58617	2.598033	0.884272	0.347034	0.485086
bta-miR-6529a	-0.23059	9.333744	0.877959	0.348761	0.486833
bta-miR-365-5p	1.201949	1.42121	0.868972	0.35124	0.489622
bta-miR-10174-3p	0.251127	5.651073	0.848435	0.356995	0.496965
Novel:NC_037558.1_32781	0.314749	8.689513	0.843551	0.358383	0.498216
bta-miR-6529b	-0.22433	9.40993	0.838323	0.359877	0.499465
Novel:NC_037547.1_7477	1.105001	1.411062	0.835781	0.360606	0.499465
Novel:NC_037547.1_9992	0.301454	6.814331	0.834496	0.360976	0.499465
Novel:NC_037558.1_32782	0.313081	8.68859	0.833572	0.361242	0.499465
bta-miR-4286	-1.19131	1.540756	0.830685	0.362075	0.499938
bta-miR-491	-0.98703	1.729786	0.818936	0.365492	0.503973
Novel:NC_037546.1_6394	1.173465	1.451483	0.800028	0.371085	0.510387
bta-miR-191b	-0.54956	3.01138	0.79959	0.371216	0.510387
Novel:NC_037551.1_20226	-1.0528	1.821905	0.798155	0.371646	0.510387
bta-miR-2285bz	0.29342	5.554742	0.787471	0.374866	0.513591
Novel:NC_037552.1_21285	1.001657	1.521653	0.78678	0.375075	0.513591
Novel:NC_037565.1_42436	0.828667	1.790135	0.783906	0.375949	0.513591
Novel:hsa-miR-5681a	-1.6349	1.500385	0.783756	0.375995	0.513591
Novel:NC_037549.1_15054	0.832573	1.83697	0.776621	0.378176	0.515625
Novel:hsa-miR-4761-5p	-1.17799	1.467307	0.775581	0.378496	0.515625
bta-miR-2285z	0.378984	3.386907	0.768883	0.380563	0.517748
bta-miR-2285aw	0.828043	1.638404	0.763655	0.382187	0.518643
Novel:NC_037569.1_46177	-0.66581	1.730344	0.763326	0.38229	0.518643
Novel:NC_037553.1_23515	1.126018	1.539841	0.76186	0.382747	0.518643
bta-miR-148b	0.239468	8.059183	0.756567	0.384405	0.520198
Novel:hsa-miR-12135	0.418799	2.725987	0.753538	0.385358	0.520796
Novel:NC_037564.1_40338	0.917578	1.778492	0.751002	0.386159	0.521084
bta-miR-181b	-0.20476	9.663566	0.749628	0.386594	0.521084
Novel:hsa-miR-3670	0.512654	2.494317	0.7415	0.389181	0.523795
Novel:hsa-miR-7106-5p	1.184163	1.536598	0.737964	0.390314	0.523795
Novel:hsa-miR-7160-3p	-1.51131	1.406318	0.73782	0.39036	0.523795
bta-miR-16b	-0.31096	6.939729	0.734681	0.39137	0.523795
Novel:NC_037556.1_29844	-0.44763	5.821919	0.734473	0.391437	0.523795
Novel:NC_037552.1_21749	0.970302	2.009698	0.732641	0.392029	0.523795
bta-miR-2285ce	-0.3573	2.833263	0.732098	0.392204	0.523795
Novel:NC_037568.1_46017	0.415992	2.602865	0.727725	0.393622	0.52435
Novel:NC_037560.1_35659	0.41599	2.602865	0.727643	0.393648	0.52435
bta-miR-11986c	0.381802	3.25584	0.712778	0.398523	0.530102
Novel:NC_037546.1_4552	-0.55147	2.573276	0.711317	0.399007	0.530102
Novel:NC_037546.1_4856	-0.81939	1.678301	0.708125	0.400067	0.530634
Novel:hsa-miR-6828-3p	-1.41254	1.54772	0.706977	0.400449	0.530634
bta-miR-146b	0.360356	4.309754	0.705398	0.400976	0.530642
Novel:hsa-miR-152-5p	1.424243	1.41053	0.692524	0.405307	0.535678
Novel:NC_037557.1_30645	0.70473	2.361504	0.673552	0.411816	0.543576
Novel:hsa-miR-4668-3p	-0.79569	1.595782	0.668835	0.413458	0.545037
bta-miR-374b	-0.35717	3.207183	0.659082	0.416884	0.548844

bta-miR-147	-0.78278	1.927599	0.646249	0.421457	0.554148
Novel:NC_037558.1_33240	1.387331	1.381758	0.629372	0.427586	0.561482
bta-miR-190b	-0.30869	3.308115	0.623093	0.4299	0.563794
Novel:chi-miR-3432-5p	0.623295	2.92203	0.615416	0.432756	0.566061
Novel:NC_037556.1_30278	-0.93807	1.750098	0.615008	0.432909	0.566061
Novel:NC_037565.1_42764	-1.3127	1.449241	0.613974	0.433295	0.566061
Novel:NC_037566.1_42967	-0.48501	2.701115	0.611166	0.434349	0.566712
Novel:hsa-miR-6089	-0.42743	2.856436	0.601637	0.437954	0.570262
bta-miR-433	1.013048	1.556832	0.601018	0.43819	0.570262
Novel:NC_037550.1_18169	-0.83791	1.427537	0.598047	0.439324	0.571009
Novel:NC_037545.1_558	0.449279	3.198568	0.589793	0.442499	0.57379
Novel:NC_037551.1_19577	1.316159	1.390927	0.58956	0.442589	0.57379
bta-miR-545-5p	0.616932	2.194858	0.584816	0.444431	0.575445
Novel:NC_037564.1_40741	0.631118	2.144281	0.577623	0.447245	0.578354
bta-miR-2483-3p	-0.21204	5.930042	0.575997	0.447886	0.578448
Novel:NC_037568.1_45672	1.069785	1.571002	0.572736	0.449174	0.579121
bta-miR-885	0.876921	1.553142	0.571803	0.449543	0.579121
Novel:hsa-miR-4706	-0.79647	1.504386	0.56803	0.451043	0.580144
bta-miR-202	0.289282	9.48039	0.566942	0.451476	0.580144
Novel:hsa-miR-448	1.148759	1.366666	0.559024	0.454654	0.582578
bta-miR-154c	-0.29487	3.953832	0.558535	0.454851	0.582578
Novel:hsa-miR-766-5p	0.736125	1.975635	0.557955	0.455085	0.582578
bta-miR-374a	0.492428	2.607325	0.556385	0.45572	0.582659
bta-miR-2403	-0.42671	2.600135	0.553281	0.45698	0.583538
Novel:NC_037546.1_6618	-0.48055	2.502173	0.544663	0.460507	0.587305
Novel:NC_037569.1_48203	0.711211	1.88214	0.538879	0.462898	0.589616
bta-miR-370	-0.64126	2.262877	0.536084	0.46406	0.590359
Novel:NC_037569.1_46552	0.274071	6.856453	0.52573	0.468407	0.595146
Novel:hsa-miR-9900	-1.19781	1.507048	0.523787	0.469231	0.59545
bta-miR-1468	-0.27686	5.855284	0.515811	0.472634	0.598647
Novel:hsa-miR-545-3p	-0.27718	5.858608	0.515133	0.472925	0.598647
bta-miR-449c	0.945603	1.375909	0.510518	0.474915	0.600419
Novel:chi-miR-499-3p	0.643077	1.90261	0.508308	0.475872	0.600885
bta-miR-23b-3p	0.22862	4.901706	0.49464	0.481865	0.607698
bta-miR-331-5p	0.228934	5.283801	0.491493	0.483262	0.608115
Novel:NC_037560.1_35002	-0.63783	1.662176	0.491209	0.483388	0.608115
Novel:NC_037562.1_38494	0.475125	2.320962	0.488739	0.48449	0.608749
Novel:hsa-miR-943	-0.54997	2.019843	0.48366	0.486769	0.610161
Novel:hsa-miR-4707-5p	-0.56158	1.872888	0.483567	0.486811	0.610161
Novel:NC_037547.1_8180	0.727982	1.75354	0.473797	0.491246	0.614962
Novel:NC_037553.1_23471	-0.83642	1.614853	0.460921	0.497194	0.620541
Novel:NC_037550.1_18493	0.660391	1.464322	0.458826	0.498173	0.620541
Novel:NC_037550.1_17382	0.660388	1.464322	0.458706	0.49823	0.620541
Novel:NC_037547.1_7908	-0.69112	1.392902	0.457923	0.498596	0.620541
Novel:NC_037547.1_9916	-0.69116	1.392902	0.457601	0.498747	0.620541
Novel:hsa-miR-2355-5p	0.783414	1.516473	0.448791	0.50291	0.624957
bta-miR-2355-3p	-0.76999	1.444529	0.437104	0.508524	0.631164
Novel:hsa-miR-4479	-0.78247	1.479939	0.429232	0.512366	0.635159
Novel:NC_037557.1_30641	-0.3332	4.284197	0.413301	0.520299	0.642806

bta-miR-664b	-0.62582	1.867459	0.41312	0.52039	0.642806
Novel:NC_037549.1_16296	0.487655	2.251592	0.413047	0.520427	0.642806
Novel:hsa-miR-2110	0.704623	1.929851	0.405124	0.524455	0.646997
Novel:NC_037552.1_21636	-0.71737	1.650839	0.403571	0.525251	0.647196
bta-miR-450b	-0.21684	7.936354	0.39966	0.527265	0.648523
bta-miR-744	-0.1486	7.10509	0.399009	0.527602	0.648523
Novel:chi-miR-2331	-0.53143	2.374601	0.397352	0.52846	0.648796
bta-miR-2285v	0.478199	1.993072	0.396077	0.529122	0.648828
Novel:NC_037549.1_16572	0.548815	1.89383	0.378523	0.538395	0.658479
Novel:hsa-miR-4721	0.660791	1.571524	0.377527	0.53893	0.658479
bta-miR-6517	0.1547	5.921419	0.377523	0.538932	0.658479
Novel:hsa-miR-762	-0.96901	1.342779	0.373029	0.541358	0.659342
Novel:chi-miR-432-5p	-0.96902	1.342779	0.372746	0.541511	0.659342
Novel:NC_037569.1_47131	-0.39765	2.622074	0.372621	0.541579	0.659342
bta-miR-20a	-0.31116	4.2644	0.367874	0.544165	0.6617
bta-miR-2285db	0.824809	1.447601	0.363201	0.546734	0.664031
Novel:NC_037551.1_20840	-0.93447	1.631378	0.352697	0.55259	0.670345
bta-miR-2285cr	0.392218	2.245314	0.349017	0.55467	0.671421
bta-miR-877	0.195448	5.469214	0.348796	0.554795	0.671421
Novel:NC_037550.1_17830	0.494645	1.794274	0.345192	0.556847	0.673105
bta-miR-296-5p	-0.56121	1.667345	0.342461	0.558412	0.674181
Novel:hsa-miR-3129-3p	-0.29125	7.652087	0.341333	0.559061	0.674181
Novel:hsa-miR-1827	0.64157	1.493237	0.338335	0.560792	0.675469
Novel:NC_037550.1_18986	0.497231	1.794213	0.336477	0.56187	0.675968
Novel:NC_037565.1_42179	-0.41866	3.345401	0.331755	0.564627	0.678485
bta-miR-3578	0.337582	3.184512	0.327375	0.567209	0.680784
Novel:hsa-miR-4467	0.5945	1.637615	0.325793	0.568147	0.681108
Novel:hsa-miR-3202	1.077754	1.442831	0.321347	0.570799	0.683072
Novel:hsa-miR-1321	0.50877	1.79282	0.320803	0.571126	0.683072
bta-miR-2285ad	0.517839	1.61693	0.318319	0.572619	0.684055
Novel:hsa-miR-4677-3p	0.600055	1.646145	0.313816	0.575348	0.68651
Novel:hsa-miR-6501-3p	-0.76157	1.661823	0.301908	0.582689	0.694121
Novel:NC_037558.1_31787	-0.76145	1.661823	0.301268	0.583089	0.694121
bta-miR-2411-3p	-0.74699	1.30351	0.298202	0.585012	0.694904
bta-miR-34a	-0.27127	2.886172	0.29774	0.585303	0.694904
Novel:NC_037547.1_6928	-1.11606	1.851927	0.296964	0.585792	0.694904
Novel:NC_037561.1_36265	-0.26695	4.383253	0.294072	0.587623	0.696215
Novel:hsa-miR-4663	-0.58348	1.718108	0.293063	0.588264	0.696215
Novel:NC_037547.1_7559	-0.12737	7.074503	0.289694	0.590417	0.697952
bta-miR-2330-5p	0.659758	1.3459	0.282586	0.595011	0.702569
bta-miR-3120	0.612696	1.842699	0.272246	0.601829	0.709796
bta-miR-502b	-0.17857	3.605912	0.270763	0.60282	0.709866
Novel:hsa-miR-3144-5p	0.391835	1.760289	0.270075	0.603282	0.709866
Novel:NC_037553.1_24557	0.5861	1.553285	0.268907	0.604066	0.709969
Novel:hsa-miR-4723-5p	0.181169	13.21151	0.266831	0.605465	0.710794
Novel:hsa-miR-5739	-0.71508	1.434974	0.258012	0.611489	0.71704
bta-miR-93	0.106848	9.006016	0.252459	0.615349	0.720736
Novel:hsa-miR-4675	0.244703	3.422662	0.249402	0.617496	0.722254
Novel:NC_037568.1_44905	0.106253	8.99546	0.2486	0.618063	0.722254

Novel:hsa-miR-4753-5p	-0.28724	2.539455	0.247496	0.618844	0.722339
Novel:hsa-miR-4641	-0.66514	1.410187	0.246048	0.619872	0.722711
bta-miR-452	-0.2974	2.850375	0.243301	0.621832	0.724168
Novel:NC_037557.1_30647	-0.19137	4.710252	0.232942	0.629351	0.732087
Novel:hsa-miR-3654	0.500376	2.845149	0.227671	0.633257	0.735791
bta-miR-2284p	-0.4352	1.531315	0.22505	0.635219	0.736701
bta-miR-382	0.278369	3.185106	0.22385	0.636122	0.736701
Novel:NC_037568.1_45170	-1.02419	1.532359	0.223735	0.636209	0.736701
bta-miR-184	0.472942	1.585598	0.221628	0.637802	0.737708
bta-miR-2285ar	-0.53492	1.479676	0.214108	0.643566	0.74353
bta-miR-2285bn	-0.17945	3.102633	0.20061	0.654229	0.754994
bta-miR-326	0.167208	3.364327	0.197589	0.656674	0.756958
Novel:chi-miR-485-5p	0.355116	2.290288	0.192978	0.660449	0.760449
bta-miR-127	-0.1126	10.30026	0.189578	0.663268	0.762833
Novel:NC_037550.1_19148	0.64888	1.472719	0.179713	0.67162	0.771568
bta-miR-874	-0.13974	4.531472	0.172904	0.677544	0.777497
bta-miR-100	0.151103	9.69826	0.170814	0.679389	0.778737
Novel:NC_037560.1_35884	0.147623	9.68554	0.160538	0.688662	0.788479
bta-miR-323b-3p	-0.21547	2.709811	0.158019	0.690987	0.790254
bta-miR-2887	0.151228	3.505352	0.152547	0.696113	0.795223
Novel:NC_037550.1_18703	-0.46031	1.522627	0.149848	0.69868	0.797262
Novel:hsa-miR-6071	0.373845	1.512669	0.141615	0.706681	0.80549
bta-miR-655	0.451581	1.473302	0.139876	0.708405	0.806552
Novel:hsa-miR-3921	-0.46947	1.704457	0.136245	0.712043	0.808296
Novel:NC_037547.1_10096	-0.33677	2.051234	0.135985	0.712305	0.808296
Novel:NC_037549.1_14335	-0.56614	1.414189	0.134999	0.713305	0.808296
bta-miR-2340	-0.44492	1.460292	0.134895	0.71341	0.808296
bta-miR-2898	-0.25699	2.055909	0.134411	0.713902	0.808296
Novel:NC_037564.1_40376	0.101515	7.462055	0.132416	0.71594	0.808857
bta-miR-15a	-0.10287	6.635126	0.131963	0.716405	0.808857
bta-miR-214	-0.11065	4.734748	0.1316	0.716779	0.808857
Novel:hsa-miR-10226	-0.71499	1.506647	0.125534	0.723109	0.814774
Novel:NC_037563.1_40142	0.547126	1.390429	0.125049	0.723622	0.814774
Novel:NC_037548.1_12197	0.136914	3.095342	0.122777	0.726042	0.816597
bta-miR-411c-5p	0.495302	1.681055	0.121273	0.727658	0.817513
Novel:hsa-miR-6780b-3p	-0.3523	1.554953	0.119174	0.729933	0.818321
Novel:NC_037556.1_29648	0.318216	1.857184	0.118652	0.730501	0.818321
bta-miR-485	-0.36064	1.510025	0.11839	0.730787	0.818321
Novel:NC_037546.1_5469	0.398839	1.636961	0.114781	0.734765	0.821585
Novel:NC_037548.1_11085	-0.64912	1.370359	0.114288	0.735314	0.821585
Novel:NC_037561.1_36929	-0.30494	1.850159	0.113408	0.736297	0.821781
Novel:hsa-miR-6124	-0.21025	3.793067	0.104073	0.746995	0.832393
bta-let-7a-3p	0.118471	4.745539	0.103697	0.747438	0.832393
Novel:NC_037564.1_40336	0.322503	1.517282	0.101442	0.750106	0.834452
bta-miR-9-5p	0.489781	1.58927	0.099742	0.75214	0.835802
Novel:NC_037567.1_44553	0.130344	4.017203	0.098881	0.753177	0.835879
Novel:hsa-miR-135a-3p	-0.43191	1.449439	0.098054	0.754177	0.835879
Novel:NC_037554.1_25273	-0.30411	1.965587	0.097648	0.75467	0.835879
Novel:hsa-miR-4468	0.333568	1.615455	0.094723	0.758257	0.837935

bta-miR-2285s	-0.2081	2.572123	0.094199	0.758906	0.837935
Novel:NC_037549.1_17067	-0.1295	5.627293	0.094128	0.758993	0.837935
Novel:NC_037553.1_23783	0.09886	5.853488	0.09341	0.759886	0.838012
Novel:hsa-miR-1324	0.497607	1.407877	0.091525	0.762247	0.839708
bta-miR-122	-0.29094	1.859118	0.090778	0.76319	0.839838
Novel:hsa-miR-1290	-0.17341	3.488695	0.088268	0.76639	0.842451
bta-miR-32	-0.06821	6.432966	0.085463	0.770026	0.844936
bta-miR-17-5p	-0.11518	4.720531	0.085246	0.77031	0.844936
Novel:NC_037555.1_26952	-0.44962	1.304828	0.084551	0.771222	0.84495
bta-miR-495	0.259062	1.606526	0.083463	0.772659	0.84495
bta-miR-30e-5p	0.059669	11.10631	0.082623	0.773774	0.84495
bta-miR-503-3p	0.182766	2.669537	0.082176	0.77437	0.84495
Novel:chi-miR-22-5p	0.175187	2.351536	0.082102	0.774469	0.84495
Novel:oar-miR-3957-3p	-0.36787	1.508842	0.080774	0.776252	0.84599
Novel:NC_037569.1_48122	0.375878	1.572726	0.079656	0.777764	0.846732
bta-miR-219b-3p	0.293083	1.81252	0.07858	0.779232	0.847425
Novel:hsa-miR-4687-5p	-0.16845	2.324861	0.075641	0.783294	0.850935
bta-miR-196a	0.297062	1.459675	0.074527	0.784856	0.851101
Novel:NC_037569.1_47135	-0.28016	1.952805	0.074341	0.785118	0.851101
bta-miR-2484	-0.30776	1.661976	0.0737	0.786024	0.851178
Novel:NC_037565.1_42322	-0.15953	2.972778	0.071924	0.788556	0.853013
Novel:chi-miR-671-5p	-0.16069	2.253083	0.070811	0.790159	0.853841
Novel:NC_037546.1_5830	-0.08913	4.541532	0.070174	0.791084	0.853935
Novel:NC_037564.1_40975	-0.28994	1.499465	0.064384	0.799696	0.862318
Novel:hsa-miR-4802-5p	-0.38747	1.478286	0.062042	0.803297	0.865285
Novel:NC_037556.1_28514	0.349749	1.407047	0.060306	0.806013	0.867294
bta-miR-2285dh	0.087295	3.496263	0.059022	0.808048	0.868225
Novel:hsa-miR-3529-3p	0.286872	1.379496	0.058688	0.808582	0.868225
bta-miR-2285br	0.157727	2.331758	0.057004	0.811295	0.870221
Novel:hsa-miR-6755-5p	-0.17572	2.005353	0.05495	0.814663	0.872915
Novel:hsa-miR-6499-5p	0.10089	4.063658	0.052908	0.818079	0.875435
Novel:NC_037555.1_27132	0.294423	1.544503	0.051821	0.819924	0.875435
Novel:NC_037551.1_19765	-0.07624	4.535861	0.051676	0.820172	0.875435
Novel:hsa-miR-548as-3p	0.076414	4.406561	0.051512	0.820452	0.875435
Novel:NC_037555.1_28111	0.080437	3.573876	0.050549	0.822111	0.875821
bta-miR-299	0.279095	1.374595	0.050103	0.822885	0.875821
bta-miR-652	-0.06521	6.101514	0.049811	0.823392	0.875821
Novel:hsa-miR-6787-3p	-0.31932	1.424532	0.048901	0.824987	0.876602
Novel:hsa-miR-12136	-0.07056	4.501981	0.047541	0.827399	0.878249
bta-miR-12031	0.274058	1.541212	0.043712	0.834391	0.884749
Novel:NC_037547.1_8774	0.321892	1.59016	0.042728	0.836238	0.885
Novel:NC_037553.1_24219	-0.15543	1.872761	0.042661	0.836364	0.885
Novel:chi-miR-22-3p	0.111052	2.359169	0.041954	0.837707	0.885502
bta-miR-181c	0.072802	4.207374	0.038638	0.844168	0.890371
bta-miR-362-5p	-0.30189	1.385905	0.038311	0.84482	0.890371
Novel:NC_037561.1_36447	0.307932	1.505557	0.037702	0.846042	0.890371
bta-miR-12057	0.155638	1.956092	0.03765	0.846148	0.890371
Novel:NC_037555.1_27691	-0.17271	1.900934	0.037386	0.846682	0.890371
Novel:NC_037559.1_33762	-0.22679	1.700942	0.034353	0.852959	0.895331

Novel:NC_037567.1_44107	0.074729	4.009912	0.034259	0.853157	0.895331
Novel:hsa-miR-1289	0.224274	1.434187	0.031831	0.8584	0.899907
Novel:hsa-miR-4537	0.134093	2.452119	0.030736	0.860831	0.901528
Novel:hsa-miR-3714	-0.08485	9.776923	0.028911	0.864986	0.904949
Novel:hsa-miR-6765-3p	0.103574	3.063649	0.027993	0.867125	0.906221
bta-miR-7863	0.160784	1.763608	0.02763	0.867981	0.906221
Novel:hsa-miR-4428	-0.07899	6.013551	0.026917	0.86968	0.907066
bta-miR-3596	0.036998	10.58291	0.025526	0.873064	0.908864
bta-miR-301b	-0.13574	1.893368	0.025476	0.873187	0.908864
Novel:NC_037547.1_10878	-0.20501	1.655689	0.024154	0.876492	0.911373
Novel:NC_037558.1_32628	0.091582	2.392562	0.023559	0.878013	0.912024
Novel:NC_037561.1_36813	-0.15439	1.680072	0.023131	0.879118	0.912241
bta-miR-551b	-0.0722	2.496532	0.021541	0.883313	0.915663
bta-miR-324	-0.12875	2.412198	0.019815	0.888055	0.919642
Novel:hsa-miR-21-3p	-0.12723	1.901793	0.018241	0.892565	0.923374
bta-miR-2299-5p	0.063179	3.614711	0.017283	0.895408	0.925376
Novel:chi-miR-125a-3p	0.110983	1.505696	0.016929	0.896478	0.925543
Novel:hsa-miR-3672	-0.0481	10.00107	0.015907	0.899634	0.927862
Novel:hsa-miR-6890-3p	0.265809	1.462249	0.015362	0.901361	0.928702
Novel:NC_037549.1_16794	-0.15028	1.771997	0.014236	0.905026	0.931537
Novel:hsa-miR-6781-5p	-0.07759	1.945428	0.011417	0.914909	0.940759
bta-let-7d	0.043585	4.513162	0.010746	0.917436	0.942406
bta-miR-767	0.096392	1.849266	0.009813	0.921091	0.94443
Novel:chi-miR-874-5p	0.135883	1.453524	0.009553	0.922137	0.94443
bta-miR-6119-3p	-0.10015	1.634051	0.009541	0.922186	0.94443
bta-miR-365-3p	0.056026	3.434738	0.008414	0.926913	0.947801
bta-miR-2415-3p	0.072527	1.820711	0.008316	0.927338	0.947801
Novel:NC_037546.1_2774	-0.07983	1.507721	0.006737	0.934583	0.954248
Novel:NC_037561.1_37123	-0.16114	1.574776	0.006479	0.935847	0.954583
bta-miR-200a	-0.03941	3.182861	0.005898	0.938785	0.956622
Novel:NC_037546.1_4148	-0.07938	1.41638	0.004469	0.9467	0.963724
bta-miR-23a	0.017223	6.473372	0.004183	0.948433	0.964524
Novel:hsa-miR-6747-3p	-0.03692	2.874572	0.003975	0.949728	0.964878
Novel:hsa-miR-548d-3p	-0.06103	1.472419	0.003362	0.953759	0.968009
Novel:NC_037559.1_34348	-0.0712	1.625116	0.002741	0.958247	0.971595
Novel:hsa-miR-658	-0.07809	1.354872	0.002339	0.961426	0.97385
Novel:NC_037551.1_19485	0.026638	2.238591	0.002113	0.963339	0.974225
Novel:hsa-miR-4691-3p	0.020426	4.099085	0.00207	0.963708	0.974225
bta-miR-12017	0.08615	1.648496	0.001761	0.96653	0.976109
Novel:NC_037547.1_9224	-0.0439	1.956295	0.00153	0.968798	0.977431
Novel:hsa-miR-3960	-0.03268	2.469183	0.001192	0.972455	0.98015
bta-miR-2285by	-0.01454	2.73102	0.00097	0.975154	0.981899
Novel:NC_037566.1_43651	-0.02426	1.480162	0.000196	0.988832	0.993582
Novel:NC_037566.1_43159	-0.02425	1.480162	0.000196	0.988836	0.993582
bta-miR-494	0.010311	2.770627	0.000167	0.989682	0.993582
bta-miR-2378	-0.00087	1.483677	0.000124	0.99112	0.994047
bta-miR-345-3p	0.002161	7.322487	5.83E-05	0.99391	0.995496
Novel:NC_037568.1_45368	-0.00438	2.326407	4.72E-05	0.994519	0.995496
Novel:NC_037545.1_1635	0.000757	3.032063	1.82E-05	0.996598	0.996598

An&pO(NBS_vs_BS)	logFC	logCPM	LR	PValue	FDR
Novel:NC_037567.1_44155	-5.13748	2.056331	11.9038	0.00056	0.363404
Novel:NC_037560.1_35256	-3.73252	1.59603	11.22143	0.000809	0.363404
bta-miR-2285bf	-4.30231	1.843113	10.19241	0.00141	0.363404
Novel:NC_037552.1_22617	-1.95475	3.143943	10.04727	0.001526	0.363404
Novel:NC_037555.1_27452	-2.11192	2.208171	9.4523	0.002109	0.363404
bta-miR-655	-3.341	1.474007	9.378459	0.002196	0.363404
Novel:NC_037565.1_42179	-2.10045	3.367085	9.143235	0.002496	0.363404
Novel:hsa-miR-4783-5p	-2.24786	3.237171	7.93582	0.004847	0.513619
Novel:hsa-miR-3714	-1.37382	9.776439	7.89781	0.004949	0.513619
Novel:NC_037557.1_30571	4.241668	1.613441	7.697536	0.00553	0.513619
bta-miR-324	2.212794	2.425655	7.692699	0.005544	0.513619
Novel:hsa-miR-6734-3p	-1.39301	7.950726	7.509826	0.006136	0.521077
Novel:hsa-miR-548u	-4.09184	1.620337	7.324604	0.006802	0.533148
Novel:NC_037546.1_4148	-3.00425	1.403333	6.675403	0.009775	0.627202
Novel:hsa-miR-4641	-3.01503	1.403115	6.491439	0.01084	0.627202
Novel:NC_037553.1_24733	-2.84957	1.659445	6.440606	0.011154	0.627202
Novel:NC_037568.1_45170	4.073781	1.57035	6.430787	0.011216	0.627202
Novel:hsa-miR-6077	-2.86362	2.233312	6.400814	0.011407	0.627202
bta-miR-11986c	-1.11744	3.243416	6.356585	0.011695	0.627202
Novel:hsa-miR-10226	3.769501	1.532612	6.221592	0.01262	0.642997
bta-miR-200a	-1.21273	3.170673	5.881065	0.015305	0.670539
Novel:NC_037558.1_32628	-1.40192	2.378134	5.822241	0.015825	0.670539
Novel:NC_037556.1_28345	-2.27096	1.643088	5.758337	0.016411	0.670539
Novel:NC_037556.1_28346	-2.27065	1.643088	5.750749	0.016482	0.670539
bta-miR-485	-2.33434	1.511239	5.7494	0.016494	0.670539
Novel:hsa-miR-766-5p	-2.09555	1.960906	5.490279	0.019122	0.670539
Novel:NC_037546.1_4277	-2.24129	1.947836	5.45947	0.019463	0.670539
Novel:NC_037561.1_36258	-1.49064	2.860777	5.429864	0.019795	0.670539
bta-miR-487b	-2.16875	2.139653	5.383213	0.020331	0.670539
Novel:NC_037564.1_40932	-2.92766	1.682084	5.373934	0.02044	0.670539
Novel:NC_037565.1_41975	-3.69771	1.518043	5.370091	0.020485	0.670539
Novel:NC_037565.1_42436	-1.95573	1.765275	5.225864	0.022253	0.670539
Novel:hsa-miR-658	2.808417	1.350362	5.219317	0.022337	0.670539
Novel:chi-miR-22-5p	-1.33901	2.336291	5.216522	0.022373	0.670539
Novel:NC_037568.1_45368	-1.83961	2.304425	5.104198	0.023868	0.682032
bta-miR-138	-2.43073	1.622623	5.060951	0.024471	0.682032
Novel:NC_037550.1_18703	2.344813	1.521966	4.97298	0.025746	0.682032
Novel:hsa-miR-6777-5p	-1.5162	9.585945	4.879158	0.027183	0.682032
bta-miR-483	-2.13872	1.689586	4.842656	0.027764	0.682032
Novel:NC_037547.1_10260	-2.10691	3.661737	4.82412	0.028064	0.682032
bta-miR-2285af	-2.77692	1.367614	4.823003	0.028082	0.682032
bta-miR-2881	-2.78747	1.334435	4.821234	0.028111	0.682032
bta-miR-28	0.941961	3.962806	4.660989	0.030855	0.731202
Novel:hsa-miR-6765-3p	1.277909	3.052786	4.620693	0.031588	0.73156
Novel:NC_037561.1_36265	-1.05286	4.382779	4.509304	0.033711	0.737991
Novel:NC_037556.1_29648	-1.87479	1.842136	4.45442	0.034811	0.737991
Novel:NC_037548.1_11583	-2.72623	1.730947	4.42493	0.035418	0.737991
Novel:hsa-miR-3129-3p	1.061453	7.651245	4.383313	0.036292	0.737991

Novel:hsa-miR-6501-3p	-2.79853	1.654852	4.377577	0.036415	0.737991
Novel:NC_037558.1_31787	-2.79846	1.654852	4.374987	0.03647	0.737991
Novel:chi-miR-24-5p	0.943766	6.443426	4.346781	0.037079	0.737991
Novel:NC_037548.1_12197	-0.81921	3.084852	4.320314	0.03766	0.737991
Novel:NC_037549.1_16021	-1.34923	3.45145	4.265057	0.038904	0.747983
Novel:hsa-miR-1908-3p	1.488537	3.511269	4.186556	0.040746	0.768888
bta-miR-454	2.712598	1.944047	4.108814	0.04266	0.782882
Novel:NC_037545.1_1896	-1.97157	1.586713	4.09446	0.043024	0.782882
Novel:NC_037562.1_38397	-2.68771	2.757499	4.052545	0.044105	0.784493
Novel:NC_037562.1_38494	1.30323	2.327732	4.031728	0.044652	0.784493
bta-miR-194b	2.503111	1.78393	3.967671	0.046382	0.794941
Novel:NC_037547.1_7828	-0.95275	3.397395	3.925362	0.047563	0.794941
Novel:hsa-miR-203a-5p	-2.49553	2.32601	3.90578	0.04812	0.794941
bta-miR-12017	3.185531	1.686101	3.897175	0.048367	0.794941
bta-let-7e	0.895048	5.930813	3.804602	0.051112	0.826715
Novel:NC_037550.1_18832	1.703132	2.207845	3.747619	0.052883	0.832632
Novel:NC_037569.1_46660	-1.90319	4.124395	3.725789	0.053578	0.832632
Novel:hsa-miR-6727-5p	1.015192	10.86907	3.714894	0.053929	0.832632
Novel:NC_037553.1_24374	-0.76998	4.481959	3.662847	0.055638	0.845101
bta-miR-451	1.677086	3.274462	3.640342	0.056395	0.845101
Novel:NC_037549.1_14559	-2.53562	1.553515	3.564737	0.059019	0.871601
Novel:NC_037550.1_18158	-0.94819	7.847093	3.516671	0.060754	0.881085
bta-miR-433	2.121724	1.555357	3.485538	0.061907	0.881085
Novel:hsa-miR-545-3p	0.760347	5.864573	3.476261	0.062255	0.881085
Novel:NC_037569.1_47154	-0.96054	6.890802	3.376408	0.066136	0.890662
bta-miR-193a	-0.98094	3.879755	3.364195	0.066628	0.890662
bta-miR-652	0.603039	6.101072	3.360608	0.066773	0.890662
Novel:hsa-miR-6781-5p	1.385876	1.964148	3.351452	0.067146	0.890662
Novel:NC_037555.1_27691	-1.6215	1.897328	3.310567	0.068836	0.890662
bta-miR-9-5p	2.663643	1.579325	3.292929	0.069579	0.890662
Novel:hsa-miR-1252-3p	1.209083	2.482281	3.251263	0.071368	0.890662
Novel:hsa-miR-548d-3p	-1.6407	1.470099	3.232256	0.072201	0.890662
bta-miR-11980	1.448043	2.077619	3.225834	0.072485	0.890662
Novel:hsa-miR-4520-3p	-0.84868	5.747004	3.199266	0.073671	0.890662
Novel:hsa-miR-3909	-0.93228	3.500531	3.192146	0.073993	0.890662
Novel:NC_037547.1_10839	-2.63322	1.592886	3.145945	0.076116	0.890662
Novel:NC_037554.1_24951	-0.55384	6.40819	3.142426	0.07628	0.890662
Novel:NC_037545.1_1619	2.503684	1.450795	3.125336	0.077084	0.890662
Novel:NC_037547.1_10102	-1.23906	2.521927	3.118526	0.077407	0.890662
Novel:NC_037547.1_8560	-0.53224	6.486184	3.106951	0.077959	0.890662
Novel:hsa-miR-6068	-0.925	6.739787	3.104234	0.078089	0.890662
Novel:NC_037567.1_44553	-0.75233	3.999851	3.09227	0.078665	0.890662
Novel:NC_037547.1_10537	-0.53994	6.484622	3.056317	0.080424	0.900569
bta-miR-1468	0.712452	5.861352	3.019973	0.082245	0.904295
Novel:hsa-miR-1290	1.016715	3.485556	3.004821	0.083017	0.904295
Novel:NC_037567.1_44107	-0.72152	3.994347	2.97979	0.08431	0.904295
bta-miR-2285bw	-0.76751	3.952079	2.941871	0.086311	0.904295
bta-miR-1260b	2.17858	1.87209	2.923649	0.08729	0.904295
Novel:hsa-miR-505-3p	1.964708	1.456289	2.913877	0.087821	0.904295

Novel:NC_037564.1_40741	-1.3895	2.142565	2.913368	0.087848	0.904295
bta-miR-2312	-1.46268	1.642022	2.913227	0.087856	0.904295
Novel:hsa-miR-4434	-2.47114	1.552058	2.881161	0.089621	0.909694
Novel:hsa-miR-8057	-0.54245	8.37447	2.857212	0.090965	0.909694
Novel:chi-miR-7-5p	-1.4066	2.021781	2.855557	0.091059	0.909694
bta-miR-2285by	0.77061	2.733394	2.835507	0.092202	0.91217
bta-miR-191	0.778984	12.0314	2.806193	0.093901	0.914033
Novel:hsa-miR-6818-3p	-2.06028	1.767702	2.786948	0.095035	0.914033
Novel:NC_037556.1_29821	2.107568	1.523212	2.78516	0.095141	0.914033
bta-miR-2468	-1.06448	2.587083	2.771137	0.095978	0.914033
Novel:NC_037547.1_8583	-0.71007	5.139784	2.692019	0.100852	0.951557
Novel:hsa-miR-652-5p	0.623051	5.483182	2.640299	0.104184	0.95882
Novel:NC_037559.1_33762	1.842635	1.702476	2.620364	0.1055	0.95882
bta-miR-2285s	-1.12398	2.566143	2.618597	0.105618	0.95882
bta-miR-2285ce	-0.71419	2.836961	2.600488	0.106831	0.95882
Novel:NC_037566.1_43265	-0.82426	2.5867	2.600043	0.106861	0.95882
Novel:NC_037557.1_30645	-1.34978	2.340362	2.588833	0.10762	0.95882
Novel:hsa-miR-6747-3p	0.90394	2.884432	2.547848	0.110445	0.95882
bta-miR-31	0.494345	8.007254	2.520355	0.112385	0.95882
Novel:NC_037548.1_12074	-0.76158	9.080151	2.494852	0.114219	0.95882
Novel:NC_037562.1_38320	-1.76906	1.335823	2.486885	0.114799	0.95882
bta-miR-381	-0.61098	6.989487	2.475572	0.115627	0.95882
Novel:NC_037569.1_46587	-1.10065	4.418789	2.444024	0.117973	0.95882
Novel:NC_037557.1_31157	-1.1282	2.375427	2.427792	0.119201	0.95882
bta-miR-486	0.8138	11.54227	2.404547	0.120983	0.95882
bta-miR-1247-3p	-0.79243	3.414497	2.366769	0.123943	0.95882
Novel:NC_037564.1_40326	-0.59557	7.07666	2.348986	0.125365	0.95882
Novel:chi-miR-145-3p	-0.84863	5.571732	2.3436	0.125799	0.95882
Novel:hsa-miR-4802-5p	2.057328	1.483793	2.335806	0.12643	0.95882
bta-miR-2285bo	1.802802	1.673312	2.330725	0.126843	0.95882
bta-miR-2284y	-0.73735	4.858112	2.325324	0.127284	0.95882
Novel:NC_037547.1_10214	0.484238	8.163724	2.320249	0.1277	0.95882
bta-miR-374b	0.7164	3.203824	2.315493	0.128091	0.95882
Novel:NC_037547.1_9992	0.556792	6.812469	2.312051	0.128375	0.95882
bta-miR-2898	1.12386	2.065565	2.309115	0.128617	0.95882
Novel:NC_037545.1_315	-1.26454	2.06081	2.307081	0.128786	0.95882
bta-miR-491	1.72421	1.755214	2.291203	0.130109	0.95882
Novel:NC_037553.1_23290	0.89211	2.651417	2.290637	0.130157	0.95882
Novel:chi-miR-214-3p	-2.22088	1.910503	2.269493	0.131943	0.95882
Novel:NC_037556.1_28412	-0.65684	6.373897	2.255209	0.133166	0.95882
Novel:NC_037550.1_18169	-1.68831	1.428072	2.254468	0.133229	0.95882
bta-miR-409b	-0.60101	7.848176	2.242654	0.13425	0.95882
Novel:NC_037545.1_1689	-1.20463	2.082906	2.209801	0.137136	0.95882
bta-let-7d	0.639716	4.5025	2.208993	0.137208	0.95882
Novel:hsa-miR-1265	-0.63017	11.04828	2.203845	0.137667	0.95882
bta-miR-2285au	1.569734	1.879806	2.19691	0.138288	0.95882
Novel:NC_037563.1_39389	-2.38319	1.605404	2.180538	0.139766	0.95882
Novel:NC_037553.1_24652	-0.65069	4.589502	2.171764	0.140565	0.95882
Novel:NC_037553.1_23542	-0.66349	4.641846	2.166639	0.141034	0.95882

bta-miR-375	0.638673	7.211338	2.160057	0.141639	0.95882
bta-miR-141	0.717463	4.163821	2.153401	0.142254	0.95882
Novel:hsa-miR-6770-5p	-2.08605	1.866676	2.140392	0.143465	0.95882
Novel:NC_037564.1_41393	-1.32403	2.227126	2.136371	0.143842	0.95882
Novel:hsa-miR-4428	-0.73446	6.01139	2.133384	0.144122	0.95882
Novel:hsa-miR-4761-5p	-1.82422	1.475706	2.105678	0.146753	0.95882
bta-miR-362-3p	-0.82929	3.293233	2.088025	0.148458	0.95882
Novel:NC_037549.1_15945	1.924156	1.709236	2.087171	0.148541	0.95882
Novel:hsa-miR-7108-3p	-0.65707	11.40715	2.078431	0.149394	0.95882
Novel:chi-let-7i-3p	1.794501	2.076562	2.077225	0.149512	0.95882
Novel:NC_037557.1_30447	-1.03263	2.368975	2.075492	0.149682	0.95882
Novel:NC_037558.1_32842	-0.71582	4.173153	2.073902	0.149838	0.95882
Novel:NC_037546.1_4225	1.003061	2.874109	2.060782	0.151133	0.95882
bta-miR-26a	0.48453	11.05756	2.052986	0.151908	0.95882
Novel:chi-miR-3958-5p	0.855137	5.106169	2.05289	0.151918	0.95882
bta-miR-26c	0.482887	11.17703	2.047736	0.152433	0.95882
bta-let-7c	0.540425	9.076337	2.016214	0.155627	0.966004
Novel:NC_037556.1_29844	0.769173	5.824018	2.002176	0.157074	0.966004
Novel:NC_037559.1_34348	1.5416	1.624617	1.979967	0.159394	0.966004
Novel:NC_037548.1_11059	-1.90575	1.429365	1.972739	0.160157	0.966004
bta-miR-877	-0.51527	5.465717	1.971104	0.160331	0.966004
Novel:NC_037553.1_23265	0.849657	3.503649	1.967874	0.160674	0.966004
bta-miR-2285av	-0.84008	2.467302	1.958049	0.161722	0.966004
bta-miR-11988	-1.54068	1.58853	1.944392	0.163192	0.966004
Novel:NC_037553.1_23923	-1.16646	1.805416	1.928258	0.164949	0.966004
bta-miR-665	-0.66315	3.913987	1.925727	0.165227	0.966004
Novel:NC_037545.1_2589	1.222861	2.094629	1.925402	0.165262	0.966004
Novel:NC_037569.1_46468	0.673255	5.69168	1.914882	0.166422	0.966004
bta-miR-320a	-0.46591	13.60957	1.887112	0.169528	0.966004
Novel:NC_037548.1_11079	-0.57792	8.692579	1.883868	0.169895	0.966004
bta-miR-873	-0.85783	5.00683	1.880462	0.170282	0.966004
Novel:NC_037557.1_30688	-1.91052	1.80522	1.878067	0.170554	0.966004
Novel:hsa-miR-1321	1.234824	1.789777	1.873833	0.171037	0.966004
Novel:NC_037547.1_8180	-1.39826	1.739458	1.872413	0.171199	0.966004
Novel:hsa-miR-4632-3p	-1.87208	2.007892	1.869027	0.171587	0.966004
Novel:NC_037547.1_8540	-0.49144	13.4698	1.851792	0.173575	0.969076
Novel:hsa-miR-6868-3p	-1.16599	2.368151	1.847845	0.174034	0.969076
Novel:oar-miR-3957-3p	-1.82564	1.508606	1.791548	0.180738	0.976672
Novel:NC_037569.1_46554	-0.73999	3.32084	1.787351	0.181249	0.976672
bta-miR-205	0.745678	5.195444	1.779671	0.18219	0.976672
Novel:NC_037547.1_7786	1.793186	1.857133	1.773046	0.183006	0.976672
Novel:hsa-miR-4479	1.506919	1.485767	1.76673	0.183787	0.976672
Novel:NC_037545.1_548	-1.30088	2.100576	1.753508	0.185436	0.976672
bta-miR-6119-3p	1.356702	1.622056	1.745521	0.186441	0.976672
Novel:NC_037568.1_44853	-1.41753	1.680585	1.710887	0.19087	0.976672
bta-miR-10a	-0.63867	10.85645	1.69956	0.192346	0.976672
Novel:NC_037552.1_21299	-1.1918	2.39095	1.669244	0.19636	0.976672
Novel:NC_037553.1_24557	-1.37797	1.531951	1.666572	0.196718	0.976672
Novel:hsa-miR-1184	1.747106	1.576863	1.653403	0.198496	0.976672

Novel:NC_037549.1_14266	-1.17655	2.232758	1.649603	0.199013	0.976672
bta-miR-222	0.596109	5.783911	1.623781	0.202565	0.976672
Novel:NC_037546.1_4218	-0.67682	3.276841	1.616965	0.203515	0.976672
bta-miR-423-3p	-0.44497	12.04389	1.608861	0.204652	0.976672
bta-miR-323	-0.93893	2.289652	1.602586	0.205537	0.976672
bta-miR-1224	-0.74643	3.559936	1.60017	0.205879	0.976672
Novel:NC_037552.1_21136	1.201927	1.798985	1.599107	0.20603	0.976672
Novel:NC_037552.1_22028	1.201931	1.798985	1.599088	0.206032	0.976672
bta-miR-145	-0.63028	7.014067	1.596862	0.206349	0.976672
bta-miR-2299-3p	-1.57403	1.483627	1.59592	0.206482	0.976672
bta-miR-450a	-1.1397	2.203543	1.592209	0.207011	0.976672
bta-miR-365-3p	0.787274	3.42108	1.575888	0.209354	0.976672
Novel:NC_037564.1_40367	-0.59231	3.826741	1.55561	0.212309	0.976672
bta-miR-2892	0.553053	5.24332	1.55132	0.212941	0.976672
Novel:NC_037549.1_16477	0.676548	5.946814	1.548328	0.213382	0.976672
Novel:oar-miR-10b	0.630788	3.972301	1.54357	0.214087	0.976672
Novel:NC_037560.1_35631	1.948207	1.708878	1.533028	0.215658	0.976672
bta-miR-1271	0.758788	3.495165	1.522006	0.217316	0.976672
Novel:NC_037566.1_43276	1.706451	1.624031	1.511497	0.218911	0.976672
Novel:hsa-miR-5193	-0.83567	2.323526	1.4953	0.221396	0.976672
bta-miR-449a	1.016926	2.506259	1.479718	0.223819	0.976672
Novel:hsa-miR-1205	0.794092	2.337603	1.468516	0.22558	0.976672
bta-miR-671	0.597819	3.374836	1.459189	0.227059	0.976672
Novel:NC_037565.1_42764	-2.01396	1.450734	1.44575	0.229211	0.976672
Novel:NC_037545.1_583	-1.37074	1.456931	1.439483	0.230223	0.976672
Novel:chi-miR-151-5p	-0.99981	1.874095	1.433002	0.231275	0.976672
Novel:NC_037548.1_12165	-0.66885	2.864987	1.432212	0.231404	0.976672
bta-miR-299	-1.34091	1.364556	1.427881	0.232111	0.976672
bta-miR-2889	-0.55712	6.494151	1.424558	0.232655	0.976672
bta-miR-326	-0.47341	3.360396	1.423991	0.232748	0.976672
Novel:NC_037545.1_169	1.204486	1.924733	1.400464	0.236646	0.976672
bta-miR-12034	-0.45859	5.689224	1.396203	0.23736	0.976672
bta-miR-218	0.731583	2.861732	1.393208	0.237864	0.976672
Novel:hsa-miR-3144-5p	-0.83505	1.744832	1.39105	0.238228	0.976672
Novel:NC_037552.1_22185	-1.08377	2.394221	1.388686	0.238627	0.976672
bta-miR-192	0.429768	11.15164	1.372571	0.241371	0.976672
Novel:hsa-miR-760	-1.62274	1.859276	1.368981	0.241987	0.976672
Novel:NC_037546.1_6618	0.797526	2.519451	1.367762	0.242197	0.976672
bta-miR-210	-0.50827	9.374764	1.364666	0.242731	0.976672
Novel:hsa-miR-6832-3p	-1.06721	1.645269	1.353724	0.244628	0.976672
Novel:hsa-miR-4675	-0.58495	3.428619	1.344819	0.246186	0.976672
bta-miR-452	0.708982	2.877606	1.327424	0.249264	0.976672
bta-miR-16a	0.521217	4.921752	1.322969	0.25006	0.976672
Novel:hsa-miR-3672	-0.4826	10.00113	1.317528	0.251036	0.976672
Novel:NC_037547.1_9455	-0.89348	2.717543	1.316975	0.251136	0.976672
Novel:hsa-miR-4677-3p	1.204746	1.629485	1.310986	0.252216	0.976672
Novel:NC_037549.1_17067	-0.51684	5.627701	1.309764	0.252437	0.976672
Novel:NC_037564.1_40573	-1.85952	1.883414	1.308572	0.252653	0.976672
Novel:hsa-miR-2110	-1.23968	1.942268	1.308163	0.252727	0.976672

bta-miR-126-3p	0.803371	3.734591	1.308069	0.252745	0.976672
Novel:NC_037565.1_42254	-0.61194	3.397948	1.299136	0.254371	0.976672
Novel:NC_037558.1_31842	-0.90254	2.440594	1.299093	0.254379	0.976672
Novel:NC_037558.1_32790	-1.98037	1.412452	1.295601	0.255018	0.976672
Novel:NC_037546.1_6204	-1.13842	1.545389	1.295566	0.255025	0.976672
Novel:hsa-miR-149-3p	1.440601	1.532559	1.288722	0.256284	0.976672
bta-miR-3596	0.341589	10.58281	1.28853	0.256319	0.976672
Novel:NC_037551.1_19577	1.66475	1.378261	1.283471	0.257255	0.976672
Novel:hsa-miR-548v	-0.51389	6.573833	1.274737	0.25888	0.976672
Novel:hsa-miR-7704	1.36482	1.56124	1.264017	0.260892	0.976672
bta-miR-181b	-0.34424	9.663917	1.262942	0.261095	0.976672
Novel:hsa-miR-135a-3p	1.500483	1.453643	1.257609	0.262104	0.976672
Novel:hsa-miR-4689	0.605385	3.666136	1.256051	0.2624	0.976672
bta-miR-378d	-0.62527	2.647191	1.249891	0.263573	0.976672
bta-miR-199c	-0.61711	2.878564	1.247416	0.264047	0.976672
Novel:hsa-miR-339-5p	-0.47884	4.293632	1.246974	0.264131	0.976672
bta-miR-125a	0.593057	7.528002	1.243592	0.26478	0.976672
bta-miR-1388-5p	-0.42724	7.960954	1.222269	0.268916	0.976672
Novel:NC_037547.1_9916	-1.13942	1.405248	1.213132	0.270713	0.976672
bta-miR-196a	1.277938	1.465391	1.212852	0.270768	0.976672
Novel:NC_037547.1_7908	-1.13954	1.405248	1.212776	0.270783	0.976672
bta-miR-185	-0.98771	2.175057	1.210846	0.271165	0.976672
Novel:hsa-miR-663a	-0.52695	9.508964	1.209173	0.271496	0.976672
Novel:NC_037546.1_5554	-0.69663	2.40933	1.207368	0.271854	0.976672
Novel:NC_037553.1_23515	-1.35769	1.519142	1.205735	0.272178	0.976672
Novel:NC_037558.1_32328	1.898448	1.455762	1.19656	0.27401	0.976672
Novel:hsa-miR-21-3p	1.093353	1.926942	1.196198	0.274083	0.976672
Novel:NC_037547.1_6928	2.181332	1.895619	1.195158	0.274292	0.976672
Novel:chi-miR-323b	0.63804	5.51248	1.191732	0.27498	0.976672
Novel:NC_037546.1_4132	-1.07462	1.52907	1.190604	0.275208	0.976672
bta-miR-379	0.986343	2.001905	1.18938	0.275455	0.976672
bta-miR-2285cr	-0.70312	2.234519	1.186271	0.276083	0.976672
Novel:NC_037546.1_4552	0.742513	2.594425	1.175471	0.27828	0.976672
Novel:NC_037568.1_45446	1.289808	1.753954	1.173859	0.27861	0.976672
Novel:chi-miR-127-3p	-0.96565	2.554184	1.165661	0.280295	0.976672
bta-miR-20a	0.583166	4.25312	1.165468	0.280334	0.976672
Novel:NC_037557.1_30641	0.587623	4.272839	1.161378	0.28118	0.976672
bta-let-7i	0.372822	10.80371	1.157965	0.281888	0.976672
bta-miR-30c	0.404102	5.43528	1.153252	0.282869	0.976672
bta-let-7a-3p	0.433098	4.735242	1.139949	0.285663	0.976672
Novel:NC_037551.1_19947	0.721001	2.293281	1.139731	0.285709	0.976672
Novel:NC_037563.1_39275	0.720999	2.293281	1.139455	0.285768	0.976672
Novel:NC_037556.1_30288	0.466606	7.597551	1.135391	0.286628	0.976672
bta-miR-26b	0.326937	7.278941	1.134011	0.286921	0.976672
Novel:hsa-miR-4668-3p	-1.13611	1.59031	1.13373	0.286981	0.976672
Novel:NC_037555.1_26499	1.075024	1.735931	1.13338	0.287055	0.976672
bta-miR-126-5p	0.463039	7.544318	1.12695	0.288427	0.976672
Novel:NC_037550.1_18131	-1.37652	1.535732	1.12169	0.289555	0.976672
Novel:NC_037561.1_36340	-1.21246	1.687369	1.1214	0.289617	0.976672

bta-miR-30f	0.548702	3.20722	1.116969	0.290572	0.976672
bta-miR-10182-5p	1.725751	1.382405	1.111705	0.291712	0.976672
Novel:hsa-miR-3974	1.212421	2.166291	1.105931	0.292968	0.976672
Novel:hsa-miR-1289	-1.36246	1.436172	1.096673	0.294997	0.976672
Novel:NC_037547.1_8396	0.551573	4.998092	1.089748	0.296527	0.976672
Novel:NC_037555.1_27132	1.316401	1.530422	1.075893	0.299618	0.976672
Novel:chi-miR-2331	-0.91234	2.406823	1.071119	0.300693	0.976672
Novel:chi-miR-485-5p	0.849102	2.310919	1.067667	0.301473	0.976672
bta-miR-2285da	-0.80758	1.950808	1.066846	0.301659	0.976672
bta-miR-130b	-0.34543	7.880577	1.065757	0.301906	0.976672
bta-miR-98	0.610239	5.394996	1.062056	0.302747	0.976672
bta-miR-18a	0.807671	2.102516	1.047722	0.306032	0.976672
Novel:NC_037545.1_1965	-1.34605	1.451591	1.032005	0.309688	0.976672
Novel:NC_037557.1_30647	0.436281	4.69884	1.029938	0.310173	0.976672
Novel:hsa-miR-548as-3p	-0.3807	4.397595	1.028095	0.310607	0.976672
Novel:hsa-miR-4482-3p	-0.53283	3.807386	1.025594	0.311196	0.976672
Novel:hsa-miR-146b-3p	-0.72511	2.703041	1.025397	0.311242	0.976672
Novel:hsa-miR-4707-5p	0.879918	1.871987	1.024113	0.311545	0.976672
bta-miR-148b	0.337409	8.057821	1.024062	0.311557	0.976672
bta-miR-2285bl	-0.37128	4.460435	1.022204	0.311997	0.976672
bta-miR-502a	-0.50545	3.339576	1.014448	0.31384	0.976672
Novel:NC_037546.1_2906	-0.95578	2.528889	1.006121	0.315834	0.976672
bta-miR-23a	0.321971	6.474894	1.005586	0.315963	0.976672
Novel:NC_037561.1_37534	0.417585	5.222092	1.004757	0.316162	0.976672
bta-miR-2285ar	-1.2559	1.486034	1.004607	0.316198	0.976672
bta-miR-147	-1.04318	1.93486	1.003895	0.31637	0.976672
Novel:NC_037567.1_44073	-0.33479	9.585328	0.997618	0.317888	0.976672
Novel:NC_037564.1_41171	-0.74514	7.373589	0.991371	0.319408	0.976672
Novel:hsa-miR-1251-3p	-0.46714	9.568711	0.976551	0.323052	0.976672
bta-miR-411c-5p	1.312843	1.696391	0.976392	0.323091	0.976672
Novel:NC_037558.1_33194	1.669752	1.414204	0.972098	0.324158	0.976672
bta-miR-12023	-0.84622	1.824676	0.971088	0.324409	0.976672
bta-miR-2887	-0.41589	3.500298	0.965715	0.325751	0.976672
bta-miR-3956	-1.28821	1.311098	0.965442	0.32582	0.976672
bta-miR-323b-3p	-0.55367	2.708988	0.956281	0.328126	0.976672
bta-miR-1247-5p	0.550588	3.754145	0.954802	0.3285	0.976672
bta-miR-181d	-0.46777	4.564889	0.95337	0.328863	0.976672
bta-miR-2387	-1.38515	1.465111	0.947941	0.330244	0.976672
Novel:NC_037548.1_11028	-1.50892	1.775917	0.943705	0.331327	0.976672
bta-miR-410	-0.33117	7.524986	0.943572	0.331361	0.976672
Novel:NC_037549.1_14209	-0.95761	1.646391	0.93348	0.33396	0.976672
bta-miR-2904	0.493574	5.059082	0.932578	0.334194	0.976672
bta-miR-17-5p	0.412178	4.708666	0.930352	0.334771	0.976672
Novel:NC_037557.1_31285	-1.87565	1.527766	0.929554	0.334979	0.976672
Novel:hsa-miR-6808-3p	0.902395	1.6333	0.923356	0.336595	0.976672
Novel:NC_037555.1_27844	0.578452	5.226546	0.919651	0.337567	0.976672
bta-miR-378c	-0.40384	6.777032	0.919079	0.337717	0.976672
Novel:NC_037569.1_46177	-0.79739	1.728043	0.913045	0.339308	0.976672
bta-miR-2403	0.571994	2.612657	0.911663	0.339674	0.976672

Novel:NC_037567.1_43945	-1.31199	1.70955	0.909823	0.340161	0.976672
Novel:hsa-miR-6784-3p	0.891439	2.179059	0.908471	0.34052	0.976672
Novel:hsa-miR-1324	1.383801	1.417649	0.904378	0.34161	0.976672
bta-miR-122	0.966246	1.88632	0.903718	0.341787	0.976672
Novel:NC_037545.1_2499	-0.53845	11.53777	0.902714	0.342055	0.976672
Novel:hsa-miR-1199-5p	0.947403	1.513909	0.891391	0.345101	0.976672
Novel:NC_037555.1_26952	1.307562	1.304736	0.889363	0.34565	0.976672
Novel:hsa-miR-619-3p	-1.27328	1.54481	0.88796	0.346031	0.976672
bta-miR-1343-5p	-0.73944	2.127707	0.887343	0.346198	0.976672
bta-miR-34c	-0.82771	2.538358	0.886617	0.346396	0.976672
Novel:NC_037555.1_28194	0.936379	1.55331	0.880802	0.347982	0.976672
bta-miR-103	0.404629	13.67231	0.878135	0.348713	0.976672
bta-miR-2285bz	0.350918	5.55262	0.878122	0.348716	0.976672
Novel:NC_037561.1_36931	-0.36898	7.131084	0.872071	0.350382	0.976672
bta-miR-2285co	-0.28721	6.949227	0.871502	0.35054	0.976672
Novel:NC_037550.1_18908	0.330173	9.506429	0.869494	0.351095	0.976672
Novel:NC_037558.1_32528	1.053232	1.771418	0.869224	0.35117	0.976672
Novel:hsa-miR-6846-5p	-1.03611	1.52248	0.863892	0.352652	0.976672
Novel:hsa-miR-6787-3p	1.317502	1.422798	0.862313	0.353092	0.976672
Novel:hsa-miR-4767	-1.21322	1.563607	0.86224	0.353113	0.976672
bta-miR-6517	-0.2876	5.919003	0.858237	0.354232	0.976672
Novel:NC_037561.1_37123	-1.64169	1.592508	0.855871	0.354897	0.976672
bta-miR-107	0.405464	12.66822	0.855769	0.354926	0.976672
bta-miR-424-5p	-0.40517	5.982899	0.855069	0.355122	0.976672
bta-miR-2285aa	-0.60178	2.742698	0.853169	0.355657	0.976672
Novel:hsa-miR-3150b-3p	-0.86119	2.428386	0.840136	0.359358	0.976672
Novel:hsa-miR-6885-3p	-1.3682	1.525777	0.832833	0.361455	0.976672
Novel:chi-miR-500-5p	-0.41624	3.041855	0.831376	0.361875	0.976672
Novel:NC_037545.1_698	1.091747	1.678895	0.827566	0.362977	0.976672
Novel:NC_037569.1_48122	1.188614	1.586578	0.826666	0.363238	0.976672
Novel:chi-miR-378-3p	-0.56757	2.613185	0.821193	0.364832	0.976672
Novel:NC_037554.1_24998	0.684247	3.27619	0.81628	0.36627	0.976672
Novel:NC_037552.1_21762	1.180401	1.421997	0.81466	0.366746	0.976672
Novel:oar-miR-299-3p	1.457373	1.522333	0.814567	0.366773	0.976672
Novel:NC_037566.1_43159	1.561881	1.471188	0.811817	0.367584	0.976672
Novel:NC_037566.1_43651	1.561949	1.471188	0.811424	0.367699	0.976672
bta-miR-130a	-0.41449	12.86231	0.809114	0.368382	0.976672
bta-miR-184	-0.86836	1.578389	0.807063	0.36899	0.976672
bta-miR-1343-3p	0.362068	5.049214	0.805095	0.369574	0.976672
Novel:NC_037553.1_24219	0.700793	1.86876	0.803899	0.36993	0.976672
bta-miR-378	-0.44241	13.21861	0.803648	0.370005	0.976672
Novel:NC_037557.1_31699	0.468097	6.42525	0.803213	0.370134	0.976672
Novel:hsa-miR-6834-5p	-0.51185	2.968189	0.799555	0.371227	0.976672
Novel:hsa-miR-3074-5p	-0.30065	9.864352	0.798622	0.371506	0.976672
bta-miR-330	-0.77198	2.503794	0.797582	0.371817	0.976672
Novel:hsa-miR-1237-3p	-0.4406	13.25207	0.79479	0.372656	0.976672
bta-miR-142-5p	0.559429	3.489653	0.794146	0.372849	0.976672
Novel:chi-miR-320-3p	-0.78692	1.674774	0.784684	0.375712	0.976672
bta-miR-193a-5p	-0.41951	4.018662	0.780209	0.377077	0.976672

bta-miR-186	0.313749	9.529399	0.778045	0.37774	0.976672
Novel:hsa-miR-3689d	0.405672	10.87231	0.775445	0.378538	0.976672
bta-miR-378b	-0.43977	6.583714	0.77533	0.378573	0.976672
bta-miR-2299-5p	-0.44994	3.619966	0.775268	0.378592	0.976672
Novel:hsa-miR-6089	-0.51597	2.861319	0.765261	0.381687	0.980771
bta-miR-2890	-0.39198	5.200276	0.762504	0.382546	0.980771
bta-miR-2285b	0.812888	2.397775	0.759768	0.383401	0.980771
Novel:NC_037562.1_38773	-0.28604	12.54985	0.755727	0.384669	0.980771
Novel:NC_037565.1_42130	1.214179	1.814534	0.754696	0.384993	0.980771
Novel:NC_037553.1_23200	-0.42326	3.48997	0.744775	0.388135	0.983256
Novel:hsa-miR-1304-3p	-1.2588	1.494868	0.744554	0.388206	0.983256
Novel:NC_037545.1_1635	-0.42025	3.025389	0.741744	0.389103	0.983256
bta-miR-11977	-0.32211	4.863178	0.738878	0.390021	0.983256
Novel:NC_037566.1_42967	-0.55966	2.707009	0.736471	0.390794	0.983256
bta-miR-11972	-0.57924	3.112008	0.729341	0.393097	0.986343
bta-miR-2440	-0.69659	5.352067	0.725765	0.39426	0.986343
bta-let-7a-5p	0.37079	10.11142	0.723726	0.394924	0.986343
bta-miR-2285k	-0.56505	1.767522	0.713112	0.398413	0.990313
bta-let-7b	0.264215	10.04148	0.709966	0.399455	0.990313
bta-miR-140	-0.27718	12.4505	0.708678	0.399883	0.990313
Novel:NC_037569.1_47087	-0.36532	6.103518	0.7069	0.400475	0.990313
Novel:NC_037554.1_25895	-1.60374	1.493615	0.699592	0.402921	0.990313
Novel:hsa-miR-1199-3p	-0.4045	5.053842	0.698077	0.403431	0.990313
Novel:hsa-miR-5580-5p	-0.37895	5.036506	0.697265	0.403704	0.990313
Novel:NC_037564.1_40338	0.847184	1.751197	0.695534	0.404289	0.990313
bta-miR-2285bn	-0.34621	3.107124	0.682484	0.408733	0.992728
Novel:hsa-miR-3173-3p	1.063295	2.045173	0.68232	0.408789	0.992728
Novel:NC_037547.1_10878	1.126807	1.637208	0.678862	0.409979	0.992728
Novel:NC_037558.1_32847	-0.41461	6.121398	0.67509	0.411282	0.992728
bta-miR-12031	1.078749	1.526793	0.675047	0.411298	0.992728
Novel:oar-miR-134-3p	-0.85596	1.919393	0.664909	0.414832	0.992728
Novel:hsa-miR-219a-2-3p	-0.67578	3.400134	0.662312	0.415745	0.992728
Novel:NC_037552.1_21749	-0.9121	2.024414	0.661926	0.415881	0.992728
bta-miR-500	1.107795	1.438251	0.661829	0.415915	0.992728
bta-miR-2285v	-0.60399	1.992424	0.656286	0.417874	0.992728
Novel:NC_037567.1_44728	0.481553	3.079429	0.654238	0.418602	0.992728
bta-miR-664b	-0.84872	1.878909	0.649698	0.42022	0.992728
bta-miR-2285cj	-0.45649	3.42785	0.649359	0.420342	0.992728
bta-miR-143	0.355649	14.11462	0.648421	0.420678	0.992728
Novel:NC_037569.1_46552	-0.33466	6.853613	0.647365	0.421056	0.992728
Novel:NC_037560.1_35426	0.520831	9.595805	0.64317	0.422565	0.992728
Novel:chi-miR-125a-3p	0.599945	1.505286	0.639622	0.423848	0.992728
bta-miR-2284p	-0.79638	1.523461	0.633316	0.426141	0.992728
Novel:hsa-miR-149-5p	-0.42982	2.182076	0.628716	0.427827	0.992728
Novel:NC_037555.1_27478	0.962248	1.330722	0.627208	0.428381	0.992728
Novel:NC_037552.1_21285	-0.81381	1.499561	0.623119	0.429891	0.992728
bta-miR-4286	-1.11377	1.545896	0.62022	0.430966	0.992728
Novel:NC_037555.1_26458	0.950851	1.329218	0.617873	0.431839	0.992728
bta-miR-411c-3p	-1.28606	1.877809	0.614527	0.433089	0.992728

Novel:NC_037565.1_42675	-0.69516	2.540366	0.614381	0.433143	0.992728
Novel:hsa-miR-4743-5p	-0.42247	3.820097	0.612693	0.433776	0.992728
Novel:NC_037545.1_498	-0.31813	8.495374	0.60938	0.435021	0.992728
bta-miR-574	-0.25747	10.50746	0.609186	0.435094	0.992728
Novel:hsa-miR-448	1.089571	1.37282	0.608411	0.435387	0.992728
bta-miR-708	0.762719	3.901433	0.607225	0.435834	0.992728
bta-miR-155	0.293222	8.200515	0.606644	0.436054	0.992728
Novel:NC_037564.1_40415	0.715835	2.112371	0.605599	0.43645	0.992728
Novel:NC_037550.1_19148	-1.18213	1.467929	0.599588	0.438735	0.993003
Novel:NC_037553.1_22994	0.33505	13.70476	0.59732	0.439602	0.993003
Novel:NC_037546.1_3761	-0.76724	2.322872	0.59424	0.440784	0.993003
Novel:hsa-miR-486-3p	0.876257	1.482927	0.592237	0.441556	0.993003
Novel:NC_037569.1_48203	0.75323	1.869625	0.591225	0.441946	0.993003
bta-miR-12006	0.670277	1.903428	0.590004	0.442418	0.993003
Novel:NC_037558.1_32782	0.301307	8.688377	0.5855	0.444165	0.993057
Novel:NC_037551.1_19485	-0.48561	2.248364	0.581613	0.445681	0.993057
Novel:NC_037546.1_4816	0.765271	1.857943	0.578992	0.446708	0.993057
Novel:NC_037558.1_32781	0.299238	8.689296	0.577362	0.447348	0.993057
Novel:NC_037558.1_33041	0.832342	1.515548	0.576721	0.4476	0.993057
Novel:NC_037547.1_8774	1.142422	1.566359	0.5744	0.448516	0.993057
Novel:NC_037567.1_44300	0.451592	3.111543	0.57251	0.449263	0.993057
bta-miR-494	0.453164	2.752564	0.568877	0.450706	0.994089
Novel:NC_037553.1_22953	-0.37784	3.055144	0.564686	0.452378	0.99496
Novel:NC_037564.1_40358	-0.27515	6.201466	0.562552	0.453234	0.99496
bta-miR-380-3p	0.311643	4.729518	0.560572	0.45403	0.99496
Novel:NC_037560.1_35002	-0.71631	1.657889	0.556741	0.455576	0.996207
bta-miR-362-5p	0.997535	1.399262	0.541397	0.461854	0.998817
bta-miR-365-5p	-0.88256	1.403876	0.528285	0.467329	0.998817
bta-miR-24-3p	-0.23864	9.972446	0.527899	0.467492	0.998817
bta-miR-2355-3p	-0.93267	1.459753	0.527066	0.467843	0.998817
bta-miR-29c	-0.36776	5.998407	0.526112	0.468246	0.998817
bta-miR-296-5p	-0.75863	1.658473	0.52377	0.469238	0.998817
bta-miR-425-3p	-0.25578	4.501255	0.523226	0.469469	0.998817
Novel:hsa-miR-185-5p	-1.12581	1.672244	0.522965	0.46958	0.998817
Novel:hsa-miR-4663	0.775186	1.727191	0.510532	0.474908	0.998817
Novel:hsa-miR-4706	-0.73441	1.530384	0.510121	0.475086	0.998817
Novel:NC_037561.1_36447	-1.21816	1.515638	0.507748	0.476115	0.998817
Novel:hsa-miR-1252-5p	0.615243	1.760208	0.503217	0.47809	0.998817
bta-miR-27a-3p	-0.2013	8.169508	0.500993	0.479064	0.998817
Novel:hsa-miR-5739	1.038895	1.429923	0.494525	0.481916	0.998817
Novel:NC_037552.1_21735	1.068502	1.33481	0.49212	0.482983	0.998817
Novel:hsa-miR-943	-0.56034	2.051101	0.490569	0.483673	0.998817
bta-miR-335	-0.56478	4.903698	0.484623	0.486336	0.998817
bta-miR-17-3p	-0.71443	1.739361	0.484088	0.486576	0.998817
bta-miR-219	0.703704	2.036447	0.482631	0.487233	0.998817
Novel:hsa-miR-4753-5p	0.414482	2.552757	0.481218	0.487871	0.998817
bta-miR-455-5p	-0.31332	3.983553	0.480807	0.488057	0.998817
bta-miR-23b-3p	0.25712	4.90017	0.480378	0.488251	0.998817
Novel:hsa-miR-4308	0.320763	4.667962	0.478845	0.488946	0.998817

bta-miR-301a	0.318868	6.198477	0.475473	0.49048	0.998817
Novel:NC_037549.1_15915	-0.90923	1.640019	0.47535	0.490536	0.998817
Novel:hsa-miR-5195-5p	0.260874	8.262356	0.474225	0.49105	0.998817
Novel:NC_037552.1_22398	-0.55485	4.884735	0.472827	0.49169	0.998817
Novel:NC_037552.1_21699	0.893321	1.373136	0.472593	0.491797	0.998817
Novel:hsa-miR-6814-5p	0.524957	3.228237	0.471801	0.49216	0.998817
bta-miR-6520	-0.37316	4.508228	0.459508	0.497854	0.998817
Novel:NC_037548.1_12436	-1.06478	1.590218	0.454299	0.500301	0.998817
Novel:hsa-miR-4800-3p	-0.53878	5.303957	0.452681	0.501064	0.998817
Novel:NC_037555.1_27170	-0.66881	1.775264	0.45082	0.501946	0.998817
bta-miR-6528	-0.97487	1.845175	0.449399	0.50262	0.998817
bta-miR-767	-0.68228	1.853739	0.447757	0.503402	0.998817
Novel:hsa-miR-4800-5p	-0.22254	7.320013	0.447198	0.503668	0.998817
Novel:hsa-miR-4723-5p	0.265545	13.2115	0.443322	0.505523	0.998817
bta-miR-2285aj-5p	-0.24084	8.991113	0.443205	0.505579	0.998817
Novel:hsa-miR-7106-5p	-0.8518	1.523403	0.440826	0.506724	0.998817
bta-miR-541	-0.37098	3.758513	0.440475	0.506893	0.998817
bta-miR-425-5p	0.2367	5.765868	0.440196	0.507028	0.998817
Novel:hsa-miR-3064-5p	0.659412	1.520314	0.439184	0.507517	0.998817
bta-miR-493	0.472981	2.676936	0.43665	0.508744	0.998817
Novel:hsa-miR-3919	0.429927	2.589714	0.432695	0.51067	0.998817
bta-miR-16b	0.267201	6.938761	0.431592	0.511209	0.998817
Novel:hsa-miR-12117	0.914457	1.487252	0.428404	0.512773	0.998817
bta-miR-193b	-0.3363	10.34341	0.428038	0.512953	0.998817
Novel:NC_037546.1_4112	-0.26609	2.995341	0.425495	0.514208	0.998817
Novel:NC_037547.1_7372	-0.26614	2.995341	0.425419	0.514245	0.998817
bta-miR-101	0.32678	9.744422	0.423178	0.515356	0.998817
bta-miR-30d	0.29925	10.29538	0.419463	0.517205	0.998817
Novel:hsa-miR-7160-3p	-1.25613	1.43014	0.417982	0.517946	0.998817
Novel:NC_037560.1_35881	0.433247	1.858417	0.417744	0.518065	0.998817
Novel:hsa-miR-6828-3p	-1.17798	1.575023	0.417635	0.518119	0.998817
Novel:hsa-miR-4746-5p	-0.32469	3.9175	0.416274	0.518802	0.998817
Novel:NC_037549.1_16300	0.451481	11.2702	0.415618	0.519132	0.998817
Novel:NC_037564.1_41300	0.601783	1.906931	0.412667	0.520619	0.998817
Novel:NC_037547.1_9757	0.595723	8.363249	0.409747	0.522098	0.998817
Novel:NC_037568.1_46017	-0.31178	2.592558	0.409611	0.522167	0.998817
Novel:NC_037560.1_35659	-0.3118	2.592558	0.409474	0.522237	0.998817
Novel:hsa-miR-5190	-0.51025	5.329473	0.409286	0.522332	0.998817
Novel:hsa-miR-6849-3p	0.574795	3.33296	0.40759	0.523195	0.998817
bta-miR-592	0.509239	2.188874	0.405684	0.524168	0.998817
Novel:NC_037550.1_17420	-0.4399	3.150406	0.404416	0.524818	0.998817
Novel:hsa-miR-5587-3p	-0.67164	1.655402	0.399393	0.527403	0.998817
bta-miR-542-5p	-0.36188	3.395832	0.3941	0.530152	0.998817
bta-miR-214	0.217209	4.730785	0.390591	0.531989	0.998817
bta-miR-424-3p	-0.23425	5.126908	0.390507	0.532033	0.998817
Novel:NC_037551.1_20226	0.783291	1.864643	0.390364	0.532108	0.998817
Novel:NC_037560.1_35933	-0.49734	7.556271	0.388345	0.533171	0.998817
bta-miR-15b	0.30273	3.079038	0.387299	0.533723	0.998817
Novel:chi-miR-326-3p	-0.56917	1.706565	0.380324	0.53743	0.998817

bta-miR-33b	0.5388	2.056404	0.379553	0.537842	0.998817
Novel:hsa-miR-3185	-0.49037	7.497936	0.378893	0.538196	0.998817
Novel:NC_037564.1_40897	0.668805	1.570094	0.377484	0.538953	0.998817
Novel:NC_037549.1_16572	-0.5714	1.902996	0.377261	0.539073	0.998817
Novel:NC_037547.1_9951	0.284787	6.207324	0.376553	0.539454	0.998817
Novel:hsa-miR-3654	0.640413	2.860725	0.362958	0.546868	0.998817
Novel:NC_037565.1_42322	0.375023	2.988204	0.361805	0.547505	0.998817
Novel:NC_037545.1_137	0.47909	1.838993	0.360622	0.548161	0.998817
bta-miR-2411-5p	0.916939	1.310194	0.359949	0.548535	0.998817
Novel:hsa-miR-3122	-0.5711	1.360694	0.358238	0.549487	0.998817
bta-miR-15a	0.202247	6.633103	0.356918	0.550223	0.998817
Novel:hsa-miR-3620-5p	-0.54118	1.701954	0.355998	0.550737	0.998817
Novel:NC_037564.1_41137	0.53693	2.058176	0.349679	0.554295	0.998817
Novel:NC_037548.1_12213	0.400991	1.621743	0.344853	0.557041	0.998817
Novel:NC_037568.1_45248	-0.31144	9.940385	0.342416	0.558438	0.998817
bta-miR-2285cs	-0.29264	2.068689	0.341323	0.559067	0.998817
bta-miR-191b	0.382307	3.038962	0.340359	0.559622	0.998817
bta-miR-2483-5p	-0.43322	2.745863	0.339692	0.560007	0.998817
Novel:hsa-miR-4491	0.468844	1.916223	0.339233	0.560272	0.998817
Novel:NC_037547.1_8021	-0.85308	1.362173	0.336384	0.561924	0.998817
bta-miR-2285ad	-0.52651	1.608069	0.33385	0.563401	0.998817
bta-miR-532	-0.17951	9.20238	0.333709	0.563483	0.998817
Novel:NC_037546.1_3187	0.85153	1.373278	0.332262	0.56433	0.998817
Novel:NC_037569.1_47783	-0.57318	1.979033	0.332199	0.564367	0.998817
Novel:hsa-miR-9900	1.016679	1.527884	0.331985	0.564493	0.998817
Novel:NC_037569.1_46559	-0.17928	9.273797	0.330179	0.565553	0.998817
Novel:hsa-miR-6071	-0.5606	1.493592	0.329667	0.565855	0.998817
bta-miR-2331-3p	0.476715	1.637624	0.328277	0.566675	0.998817
Novel:NC_037550.1_19123	-0.52687	1.431011	0.327726	0.567001	0.998817
bta-miR-193a-3p	-0.26939	4.884875	0.324976	0.568633	0.998817
Novel:NC_037552.1_21563	-0.54471	2.410744	0.320594	0.571251	0.998817
Novel:chi-miR-1307-3p	-0.24125	6.597135	0.318588	0.572457	0.998817
Novel:NC_037552.1_22436	-0.54093	2.43248	0.317047	0.573388	0.998817
bta-miR-30a-5p	-0.17363	12.86627	0.313875	0.575312	0.998817
Novel:NC_037547.1_8898	-0.61813	1.498267	0.311666	0.57666	0.998817
bta-miR-2284z	0.516442	2.509713	0.311518	0.57675	0.998817
Novel:NC_037564.1_40393	-0.94951	1.666909	0.309784	0.577813	0.998817
bta-miR-3601	-0.31537	3.472468	0.308011	0.578903	0.998817
Novel:hsa-miR-208a-5p	0.438901	3.159706	0.307949	0.578942	0.998817
Novel:NC_037553.1_23471	0.73626	1.63917	0.307643	0.57913	0.998817
bta-miR-431	-0.72529	1.460247	0.304849	0.580858	0.998817
Novel:NC_037564.1_40946	-0.65102	1.605134	0.304537	0.581052	0.998817
Novel:NC_037553.1_24410	0.480474	1.725979	0.304083	0.581334	0.998817
Novel:NC_037564.1_40695	0.587429	1.360469	0.301897	0.582696	0.998817
Novel:hsa-miR-6776-5p	0.67712	1.595192	0.300614	0.583498	0.998817
Novel:NC_037552.1_21636	-0.68033	1.682432	0.300404	0.583629	0.998817
bta-miR-2285cm	-0.42279	2.098342	0.300172	0.583775	0.998817
Novel:NC_037545.1_434	-0.3745	3.754237	0.299069	0.584467	0.998817
bta-miR-127	-0.17836	10.30025	0.29619	0.586281	0.998817

bta-miR-432	0.229802	5.258919	0.295488	0.586725	0.998817
Novel:NC_037567.1_44673	-0.80638	1.516327	0.292644	0.588531	0.998817
Novel:NC_037560.1_35403	0.492717	5.685683	0.288991	0.590868	0.998817
Novel:hsa-miR-203a-3p	-0.31809	3.369797	0.288656	0.591083	0.998817
bta-miR-3578	-0.3279	3.171708	0.287814	0.591625	0.998817
bta-miR-449c	0.634035	1.360299	0.28752	0.591814	0.998817
Novel:NC_037546.1_3986	-0.86495	1.757626	0.285708	0.592984	0.998817
Novel:NC_037554.1_25983	-0.40567	1.755317	0.285545	0.59309	0.998817
bta-miR-2285dd	-0.37893	1.798983	0.28531	0.593242	0.998817
bta-miR-382	-0.3257	3.172409	0.285183	0.593324	0.998817
bta-miR-1306	-0.37281	2.540497	0.284002	0.59409	0.998817
bta-miR-2285aw	-0.48801	1.613761	0.282712	0.594929	0.998817
bta-miR-4449	-0.76106	1.574999	0.281385	0.595795	0.998817
Novel:hsa-miR-6888-5p	-0.72675	1.692053	0.281239	0.59589	0.998817
bta-miR-2408	-0.40327	1.932868	0.276708	0.598867	0.998817
Novel:chi-miR-345-5p	0.571676	1.637085	0.276112	0.599261	0.998817
Novel:hsa-miR-572	-0.69926	1.649054	0.270794	0.602799	0.998817
bta-miR-497	-0.18012	6.964418	0.270411	0.603057	0.998817
bta-let-7f	0.204928	10.52247	0.268943	0.604041	0.998817
bta-miR-2435	-0.20001	5.503499	0.268443	0.604378	0.998817
bta-miR-345-5p	-0.80063	1.534622	0.264511	0.607038	0.998817
Novel:hsa-miR-372-5p	0.414764	3.034069	0.263889	0.607461	0.998817
Novel:NC_037555.1_26610	-0.50666	1.905966	0.261052	0.609398	0.998817
Novel:NC_037557.1_30640	-0.26213	9.614681	0.260128	0.610032	0.998817
Novel:NC_037549.1_14540	-0.65287	2.10191	0.256914	0.612248	0.998817
bta-miR-1291	0.441617	1.503812	0.255127	0.613488	0.998817
Novel:NC_037550.1_17628	-0.16318	7.79162	0.255081	0.61352	0.998817
bta-miR-2899	0.408072	2.213476	0.253528	0.614602	0.998817
Novel:NC_037551.1_19470	0.632425	1.892972	0.251731	0.615859	0.998817
bta-miR-503-5p	0.586915	1.483686	0.250818	0.6165	0.998817
Novel:hsa-miR-744-5p	0.555519	1.690764	0.249969	0.617097	0.998817
Novel:NC_037549.1_16296	0.369049	2.234232	0.245791	0.620055	0.998817
bta-miR-2484	-0.60957	1.669829	0.242332	0.622527	0.998817
Novel:NC_037546.1_2774	0.542135	1.495197	0.23974	0.624394	0.998817
bta-miR-6535	-0.53105	1.482741	0.237409	0.626083	0.998817
bta-miR-133a	0.302943	3.202732	0.237169	0.626258	0.998817
bta-miR-10174-3p	0.160158	5.64984	0.236803	0.626525	0.998817
bta-miR-199b	-0.35308	2.841184	0.23352	0.628926	0.998817
Novel:NC_037569.1_47131	0.329777	2.645673	0.23233	0.629802	0.998817
bta-miR-190b	-0.20856	3.306474	0.229981	0.631538	0.998817
Novel:hsa-miR-136-3p	0.374418	2.184721	0.229585	0.631832	0.998817
bta-miR-215	0.385807	10.94262	0.229056	0.632224	0.998817
Novel:NC_037547.1_9101	0.248387	4.467413	0.227245	0.633574	0.998817
bta-miR-760-3p	-0.1529	7.630499	0.225858	0.634612	0.998817
Novel:NC_037547.1_9353	-0.26294	5.188939	0.22501	0.635249	0.998817
bta-miR-25	-0.13876	14.57389	0.22419	0.635866	0.998817
Novel:hsa-miR-6715a-3p	-0.16742	7.036325	0.223808	0.636154	0.998817
Novel:hsa-miR-5002-5p	-0.30909	2.277625	0.223289	0.636545	0.998817
Novel:hsa-miR-10392-3p	-0.37927	2.474042	0.220233	0.638863	0.998817

bta-miR-19b	-0.22638	9.825117	0.220207	0.638883	0.998817
bta-miR-374a	-0.32591	2.564467	0.219549	0.639384	0.998817
bta-miR-154c	-0.20708	3.96167	0.219502	0.63942	0.998817
bta-miR-6518	-0.17933	6.307946	0.217203	0.641179	0.998817
bta-miR-411a	0.28209	5.320233	0.21639	0.641804	0.998817
Novel:NC_037564.1_40345	0.278566	5.408855	0.216041	0.642073	0.998817
bta-miR-1307	-0.16259	6.143943	0.215734	0.64231	0.998817
bta-miR-11987	-0.23612	5.25986	0.21513	0.642776	0.998817
bta-miR-92b	0.175275	12.03648	0.215127	0.642778	0.998817
bta-miR-2284f	0.417098	1.650494	0.211997	0.645206	0.998817
Novel:NC_037549.1_15080	-0.34588	5.471856	0.210708	0.646213	0.998817
Novel:NC_037566.1_43137	0.234197	6.23957	0.210651	0.646258	0.998817
bta-miR-106b	0.172629	5.118988	0.209842	0.646892	0.998817
bta-miR-21-5p	0.165767	11.53566	0.209161	0.647426	0.998817
Novel:hsa-miR-4691-3p	0.215573	4.086311	0.203469	0.651935	0.998817
Novel:NC_037564.1_40376	-0.15044	7.460968	0.201105	0.65383	0.998817
Novel:NC_037564.1_40236	-0.26313	3.447547	0.200623	0.654218	0.998817
Novel:NC_037555.1_28111	-0.17666	3.555113	0.200232	0.654534	0.998817
bta-miR-2285x	0.501048	1.589517	0.200006	0.654716	0.998817
Novel:NC_037568.1_45672	-0.5999	1.555651	0.198479	0.655951	0.998817
bta-miR-885	0.530108	1.530899	0.197033	0.657127	0.998817
bta-miR-199a-3p	0.176456	7.30592	0.196174	0.657827	0.998817
bta-miR-2285j	0.477126	1.60735	0.193808	0.659766	0.998817
bta-miR-188	-0.19518	4.12906	0.193149	0.660308	0.998817
bta-miR-331-5p	-0.16381	5.274609	0.192122	0.661157	0.998817
Novel:NC_037545.1_1494	0.295519	2.445654	0.186434	0.665901	0.998817
Novel:NC_037545.1_431	-0.14452	8.010662	0.186289	0.666023	0.998817
Novel:NC_037547.1_8573	-0.14452	8.010662	0.186101	0.666182	0.998817
Novel:hsa-miR-3169	0.650245	1.420129	0.182087	0.669586	0.998817
Novel:NC_037553.1_23622	-0.38745	1.677589	0.17839	0.67276	0.998817
bta-miR-224	0.278002	2.625437	0.175687	0.675106	0.998817
Novel:NC_037558.1_32026	-0.18755	10.02452	0.175566	0.675212	0.998817
bta-miR-2446	-0.33715	2.288119	0.172819	0.677618	0.998817
Novel:NC_037553.1_23783	-0.15241	5.851896	0.167751	0.682118	0.998817
Novel:hsa-miR-6125	0.216248	4.523354	0.167382	0.682449	0.998817
bta-miR-10164-3p	0.218808	4.528462	0.166393	0.683338	0.998817
Novel:hsa-miR-3529-3p	-0.48425	1.36425	0.16626	0.683457	0.998817
bta-miR-3432a	0.586464	1.414465	0.165726	0.683939	0.998817
Novel:NC_037555.1_27093	-0.38991	1.708315	0.165499	0.684144	0.998817
Novel:hsa-miR-4514	-0.23926	3.76391	0.163505	0.68595	0.998817
Novel:NC_037551.1_19861	0.323094	2.994439	0.160489	0.688706	0.998817
Novel:NC_037554.1_25273	-0.42579	1.955214	0.15941	0.6897	0.998817
Novel:chi-miR-221-5p	-0.40862	2.105577	0.15863	0.690421	0.998817
bta-miR-677	0.24369	5.194664	0.154665	0.694117	0.998817
bta-miR-301b	0.342978	1.885057	0.154284	0.694475	0.998817
Novel:NC_037545.1_558	-0.24215	3.186758	0.154276	0.694482	0.998817
bta-miR-22-3p	0.139206	16.18844	0.153977	0.694763	0.998817
Novel:NC_037569.1_46557	-0.17237	4.21276	0.15331	0.695392	0.998817
Novel:hsa-miR-4687-5p	-0.24818	2.31325	0.152134	0.696504	0.998817

bta-miR-202	0.168754	9.479483	0.152033	0.6966	0.998817
Novel:NC_037562.1_38088	-0.53514	1.473733	0.150595	0.697967	0.998817
Novel:NC_037547.1_7667	-0.41614	1.709995	0.149515	0.698999	0.998817
bta-miR-3604	0.154043	7.958153	0.149339	0.699167	0.998817
Novel:hsa-miR-6867-5p	0.149363	4.867186	0.147805	0.700642	0.998817
bta-miR-208a	-0.4338	1.358376	0.147673	0.700769	0.998817
bta-miR-2285f	0.15389	3.46608	0.147491	0.700945	0.998817
bta-miR-3600	0.13599	16.21493	0.147135	0.701289	0.998817
Novel:hsa-miR-6124	-0.26096	3.804961	0.146016	0.702372	0.998817
Novel:NC_037545.1_503	-0.18682	8.718988	0.14538	0.702991	0.998817
bta-miR-181a	-0.105	12.30764	0.145137	0.703227	0.998817
Novel:NC_037566.1_43625	0.196616	6.255769	0.14486	0.703497	0.998817
Novel:hsa-miR-6771-5p	-0.19943	5.299602	0.14447	0.703877	0.998817
Novel:hsa-miR-4475	-0.28035	3.895328	0.142921	0.705394	0.998817
Novel:hsa-miR-4778-3p	-0.39886	1.479398	0.140604	0.707682	0.998817
Novel:NC_037556.1_30106	-0.10673	11.30704	0.139547	0.708732	0.998817
Novel:NC_037549.1_15660	-0.10673	11.30704	0.13951	0.708769	0.998817
bta-miR-502b	0.143025	3.607237	0.139144	0.709133	0.998817
Novel:hsa-miR-12135	-0.18639	2.71407	0.137402	0.710877	0.998817
bta-miR-2285db	0.471267	1.444275	0.136719	0.711565	0.998817
bta-miR-345-3p	-0.12438	7.321573	0.135466	0.71283	0.998817
Novel:NC_037545.1_1554	-0.19039	3.523403	0.133857	0.714466	0.998817
bta-miR-92a	-0.14567	14.06716	0.133341	0.714993	0.998817
Novel:hsa-miR-3960	0.31287	2.49191	0.132724	0.715624	0.998817
Novel:chi-miR-9-5p	0.409199	1.786942	0.132694	0.715655	0.998817
Novel:NC_037553.1_24444	-0.58369	1.674185	0.132194	0.716168	0.998817
bta-miR-10b	-0.11488	18.76372	0.131227	0.717163	0.998817
Novel:NC_037546.1_5469	0.386663	1.624576	0.129829	0.718609	0.998817
Novel:hsa-miR-4276	-0.32924	2.186046	0.129706	0.718737	0.998817
bta-let-7g	-0.13731	8.52757	0.127516	0.721021	0.998817
Novel:NC_037546.1_3818	-0.37377	1.630775	0.127177	0.721377	0.998817
bta-miR-2285q	-0.16318	3.320026	0.12629	0.72231	0.998817
bta-miR-125b	0.195304	7.162828	0.12621	0.722395	0.998817
Novel:chi-miR-24-3p	-0.29545	3.005225	0.125982	0.722635	0.998817
Novel:NC_037548.1_12259	0.143179	5.22213	0.125548	0.723094	0.998817
Novel:NC_037564.1_41155	0.15536	4.869911	0.125541	0.723101	0.998817
Novel:hsa-miR-6755-5p	-0.28505	2.003525	0.124825	0.723859	0.998817
Novel:NC_037552.1_21521	-0.6329	1.75045	0.123526	0.725241	0.998817
Novel:NC_037564.1_40877	-0.32236	2.183696	0.122742	0.72608	0.998817
Novel:hsa-miR-2355-5p	-0.40935	1.502895	0.121665	0.727236	0.998817
Novel:hsa-miR-591	0.298882	8.616183	0.120875	0.728087	0.998817
Novel:NC_037558.1_32366	-0.61398	1.51628	0.120486	0.728507	0.998817
Novel:NC_037558.1_33220	-0.61409	1.51628	0.120382	0.728621	0.998817
Novel:NC_037558.1_31888	-0.42681	1.853161	0.117996	0.731218	0.998817
Novel:hsa-miR-185-3p	0.205813	3.531884	0.117344	0.731933	0.998817
Novel:hsa-miR-1827	0.404402	1.475856	0.116983	0.73233	0.998817
Novel:NC_037549.1_15054	-0.33968	1.839462	0.116533	0.732825	0.998817
Novel:NC_037548.1_13020	-0.4797	1.451724	0.115634	0.733819	0.998817
bta-miR-11971	-0.15771	4.580028	0.114759	0.73479	0.998817

bta-miR-2285dh	-0.13236	3.480206	0.113206	0.736524	0.998817
bta-miR-199a-5p	-0.18061	4.49218	0.1125	0.737315	0.998817
Novel:NC_037548.1_13083	0.120759	5.441075	0.11129	0.73868	0.998817
Novel:hsa-miR-3917	0.470249	1.634354	0.110317	0.739784	0.998817
Novel:hsa-miR-4687-3p	0.723078	1.529685	0.109821	0.740348	0.998817
bta-miR-1296	0.131948	5.233318	0.10778	0.742686	0.998817
bta-miR-328	0.17843	4.65803	0.107694	0.742785	0.998817
Novel:NC_037549.1_15917	0.155847	4.600526	0.105989	0.744758	0.998817
Novel:NC_037547.1_7586	0.395188	1.631563	0.104694	0.746268	0.998817
Novel:hsa-miR-6499-5p	0.152481	4.048527	0.103929	0.747165	0.998817
bta-miR-2285ae	-0.2692	1.538271	0.103183	0.748042	0.998817
bta-miR-99b	-0.13217	12.01016	0.102982	0.74828	0.998817
bta-miR-148a	-0.09853	13.32264	0.102495	0.748856	0.998817
Novel:NC_037560.1_35884	-0.1306	9.685204	0.098165	0.754043	0.998817
bta-miR-12057	-0.24416	1.953441	0.098116	0.754103	0.998817
Novel:hsa-miR-4695-5p	0.350883	1.507279	0.09715	0.755277	0.998817
bta-miR-339a	0.100104	7.623771	0.09568	0.757077	0.998817
Novel:hsa-miR-3157-5p	0.229343	2.545641	0.094408	0.758647	0.998817
Novel:hsa-miR-6780b-3p	0.326613	1.54759	0.093663	0.759571	0.998817
Novel:chi-miR-3432-5p	0.249438	2.912656	0.093265	0.760067	0.998817
Novel:NC_037545.1_824	0.132664	9.345255	0.091517	0.762258	0.998817
Novel:hsa-miR-3126-5p	-0.47622	1.355205	0.091199	0.762659	0.998817
Novel:hsa-miR-3670	0.177945	2.489146	0.090747	0.76323	0.998817
bta-miR-331-3p	-0.20061	2.269242	0.089761	0.764481	0.998817
Novel:NC_037557.1_30644	-0.15836	6.768314	0.089621	0.76466	0.998817
bta-miR-369-3p	-0.17113	2.91701	0.08936	0.764992	0.998817
Novel:NC_037546.1_4856	0.272276	1.686451	0.088639	0.765915	0.998817
bta-miR-11986b	0.252612	1.674508	0.08862	0.765939	0.998817
bta-miR-204	0.154731	5.475664	0.088418	0.766197	0.998817
bta-miR-132	0.258487	9.123071	0.086871	0.768193	0.998817
Novel:NC_037556.1_30278	-0.38035	1.777807	0.086854	0.768216	0.998817
Novel:NC_037565.1_42672	-0.11219	8.590043	0.086492	0.768686	0.998817
bta-miR-296-3p	-0.12448	10.25381	0.086334	0.768891	0.998817
Novel:NC_037561.1_36929	0.273654	1.841088	0.08602	0.7693	0.998817
Novel:hsa-miR-152-5p	-0.50236	1.399497	0.085834	0.769542	0.998817
Novel:hsa-miR-4450	0.365127	1.680496	0.082763	0.773588	0.998817
bta-miR-2285p	0.347302	1.390561	0.081466	0.775321	0.998817
Novel:NC_037548.1_13397	-0.1028	4.584731	0.081434	0.775363	0.998817
Novel:hsa-miR-10522-5p	-0.51284	1.71963	0.080849	0.77615	0.998817
bta-miR-21-3p	0.10339	10.42971	0.080031	0.777255	0.998817
Novel:NC_037562.1_38928	-0.46432	1.689302	0.079474	0.778012	0.998817
Novel:NC_037556.1_28514	-0.42357	1.392367	0.077648	0.780511	0.998817
bta-miR-6119-5p	0.177221	5.031294	0.077512	0.780697	0.998817
Novel:hsa-miR-5589-5p	-0.13598	5.028429	0.077289	0.781005	0.998817
bta-miR-2285z	-0.13079	3.367674	0.077215	0.781107	0.998817
bta-miR-100	-0.11414	9.697923	0.07594	0.782876	0.998817
bta-miR-551b	0.14353	2.480417	0.07518	0.783939	0.998817
Novel:NC_037569.1_46466	-0.11353	9.785854	0.074653	0.784678	0.998817
Novel:NC_037547.1_10635	0.083206	13.59168	0.07296	0.787075	0.998817

Novel:hsa-miR-4501	0.451925	1.845173	0.072899	0.787162	0.998817
Novel:hsa-miR-5088-5p	-0.43942	1.520404	0.072718	0.787419	0.998817
Novel:NC_037560.1_35724	-0.18278	2.151776	0.071878	0.788621	0.998817
bta-miR-221	-0.10997	9.820457	0.070845	0.790111	0.998817
bta-miR-129-3p	0.337967	1.741165	0.068847	0.793023	0.998817
Novel:NC_037561.1_36221	-0.16871	2.102648	0.064559	0.79943	0.998817
bta-miR-129	-0.16137	6.649794	0.064197	0.799981	0.998817
bta-miR-129-5p	-0.16137	6.649794	0.064179	0.800009	0.998817
Novel:hsa-miR-198	-0.16989	4.184701	0.063874	0.800475	0.998817
Novel:hsa-miR-4537	0.215697	2.469144	0.063828	0.800545	0.998817
Novel:hsa-miR-3908	0.156321	6.87256	0.06376	0.800649	0.998817
Novel:chi-miR-499-3p	0.223428	1.881167	0.063484	0.801071	0.998817
bta-miR-1246	-0.15674	10.87025	0.06284	0.802063	0.998817
Novel:NC_037549.1_16794	0.31185	1.757786	0.062419	0.802712	0.998817
Novel:NC_037561.1_37162	-0.12566	2.82648	0.062192	0.803064	0.998817
Novel:chi-miR-103-5p	0.216999	1.65585	0.061944	0.80345	0.998817
bta-miR-197	0.11864	5.136002	0.061601	0.803983	0.998817
bta-miR-484	0.082631	7.625436	0.061085	0.80479	0.998817
Novel:hsa-miR-4795-5p	0.418941	1.508214	0.05892	0.808211	0.998817
Novel:NC_037563.1_39679	-0.10538	6.306133	0.058914	0.808221	0.998817
Novel:NC_037560.1_34765	-0.15842	5.735837	0.057765	0.810063	0.998817
Novel:NC_037552.1_22370	-0.15842	5.735837	0.057701	0.810167	0.998817
Novel:hsa-miR-4447	-0.17346	3.368278	0.057628	0.810285	0.998817
Novel:NC_037549.1_14328	-0.23263	1.717787	0.057624	0.810292	0.998817
Novel:NC_037564.1_40975	0.323686	1.51436	0.057023	0.811264	0.998817
bta-miR-12030	0.140164	3.543152	0.056569	0.812004	0.998817
bta-miR-148d	-0.13341	10.46831	0.056215	0.812581	0.998817
Novel:NC_037564.1_40643	-0.16687	2.011339	0.053762	0.816642	0.998817
Novel:chi-miR-411b-5p	-0.11245	3.219401	0.053436	0.817189	0.998817
bta-miR-2284x	-0.07915	10.54715	0.052292	0.819123	0.998817
bta-miR-27a-5p	-0.27861	1.719241	0.052071	0.819497	0.998817
Novel:NC_037569.1_47107	0.152008	5.037839	0.05203	0.819567	0.998817
bta-miR-128	-0.07387	8.760547	0.051917	0.819761	0.998817
bta-miR-2330-5p	-0.17571	1.351832	0.050807	0.821664	0.998817
Novel:NC_037547.1_8809	0.191988	1.719731	0.050439	0.822302	0.998817
Novel:NC_037557.1_30978	-0.12858	2.003143	0.050125	0.822846	0.998817
bta-miR-99a-3p	-0.13602	4.649453	0.049265	0.824347	0.998817
bta-miR-22-5p	0.092257	5.235736	0.048938	0.824922	0.998817
bta-miR-7863	-0.22066	1.777785	0.048521	0.825657	0.998817
bta-miR-342	0.078851	7.141012	0.048482	0.825727	0.998817
Novel:NC_037565.1_41728	0.284911	1.474657	0.047512	0.827451	0.998817
Novel:NC_037557.1_31142	-0.0908	5.884537	0.047135	0.828125	0.998817
Novel:NC_037564.1_41452	-0.09079	5.884537	0.047097	0.828194	0.998817
Novel:NC_037569.1_46961	-0.09079	5.884537	0.047061	0.828258	0.998817
Novel:hsa-miR-765	-0.27021	1.627709	0.046477	0.829311	0.998817
Novel:chi-miR-22-3p	0.123131	2.358156	0.04607	0.830048	0.998817
Novel:chi-miR-432-5p	0.304294	1.346002	0.045523	0.831045	0.998817
Novel:hsa-miR-4720-5p	0.169505	4.672684	0.045352	0.831357	0.998817
Novel:hsa-miR-762	0.303881	1.346002	0.045313	0.831429	0.998817

Novel:hsa-miR-629-3p	-0.296	1.786452	0.044267	0.833357	0.998817
bta-miR-29a	-0.08197	9.713153	0.043914	0.834013	0.998817
Novel:hsa-miR-4659a-3p	0.301296	2.198401	0.041245	0.839067	0.998817
Novel:hsa-miR-3683	0.337552	1.635336	0.039741	0.841988	0.998817
bta-miR-2285br	0.147218	2.327732	0.039276	0.842902	0.998817
bta-miR-99a-5p	-0.09371	8.969573	0.039236	0.842981	0.998817
bta-miR-2378	0.21104	1.477345	0.038452	0.844539	0.998817
Novel:NC_037564.1_40336	-0.17803	1.496511	0.037896	0.845653	0.998817
Novel:NC_037564.1_40729	-0.06893	8.722099	0.037801	0.845844	0.998817
bta-miR-2285u	-0.06132	7.132351	0.037791	0.845863	0.998817
bta-miR-27b	0.055009	13.93009	0.037247	0.846963	0.998817
Novel:hsa-miR-7113-3p	-0.13456	10.9965	0.037202	0.847053	0.998817
Novel:NC_037558.1_32071	-0.124	4.776886	0.036519	0.848448	0.998817
Novel:chi-miR-103-3p	0.095691	3.581319	0.036081	0.849347	0.998817
Novel:NC_037569.1_47935	0.081578	4.807561	0.035846	0.849835	0.998817
bta-miR-219b-3p	-0.19524	1.792298	0.034878	0.851852	0.998817
bta-miR-30b-5p	-0.08065	4.686871	0.034481	0.852687	0.998817
Novel:hsa-miR-4721	-0.17045	1.553775	0.033929	0.853858	0.998817
bta-miR-2284c	-0.2024	1.755566	0.033505	0.854763	0.998817
bta-miR-744	0.05469	7.104264	0.033122	0.855587	0.998817
Novel:hsa-miR-1301-3p	0.344467	1.682307	0.032073	0.857868	0.998817
bta-miR-196b	-0.15924	1.902012	0.031772	0.858528	0.998817
Novel:hsa-miR-4443	-0.18772	1.325245	0.031396	0.859361	0.998817
bta-miR-32	0.052005	6.431804	0.030632	0.861063	0.998817
Novel:NC_037549.1_16548	-0.10747	2.23514	0.030615	0.861101	0.998817
bta-miR-151-3p	0.064292	11.36817	0.030375	0.861642	0.998817
bta-miR-370	-0.16691	2.279613	0.029796	0.862954	0.998817
Novel:hsa-miR-548c-3p	0.125562	1.850714	0.029672	0.863236	0.998817
Novel:NC_037567.1_44693	0.066171	5.895763	0.029426	0.863799	0.998817
bta-miR-2285ak-5p	0.158579	1.542306	0.029201	0.864316	0.998817
Novel:NC_037551.1_20645	-0.16827	1.704587	0.029019	0.864736	0.998817
bta-miR-146b	-0.07803	4.29228	0.028704	0.865463	0.998817
bta-miR-423-5p	0.050051	14.18471	0.02783	0.867508	0.998817
Novel:NC_037569.1_47135	-0.17143	1.967749	0.027818	0.867536	0.998817
bta-miR-181c	0.068754	4.209579	0.027627	0.867988	0.998817
bta-miR-2415-3p	-0.15019	1.812914	0.027374	0.868588	0.998817
Novel:hsa-miR-2115-5p	-0.0528	13.02771	0.027319	0.868721	0.998817
Novel:NC_037546.1_4370	-0.09602	10.33433	0.026923	0.869666	0.998817
Novel:NC_037550.1_17666	0.071977	4.813186	0.02599	0.871925	0.998817
bta-miR-34a	-0.08465	2.886944	0.025336	0.873533	0.998817
bta-miR-2285cv	-0.16265	1.675488	0.024909	0.874593	0.998817
Novel:NC_037564.1_40744	-0.06499	5.981801	0.02451	0.875594	0.998817
Novel:NC_037557.1_30428	-0.06498	5.981801	0.024489	0.875647	0.998817
Novel:NC_037546.1_6567	0.259963	1.737585	0.024029	0.876811	0.998817
bta-miR-2478	0.120959	2.617066	0.02367	0.877727	0.998817
Novel:hsa-miR-6748-3p	-0.12284	2.251738	0.023644	0.877795	0.998817
Novel:chi-miR-493-3p	-0.19455	1.670329	0.023458	0.878272	0.998817
Novel:hsa-miR-4515	0.076668	6.793849	0.023288	0.87871	0.998817
bta-miR-450b	0.059568	7.935033	0.023026	0.879389	0.998817

bta-miR-93	0.044528	9.005703	0.023026	0.87939	0.998817
Novel:NC_037548.1_11085	-0.37827	1.378513	0.022504	0.880753	0.998817
Novel:NC_037569.1_47983	-0.06182	5.983254	0.02212	0.881768	0.998817
Novel:hsa-miR-10399-3p	-0.19488	1.947679	0.022027	0.882015	0.998817
Novel:NC_037559.1_34015	-0.1538	1.891263	0.021208	0.884212	0.998817
Novel:NC_037567.1_44092	-0.0848	10.1975	0.020873	0.885126	0.998817
bta-miR-195	-0.05555	4.766101	0.020663	0.8857	0.998817
bta-miR-152	-0.05594	8.352606	0.020638	0.885768	0.998817
bta-miR-505	0.061989	4.820603	0.020609	0.88585	0.998817
bta-miR-409a	0.224645	1.833665	0.020573	0.885947	0.998817
Novel:NC_037559.1_33968	-0.05247	10.31769	0.020473	0.886225	0.998817
bta-miR-2285dk	-0.24881	1.492365	0.020187	0.887017	0.998817
Novel:NC_037569.1_47143	0.132798	2.148578	0.019699	0.88838	0.998817
Novel:NC_037546.1_6394	-0.13942	1.445648	0.018913	0.890617	0.998817
bta-miR-1839	-0.04657	8.844883	0.018582	0.891571	0.998817
Novel:hsa-miR-6741-5p	-0.04924	6.731156	0.018508	0.891787	0.998817
bta-miR-3431	-0.06172	3.321907	0.018281	0.892449	0.998817
Novel:NC_037553.1_24045	0.10204	1.437736	0.017477	0.894825	0.998817
bta-miR-9851	0.116491	2.036511	0.017071	0.896048	0.998817
bta-miR-153	0.11978	1.917494	0.016908	0.896542	0.998817
bta-miR-146a	0.139093	2.104411	0.016652	0.897325	0.998817
Novel:NC_037560.1_34839	0.09447	2.233743	0.016147	0.898885	0.998817
bta-miR-669	-0.05082	11.02623	0.01601	0.899311	0.998817
bta-miR-2285ba	0.071855	3.285701	0.015999	0.899347	0.998817
Novel:NC_037564.1_40307	-0.06187	4.092234	0.015797	0.899981	0.998817
Novel:NC_037568.1_44905	0.036333	8.995136	0.015329	0.901465	0.998817
Novel:NC_037549.1_15024	-0.07615	2.313063	0.014908	0.90282	0.998817
Novel:NC_037549.1_15077	-0.0424	7.969521	0.014786	0.903217	0.998817
bta-miR-2284j	-0.08013	2.080774	0.014358	0.90462	0.998817
bta-miR-3120	0.143644	1.819413	0.014317	0.904756	0.998817
bta-miR-149-5p	-0.04941	4.632354	0.01419	0.905179	0.998817
Novel:hsa-miR-6839-3p	-0.08815	9.860327	0.014146	0.905324	0.998817
Novel:chi-miR-671-5p	-0.06845	2.252709	0.01409	0.905512	0.998817
bta-miR-660	0.052861	11.78519	0.013866	0.906263	0.998817
bta-miR-545-5p	0.094826	2.177937	0.013574	0.90725	0.998817
Novel:NC_037546.1_5605	-0.13297	1.544269	0.01356	0.907297	0.998817
bta-miR-503-3p	-0.07841	2.648943	0.013384	0.9079	0.998817
bta-miR-1388-3p	-0.04854	6.043896	0.012629	0.910523	0.998817
Novel:NC_037545.1_1192	0.042885	3.237798	0.012493	0.911005	0.998817
Novel:hsa-miR-8077	-0.04128	5.023806	0.012309	0.911661	0.998817
Novel:NC_037569.1_46549	0.047953	11.78555	0.011372	0.915073	0.998817
Novel:NC_037547.1_8236	-0.14038	1.860861	0.011226	0.915618	0.998817
bta-miR-30e-5p	0.030232	11.10622	0.010926	0.91675	0.998817
Novel:hsa-miR-3202	-0.19779	1.450629	0.010808	0.9172	0.998817
Novel:NC_037556.1_29371	0.099898	1.94634	0.010782	0.917299	0.998817
Novel:hsa-miR-6131	-0.0343	6.620803	0.010647	0.917816	0.998817
Novel:NC_037547.1_10096	0.107883	2.083482	0.01033	0.919045	0.998817
Novel:NC_037567.1_44260	0.038832	5.969771	0.010256	0.919334	0.998817
Novel:NC_037547.1_7477	0.136127	1.3978	0.010017	0.920277	0.998817

bta-miR-495	0.10333	1.589364	0.009784	0.921208	0.998817
Novel:NC_037550.1_19237	-0.18779	1.546192	0.009642	0.921778	0.998817
bta-miR-2338	0.133748	1.276766	0.00887	0.924967	0.998817
bta-miR-29b	0.073788	2.684435	0.008786	0.925319	0.998817
Novel:NC_037547.1_7559	0.027835	7.073511	0.008551	0.926323	0.998817
Novel:NC_037568.1_44803	0.047472	4.836278	0.008139	0.928117	0.998817
bta-miR-2285o	-0.07551	1.90037	0.007883	0.92925	0.998817
Novel:chi-miR-491-5p	-0.03744	3.060353	0.007174	0.932501	0.998817
bta-miR-769	0.028953	6.587312	0.007047	0.933101	0.998817
Novel:NC_037558.1_33240	-0.1288	1.380179	0.006676	0.934879	0.998817
Novel:hsa-miR-3179	-0.17305	1.548625	0.006659	0.934962	0.998817
Novel:NC_037556.1_30323	0.077424	2.336564	0.006653	0.934994	0.998817
Novel:NC_037546.1_3432	-0.06268	2.067479	0.00653	0.935594	0.998817
Novel:hsa-miR-5681a	0.143801	1.517702	0.005687	0.939886	0.998817
bta-miR-2284m	0.082777	1.825244	0.005638	0.940148	0.998817
Novel:hsa-miR-3153	-0.0746	2.209302	0.005469	0.941047	0.998817
bta-miR-361	-0.03724	8.063139	0.005433	0.941241	0.998817
Novel:NC_037553.1_22911	-0.03393	4.415704	0.005381	0.941523	0.998817
Novel:hsa-miR-759	-0.03894	2.663097	0.005294	0.941996	0.998817
bta-miR-2285e	-0.03142	3.830827	0.005216	0.942426	0.998817
bta-miR-874	0.027908	4.532811	0.005205	0.942483	0.998817
Novel:hsa-miR-635	-0.06599	1.6938	0.005155	0.942763	0.998817
Novel:hsa-miR-3921	-0.08878	1.705055	0.005091	0.94312	0.998817
Novel:NC_037547.1_9224	0.060367	1.942277	0.00497	0.943797	0.998817
Novel:NC_037546.1_2960	0.049807	3.057899	0.004937	0.943983	0.998817
bta-miR-654	0.091234	1.759398	0.004813	0.944689	0.998817
bta-miR-2389	-0.04967	2.505953	0.004709	0.945292	0.998817
Novel:hsa-miR-12136	-0.02513	4.495823	0.004643	0.945677	0.998817
bta-miR-212	0.115957	1.7456	0.00452	0.946399	0.998817
Novel:NC_037563.1_39396	-0.03314	14.43255	0.004252	0.948008	0.998817
Novel:NC_037551.1_20840	0.072146	1.649013	0.004133	0.94874	0.998817
Novel:hsa-miR-4467	-0.1003	1.629531	0.004063	0.949179	0.998817
bta-miR-421	0.024081	5.588156	0.003709	0.951438	0.998817
Novel:NC_037550.1_18493	-0.01648	1.451215	0.003635	0.951926	0.998817
Novel:NC_037550.1_17382	-0.01637	1.451215	0.003619	0.952027	0.998817
bta-miR-182	0.029794	3.617287	0.00342	0.953364	0.998817
bta-miR-339b	0.018324	7.371061	0.003149	0.955246	0.998817
bta-miR-2285y	0.037167	2.441697	0.002931	0.956822	0.998817
Novel:NC_037563.1_39878	-0.028	14.40007	0.002899	0.957063	0.998817
Novel:chi-miR-125a-5p	0.020953	5.345684	0.002742	0.958236	0.998817
Novel:NC_037546.1_4402	0.051354	2.035642	0.002674	0.958759	0.998817
bta-miR-148c	-0.02078	8.602825	0.002569	0.959579	0.998817
Novel:NC_037550.1_17830	-0.03842	1.779703	0.002098	0.963465	0.998817
Novel:NC_037550.1_18986	-0.03916	1.779636	0.002097	0.963473	0.998817
bta-miR-6529a	0.014631	9.333944	0.002075	0.963669	0.998817
bta-miR-136	0.039975	2.320077	0.002014	0.964203	0.998817
Novel:hsa-miR-1915-3p	-0.04943	1.864617	0.001942	0.964852	0.998817
bta-miR-2284aa	0.041185	1.853689	0.001812	0.966042	0.998817
Novel:NC_037564.1_41423	-0.02695	2.483915	0.001795	0.966209	0.998817

Novel:NC_037569.1_48167	-0.03478	2.167634	0.001727	0.966852	0.998817
Novel:NC_037556.1_29299	0.040709	2.303306	0.001688	0.967224	0.998817
Novel:NC_037549.1_14335	0.058071	1.420958	0.001638	0.967716	0.998817
bta-miR-139	-0.03319	1.726243	0.00154	0.968694	0.998817
Novel:NC_037563.1_40142	0.061795	1.388247	0.001448	0.969642	0.998817
Novel:NC_037561.1_36492	-0.01957	2.860607	0.001441	0.969722	0.998817
Novel:chi-miR-34a	-0.06653	1.483574	0.001361	0.970576	0.998817
bta-miR-29d-3p	-0.02506	2.254234	0.001267	0.971602	0.998817
Novel:hsa-miR-4277	-0.02197	1.901118	0.00118	0.972601	0.998817
Novel:NC_037568.1_45118	-0.03167	2.226539	0.001104	0.973498	0.998817
Novel:NC_037548.1_12850	-0.02804	8.775802	0.001082	0.973759	0.998817
bta-miR-194	0.032784	5.913773	0.001031	0.974382	0.998817
bta-miR-376b	0.072586	1.309163	0.001013	0.97461	0.998817
Novel:NC_037569.1_46653	-0.02646	2.14586	0.000828	0.977047	0.998817
Novel:NC_037550.1_18976	-0.00559	1.468312	0.000687	0.979083	0.998817
Novel:NC_037569.1_46992	0.009213	5.774578	0.000636	0.979876	0.998817
Novel:hsa-miR-6890-3p	-0.05423	1.483615	0.000634	0.979916	0.998817
bta-miR-6123	0.009772	4.660909	0.000492	0.982306	0.998817
Novel:NC_037546.1_5830	0.008139	4.535472	0.000478	0.982559	0.998817
Novel:hsa-miR-4683	0.058479	1.473109	0.000443	0.983207	0.998817
bta-miR-2411-3p	-0.02481	1.308628	0.000443	0.983217	0.998817
Novel:hsa-miR-4442	-0.02818	1.521038	0.000435	0.983361	0.998817
Novel:NC_037558.1_32412	0.007962	6.131854	0.000352	0.985022	0.998817
bta-miR-2483-3p	0.005763	5.931274	0.000289	0.986443	0.998817
Novel:NC_037561.1_36813	0.010371	1.666611	0.000264	0.98703	0.998817
Novel:hsa-miR-4524a-3p	0.021281	1.720879	0.000208	0.988492	0.998817
bta-miR-504	-0.00665	2.660128	0.000208	0.988505	0.998817
bta-miR-19a	0.005387	7.207161	0.000169	0.989628	0.998817
Novel:chi-miR-874-5p	-0.05009	1.440163	0.000129	0.990937	0.998817
Novel:hsa-miR-548a-3p	0.004897	5.957002	0.000126	0.99103	0.998817
bta-miR-499	-0.00468	1.80783	0.000101	0.991966	0.998817
Novel:NC_037551.1_19765	-0.00373	4.529654	9.02E-05	0.992422	0.998817
bta-miR-455-3p	-0.00809	1.800891	8.67E-05	0.99257	0.998817
Novel:hsa-miR-4468	-0.02236	1.597158	7.64E-05	0.993028	0.998817
bta-miR-2284ab	-0.00267	4.911518	5.38E-05	0.994146	0.998817
Novel:NC_037558.1_32697	-0.00565	1.583999	3.28E-05	0.995433	0.998817
bta-miR-6529b	0.001582	9.410117	2.43E-05	0.996064	0.998817
bta-miR-151-5p	-0.00132	6.909447	1.55E-05	0.996856	0.998817
Novel:chi-miR-202-3p	0.00075	11.14771	5.48E-06	0.998132	0.999113
bta-miR-2340	0.006551	1.451863	1.15E-08	0.999915	0.999915

**An(NBS_vs_BS)**	**logFC**	**logCPM**	**LR**	**PValue**	**FDR**
Novel:chi-miR-24-5p	1.759425	6.711139	23.44098	1.29E-06	0.001112
Novel:hsa-miR-4783-5p	-2.28792	3.501613	18.9941	1.31E-05	0.005658
Novel:NC_037560.1_35933	2.292563	4.039258	17.03357	3.67E-05	0.010564
bta-miR-2285bf	-5.71169	1.652003	16.15636	5.83E-05	0.012227
bta-let-7e	1.995393	5.955978	15.58025	7.91E-05	0.012227
Novel:hsa-miR-3185	2.222827	4.010231	15.44359	8.50E-05	0.012227
Novel:NC_037560.1_35256	-4.97147	1.207092	14.31441	0.000155	0.019069
Novel:hsa-miR-3689d	1.49203	10.32475	13.63644	0.000222	0.023931

bta-miR-669	-1.28623	10.21008	13.18732	0.000282	0.027026
Novel:hsa-miR-766-5p	-3.32247	1.556366	12.61439	0.000383	0.033035
Novel:NC_037556.1_29844	2.205352	5.336525	12.35582	0.00044	0.03449
Novel:NC_037547.1_8396	1.78202	4.378567	12.0799	0.00051	0.036655
bta-miR-193a	-1.61912	3.932286	11.52013	0.000688	0.045234
Novel:hsa-miR-6741-5p	-1.04309	6.174605	11.40162	0.000734	0.045234
Novel:NC_037555.1_27452	-2.58873	1.908699	10.33286	0.001307	0.075186
Novel:hsa-miR-4475	2.53521	2.303457	10.13226	0.001457	0.077034
Novel:hsa-miR-4723-5p	1.030215	12.98765	9.984584	0.001579	0.077034
Novel:hsa-miR-663a	1.175565	8.487989	9.952013	0.001607	0.077034
Novel:hsa-miR-6777-5p	-1.39699	10.13302	9.72372	0.001819	0.081306
bta-let-7f	1.13799	10.55746	9.599893	0.001946	0.081306
Novel:NC_037565.1_42179	-2.51953	2.804396	9.569388	0.001978	0.081306
Novel:hsa-miR-6727-5p	1.913788	10.9649	9.390945	0.002181	0.08248
bta-miR-328	-1.45884	3.95284	9.376206	0.002198	0.08248
bta-miR-143	0.903771	14.39301	9.232497	0.002378	0.085494
Novel:hsa-miR-8057	-0.88677	8.267578	8.986602	0.00272	0.093148
bta-miR-222	1.184545	5.936563	8.911306	0.002834	0.093148
Novel:NC_037569.1_46468	1.214861	5.882272	8.860398	0.002914	0.093148
Novel:NC_037558.1_32842	-1.17185	4.16919	8.632925	0.003301	0.101754
bta-miR-486	1.753488	11.65926	8.490039	0.003571	0.106267
Novel:NC_037553.1_22994	0.905403	13.93755	8.384404	0.003785	0.108869
Novel:hsa-miR-7108-3p	-0.9374	11.6862	8.228338	0.004124	0.110057
bta-let-7c	0.984191	9.254602	8.189267	0.004214	0.110057
bta-miR-126-5p	1.176349	7.532982	8.134649	0.004343	0.110057
Novel:hsa-miR-6765-3p	1.610863	2.673964	8.122395	0.004372	0.110057
bta-miR-342	-0.86297	6.386327	8.084922	0.004464	0.110057
Novel:hsa-miR-4689	1.369995	3.051422	7.959321	0.004784	0.114684
Novel:NC_037556.1_28345	-2.70199	1.287191	7.832129	0.005133	0.115259
Novel:NC_037556.1_28346	-2.70191	1.287191	7.826109	0.00515	0.115259
Novel:NC_037556.1_30288	1.168811	7.578888	7.805521	0.005209	0.115259
bta-miR-193b	-1.03859	8.591068	7.713372	0.005481	0.11826
bta-miR-197	-1.22951	4.194645	7.510676	0.006133	0.127913
Novel:NC_037568.1_45248	-1.04166	8.124223	7.476651	0.00625	0.127913
Novel:chi-miR-24-3p	2.471498	1.757207	7.441574	0.006373	0.127913
Novel:hsa-miR-6124	1.435616	3.345789	7.17492	0.007393	0.145003
Novel:NC_037569.1_47154	-1.00312	7.270513	7.079958	0.007795	0.148305
bta-miR-3596	0.827828	10.31932	7.054862	0.007905	0.148305
Novel:hsa-miR-6125	-1.2448	3.768948	6.991864	0.008188	0.1486
Novel:hsa-miR-6747-3p	1.68022	2.407509	6.975107	0.008265	0.1486
Novel:hsa-miR-1252-3p	1.53865	2.427918	6.856765	0.008831	0.155527
Novel:NC_037550.1_18158	-0.99902	8.212392	6.662491	0.009846	0.169947
Novel:hsa-miR-3074-5p	-0.88216	9.671895	6.523689	0.010645	0.178232
Novel:NC_037548.1_11583	-3.10693	1.449028	6.507943	0.010739	0.178232
Novel:hsa-miR-4520-3p	-1.15529	5.943527	6.369562	0.011609	0.189037
bta-miR-6123	-0.91565	3.842098	6.29024	0.01214	0.193337
Novel:hsa-miR-6068	-0.9615	7.126713	6.263996	0.012322	0.193337
bta-miR-16b	0.861051	6.493388	6.219257	0.012637	0.194742
Novel:NC_037553.1_24733	-3.17785	1.204447	6.137113	0.013237	0.200418

bta-miR-11977	-0.86472	4.731658	6.068843	0.013759	0.20472
Novel:hsa-miR-3672	-1.21442	9.621211	6.029231	0.014071	0.205816
Novel:NC_037561.1_37534	0.900889	5.15976	5.971048	0.014543	0.209172
bta-miR-155	0.77246	7.320686	5.919202	0.014977	0.211703
Novel:oar-miR-10b	1.042825	4.156257	5.892062	0.015209	0.211703
bta-let-7a-5p	1.097118	10.25244	5.863221	0.01546	0.211784
bta-miR-2285af	-3.9332	0.769043	5.731659	0.016662	0.222852
bta-miR-433	3.447732	0.964294	5.718728	0.016785	0.222852
bta-miR-12034	-0.85921	5.652566	5.690091	0.017061	0.223085
Novel:NC_037546.1_4277	-2.64852	1.612247	5.629136	0.017664	0.226255
Novel:hsa-miR-5195-5p	0.754857	7.365178	5.596085	0.018001	0.226255
bta-miR-11986c	-1.10546	2.944947	5.58742	0.01809	0.226255
Novel:NC_037565.1_42254	-1.38436	3.203189	5.504926	0.018963	0.233786
bta-miR-451	1.848703	3.481833	5.394385	0.020202	0.24555
bta-miR-24-3p	-0.74411	9.798492	5.20058	0.022579	0.270639
Novel:NC_037552.1_21563	-1.61619	2.200701	5.128212	0.02354	0.277812
Novel:hsa-miR-4675	-1.31828	3.122229	5.107572	0.023822	0.277812
bta-let-7b	0.702311	9.529969	4.953674	0.026035	0.299023
Novel:NC_037567.1_44073	-0.71437	9.450056	4.932363	0.026358	0.299023
Novel:NC_037565.1_42436	-2.36667	1.316672	4.911405	0.02668	0.299023
Novel:NC_037555.1_27844	0.798276	5.62992	4.865585	0.027398	0.301591
Novel:NC_037552.1_22436	-1.56063	2.237052	4.848369	0.027672	0.301591
Novel:NC_037565.1_42672	0.772167	7.981502	4.829408	0.027978	0.301591
bta-miR-10a	-0.85255	11.19243	4.809279	0.028307	0.301591
Novel:hsa-miR-545-3p	0.879856	5.417928	4.734275	0.029567	0.306086
bta-miR-205	-0.98131	3.70056	4.724657	0.029733	0.306086
Novel:hsa-miR-146b-3p	-1.38236	2.56263	4.721205	0.029793	0.306086
bta-miR-1468	0.871323	5.414486	4.646039	0.031125	0.316013
bta-let-7g	0.761659	7.89541	4.594886	0.032067	0.321793
bta-miR-219	-2.82537	1.048769	4.561981	0.032689	0.322578
Novel:NC_037551.1_20226	3.361195	0.887508	4.523825	0.033426	0.322578
Novel:NC_037551.1_19470	3.361471	0.887508	4.517628	0.033547	0.322578
bta-miR-138	-2.59871	1.179031	4.512865	0.033641	0.322578
Novel:hsa-miR-3960	-1.50573	1.974231	4.416647	0.03559	0.337517
bta-miR-2446	1.709652	1.415494	4.3098	0.037893	0.353023
Novel:NC_037557.1_31699	0.910479	5.177282	4.303096	0.038043	0.353023
Novel:NC_037565.1_42322	1.522552	2.43851	4.257179	0.039085	0.358831
Novel:hsa-miR-3909	-0.85431	3.551733	4.213437	0.040105	0.362406
bta-miR-126-3p	1.139496	4.028097	4.20163	0.040385	0.362406
bta-miR-3600	-0.51382	15.13297	4.187049	0.040734	0.362406
bta-miR-22-3p	-0.51221	15.10745	4.106298	0.042724	0.376231
bta-miR-423-3p	-0.5336	12.18732	4.079339	0.043411	0.378029
Novel:hsa-miR-5580-5p	-0.83674	5.013974	4.064097	0.043804	0.378029
bta-let-7d	0.929387	4.204218	3.983319	0.045953	0.389227
bta-miR-185	-1.65207	1.915923	3.96788	0.046376	0.389227
Novel:NC_037553.1_23542	-0.78402	4.754584	3.947195	0.04695	0.389227
bta-miR-103	0.598915	14.00604	3.93836	0.047197	0.389227
Novel:hsa-miR-339-5p	-0.77748	4.319026	3.912656	0.047924	0.389227
Novel:hsa-miR-4800-5p	-0.57224	6.695168	3.910821	0.047976	0.389227

bta-miR-181d	-0.92869	3.668685	3.90095	0.048259	0.389227
bta-let-7i	0.661756	10.88837	3.876193	0.048975	0.39135
Novel:chi-miR-145-3p	-0.67825	5.910455	3.841849	0.049988	0.395779
Novel:NC_037556.1_29821	2.740327	1.037276	3.824893	0.050497	0.396169
Novel:NC_037547.1_8540	-0.46366	13.65086	3.788818	0.051596	0.396452
bta-miR-320a	-0.45843	13.73077	3.785517	0.051698	0.396452
bta-miR-2312	-1.94683	1.116738	3.778641	0.051911	0.396452
Novel:NC_037552.1_21299	-1.36866	2.369702	3.74554	0.052949	0.39733
bta-miR-200a	-1.12361	2.753102	3.737944	0.05319	0.39733
Novel:NC_037557.1_30645	-1.72133	2.004752	3.723443	0.053654	0.39733
Novel:NC_037564.1_40376	-0.50995	7.193875	3.716804	0.053867	0.39733
bta-miR-432	0.862741	4.71426	3.686877	0.054842	0.401092
Novel:hsa-miR-4537	-1.38098	1.994498	3.667043	0.055499	0.402481
bta-miR-2285cm	-1.40737	1.781547	3.630857	0.056718	0.407894
bta-miR-345-3p	-0.48759	7.004857	3.616682	0.057203	0.407984
bta-miR-2285bo	2.176148	1.386727	3.57645	0.058604	0.412796
Novel:NC_037545.1_1896	-2.12356	1.099884	3.558194	0.059252	0.412796
bta-miR-107	0.599022	13.002	3.554382	0.059388	0.412796
bta-miR-2285br	-1.31968	1.862083	3.543177	0.059791	0.412796
Novel:NC_037547.1_9353	-0.69765	5.413298	3.506794	0.061118	0.413197
bta-miR-2285au	1.882011	1.708454	3.506419	0.061131	0.413197
Novel:NC_037553.1_24652	-0.73888	4.697056	3.502257	0.061285	0.413197
Novel:NC_037547.1_7828	-0.78506	3.37268	3.461534	0.062812	0.417352
bta-miR-497	-0.53392	6.971978	3.460042	0.062869	0.417352
bta-miR-2285s	-1.4264	2.019282	3.419178	0.064443	0.422387
Novel:chi-miR-22-5p	-1.1947	1.866393	3.413995	0.064646	0.422387
bta-miR-101	0.638723	10.16765	3.402554	0.065096	0.422387
Novel:hsa-miR-6734-3p	-1.0899	8.002445	3.371478	0.066334	0.427212
Novel:NC_037546.1_5469	2.449186	1.021517	3.350838	0.067171	0.42794
Novel:hsa-miR-6715a-3p	-0.53281	7.033089	3.344281	0.067439	0.42794
Novel:NC_037552.1_22185	-1.29578	2.36705	3.281725	0.070055	0.441297
Novel:hsa-miR-4482-3p	1.0247	2.767106	3.266597	0.070704	0.442157
Novel:hsa-miR-10392-3p	1.666398	1.397766	3.230059	0.072298	0.448872
bta-miR-1247-3p	-0.89097	3.252555	3.188554	0.074156	0.452012
bta-miR-129	0.877749	4.153271	3.187051	0.074224	0.452012
bta-miR-210	-0.592	9.603203	3.183279	0.074395	0.452012
bta-miR-129-5p	0.877685	4.153271	3.172256	0.074899	0.452012
Novel:NC_037564.1_41171	-0.66287	7.93861	3.133915	0.076679	0.459544
bta-miR-6529b	-0.55764	8.967767	3.096211	0.078475	0.465865
bta-miR-29c	-0.52739	6.333785	3.089195	0.078814	0.465865
Novel:NC_037553.1_23783	0.611611	5.609087	3.066482	0.079922	0.466505
bta-miR-450b	0.734208	7.545075	3.064836	0.080003	0.466505
Novel:NC_037545.1_2589	1.389779	2.015128	3.03382	0.081546	0.472309
bta-miR-6529a	-0.54709	8.888167	2.999253	0.083303	0.474485
bta-miR-1388-5p	-0.51764	8.144295	2.997236	0.083407	0.474485
bta-miR-218	0.895722	2.98197	2.994049	0.083571	0.474485
Novel:NC_037545.1_558	1.032625	2.936692	2.966228	0.08502	0.477215
Novel:NC_037569.1_46660	2.021541	0.924127	2.963608	0.085158	0.477215
Novel:NC_037558.1_32782	0.72736	8.527868	2.952028	0.08577	0.477498

Novel:NC_037557.1_31157	-1.06649	2.264603	2.932974	0.086788	0.477498
bta-miR-323b-3p	-1.18233	2.163074	2.914699	0.087776	0.477498
Novel:NC_037558.1_32781	0.722565	8.52967	2.913976	0.087815	0.477498
Novel:chi-miR-3958-5p	0.923837	3.285218	2.911047	0.087975	0.477498
Novel:NC_037562.1_38320	-2.29365	0.755025	2.894033	0.088908	0.479548
Novel:hsa-miR-1199-3p	-0.67781	5.086656	2.873916	0.090026	0.482559
Novel:hsa-miR-1251-3p	-0.76114	9.003746	2.861013	0.09075	0.483426
bta-miR-331-5p	-0.60879	5.059752	2.851167	0.091308	0.483426
Novel:NC_037547.1_9224	1.989126	1.38682	2.826378	0.092727	0.487949
bta-miR-132	0.85869	3.97229	2.812506	0.093532	0.489202
Novel:NC_037569.1_46552	-0.86763	6.675854	2.787941	0.094976	0.490978
Novel:NC_037549.1_14266	-1.15795	2.199328	2.787378	0.09501	0.490978
bta-miR-182	-0.82057	2.542893	2.759301	0.09669	0.49669
Novel:hsa-miR-619-3p	-2.64085	1.029563	2.744934	0.097563	0.498207
Novel:hsa-miR-7106-5p	-2.4222	0.926217	2.71255	0.099562	0.502404
Novel:hsa-miR-6089	-1.1474	2.241472	2.693587	0.100753	0.502404
Novel:chi-miR-323b	0.755912	5.90931	2.685908	0.10124	0.502404
bta-miR-378d	-0.83042	2.611257	2.685552	0.101262	0.502404
Novel:hsa-miR-6832-3p	-1.7041	1.223858	2.685021	0.101296	0.502404
Novel:NC_037568.1_45368	-1.2787	1.779064	2.666247	0.102497	0.502625
bta-miR-11988	-1.8303	1.23367	2.666127	0.102505	0.502625
Novel:NC_037564.1_40741	-1.44499	1.713312	2.646277	0.103793	0.506063
Novel:NC_037560.1_34765	0.803466	3.301378	2.589006	0.107608	0.518737
Novel:NC_037552.1_22370	0.803463	3.301378	2.588545	0.107639	0.518737
Novel:hsa-miR-6868-3p	-1.26552	2.328063	2.579352	0.108266	0.518737
bta-miR-2299-5p	-0.88419	3.274595	2.571616	0.108797	0.518737
Novel:NC_037558.1_32628	-0.98824	1.900264	2.559915	0.109604	0.519718
bta-miR-362-3p	-0.85267	3.165671	2.51458	0.112798	0.529601
Novel:NC_037549.1_14209	-1.77853	1.184843	2.512928	0.112916	0.529601
bta-miR-324	1.326158	1.913644	2.486585	0.114821	0.533191
bta-miR-378c	-0.46035	7.046698	2.480095	0.115295	0.533191
bta-miR-11980	1.336167	1.800621	2.450347	0.117499	0.533191
bta-miR-1306	-1.22568	1.722093	2.448597	0.11763	0.533191
Novel:NC_037547.1_10102	-1.06958	2.338959	2.445825	0.117838	0.533191
Novel:NC_037548.1_11079	0.544647	7.972006	2.44147	0.118165	0.533191
Novel:NC_037549.1_16296	1.287118	1.833751	2.434806	0.118669	0.533191
bta-miR-188	-0.76112	3.98529	2.42745	0.119227	0.533191
Novel:NC_037545.1_1192	0.617937	3.05484	2.42725	0.119242	0.533191
Novel:NC_037557.1_30447	-0.94849	2.255856	2.394464	0.121765	0.541668
Novel:NC_037566.1_42967	-1.16889	2.053895	2.377874	0.123065	0.541863
bta-miR-21-5p	0.572995	11.61536	2.370658	0.123635	0.541863
Novel:NC_037547.1_8583	-0.5474	5.254436	2.369925	0.123693	0.541863
bta-miR-30d	0.537771	8.731629	2.355976	0.124804	0.543037
bta-miR-98	0.745873	5.76572	2.34898	0.125365	0.543037
bta-miR-199c	-0.67413	2.956899	2.334129	0.126566	0.543037
bta-miR-2904	-0.57848	4.31917	2.330843	0.126833	0.543037
bta-miR-335	-0.54153	5.383404	2.327486	0.127107	0.543037
Novel:NC_037548.1_11059	-2.41164	0.873483	2.311454	0.128424	0.54596
bta-miR-145	-0.47416	7.322062	2.291631	0.130073	0.547096

Novel:NC_037559.1_33968	-0.46886	9.34	2.286202	0.130529	0.547096
bta-miR-130b	-0.45897	7.953426	2.28544	0.130593	0.547096
Novel:NC_037552.1_22398	-0.54263	5.357459	2.274529	0.131515	0.548297
Novel:NC_037545.1_169	1.409848	1.786017	2.265883	0.132251	0.548714
Novel:NC_037569.1_46554	-0.81892	3.220513	2.253347	0.133326	0.550421
Novel:hsa-miR-1199-5p	1.776148	0.969251	2.244364	0.134102	0.550421
bta-miR-449a	0.975974	2.612749	2.225751	0.135727	0.550421
Novel:NC_037568.1_44853	-1.61874	1.313985	2.224389	0.135846	0.550421
bta-miR-31	0.439818	7.524877	2.224334	0.135851	0.550421
bta-miR-494	0.849593	2.320645	2.196605	0.138315	0.557785
Novel:NC_037569.1_46557	-0.70133	4.067949	2.176137	0.140166	0.562619
bta-miR-370	1.368979	1.510231	2.151106	0.142467	0.567033
Novel:NC_037548.1_12074	-0.67123	9.328697	2.14694	0.142854	0.567033
bta-miR-874	-0.60469	4.140385	2.139177	0.143579	0.567033
Novel:chi-miR-7-5p	-1.34415	1.60512	2.130208	0.144421	0.567033
bta-miR-484	-0.45256	6.919158	2.128834	0.144551	0.567033
Novel:NC_037560.1_35724	-1.32274	1.428368	2.120609	0.145329	0.567506
Novel:hsa-miR-372-5p	1.381755	1.228486	2.079721	0.149267	0.58026
Novel:hsa-miR-3974	1.39454	2.121478	2.071572	0.150067	0.58043
Novel:NC_037545.1_315	-1.17392	1.834487	2.065594	0.150656	0.58043
Novel:NC_037556.1_30323	-1.54393	1.249339	2.05216	0.15199	0.58224
Novel:hsa-miR-548v	-0.46758	6.773284	2.046026	0.152604	0.58224
Novel:hsa-miR-1184	2.054367	1.118663	2.035762	0.153637	0.58224
bta-miR-2285bw	-0.55844	4.03653	2.023219	0.15491	0.58224
Novel:chi-miR-151-5p	-1.09287	1.697477	2.017144	0.155531	0.58224
bta-miR-502a	-0.67384	3.348507	2.017021	0.155544	0.58224
Novel:hsa-miR-4746-5p	-0.68852	3.855013	2.013795	0.155875	0.58224
bta-miR-379	1.144822	1.867853	2.007497	0.156523	0.58224
Novel:NC_037545.1_1689	-1.10218	1.869481	1.994565	0.157864	0.583735
Novel:chi-miR-127-3p	-0.90521	2.662173	1.990601	0.158278	0.583735
Novel:NC_037547.1_10260	0.908688	2.180576	1.974577	0.159963	0.587438
Novel:hsa-miR-6781-5p	1.403085	1.301194	1.965272	0.160951	0.588561
bta-miR-503-3p	0.869365	2.240763	1.946513	0.162963	0.590848
Novel:NC_037545.1_583	-1.74465	1.015086	1.944909	0.163137	0.590848
Novel:NC_037553.1_23622	-1.39425	1.174771	1.937766	0.163911	0.590848
bta-miR-2285k	-1.10525	1.281109	1.934064	0.164315	0.590848
bta-miR-339a	-0.40542	7.429599	1.921705	0.165669	0.593246
Novel:NC_037554.1_24951	-0.38144	6.274806	1.913071	0.166623	0.594195
Novel:NC_037564.1_40236	-0.98086	2.462441	1.903063	0.167736	0.595704
Novel:NC_037547.1_10214	0.414836	7.653818	1.881336	0.170182	0.601915
Novel:NC_037550.1_17628	-0.42947	7.6362	1.873234	0.171105	0.602245
Novel:NC_037566.1_43276	1.954484	1.208141	1.866306	0.171899	0.602245
Novel:NC_037556.1_29299	-1.45696	1.214956	1.862218	0.172369	0.602245
Novel:hsa-miR-4514	-0.83275	2.886493	1.855782	0.173112	0.602403
bta-miR-16a	0.638158	4.284999	1.843986	0.174485	0.602529
bta-miR-411c-5p	2.295094	0.982704	1.843468	0.174545	0.602529
bta-miR-369-3p	0.654811	2.78219	1.814689	0.177947	0.611825
bta-miR-2299-3p	-1.85215	0.979402	1.801634	0.179515	0.614508
Novel:NC_037555.1_26499	1.267341	1.467932	1.794566	0.180371	0.614508

bta-miR-151-3p	-0.39875	10.32431	1.788309	0.181132	0.614508
bta-miR-760-3p	-0.41293	7.47145	1.781809	0.181927	0.614508
Novel:NC_037556.1_29648	-1.47192	1.331085	1.773676	0.182928	0.614508
bta-miR-12057	-1.33845	1.376483	1.773094	0.183	0.614508
Novel:NC_037548.1_12259	0.470739	5.228139	1.76643	0.183825	0.614886
bta-miR-186	-0.46425	8.853881	1.750805	0.185776	0.619013
Novel:NC_037551.1_19861	1.291496	1.192878	1.743829	0.186654	0.619549
Novel:hsa-miR-5193	-0.79516	2.204467	1.731338	0.18824	0.622328
Novel:NC_037562.1_38494	0.892951	1.898059	1.723234	0.189277	0.622328
Novel:NC_037546.1_4225	0.953926	2.741856	1.717855	0.189969	0.622328
Novel:hsa-miR-1237-3p	-0.34068	13.68118	1.714704	0.190376	0.622328
bta-miR-378	-0.34056	13.64633	1.708103	0.191232	0.622766
bta-miR-2285by	0.738692	2.2796	1.696197	0.192786	0.623993
bta-miR-339b	-0.37482	7.26111	1.694153	0.193054	0.623993
Novel:hsa-miR-591	0.657046	3.662172	1.685306	0.194221	0.62542
Novel:hsa-miR-3919	0.824003	2.400503	1.671096	0.196112	0.627855
bta-miR-411a	0.477337	5.634665	1.663897	0.197078	0.627855
Novel:NC_037545.1_434	1.002442	1.740796	1.656718	0.198047	0.627855
Novel:NC_037549.1_14540	-1.67222	1.805012	1.656319	0.198101	0.627855
bta-miR-1296	0.457518	5.230527	1.652015	0.198685	0.627855
bta-miR-2285cj	-0.57671	3.576207	1.645039	0.199635	0.627855
Novel:hsa-miR-149-3p	1.729073	1.118174	1.641866	0.20007	0.627855
bta-miR-2284y	-0.44232	5.0426	1.627521	0.202046	0.630202
bta-miR-6518	-0.41625	5.538955	1.620826	0.202977	0.630202
Novel:hsa-miR-943	-1.32091	1.280578	1.613336	0.204023	0.630202
Novel:chi-miR-1307-3p	-0.41158	6.812597	1.600772	0.205794	0.630202
Novel:hsa-miR-4308	0.467915	4.879518	1.600549	0.205825	0.630202
Novel:NC_037550.1_18908	-0.43866	8.826386	1.600117	0.205887	0.630202
Novel:NC_037546.1_6204	-1.38372	1.162265	1.59497	0.206617	0.630202
bta-miR-378b	-0.38542	6.972281	1.594676	0.206659	0.630202
Novel:chi-miR-125a-5p	-0.50875	4.473191	1.572219	0.209885	0.637785
bta-miR-421	0.531399	4.826969	1.562358	0.21132	0.639893
Novel:NC_037545.1_1965	-1.76548	1.013785	1.553498	0.21262	0.641124
Novel:chi-miR-671-5p	-0.91582	1.677037	1.546985	0.213581	0.641124
Novel:NC_037563.1_39275	0.808649	2.209479	1.536474	0.215143	0.641124
Novel:NC_037568.1_45672	-1.89301	0.980497	1.534916	0.215376	0.641124
Novel:NC_037551.1_19947	0.808644	2.209479	1.534479	0.215441	0.641124
bta-miR-11972	0.979283	1.684552	1.523536	0.217085	0.643796
Novel:NC_037564.1_40345	0.452395	5.704946	1.513037	0.218676	0.646293
Novel:NC_037549.1_15915	-1.6962	1.180258	1.495144	0.22142	0.650999
Novel:NC_037567.1_44553	-0.62223	3.718446	1.492834	0.221777	0.650999
Novel:chi-miR-378-3p	-0.6045	2.657152	1.485915	0.222851	0.651935
bta-miR-26b	0.379183	7.222355	1.479345	0.223877	0.652723
bta-miR-2468	-0.77255	2.417928	1.452817	0.228077	0.662727
bta-miR-11971	-0.6462	3.775849	1.44134	0.229923	0.66542
Novel:NC_037545.1_824	0.377914	9.670126	1.437494	0.230545	0.66542
Novel:hsa-miR-4515	-0.3864	7.026583	1.432557	0.231348	0.66551
Novel:NC_037546.1_4132	-1.31169	1.132636	1.42208	0.233061	0.668134
Novel:hsa-miR-3714	-0.60814	9.388423	1.410016	0.235054	0.668134

Novel:chi-miR-125a-3p	1.252232	0.798463	1.409145	0.235198	0.668134
Novel:NC_037558.1_32328	2.217591	0.896194	1.408194	0.235356	0.668134
bta-miR-2285bz	0.442956	5.361087	1.401918	0.236403	0.668903
Novel:NC_037552.1_22617	-1.00217	2.132185	1.393985	0.237733	0.669172
bta-miR-33b	1.190042	1.680012	1.392115	0.238048	0.669172
bta-miR-2285av	-0.65326	2.429801	1.371075	0.241627	0.677027
Novel:hsa-miR-6834-5p	-0.63096	2.902806	1.354293	0.244529	0.678961
Novel:NC_037555.1_28194	1.128249	1.190671	1.353119	0.244734	0.678961
Novel:NC_037547.1_10537	-0.32072	6.352544	1.349963	0.245285	0.678961
Novel:NC_037560.1_35426	0.559291	7.034216	1.348934	0.245465	0.678961
bta-miR-130a	-0.26522	13.25457	1.33704	0.247557	0.681109
Novel:NC_037561.1_36258	-0.90397	1.696265	1.335555	0.247819	0.681109
Novel:NC_037567.1_44107	-0.5816	3.683211	1.319175	0.25074	0.682325
Novel:NC_037553.1_24374	-0.48484	4.435379	1.317052	0.251122	0.682325
bta-miR-664b	-1.6576	1.005463	1.311642	0.252098	0.682325
Novel:hsa-miR-203a-3p	-0.84459	2.387522	1.31063	0.252281	0.682325
Novel:hsa-miR-1205	0.676224	2.25081	1.307033	0.252933	0.682325
bta-miR-2285db	1.741167	0.763345	1.302843	0.253695	0.682325
Novel:hsa-miR-4428	0.481057	5.639157	1.301798	0.253885	0.682325
bta-miR-6517	-0.34824	5.666447	1.297956	0.254587	0.682325
Novel:hsa-miR-4721	-1.26708	0.94839	1.270205	0.259728	0.693209
Novel:NC_037545.1_498	-0.33139	8.767817	1.267308	0.260272	0.693209
bta-miR-99b	-0.55069	12.01332	1.263138	0.261058	0.693209
bta-miR-2285f	-0.45434	3.280417	1.252409	0.263093	0.694644
Novel:NC_037545.1_2499	0.568119	9.546542	1.251806	0.263208	0.694644
Novel:hsa-miR-2115-5p	0.331558	12.26054	1.239155	0.265634	0.698909
Novel:NC_037547.1_8560	-0.30323	6.363159	1.22838	0.267722	0.699932
bta-miR-592	0.861966	2.01459	1.227624	0.26787	0.699932
bta-miR-128	-0.36423	8.571693	1.224619	0.268456	0.699932
bta-miR-499	0.978441	1.381111	1.218895	0.269578	0.70074
Novel:oar-miR-299-3p	1.888645	0.993849	1.198156	0.273691	0.708413
Novel:NC_037554.1_24998	0.6407	3.402837	1.195756	0.274172	0.708413
bta-miR-2285b	0.77802	2.481944	1.186377	0.276062	0.711168
bta-miR-2890	0.425573	4.076167	1.178207	0.277721	0.713314
Novel:hsa-miR-6784-3p	-1.12743	0.962697	1.155923	0.282313	0.722955
bta-miR-193a-5p	-0.53921	4.024352	1.142448	0.285136	0.72662
Novel:hsa-miR-136-3p	0.829774	1.959349	1.139714	0.285713	0.72662
Novel:hsa-miR-149-5p	-0.67251	1.853561	1.132433	0.287257	0.72662
bta-miR-2285v	-0.9348	1.49234	1.13148	0.28746	0.72662
bta-miR-330	-1.15541	1.439368	1.128082	0.288185	0.72662
bta-miR-660	0.290613	12.16388	1.125134	0.288816	0.72662
bta-miR-133a	0.578342	3.142725	1.121305	0.289638	0.72662
Novel:hsa-miR-198	-0.83255	4.072894	1.11213	0.291619	0.728463
bta-miR-193a-3p	-0.47194	4.818165	1.110099	0.29206	0.728463
Novel:chi-miR-499-3p	0.921121	1.439355	1.105311	0.293103	0.728958
Novel:NC_037558.1_33194	1.956447	0.829462	1.098421	0.294613	0.730606
Novel:chi-miR-500-5p	-0.48068	2.951319	1.089441	0.296595	0.733414
Novel:NC_037547.1_8180	-1.33861	1.19856	1.082963	0.298036	0.734871
Novel:NC_037569.1_46549	0.283742	12.16525	1.075653	0.299672	0.7368

Novel:NC_037569.1_47143	1.136511	0.888697	1.053891	0.304613	0.74682
Novel:NC_037552.1_22028	1.124099	1.37504	1.031487	0.30981	0.752835
Novel:NC_037552.1_21136	1.124289	1.37504	1.031077	0.309906	0.752835
Novel:hsa-miR-7704	1.417895	1.04771	1.025241	0.311279	0.752835
Novel:NC_037561.1_36931	-0.31626	7.366875	1.018451	0.312887	0.752835
Novel:hsa-miR-765	1.504279	0.597397	1.018175	0.312952	0.752835
Novel:NC_037564.1_41155	0.384536	3.807081	1.014952	0.313719	0.752835
bta-miR-181c	-0.38664	3.883643	1.01153	0.314537	0.752835
bta-miR-2284z	0.656615	2.625135	1.010621	0.314754	0.752835
Novel:hsa-miR-6814-5p	0.589631	3.445328	1.006639	0.315709	0.752835
Novel:NC_037553.1_23923	-0.90912	1.421407	1.006306	0.315789	0.752835
Novel:NC_037559.1_33762	1.093787	1.015255	0.984384	0.321119	0.763431
Novel:NC_037564.1_40415	0.957935	1.907681	0.978	0.322693	0.765066
Novel:hsa-miR-6885-3p	-1.50639	1.053312	0.970362	0.32459	0.765809
Novel:NC_037569.1_46466	-0.31741	9.997627	0.969595	0.324781	0.765809
bta-miR-409b	0.313514	6.958734	0.964214	0.326128	0.766347
Novel:NC_037548.1_12197	-0.45089	2.711814	0.961595	0.326785	0.766347
Novel:NC_037546.1_4816	0.927679	1.607554	0.956599	0.328045	0.767217
bta-miR-2440	-0.52028	5.872005	0.952748	0.329021	0.767419
bta-miR-1224	-0.5815	3.65056	0.940367	0.332183	0.772707
bta-miR-152	-0.32564	8.488006	0.934889	0.333596	0.773906
Novel:hsa-miR-4491	0.665293	1.756164	0.931424	0.334493	0.773908
bta-miR-154c	0.377079	3.463731	0.919279	0.337664	0.779156
Novel:hsa-miR-4767	-1.24243	1.177265	0.903832	0.341756	0.786495
Novel:hsa-miR-5190	0.719048	1.963134	0.896338	0.343766	0.788209
Novel:NC_037552.1_21285	-1.32184	0.96405	0.894251	0.344328	0.788209
bta-miR-1343-5p	-0.74539	1.848976	0.884201	0.347054	0.790375
Novel:NC_037547.1_9992	0.383444	6.654487	0.884011	0.347106	0.790375
Novel:hsa-miR-1290	0.55869	3.033662	0.865556	0.352188	0.79727
Novel:hsa-miR-486-3p	1.171923	0.969853	0.865297	0.352261	0.79727
bta-miR-142-5p	0.626581	3.685602	0.862983	0.352905	0.79727
bta-miR-2285cr	-0.69707	1.779077	0.8501	0.356524	0.800382
Novel:NC_037564.1_40307	0.4784	4.025599	0.843809	0.35831	0.800382
Novel:NC_037558.1_33041	1.071858	1.102891	0.843565	0.358379	0.800382
Novel:NC_037548.1_12165	-0.64113	2.163525	0.842302	0.358739	0.800382
bta-miR-1388-3p	-0.26172	6.251751	0.841622	0.358933	0.800382
bta-miR-2435	-0.31434	5.486788	0.837584	0.360089	0.800382
Novel:NC_037563.1_39679	0.3152	6.494049	0.83271	0.36149	0.800382
Novel:NC_037548.1_12213	0.727035	1.188352	0.831974	0.361702	0.800382
bta-miR-92a	-0.20598	14.34975	0.826763	0.36321	0.801664
Novel:NC_037558.1_32528	1.101698	1.461494	0.817348	0.365957	0.80489
bta-miR-221	-0.28901	10.03584	0.81537	0.366537	0.80489
Novel:NC_037546.1_3761	-0.87723	2.149004	0.80431	0.369808	0.807768
Novel:NC_037555.1_27093	-0.94902	1.318994	0.800159	0.371046	0.807768
Novel:oar-miR-134-3p	-0.86386	1.717807	0.794546	0.372729	0.807768
Novel:NC_037569.1_48167	0.971301	0.843753	0.794415	0.372768	0.807768
bta-miR-17-3p	-0.9539	1.387726	0.793274	0.373112	0.807768
Novel:hsa-miR-12117	1.282287	1.057318	0.792106	0.373464	0.807768
bta-miR-127	-0.28985	9.938267	0.779717	0.377228	0.813869

bta-miR-30a-5p	0.262936	12.21308	0.761406	0.382889	0.824023
Novel:hsa-miR-4467	1.033812	1.05494	0.749656	0.386585	0.829908
Novel:NC_037569.1_46961	-0.25647	6.048232	0.740162	0.389609	0.830442
Novel:NC_037557.1_31142	-0.25647	6.048232	0.740088	0.389633	0.830442
Novel:NC_037564.1_41452	-0.25647	6.048232	0.739813	0.389721	0.830442
Novel:hsa-miR-6771-5p	-0.35475	4.35051	0.727215	0.393787	0.834795
bta-miR-19b	-0.29965	10.1332	0.724926	0.394533	0.834795
bta-miR-1839	0.267706	8.893869	0.724518	0.394666	0.834795
Novel:NC_037566.1_43265	-0.50577	2.285626	0.720283	0.396051	0.835678
bta-miR-2285dd	-0.57652	1.513467	0.715835	0.397513	0.835845
Novel:hsa-miR-548d-3p	-1.06859	0.725734	0.714157	0.398067	0.835845
bta-miR-2285ce	-0.48104	2.248285	0.710177	0.399385	0.836576
bta-miR-148c	-0.28602	8.777166	0.70495	0.401125	0.838187
Novel:NC_037564.1_40946	-1.1504	1.145892	0.69647	0.403972	0.840363
Novel:NC_037553.1_24557	-1.03178	0.945576	0.692893	0.405182	0.840363
bta-miR-184	-1.07299	0.950462	0.692628	0.405272	0.840363
Novel:NC_037552.1_21636	1.209335	0.808692	0.68747	0.407026	0.840363
Novel:NC_037567.1_44673	-1.36548	1.005957	0.687443	0.407035	0.840363
Novel:NC_037553.1_23515	-1.15683	0.943813	0.683476	0.408392	0.841152
bta-miR-1246	-0.26904	11.3519	0.678888	0.40997	0.842391
Novel:hsa-miR-3129-3p	0.313828	7.168345	0.674433	0.41151	0.843387
Novel:hsa-miR-4800-3p	0.628849	1.922684	0.67079	0.412776	0.843387
Novel:NC_037554.1_25273	0.975789	1.258848	0.655215	0.418254	0.843387
bta-miR-30b-5p	0.362553	3.758453	0.648755	0.420558	0.843387
Novel:NC_037564.1_40326	0.247166	6.972849	0.648576	0.420622	0.843387
Novel:NC_037553.1_22953	-0.38374	3.078829	0.647593	0.420974	0.843387
bta-miR-27a-5p	-1.17291	1.400976	0.647051	0.421169	0.843387
Novel:NC_037545.1_1494	0.407123	2.498127	0.64562	0.421683	0.843387
Novel:NC_037564.1_40744	-0.23284	6.140573	0.644784	0.421984	0.843387
Novel:NC_037557.1_30428	-0.23284	6.140573	0.644484	0.422092	0.843387
Novel:NC_037559.1_34348	0.861925	0.939235	0.644204	0.422192	0.843387
Novel:NC_037553.1_24219	0.688361	1.209617	0.640805	0.423419	0.843387
bta-miR-2892	0.392784	5.370126	0.638506	0.424252	0.843387
Novel:NC_037550.1_18832	0.91019	1.01823	0.633037	0.426244	0.843387
Novel:NC_037564.1_40897	0.87009	1.205491	0.62865	0.427851	0.843387
bta-miR-202	0.468783	9.38446	0.624475	0.429389	0.843387
Novel:NC_037555.1_27478	1.124461	0.745737	0.619284	0.431314	0.843387
Novel:NC_037555.1_26458	1.120482	0.745179	0.618526	0.431595	0.843387
Novel:NC_037569.1_47983	-0.22807	6.142678	0.61753	0.431967	0.843387
bta-miR-2285da	-0.52416	1.69204	0.616517	0.432345	0.843387
bta-miR-2285z	-0.37096	3.143187	0.611746	0.434131	0.843387
Novel:NC_037546.1_4370	-0.273	10.78655	0.611077	0.434383	0.843387
Novel:NC_037561.1_36340	-0.9268	1.238178	0.609612	0.434934	0.843387
Novel:NC_037551.1_19485	-0.59865	1.680423	0.607179	0.435852	0.843387
Novel:NC_037551.1_20645	-0.9253	1.24414	0.606808	0.435992	0.843387
Novel:chi-miR-2331	0.786571	1.673616	0.60291	0.43747	0.843387
bta-miR-1247-5p	0.390323	3.843226	0.60028	0.438471	0.843387
Novel:hsa-miR-652-5p	0.287842	5.30571	0.599574	0.438741	0.843387
bta-miR-34c	-0.58744	2.541589	0.596304	0.439992	0.843387

bta-miR-2285bn	-0.42438	2.613086	0.593016	0.441255	0.843387
Novel:NC_037564.1_41137	0.741751	1.829418	0.586957	0.443598	0.843387
bta-miR-151-5p	-0.23109	6.861045	0.586789	0.443664	0.843387
Novel:NC_037567.1_44092	-0.26848	10.64746	0.586783	0.443666	0.843387
bta-miR-365-3p	0.476778	3.063498	0.58674	0.443682	0.843387
bta-miR-12006	0.652495	1.635486	0.581147	0.445863	0.84567
Novel:NC_037560.1_35881	0.567679	1.467853	0.567211	0.451369	0.847288
bta-miR-381	0.231751	6.878338	0.56581	0.451929	0.847288
bta-miR-2484	1.076929	0.919446	0.555749	0.455978	0.847288
bta-miR-27a-3p	-0.22404	7.948595	0.555322	0.456151	0.847288
Novel:hsa-miR-8077	0.31475	4.477388	0.554565	0.456458	0.847288
bta-miR-424-5p	-0.24031	6.021019	0.552373	0.45735	0.847288
Novel:chi-miR-485-5p	0.568514	1.832502	0.550954	0.457928	0.847288
bta-miR-99a-5p	-0.38666	9.108653	0.549996	0.45832	0.847288
bta-miR-1343-3p	0.327131	4.316624	0.549388	0.458568	0.847288
Novel:NC_037545.1_503	-0.42486	8.869565	0.549226	0.458634	0.847288
bta-miR-147	-1.08045	1.124524	0.544539	0.460558	0.847288
Novel:hsa-miR-3654	-0.69407	2.524317	0.542089	0.461568	0.847288
Novel:NC_037564.1_41423	0.418533	2.253351	0.535027	0.464501	0.847288
Novel:NC_037550.1_17420	-0.41359	3.276573	0.535026	0.464501	0.847288
bta-miR-28	0.372593	3.206369	0.532812	0.465427	0.847288
Novel:hsa-miR-5589-5p	-0.2712	5.254535	0.531149	0.466124	0.847288
bta-miR-26a	0.222733	10.31227	0.529882	0.466656	0.847288
Novel:NC_037564.1_40338	0.860132	1.292967	0.529569	0.466788	0.847288
Novel:hsa-miR-6776-5p	0.912578	1.18856	0.526658	0.468015	0.847288
bta-miR-374b	0.388532	2.663226	0.52614	0.468234	0.847288
bta-miR-191b	-0.59163	2.358217	0.525454	0.468524	0.847288
Novel:NC_037563.1_40142	1.242103	0.642147	0.523752	0.469245	0.847288
bta-miR-23b-3p	-0.25998	4.646165	0.522542	0.469759	0.847288
Novel:NC_037555.1_27170	-0.78238	1.444522	0.52036	0.470688	0.847288
Novel:NC_037552.1_21735	1.251864	0.725303	0.519018	0.471261	0.847288
Novel:NC_037547.1_8021	-1.13354	0.824712	0.516625	0.472285	0.847363
bta-miR-23a	-0.19762	6.151218	0.512172	0.4742	0.849035
Novel:NC_037564.1_40729	0.226018	8.815798	0.507065	0.476412	0.849802
Novel:hsa-miR-1304-3p	-1.13721	0.968675	0.502531	0.47839	0.849802
Novel:hsa-miR-759	-0.4057	2.500039	0.499656	0.479651	0.849802
Novel:NC_037546.1_6394	-1.08917	0.85267	0.49351	0.482366	0.849802
Novel:hsa-miR-185-3p	-0.27483	3.631212	0.493242	0.482485	0.849802
Novel:hsa-miR-6849-3p	-1.05033	1.03718	0.491907	0.483078	0.849802
bta-miR-194	-0.88658	1.360026	0.490605	0.483657	0.849802
bta-miR-26c	0.213625	10.43829	0.488862	0.484436	0.849802
Novel:NC_037564.1_40695	0.859616	0.78173	0.487003	0.485267	0.849802
Novel:hsa-miR-744-5p	0.783199	1.351039	0.481555	0.487719	0.849802
Novel:NC_037547.1_8809	0.651653	1.337794	0.479468	0.488664	0.849802
bta-miR-6520	0.265904	4.602453	0.475029	0.490683	0.849802
Novel:hsa-miR-4687-5p	-0.48013	1.769136	0.473138	0.491547	0.849802
bta-miR-15b	0.357484	2.950566	0.46792	0.493946	0.849802
Novel:chi-miR-3432-5p	0.518636	2.669421	0.466717	0.494502	0.849802
bta-miR-2285q	-0.32485	3.117366	0.465792	0.494929	0.849802

bta-miR-2340	-1.04414	0.648203	0.464347	0.4956	0.849802
bta-let-7a-3p	0.249886	4.469017	0.4637	0.4959	0.849802
Novel:NC_037547.1_9951	-0.27296	4.867928	0.463171	0.496146	0.849802
bta-miR-199b	-0.44063	2.915331	0.46118	0.497073	0.849802
Novel:hsa-miR-4720-5p	0.248416	5.136067	0.460864	0.497221	0.849802
Novel:NC_037558.1_31842	-0.83599	1.23339	0.460703	0.497296	0.849802
Novel:NC_037568.1_45118	-0.57654	2.031899	0.460684	0.497304	0.849802
Novel:NC_037557.1_30640	-0.24856	9.97009	0.458638	0.498261	0.849802
Novel:chi-miR-22-3p	-0.45095	1.862075	0.454004	0.50044	0.850403
Novel:NC_037550.1_17382	-0.7861	0.819032	0.45356	0.500649	0.850403
Novel:NC_037550.1_18493	-0.78544	0.819032	0.451613	0.50157	0.850403
Novel:NC_037552.1_21699	0.985429	0.836466	0.447746	0.503407	0.851844
Novel:chi-miR-320-3p	-0.63547	1.277468	0.445414	0.504521	0.852058
bta-miR-2355-3p	1.040104	0.560441	0.440467	0.506897	0.854399
Novel:hsa-miR-1321	0.619873	1.250173	0.43418	0.509945	0.854759
bta-miR-10164-3p	-0.3058	3.29045	0.431798	0.511108	0.854759
bta-miR-146a	-0.65568	1.905501	0.431701	0.511156	0.854759
bta-miR-93	0.200355	8.766159	0.429814	0.51208	0.854759
bta-miR-326	-0.30743	3.003647	0.429459	0.512255	0.854759
bta-miR-873	0.42437	4.058073	0.426936	0.513496	0.854759
Novel:NC_037565.1_42675	-0.47841	2.445256	0.425826	0.514044	0.854759
Novel:hsa-miR-635	-0.75984	1.263038	0.420333	0.516771	0.85729
Novel:NC_037560.1_35884	-0.34617	9.471285	0.411091	0.521416	0.85729
bta-miR-139	-0.78547	1.261497	0.408241	0.522864	0.85729
Novel:chi-miR-411b-5p	0.34686	3.017534	0.407354	0.523316	0.85729
bta-miR-301a	-0.25123	4.84206	0.407031	0.52348	0.85729
Novel:hsa-miR-3150b-3p	0.789355	0.96003	0.406838	0.523579	0.85729
Novel:NC_037568.1_44905	0.194944	8.755579	0.405792	0.524113	0.85729
Novel:NC_037556.1_28412	-0.27783	6.228245	0.40294	0.525575	0.85729
Novel:hsa-miR-1252-5p	0.679712	1.271225	0.400649	0.526754	0.85729
Novel:NC_037569.1_46653	0.588466	1.767314	0.399868	0.527158	0.85729
bta-miR-100	-0.33884	9.485354	0.399652	0.527269	0.85729
bta-miR-545-5p	0.588897	1.767314	0.399231	0.527487	0.85729
Novel:NC_037547.1_9101	-0.297	3.335588	0.390167	0.532212	0.860198
bta-miR-2389	-0.57908	1.484411	0.389758	0.532427	0.860198
Novel:NC_037555.1_27691	-0.63192	1.248336	0.386447	0.534173	0.860198
bta-miR-30c	0.24095	4.933465	0.386366	0.534216	0.860198
Novel:hsa-miR-3670	0.429691	2.105183	0.385759	0.534537	0.860198
Novel:NC_037549.1_16477	-0.29052	4.613878	0.380782	0.537185	0.860198
Novel:hsa-miR-7113-3p	-0.20433	11.51543	0.379523	0.537859	0.860198
bta-miR-148b	-0.18922	7.869699	0.377672	0.538852	0.860198
bta-miR-141	0.298195	3.272399	0.377134	0.539141	0.860198
Novel:NC_037549.1_15054	0.662412	1.332842	0.375459	0.540044	0.860198
bta-miR-191	-0.21047	10.38731	0.375095	0.54024	0.860198
Novel:NC_037550.1_17830	-0.57935	1.227427	0.356341	0.550545	0.870076
bta-miR-744	0.174451	6.715238	0.354397	0.551635	0.870076
bta-miR-2284f	0.542463	1.349607	0.35386	0.551936	0.870076
Novel:hsa-miR-5002-5p	-0.38522	2.061955	0.351643	0.553185	0.870076
Novel:chi-miR-491-5p	-0.2756	2.849792	0.34561	0.556609	0.870076

Novel:hsa-miR-4447	-0.50277	1.419192	0.343208	0.557983	0.870076
Novel:NC_037549.1_16548	-0.40548	1.973684	0.341694	0.558853	0.870076
Novel:NC_037549.1_15080	-0.54637	5.77806	0.341589	0.558914	0.870076
Novel:hsa-miR-6846-5p	-0.74557	0.951036	0.341271	0.559097	0.870076
Novel:NC_037567.1_44260	0.171465	6.108848	0.341223	0.559124	0.870076
bta-miR-652	0.189456	5.736799	0.340866	0.55933	0.870076
bta-miR-2285j	0.616708	1.295641	0.33967	0.56002	0.870076
bta-miR-10174-3p	-0.19264	5.425126	0.337038	0.561544	0.870076
Novel:NC_037546.1_5554	-0.33898	2.193411	0.334747	0.562877	0.870076
Novel:NC_037550.1_18986	-0.57764	1.229568	0.334491	0.563027	0.870076
Novel:NC_037546.1_3187	0.913083	0.856986	0.332488	0.564198	0.870076
Novel:hsa-miR-12135	0.314977	2.371911	0.332224	0.564353	0.870076
bta-miR-25	-0.12273	14.62243	0.331816	0.564592	0.870076
Novel:hsa-miR-5587-3p	-0.58722	1.322802	0.329692	0.56584	0.870446
bta-miR-2411-5p	1.011802	0.663171	0.326383	0.567797	0.871746
Novel:NC_037547.1_8898	-0.71617	1.020413	0.324631	0.568838	0.871746
Novel:hsa-miR-4677-3p	0.698821	1.090446	0.319329	0.572011	0.871746
bta-miR-361	-0.20117	8.369811	0.319041	0.572185	0.871746
bta-miR-3432a	0.859669	0.866737	0.318794	0.572333	0.871746
bta-miR-2403	0.433578	1.965048	0.316581	0.573669	0.871746
Novel:NC_037566.1_43137	0.222797	6.567995	0.315337	0.574423	0.871746
Novel:NC_037554.1_25983	-0.44091	1.428317	0.314773	0.574766	0.871746
bta-miR-2478	0.537727	1.671845	0.310496	0.577376	0.872715
bta-miR-195	0.22541	4.795138	0.310411	0.577428	0.872715
bta-miR-224	0.458738	1.933585	0.308163	0.578809	0.873274
Novel:NC_037549.1_16572	0.544334	1.365596	0.303429	0.58174	0.876164
Novel:NC_037567.1_44693	0.163511	6.022455	0.29094	0.589619	0.885427
Novel:hsa-miR-4276	-0.40103	2.14783	0.288965	0.590884	0.885427
bta-miR-22-5p	-0.19693	5.047691	0.288834	0.590969	0.885427
bta-miR-452	0.320781	2.33678	0.284991	0.593448	0.886498
Novel:NC_037556.1_29371	-0.63673	0.882135	0.283514	0.594407	0.886498
Novel:NC_037547.1_7559	0.157782	6.694501	0.282964	0.594765	0.886498
Novel:hsa-miR-3169	0.902061	0.821725	0.278995	0.597361	0.888286
Novel:NC_037550.1_19123	-0.54057	0.925516	0.276951	0.598707	0.888286
Novel:NC_037564.1_40877	-0.39828	2.144811	0.275402	0.599731	0.888286
Novel:NC_037564.1_41300	0.482898	1.615889	0.274873	0.600082	0.888286
Novel:hsa-miR-548a-3p	-0.15412	6.144534	0.272531	0.601639	0.888849
bta-miR-199a-3p	0.163425	7.462984	0.26991	0.603392	0.888849
Novel:NC_037564.1_40336	-0.72533	0.87342	0.269671	0.603552	0.888849
Novel:hsa-miR-3122	-0.59594	0.775097	0.266579	0.605636	0.890398
Novel:hsa-miR-4663	0.749031	0.94692	0.264921	0.606759	0.890533
Novel:NC_037546.1_4218	-0.31832	2.210712	0.260379	0.60986	0.893564
bta-miR-487b	-0.59604	1.297248	0.257506	0.611839	0.894943
Novel:hsa-miR-4753-5p	0.389028	1.945999	0.253534	0.614597	0.89514
bta-miR-1307	-0.14181	6.187165	0.248981	0.617794	0.89514
Novel:NC_037569.1_47087	-0.15993	6.130017	0.247669	0.618721	0.89514
bta-miR-2285ba	0.193938	3.46953	0.246533	0.619527	0.89514
Novel:NC_037558.1_32026	-0.15146	10.36219	0.245092	0.620552	0.89514
Novel:NC_037555.1_27132	0.699932	0.913093	0.244926	0.620671	0.89514

Novel:hsa-miR-6808-3p	0.538841	1.14048	0.244639	0.620876	0.89514
bta-miR-2285x	0.569403	1.212049	0.244077	0.621277	0.89514
Novel:NC_037561.1_36813	0.595089	0.972902	0.238794	0.625079	0.89514
bta-miR-877	-0.18004	5.212886	0.238655	0.62518	0.89514
Novel:hsa-miR-4468	0.594651	0.972902	0.237615	0.625934	0.89514
Novel:hsa-miR-3917	0.677757	1.239532	0.237304	0.62616	0.89514
Novel:NC_037569.1_46587	0.332497	2.166231	0.236466	0.626771	0.89514
Novel:NC_037569.1_47131	0.399313	2.024696	0.236	0.62711	0.89514
Novel:NC_037566.1_43625	0.195154	6.595604	0.235423	0.627532	0.89514
Novel:NC_037546.1_2906	-0.38688	2.384874	0.231717	0.630253	0.897539
Novel:NC_037549.1_15024	-0.32903	2.107562	0.22957	0.631843	0.89832
bta-miR-3604	0.15537	8.110709	0.227612	0.6333	0.898417
Novel:hsa-miR-4707-5p	-0.47211	1.119932	0.225939	0.634552	0.898417
Novel:hsa-miR-4277	-0.41363	1.095936	0.225296	0.635034	0.898417
Novel:NC_037549.1_15917	-0.23469	3.850405	0.220388	0.638745	0.899251
bta-miR-2285co	-0.11863	6.94187	0.220172	0.638909	0.899251
Novel:NC_037559.1_34015	-0.49897	1.668085	0.219171	0.639672	0.899251
Novel:NC_037569.1_46992	-0.17406	5.252967	0.219015	0.639792	0.899251
bta-miR-7863	-0.59725	1.106877	0.216947	0.641376	0.899825
bta-miR-32	-0.14467	6.082342	0.21543	0.642545	0.899825
Novel:hsa-miR-4695-5p	0.58447	1.02177	0.214416	0.643328	0.899825
Novel:NC_037558.1_32412	-0.13191	6.308303	0.212922	0.644487	0.899988
bta-miR-29a	-0.17039	9.854879	0.208249	0.648144	0.902955
Novel:hsa-miR-3064-5p	0.491945	1.003008	0.207537	0.648704	0.902955
Novel:NC_037553.1_23200	-0.23555	2.814108	0.199294	0.655291	0.907103
bta-miR-12030	-0.21873	3.691706	0.199278	0.655304	0.907103
bta-miR-6119-3p	0.539354	0.937869	0.197874	0.656442	0.907103
bta-miR-2284x	0.137258	10.63668	0.197214	0.656979	0.907103
bta-miR-214	0.165431	4.355541	0.196281	0.65774	0.907103
Novel:hsa-miR-4501	0.678063	1.59164	0.195136	0.658676	0.907103
Novel:chi-let-7i-3p	-0.69227	0.911374	0.194689	0.659043	0.907103
bta-miR-2483-3p	-0.15068	5.487258	0.18972	0.66315	0.90972
Novel:NC_037558.1_32071	-0.15475	5.097647	0.188834	0.663888	0.90972
bta-miR-215	-0.17008	6.513891	0.188574	0.664106	0.90972
bta-miR-2285cs	-0.24837	1.761962	0.186825	0.665572	0.910283
bta-miR-2483-5p	-0.3959	1.583748	0.182803	0.668975	0.91349
bta-miR-99a-3p	-0.15294	4.954385	0.181019	0.670499	0.914082
bta-miR-504	-0.25535	2.442545	0.179821	0.671527	0.914082
Novel:hsa-miR-2110	-0.47944	1.382946	0.173672	0.676869	0.917871
Novel:NC_037549.1_15077	0.122255	8.096657	0.171679	0.678624	0.917871
Novel:hsa-miR-2355-5p	-0.59812	0.893226	0.171299	0.67896	0.917871
bta-miR-671	-0.21159	2.569564	0.170798	0.679403	0.917871
Novel:NC_037549.1_16300	-0.14728	7.855003	0.168991	0.68101	0.917871
Novel:NC_037564.1_40643	-0.31087	1.66052	0.167842	0.682037	0.917871
bta-miR-677	0.161471	5.57206	0.166496	0.683245	0.917871
bta-miR-2285ad	-0.45886	0.998264	0.165893	0.683788	0.917871
bta-miR-2285aa	-0.20438	2.773905	0.164929	0.684658	0.917871
Novel:NC_037550.1_17666	-0.14364	4.964299	0.16461	0.684947	0.917871
Novel:NC_037562.1_38088	-0.55702	1.046836	0.157288	0.691665	0.918153

bta-miR-365-5p	-0.57205	0.789136	0.15427	0.694488	0.918153
bta-miR-2899	0.292823	2.1299	0.152711	0.695958	0.918153
bta-miR-374a	-0.27524	2.297745	0.152014	0.696618	0.918153
Novel:chi-miR-345-5p	0.470621	1.279476	0.150542	0.698018	0.918153
Novel:hsa-miR-4524a-3p	0.542781	1.276296	0.148936	0.699554	0.918153
bta-miR-208a	-0.49738	0.785803	0.148156	0.700304	0.918153
Novel:NC_037547.1_9455	-0.33936	1.565951	0.147779	0.700667	0.918153
bta-miR-495	-0.43882	0.976416	0.146775	0.701636	0.918153
bta-miR-2331-3p	0.34112	1.243513	0.146525	0.701879	0.918153
Novel:NC_037562.1_38773	-0.11017	12.6069	0.145873	0.70251	0.918153
bta-miR-106b	-0.13155	4.421944	0.143901	0.704434	0.918153
Novel:NC_037547.1_7586	0.466099	1.256215	0.142858	0.705456	0.918153
bta-miR-181a	0.110089	11.83234	0.142009	0.706292	0.918153
bta-miR-148d	-0.12471	10.94955	0.139466	0.708812	0.918153
Novel:NC_037547.1_7372	0.174012	2.729172	0.137341	0.710939	0.918153
Novel:NC_037546.1_4112	0.17405	2.729172	0.137236	0.711044	0.918153
Novel:NC_037546.1_2960	0.154983	3.31072	0.136317	0.71197	0.918153
bta-miR-21-3p	-0.12104	9.802458	0.135334	0.712964	0.918153
bta-miR-9851	0.272325	1.954663	0.134365	0.713948	0.918153
bta-miR-410	0.117695	7.398231	0.133669	0.714658	0.918153
bta-miR-2284c	-0.41264	1.473982	0.133541	0.714788	0.918153
bta-miR-10b	0.075397	18.95583	0.132245	0.716115	0.918153
Novel:NC_037549.1_15660	0.111671	10.83605	0.131498	0.716884	0.918153
Novel:hsa-miR-1265	0.14609	9.965939	0.131481	0.716901	0.918153
Novel:NC_037556.1_30106	0.111672	10.83605	0.131138	0.717255	0.918153
bta-miR-493	0.360876	1.652951	0.129315	0.719143	0.918153
bta-miR-2285aw	-0.43194	1.087387	0.129221	0.719241	0.918153
Novel:NC_037564.1_40358	-0.11501	6.280408	0.128313	0.720188	0.918153
Novel:NC_037547.1_10878	0.593114	0.986572	0.12753	0.721007	0.918153
Novel:NC_037560.1_35403	-0.31039	5.799896	0.126996	0.721567	0.918153
bta-miR-219b-3p	-0.4221	1.215093	0.126751	0.721825	0.918153
bta-miR-11986b	-0.35405	1.16033	0.126389	0.722206	0.918153
Novel:NC_037547.1_8774	0.575151	0.943879	0.126298	0.722302	0.918153
bta-miR-3120	-0.47406	1.299659	0.12595	0.722668	0.918153
bta-miR-2284ab	0.146663	4.770838	0.122977	0.725828	0.918153
bta-miR-146b	-0.18682	4.147388	0.121968	0.72691	0.918153
bta-miR-375	-0.12013	6.017894	0.121168	0.727772	0.918153
bta-miR-92b	-0.11659	11.07298	0.120143	0.728879	0.918153
bta-miR-3578	0.214885	2.840412	0.119965	0.729073	0.918153
Novel:hsa-miR-6839-3p	-0.12047	10.40604	0.119871	0.729174	0.918153
bta-miR-574	-0.1043	10.35824	0.117663	0.731583	0.918153
bta-miR-2285u	0.094945	7.173223	0.116531	0.732828	0.918153
Novel:NC_037568.1_46017	-0.20069	2.228924	0.115219	0.734279	0.918153
Novel:NC_037560.1_35659	-0.20071	2.228924	0.115118	0.734391	0.918153
bta-miR-323	-0.22592	1.969358	0.11472	0.734833	0.918153
bta-miR-140	-0.09853	12.50453	0.114426	0.735161	0.918153
Novel:hsa-miR-1908-3p	0.201508	2.386523	0.111633	0.738292	0.918621
bta-miR-2285y	0.212932	2.417304	0.110378	0.739714	0.918621
bta-miR-2285e	0.129772	3.919357	0.109919	0.740236	0.918621

bta-miR-192	-0.10344	10.25473	0.109578	0.740625	0.918621
bta-miR-541	0.218047	2.603791	0.10875	0.741572	0.918621
Novel:NC_037550.1_19148	-0.57506	0.792576	0.108444	0.741922	0.918621
bta-miR-15a	0.104462	6.2666	0.105049	0.745853	0.922165
Novel:hsa-miR-3157-5p	0.214995	2.513954	0.101752	0.749737	0.925507
Novel:NC_037549.1_16021	-0.21927	2.275545	0.100943	0.750701	0.925507
Novel:hsa-miR-3921	-0.47009	1.000691	0.098205	0.753995	0.925733
Novel:NC_037569.1_46177	0.301939	0.947274	0.095744	0.756997	0.925733
bta-miR-708	-0.40844	1.321974	0.095365	0.757464	0.925733
Novel:NC_037553.1_22911	-0.16202	4.31283	0.095117	0.75777	0.925733
Novel:NC_037546.1_3818	-0.38273	1.159198	0.094846	0.758105	0.925733
bta-miR-425-3p	-0.12085	4.322834	0.094504	0.758528	0.925733
Novel:NC_037547.1_7477	-0.44965	0.788652	0.094103	0.759024	0.925733
Novel:hsa-miR-6867-5p	0.121536	4.714151	0.093007	0.760389	0.925733
Novel:hsa-miR-219a-2-3p	-0.26958	3.511545	0.091997	0.761654	0.925733
bta-miR-6535	-0.37629	0.968189	0.091778	0.761929	0.925733
Novel:hsa-miR-548as-3p	0.126286	4.108145	0.091179	0.762684	0.925733
bta-miR-125a	0.174452	7.49183	0.087993	0.766744	0.929354
bta-miR-454	0.477369	0.874635	0.0857	0.769717	0.930902
bta-miR-551b	0.187317	1.991628	0.085346	0.770179	0.930902
Novel:NC_037556.1_30278	-0.48154	0.816636	0.084167	0.771728	0.931471
bta-miR-2898	-0.23343	1.398615	0.081733	0.774962	0.933427
Novel:chi-miR-493-3p	-0.41048	1.259717	0.081323	0.775512	0.933427
bta-miR-2887	0.136901	3.171637	0.080269	0.776933	0.933835
bta-miR-181b	-0.08599	9.261175	0.076976	0.781439	0.936742
Novel:hsa-miR-4443	-0.40582	0.734897	0.076813	0.781664	0.936742
bta-miR-30e-5p	-0.07942	10.83383	0.076132	0.782609	0.936742
bta-miR-190b	-0.13512	2.797322	0.074574	0.784789	0.937294
bta-miR-382	0.166857	2.821565	0.073393	0.786459	0.937294
bta-miR-502b	-0.12621	3.165444	0.072669	0.78749	0.937294
Novel:NC_037550.1_18976	-0.26853	0.909456	0.07197	0.78849	0.937294
Novel:hsa-miR-3908	0.095675	7.355681	0.071963	0.7885	0.937294
bta-miR-665	-0.15103	3.026377	0.069112	0.792635	0.939843
bta-miR-2408	-0.20582	1.706895	0.068984	0.792823	0.939843
bta-miR-18a	0.276405	1.077586	0.065807	0.797542	0.940909
Novel:NC_037548.1_13083	0.10765	4.818678	0.065408	0.798145	0.940909
Novel:NC_037555.1_28111	-0.1084	3.257487	0.065147	0.798538	0.940909
Novel:NC_037549.1_14328	-0.24723	1.462391	0.064841	0.799002	0.940909
Novel:hsa-miR-4743-5p	0.1583	2.687853	0.064053	0.800201	0.940909
Novel:NC_037561.1_37162	-0.12045	2.612221	0.061649	0.803909	0.940909
bta-miR-2285ak-5p	0.235724	1.144991	0.061609	0.80397	0.940909
Novel:hsa-miR-208a-5p	0.252701	1.935868	0.061435	0.804241	0.940909
bta-miR-331-3p	0.15895	1.959664	0.061298	0.804457	0.940909
Novel:hsa-miR-3683	0.40486	1.230058	0.060186	0.806202	0.940909
Novel:NC_037547.1_8236	-0.35377	1.471546	0.059409	0.807432	0.940909
bta-miR-27b	-0.06773	13.44362	0.05903	0.808036	0.940909
Novel:hsa-miR-3126-5p	-0.40437	0.77419	0.05851	0.808867	0.940909
Novel:chi-miR-9-5p	0.289425	1.449745	0.058436	0.808985	0.940909
bta-miR-425-5p	0.084915	5.57747	0.056408	0.812265	0.943412

Novel:hsa-miR-5088-5p	-0.3882	1.047017	0.055762	0.813324	0.943412
bta-miR-153	0.200132	1.731605	0.05493	0.814697	0.943736
bta-miR-2415-3p	0.219791	1.211592	0.053288	0.817437	0.944359
Novel:NC_037549.1_17067	0.119993	5.224977	0.052861	0.818158	0.944359
bta-miR-296-3p	-0.07097	10.54605	0.052648	0.818518	0.944359
Novel:NC_037547.1_10096	-0.26113	1.362269	0.049665	0.823648	0.94477
Novel:NC_037564.1_41393	-0.21733	1.292603	0.049656	0.823663	0.94477
bta-miR-3601	0.144096	2.300969	0.04963	0.823708	0.94477
Novel:NC_037548.1_12850	-0.09432	9.350961	0.049464	0.823999	0.94477
bta-miR-34a	0.121913	2.325489	0.048666	0.8254	0.94477
Novel:NC_037567.1_44300	-0.10201	2.976318	0.047782	0.826969	0.94477
bta-miR-491	-0.25051	0.859034	0.047406	0.82764	0.94477
Novel:NC_037568.1_44803	-0.10478	4.939648	0.046886	0.828574	0.94477
Novel:NC_037569.1_48122	0.32771	0.898578	0.046801	0.828727	0.94477
bta-miR-2889	-0.08046	6.483488	0.045013	0.831979	0.945976
Novel:NC_037561.1_36265	-0.11855	3.871647	0.044155	0.833566	0.945976
bta-miR-29d-3p	0.12036	2.151772	0.044063	0.833737	0.945976
bta-miR-455-3p	-0.22399	1.540458	0.043831	0.834168	0.945976
bta-miR-12031	0.299562	0.911973	0.04285	0.836007	0.946332
Novel:hsa-miR-548c-3p	0.157758	1.57321	0.042497	0.836676	0.946332
bta-miR-505	-0.08878	3.851986	0.040193	0.841104	0.947359
Novel:hsa-miR-135a-3p	0.298145	0.691584	0.039521	0.84242	0.947359
Novel:NC_037557.1_30641	0.092559	3.763343	0.039241	0.842971	0.947359
bta-miR-2284m	0.194712	1.60308	0.038994	0.843461	0.947359
bta-miR-20a	0.091442	3.754498	0.03855	0.844343	0.947359
Novel:hsa-miR-1827	-0.26593	0.865374	0.037717	0.846012	0.947359
Novel:NC_037553.1_23290	-0.12767	1.754238	0.037163	0.847134	0.947359
bta-miR-450a	-0.19413	1.399823	0.036816	0.847841	0.947359
bta-miR-196b	-0.15443	1.68779	0.036098	0.849314	0.947359
Novel:NC_037569.1_46559	-0.05912	9.139057	0.035816	0.849896	0.947359
Novel:NC_037551.1_19765	-0.06923	4.143875	0.034878	0.851852	0.947359
Novel:NC_037546.1_5830	-0.06928	4.143875	0.034854	0.851901	0.947359
bta-miR-455-5p	0.104094	3.34119	0.03476	0.852099	0.947359
bta-miR-2338	0.312677	0.595361	0.034356	0.852952	0.947359
bta-miR-2285bl	-0.0659	4.461792	0.032681	0.856541	0.950122
bta-miR-17-5p	0.073607	4.335585	0.031892	0.858264	0.950449
Novel:NC_037545.1_137	-0.17423	1.328284	0.031504	0.859121	0.950449
bta-miR-19a	-0.05972	7.423665	0.031045	0.86014	0.950449
bta-miR-149-5p	-0.08869	4.43387	0.02948	0.863676	0.951996
Novel:NC_037553.1_24045	0.155997	0.9461	0.028922	0.86496	0.951996
Novel:hsa-miR-3144-5p	-0.13902	1.17769	0.02884	0.865149	0.951996
bta-miR-423-5p	-0.05363	13.68636	0.028191	0.866661	0.951996
bta-miR-2285o	-0.12668	1.719167	0.027851	0.86746	0.951996
bta-miR-2285dh	-0.07216	3.174981	0.027555	0.868158	0.951996
Novel:hsa-miR-12136	-0.06039	4.113896	0.02702	0.869434	0.952184
bta-miR-199a-5p	0.067324	4.763097	0.025476	0.873187	0.955083
Novel:hsa-miR-6071	0.160558	0.858995	0.023569	0.877988	0.956199
Novel:NC_037558.1_32697	-0.15939	1.106723	0.023478	0.878221	0.956199
Novel:NC_037546.1_4402	0.132723	1.906094	0.02307	0.879275	0.956199

Novel:NC_037545.1_1635	0.093064	2.6276	0.023003	0.879448	0.956199
Novel:hsa-miR-21-3p	0.22267	1.188161	0.022889	0.879747	0.956199
Novel:NC_037563.1_39878	-0.07422	14.84224	0.021883	0.882398	0.957874
bta-miR-301b	0.149204	1.276339	0.020865	0.885148	0.959612
Novel:NC_037558.1_32847	0.064352	4.796986	0.019913	0.88778	0.959612
Novel:NC_037564.1_40367	-0.07967	2.991083	0.019668	0.888468	0.959612
bta-miR-532	-0.04265	9.062841	0.018663	0.891337	0.959612
Novel:NC_037548.1_13397	0.046345	4.588966	0.018217	0.892635	0.959612
Novel:chi-miR-103-3p	0.058185	3.733795	0.018215	0.892641	0.959612
Novel:NC_037557.1_30647	0.055338	4.290206	0.017894	0.893586	0.959612
Novel:NC_037557.1_30978	0.085525	1.704488	0.01749	0.894785	0.959612
Novel:NC_037545.1_698	-0.17901	0.752534	0.017421	0.894994	0.959612
bta-miR-2285cv	-0.12557	1.376812	0.017379	0.895119	0.959612
Novel:NC_037563.1_39396	-0.06421	14.85696	0.016833	0.89677	0.960039
Novel:hsa-miR-6131	-0.04434	6.090493	0.016516	0.897743	0.960039
Novel:NC_037547.1_9757	0.074614	2.630971	0.016048	0.899195	0.960402
Novel:NC_037569.1_47935	-0.05031	3.774532	0.013607	0.907137	0.966535
Novel:NC_037547.1_7667	-0.13379	1.328601	0.013596	0.907177	0.966535
bta-miR-3431	0.049056	3.27247	0.010187	0.919605	0.978378
Novel:NC_037552.1_21749	-0.11472	1.522452	0.009946	0.920559	0.978378
bta-miR-542-5p	0.06401	2.216362	0.009275	0.923277	0.978447
Novel:NC_037545.1_431	0.027662	8.09395	0.008882	0.924917	0.978447
Novel:NC_037547.1_8573	0.027666	8.09395	0.008871	0.92496	0.978447
Novel:NC_037555.1_26610	0.091176	1.536781	0.008824	0.925159	0.978447
Novel:NC_037561.1_36221	0.055781	1.892895	0.008387	0.927032	0.979227
bta-miR-122	-0.12825	1.100313	0.007702	0.930067	0.981232
Novel:NC_037561.1_36492	0.041232	2.673972	0.007392	0.931485	0.981475
bta-miR-424-3p	-0.03356	4.562637	0.007159	0.932572	0.981475
bta-miR-380-3p	0.034422	4.607308	0.006246	0.937006	0.98446
Novel:chi-miR-103-5p	0.065274	1.241955	0.005872	0.938917	0.98446
Novel:NC_037557.1_30644	-0.02829	7.12007	0.005839	0.939091	0.98446
Novel:NC_037569.1_48203	-0.08414	1.414182	0.005671	0.939971	0.98446
bta-miR-1291	0.069579	0.926239	0.004395	0.947145	0.987367
bta-miR-148a	-0.01603	13.3992	0.004393	0.947157	0.987367
bta-miR-29b	-0.05284	2.462837	0.004262	0.947949	0.987367
Novel:hsa-miR-3620-5p	0.058933	1.230852	0.004246	0.948043	0.987367
Novel:hsa-miR-6499-5p	-0.02924	3.732762	0.004177	0.948467	0.987367
bta-miR-2285aj-5p	-0.01988	9.049187	0.003778	0.950989	0.987448
bta-miR-2284aa	0.046709	1.670009	0.003324	0.954027	0.987448
Novel:chi-miR-326-3p	0.051831	1.232066	0.003245	0.954576	0.987448
Novel:NC_037547.1_10635	0.016619	12.99098	0.003118	0.955468	0.987448
Novel:NC_037545.1_1554	-0.02366	3.629419	0.003118	0.955472	0.987448
Novel:chi-miR-202-3p	0.018002	10.59317	0.002852	0.957412	0.987448
Novel:NC_037569.1_47107	-0.02028	5.425615	0.002601	0.959325	0.987448
Novel:NC_037567.1_44728	-0.02369	2.959116	0.002371	0.961163	0.987448
Novel:NC_037560.1_34839	-0.02804	2.160878	0.002154	0.962979	0.987448
Novel:NC_037546.1_6618	-0.03777	1.823106	0.002015	0.964194	0.987448
Novel:hsa-miR-1915-3p	0.053732	1.586562	0.001954	0.964745	0.987448
bta-miR-136	-0.02938	2.242731	0.001897	0.965261	0.987448

bta-miR-30f	-0.03047	2.531629	0.001788	0.966273	0.987448
bta-miR-769	-0.01402	5.776153	0.001783	0.966323	0.987448
Novel:NC_037569.1_47135	0.044837	1.284292	0.001768	0.966458	0.987448
bta-miR-2284j	-0.02889	1.816154	0.001641	0.96769	0.987448
Novel:NC_037546.1_4552	0.036123	1.874611	0.001471	0.969408	0.987448
bta-miR-1271	-0.0326	2.921992	0.001427	0.969871	0.987448
bta-miR-204	-0.01695	4.35936	0.001372	0.970456	0.987448
Novel:NC_037569.1_47783	0.06292	0.623521	0.001283	0.971429	0.987448
Novel:hsa-miR-4691-3p	0.014785	3.734882	0.001007	0.974688	0.989595
bta-miR-6119-5p	-0.01204	5.409071	0.000891	0.976184	0.989949
bta-miR-11987	-0.01341	5.298746	0.000581	0.980761	0.991769
Novel:NC_037561.1_36929	0.022164	1.192099	0.000551	0.981279	0.991769
Novel:hsa-miR-6748-3p	0.015942	2.003081	0.000542	0.981426	0.991769
bta-miR-125b	-0.00948	7.327796	0.000393	0.984186	0.993394
Novel:NC_037553.1_23265	-0.01509	2.914559	0.000303	0.98612	0.994146
Novel:NC_037565.1_41728	0.040951	0.895582	0.000256	0.987235	0.994146
bta-miR-885	0.018954	0.982974	0.000199	0.988737	0.994499
Novel:chi-miR-34a	0.006994	1.008203	0.000123	0.991138	0.995754
Novel:hsa-miR-6755-5p	0.008119	1.344548	5.39E-05	0.994142	0.995959
Novel:NC_037546.1_3432	-0.00322	1.994627	5.00E-05	0.994358	0.995959
bta-miR-2285ae	-0.01003	1.020536	4.24E-05	0.994805	0.995959
bta-miR-767	0.00635	1.223238	9.83E-06	0.997498	0.997498

**pO(NBS_vs_BS)**	**logFC**	**logCPM**	**LR**	**PValue**	**FDR**
Novel:NC_037560.1_35403	5.305336	5.397052	22.20597	2.45E-06	0.001756
Novel:NC_037567.1_44155	-5.22006	3.508735	16.35464	5.25E-05	0.015636
bta-let-7f	-1.35896	10.465	15.93885	6.54E-05	0.015636
Novel:NC_037564.1_40326	-1.92616	7.20017	14.91427	1.13E-04	0.016223
bta-miR-381	-1.93361	7.124194	14.90382	1.13E-04	0.016223
Novel:hsa-miR-3714	-2.1158	10.14229	12.31906	4.48E-04	0.043385
Novel:hsa-miR-6077	-3.90686	3.612931	12.19471	0.000479	0.043385
Novel:hsa-miR-4515	1.911588	6.17097	12.09971	0.000504	0.043385
Novel:NC_037552.1_22617	-2.56358	4.137116	11.79189	0.000595	0.043385
Novel:NC_037569.1_46660	-2.09775	5.481357	11.65896	0.000639	0.043385
bta-miR-487b	-4.61872	3.224257	11.58291	0.000666	0.043385
Novel:hsa-miR-4428	-2.2262	6.42771	11.24989	0.000796	0.047578
Novel:NC_037548.1_12850	2.78736	6.071088	10.93288	0.000945	0.052105
bta-miR-191	0.995604	13.00523	10.67818	0.001084	0.05552
bta-miR-324	4.764381	3.20758	10.42793	0.001241	0.059331
Novel:hsa-miR-6734-3p	-1.70254	7.69979	9.183966	0.002441	0.109407
bta-let-7a-5p	-1.17755	9.871325	8.946203	0.00278	0.111842
Novel:NC_037561.1_36265	-2.00312	4.957709	8.821679	0.002977	0.111842
bta-miR-2898	4.100571	2.997816	8.70969	0.003165	0.111842
Novel:hsa-miR-548u	-4.2074	3.040487	8.672773	0.00323	0.111842
bta-miR-454	4.64503	3.167076	8.647159	0.003276	0.111842
Novel:hsa-miR-185-3p	3.638028	3.300884	8.313265	0.003936	0.128267
Novel:NC_037545.1_558	-3.08988	3.641711	7.97643	0.004739	0.147734
bta-miR-652	1.096108	6.460656	7.429288	0.006417	0.19171
Novel:hsa-miR-663a	-1.33436	10.31251	7.136265	0.007554	0.21665
Novel:NC_037549.1_15945	3.948973	2.936089	6.845086	0.008889	0.223846

Novel:NC_037564.1_40932	-3.99512	2.992059	6.785411	0.009191	0.223846
Novel:chi-miR-24-5p	-1.11081	5.888245	6.717508	0.009547	0.223846
Novel:hsa-miR-652-5p	1.241706	5.646437	6.678362	0.009759	0.223846
Novel:NC_037562.1_38397	-2.6081	4.205316	6.627526	0.010041	0.223846
bta-miR-194b	4.350671	2.991657	6.479084	0.010915	0.223846
Novel:NC_037557.1_30571	4.28404	3.008351	6.438571	0.011167	0.223846
Novel:hsa-miR-6777-5p	-1.04928	7.039588	6.429308	0.011225	0.223846
Novel:NC_037563.1_39679	-1.25624	5.922075	6.419054	0.01129	0.223846
Novel:NC_037548.1_11079	-1.31257	9.338391	6.316483	0.011962	0.223846
Novel:NC_037547.1_10839	-3.93395	2.963932	6.25524	0.012383	0.223846
Novel:hsa-miR-4434	-3.79549	2.915686	6.239045	0.012496	0.223846
Novel:NC_037553.1_23471	3.479805	2.791035	6.203251	0.012752	0.223846
Novel:NC_037569.1_46587	-1.33061	5.643767	6.191039	0.01284	0.223846
bta-miR-1246	1.370387	9.108559	6.184249	0.012889	0.223846
Novel:NC_037565.1_41975	-3.81969	2.92142	6.104129	0.013487	0.223846
bta-miR-130a	-0.7352	11.7405	6.073613	0.013722	0.223846
bta-miR-2285br	3.055093	3.098393	6.057418	0.013848	0.223846
Novel:NC_037547.1_10260	-4.02764	4.780584	6.050277	0.013904	0.223846
Novel:NC_037547.1_9353	1.754045	4.558668	6.031983	0.014049	0.223846
Novel:NC_037555.1_27691	-3.78775	2.892564	5.830297	0.015752	0.238968
Novel:hsa-miR-3129-3p	1.855053	8.104884	5.823577	0.015813	0.238968
Novel:hsa-miR-1265	-0.98953	11.87027	5.802259	0.016006	0.238968
bta-miR-369-3p	-3.18063	3.246727	5.766862	0.016331	0.238968
bta-miR-12030	2.66415	3.23629	5.671505	0.017243	0.239376
bta-miR-148b	1.02156	8.235108	5.632416	0.017631	0.239376
Novel:hsa-miR-203a-5p	-2.70818	3.732141	5.631459	0.017641	0.239376
bta-miR-205	1.247233	6.184753	5.626152	0.017694	0.239376
Novel:NC_037550.1_18908	0.786484	10.10389	5.401007	0.020125	0.258541
bta-miR-2284y	-1.6159	4.440373	5.395682	0.020187	0.258541
bta-miR-186	0.777506	10.12398	5.348146	0.020744	0.258541
bta-miR-143	-1.05735	13.56188	5.33587	0.020891	0.258541
Novel:NC_037553.1_23783	-1.12634	6.159782	5.328598	0.020978	0.258541
bta-miR-873	-1.62556	5.838789	5.276595	0.021614	0.258541
bta-miR-328	1.344109	5.324639	5.274887	0.021635	0.258541
bta-miR-6520	-2.1599	4.349035	5.235318	0.022133	0.258701
Novel:hsa-miR-4482-3p	-1.40748	4.770885	5.216758	0.02237	0.258701
Novel:NC_037556.1_28412	-1.27298	6.487915	5.12261	0.023616	0.268773
Novel:chi-miR-2331	-2.91994	3.330474	5.016788	0.025103	0.281229
bta-miR-2285aa	-3.6834	2.823166	4.982682	0.025602	0.282413
bta-miR-2285f	1.628917	3.790432	4.936256	0.026299	0.282606
bta-miR-23a	0.952323	6.799241	4.922658	0.026507	0.282606
bta-miR-660	-0.69008	10.81039	4.865674	0.027396	0.282606
Novel:NC_037567.1_44092	1.267071	8.816531	4.856082	0.027549	0.282606
bta-miR-2285av	-3.09892	2.718389	4.823556	0.028073	0.282606
Novel:NC_037569.1_46549	-0.68905	10.80689	4.80761	0.028334	0.282606
Novel:NC_037556.1_29648	-3.30928	2.772469	4.803836	0.028396	0.282606
Novel:NC_037569.1_48203	3.137299	2.715159	4.780378	0.028786	0.282606
Novel:hsa-miR-7113-3p	1.50039	8.902213	4.742085	0.029433	0.282606
bta-miR-2440	-2.40589	3.622264	4.698967	0.030181	0.282606

bta-miR-486	-0.98822	11.23698	4.695543	0.030241	0.282606
bta-miR-2285cm	3.233591	2.733319	4.684147	0.030442	0.282606
bta-miR-409b	-1.1095	8.603096	4.640364	0.031228	0.282606
bta-miR-339a	0.849952	7.770885	4.62531	0.031504	0.282606
Novel:NC_037546.1_4370	1.225019	8.941059	4.613361	0.031724	0.282606
bta-miR-500	3.066053	2.705381	4.602453	0.031926	0.282606
bta-miR-101	-0.95053	8.674757	4.540868	0.033095	0.289378
bta-let-7e	-0.89303	5.87773	4.500797	0.033879	0.289732
Novel:NC_037553.1_23265	1.478337	4.276503	4.484626	0.034201	0.289732
Novel:NC_037553.1_24374	-1.41178	4.586343	4.47732	0.034348	0.289732
Novel:NC_037549.1_16572	-3.49895	2.768099	4.435739	0.035194	0.291243
bta-miR-125a	1.400975	7.48631	4.424605	0.035424	0.291243
Novel:NC_037553.1_22994	-0.98436	13.26997	4.409211	0.035745	0.291243
bta-miR-28	1.280753	4.742555	4.372352	0.036527	0.292533
Novel:NC_037545.1_137	3.029649	2.682493	4.36337	0.03672	0.292533
Novel:NC_037547.1_8560	-0.82705	6.597469	4.182239	0.04085	0.309705
Novel:NC_037549.1_15054	-3.04471	2.69171	4.173676	0.041057	0.309705
Novel:chi-let-7i-3p	2.918876	3.273126	4.170744	0.041128	0.309705
Novel:NC_037545.1_2499	-0.81837	12.61704	4.167692	0.041202	0.309705
Novel:hsa-miR-6770-5p	-3.12308	3.224826	4.15944	0.041403	0.309705
Novel:NC_037561.1_36258	-1.69462	3.973876	4.142898	0.04181	0.309705
bta-miR-23b-3p	1.061751	5.187666	4.139293	0.041899	0.309705
Novel:hsa-miR-21-3p	2.399415	2.894067	4.099449	0.042897	0.311754
bta-miR-122	2.399759	2.894067	4.093611	0.043046	0.311754
Novel:hsa-miR-6125	1.213754	5.232909	4.048873	0.044201	0.31692
Novel:NC_037549.1_17067	-1.07523	6.059324	3.974579	0.046192	0.323317
bta-miR-410	-0.9509	7.668837	3.943276	0.047059	0.323317
Novel:hsa-miR-3144-5p	-2.96754	2.699233	3.938252	0.0472	0.323317
bta-miR-192	0.680307	11.85302	3.925034	0.047572	0.323317
Novel:NC_037546.1_6618	1.812091	3.390142	3.915342	0.047847	0.323317
bta-miR-219	2.228545	3.191873	3.914484	0.047872	0.323317
bta-miR-2881	-2.87999	2.670944	3.892454	0.048504	0.323317
Novel:NC_037548.1_12197	-1.57277	3.660074	3.885648	0.0487	0.323317
Novel:hsa-miR-3908	1.225027	5.229417	3.835317	0.050184	0.330106
Novel:hsa-miR-3960	2.784859	3.230004	3.762882	0.052402	0.340308
bta-miR-99b	0.651101	11.89702	3.739496	0.05314	0.340308
bta-miR-877	-0.9377	5.743587	3.738938	0.053158	0.340308
Novel:NC_037547.1_10537	-0.81077	6.605949	3.713539	0.053973	0.342465
bta-miR-669	0.616121	11.67758	3.653183	0.055962	0.351149
Novel:hsa-miR-6839-3p	1.440318	7.523328	3.64254	0.056321	0.351149
bta-miR-2889	-1.24116	6.432972	3.590628	0.058106	0.353648
Novel:NC_037549.1_16021	-1.81199	4.486844	3.589217	0.058156	0.353648
Novel:NC_037562.1_38494	2.72992	3.014599	3.56214	0.059111	0.353648
Novel:NC_037558.1_32628	-2.23295	3.169815	3.55082	0.059516	0.353648
bta-miR-21-5p	-0.62943	11.37885	3.527728	0.06035	0.353648
bta-miR-148c	0.78609	8.128086	3.52763	0.060354	0.353648
bta-miR-188	1.14707	4.281374	3.512599	0.060904	0.353648
Novel:chi-miR-214-3p	-2.66627	3.28974	3.511963	0.060927	0.353648
Novel:NC_037545.1_1192	-1.50484	3.579334	3.505624	0.061161	0.353648

Novel:hsa-miR-4537	2.643576	3.176001	3.429271	0.06405	0.367393
Novel:NC_037553.1_23290	1.661017	3.643149	3.401851	0.065123	0.370583
bta-miR-2904	1.310583	5.765651	3.380023	0.065991	0.372564
bta-miR-2285bl	-1.03644	4.456416	3.310885	0.068822	0.382871
bta-miR-1271	1.319012	4.26296	3.309399	0.068885	0.382871
bta-miR-11986b	2.553712	2.603805	3.289639	0.069718	0.384523
bta-miR-339b	0.716763	7.412268	3.226685	0.072447	0.396524
bta-miR-375	0.898741	8.054555	3.139288	0.076427	0.414635
Novel:hsa-miR-6741-5p	0.739203	7.227726	3.119996	0.077337	0.414635
Novel:hsa-miR-6124	-2.09971	4.401451	3.113238	0.077658	0.414635
Novel:hsa-miR-548d-3p	-2.53208	2.609033	3.10423	0.078089	0.414635
Novel:hsa-miR-1908-3p	2.091806	4.509936	3.080251	0.079248	0.414635
bta-miR-30a-5p	-0.51803	13.46186	3.072368	0.079633	0.414635
Novel:NC_037554.1_24951	-0.75439	6.529782	3.068884	0.079804	0.414635
Novel:NC_037547.1_7786	2.181345	3.201202	3.053971	0.08054	0.415447
Novel:NC_037565.1_42179	-1.77451	4.087109	3.04028	0.081222	0.415972
bta-let-7g	-0.79495	9.126382	2.995914	0.083475	0.416327
Novel:hsa-miR-548as-3p	-1.0544	4.810776	2.990541	0.083752	0.416327
bta-miR-197	0.790329	5.902051	2.981629	0.084214	0.416327
bta-miR-2887	-1.34141	4.012106	2.970153	0.084814	0.416327
Novel:NC_037547.1_8583	-1.03989	4.856198	2.969638	0.084841	0.416327
Novel:chi-miR-145-3p	-1.1629	4.592228	2.926884	0.087116	0.416327
bta-miR-26c	0.580547	11.80043	2.926556	0.087133	0.416327
bta-miR-665	-1.02399	4.771811	2.924493	0.087245	0.416327
Novel:NC_037550.1_18832	1.797214	3.424838	2.872885	0.090083	0.416327
bta-miR-26a	0.576514	11.68502	2.872358	0.090113	0.416327
bta-miR-2285bw	-1.37611	3.78118	2.868654	0.09032	0.416327
Novel:NC_037565.1_42672	-0.77339	9.17208	2.866755	0.090427	0.416327
Novel:hsa-miR-4707-5p	2.343567	2.929803	2.858029	0.090919	0.416327
Novel:NC_037545.1_1635	-1.34418	3.682503	2.852073	0.091256	0.416327
bta-miR-1388-3p	0.79216	5.514911	2.837045	0.092113	0.416327
bta-miR-152	0.678005	7.967384	2.836504	0.092145	0.416327
Novel:hsa-miR-6818-3p	-2.16536	3.084204	2.821986	0.092981	0.416327
bta-miR-2468	-2.3408	3.022528	2.809322	0.093718	0.416327
bta-miR-2890	-0.75822	6.096583	2.808978	0.093738	0.416327
bta-miR-2446	-1.91651	3.350731	2.805052	0.093968	0.416327
Novel:NC_037569.1_46557	1.049709	4.36588	2.802673	0.094107	0.416327
bta-miR-361	0.871171	7.168105	2.796639	0.094462	0.416327
bta-miR-181b	-0.60521	10.07433	2.793521	0.094646	0.416327
Novel:NC_037567.1_44300	2.198288	3.426625	2.70628	0.099954	0.435186
bta-miR-10174-3p	0.675089	5.881886	2.700379	0.100324	0.435186
Novel:NC_037566.1_43265	-1.58859	3.112267	2.685762	0.101249	0.435186
Novel:NC_037546.1_4552	1.517044	3.467692	2.679318	0.101659	0.435186
bta-miR-342	0.645075	7.769487	2.665654	0.102536	0.435186
Novel:NC_037547.1_9757	0.527953	9.649412	2.665039	0.102575	0.435186
Novel:NC_037564.1_40729	-0.61844	8.471605	2.624367	0.105234	0.443842
Novel:NC_037569.1_46466	0.614043	9.206144	2.610386	0.106166	0.445152
Novel:hsa-miR-1290	1.593212	4.094367	2.566273	0.109165	0.454222
bta-miR-10b	-0.46603	18.35182	2.560037	0.109596	0.454222

Novel:NC_037562.1_38773	-0.49211	12.36813	2.546387	0.110547	0.455529
Novel:NC_037545.1_548	-1.73122	3.404826	2.537195	0.111192	0.45557
Novel:NC_037549.1_16477	1.038268	6.896167	2.523203	0.112183	0.457016
Novel:NC_037548.1_12074	-0.71063	8.336208	2.485473	0.114902	0.461777
Novel:NC_037564.1_40367	-0.97703	4.660655	2.485023	0.114935	0.461777
Novel:NC_037567.1_44728	2.098209	3.385028	2.472388	0.115862	0.461777
bta-miR-140	-0.4875	12.27465	2.464436	0.116449	0.461777
Novel:hsa-miR-219a-2-3p	-2.10101	3.169372	2.462794	0.116571	0.461777
Novel:NC_037553.1_24410	2.141827	2.849773	2.452147	0.117364	0.462364
Novel:NC_037546.1_4112	-1.27874	3.485167	2.423316	0.119542	0.467844
Novel:NC_037547.1_7372	-1.27863	3.485167	2.416534	0.12006	0.467844
bta-miR-1839	-0.58319	8.682424	2.381264	0.122798	0.473034
Novel:chi-miR-3958-5p	0.80124	6.196689	2.376598	0.123165	0.473034
Novel:NC_037568.1_45368	-2.51766	3.144968	2.371943	0.123533	0.473034
Novel:NC_037569.1_46177	-2.14816	2.849207	2.365664	0.124031	0.473034
bta-miR-6529a	0.501039	9.742237	2.319137	0.127791	0.482249
Novel:NC_037547.1_9992	0.737247	6.982392	2.312367	0.128348	0.482249
bta-miR-671	1.20349	4.223391	2.306429	0.12884	0.482249
bta-miR-6119-5p	1.415154	4.13987	2.30249	0.129167	0.482249
Novel:NC_037564.1_40573	-1.825	3.328787	2.288781	0.130312	0.482249
bta-miR-378	-0.5314	11.93273	2.286748	0.130483	0.482249
bta-miR-200a	-1.33282	3.80684	2.261137	0.132657	0.48568
bta-miR-6529b	0.48733	9.81573	2.25986	0.132766	0.48568
bta-miR-191b	1.340689	3.833083	2.25101	0.133527	0.485985
Novel:NC_037547.1_9224	-2.40508	2.894925	2.225376	0.13576	0.491614
Novel:hsa-miR-1237-3p	-0.52019	11.95965	2.2053	0.137537	0.493354
Novel:NC_037557.1_30688	-1.75165	3.22233	2.204413	0.137616	0.493354
bta-miR-141	0.947361	5.011159	2.155201	0.142088	0.506851
Novel:hsa-miR-4800-3p	-0.66441	6.594496	2.140944	0.143414	0.509047
bta-miR-30f	1.025705	4.011458	2.120163	0.145371	0.512397
Novel:hsa-miR-5190	-0.64026	6.617415	2.115793	0.145787	0.512397
Novel:NC_037549.1_15080	1.093188	4.668222	2.106533	0.146671	0.512992
Novel:NC_037564.1_41393	-2.09487	3.295362	2.093075	0.147968	0.513318
bta-miR-380-3p	0.870565	4.884076	2.08759	0.1485	0.513318
Novel:NC_037548.1_12436	-2.31518	2.879928	2.081856	0.149059	0.513318
Novel:hsa-miR-4632-3p	-1.82562	3.398918	2.076037	0.149628	0.513318
Novel:NC_037549.1_14559	-2.16599	2.853747	2.021791	0.155056	0.529406
Novel:NC_037548.1_11028	-1.85298	3.152537	1.998426	0.157463	0.533328
bta-miR-1247-5p	1.583437	3.609489	1.992723	0.158056	0.533328
bta-miR-221	0.535703	9.239718	1.989089	0.158436	0.533328
Novel:NC_037564.1_41423	-1.5391	2.972679	1.976105	0.159801	0.535409
Novel:hsa-miR-10392-3p	-1.42631	3.585116	1.958245	0.161701	0.539254
bta-miR-145	-0.79598	6.169116	1.927114	0.165075	0.547956
Novel:NC_037546.1_5554	-1.89667	2.883557	1.91514	0.166393	0.549789
bta-miR-2285ce	-1.08372	3.647378	1.891273	0.169058	0.556032
Novel:NC_037567.1_44107	-0.89243	4.453274	1.84125	0.174805	0.571061
Novel:chi-miR-22-5p	-1.58062	3.115835	1.834941	0.175545	0.571061
Novel:NC_037567.1_44553	-0.93057	4.435484	1.829944	0.176134	0.571061
Novel:NC_037547.1_7828	-1.47105	3.489095	1.815124	0.177895	0.571061

bta-miR-1260b	1.793039	3.22827	1.802415	0.179421	0.571061
Novel:hsa-miR-548a-3p	0.691182	5.473446	1.800401	0.179664	0.571061
Novel:NC_037552.1_21636	-2.2715	2.818051	1.797914	0.179965	0.571061
Novel:hsa-miR-7108-3p	0.52111	10.61506	1.797627	0.18	0.571061
Novel:NC_037564.1_40307	-1.12548	4.22359	1.790248	0.180896	0.571376
bta-miR-450a	-1.77989	3.26549	1.776356	0.182597	0.574221
bta-miR-99a-5p	0.675919	8.634291	1.736078	0.187636	0.582709
Novel:NC_037547.1_10102	-2.10232	2.975316	1.735195	0.187749	0.582709
Novel:NC_037569.1_47107	1.357177	4.107933	1.732646	0.188073	0.582709
bta-miR-11972	-1.02498	4.260842	1.728932	0.188547	0.582709
Novel:NC_037550.1_17666	0.82798	4.502188	1.720795	0.189591	0.583419
bta-miR-22-3p	0.384393	16.98608	1.694779	0.192972	0.587275
Novel:NC_037563.1_39389	-2.13459	2.962711	1.694419	0.193019	0.587275
Novel:hsa-miR-6715a-3p	0.576825	6.946747	1.691108	0.193455	0.587275
Novel:NC_037557.1_30647	0.84603	5.189181	1.685615	0.19418	0.587275
Novel:hsa-miR-198	1.157793	4.330476	1.673282	0.19582	0.587275
Novel:hsa-miR-6501-3p	-2.00185	2.824565	1.670514	0.19619	0.587275
Novel:NC_037558.1_31787	-2.00187	2.824565	1.667623	0.196577	0.587275
Novel:NC_037547.1_8540	-0.3949	12.98829	1.654861	0.198299	0.587982
bta-miR-3600	0.380174	17.013	1.651323	0.198779	0.587982
Novel:hsa-miR-759	1.422923	3.024438	1.633232	0.201257	0.587982
bta-miR-2892	0.740293	5.070683	1.630527	0.20163	0.587982
Novel:NC_037558.1_32790	-1.92815	2.796383	1.627474	0.202053	0.587982
Novel:hsa-miR-3150b-3p	-1.19133	3.709073	1.624255	0.202499	0.587982
bta-miR-11986c	-1.09979	3.709766	1.61969	0.203135	0.587982
bta-miR-29c	0.688466	5.018927	1.617469	0.203445	0.587982
bta-miR-2285aj-5p	-0.55046	8.81456	1.604153	0.205316	0.587982
bta-miR-296-5p	-1.8856	2.790239	1.592587	0.206957	0.587982
bta-miR-199a-5p	-1.11068	3.978772	1.588781	0.2075	0.587982
Novel:NC_037545.1_824	-0.51076	8.540742	1.587843	0.207635	0.587982
bta-miR-2285co	-0.51006	6.897288	1.583722	0.208225	0.587982
bta-miR-483	-1.40588	2.935743	1.583239	0.208295	0.587982
Novel:chi-miR-24-3p	-1.29586	4.103196	1.57715	0.209172	0.588141
Novel:hsa-miR-6784-3p	1.500524	3.399512	1.568091	0.210484	0.589521
Novel:NC_037566.1_43137	0.667689	5.317668	1.561008	0.211518	0.59011
Novel:chi-miR-22-3p	1.259251	3.139757	1.554837	0.212423	0.590146
Novel:hsa-miR-4475	-0.97439	5.001938	1.54487	0.213894	0.590146
Novel:hsa-miR-12135	-1.23274	3.301626	1.544158	0.214	0.590146
bta-miR-497	0.529946	6.869533	1.537019	0.215062	0.590803
bta-miR-374b	1.056416	3.911291	1.509713	0.219183	0.594808
bta-miR-2284x	-0.41095	10.32553	1.500826	0.220544	0.594808
Novel:NC_037569.1_47983	0.592977	5.541736	1.498544	0.220895	0.594808
Novel:NC_037564.1_40744	0.593008	5.541736	1.497438	0.221066	0.594808
bta-miR-17-5p	0.801596	5.164517	1.495414	0.221378	0.594808
Novel:NC_037557.1_30428	0.593085	5.541736	1.494643	0.221497	0.594808
bta-miR-431	-1.60431	2.778041	1.479606	0.223836	0.596198
Novel:NC_037560.1_35631	1.772359	3.107763	1.470985	0.22519	0.596198
bta-miR-31	0.512137	8.467622	1.465664	0.226031	0.596198
Novel:hsa-miR-6727-5p	-0.5738	10.5863	1.465485	0.226059	0.596198

Novel:NC_037558.1_32412	0.61254	5.677646	1.464188	0.226265	0.596198
Novel:NC_037547.1_10214	0.509309	8.644136	1.458363	0.227191	0.596198
Novel:NC_037557.1_31285	-1.93467	2.930998	1.454321	0.227836	0.596198
bta-miR-24-3p	0.396638	10.10736	1.446351	0.229114	0.597364
Novel:chi-miR-411b-5p	-1.10452	3.592642	1.412124	0.234704	0.60972
bta-miR-195	-0.66999	4.740115	1.401301	0.236506	0.610896
Novel:hsa-miR-3074-5p	0.390221	10.02227	1.389488	0.238492	0.610896
bta-miR-11977	0.623013	5.003825	1.389339	0.238517	0.610896
bta-miR-574	-0.45682	10.63639	1.375124	0.240933	0.610896
Novel:NC_037569.1_46961	0.57845	5.430806	1.374843	0.240982	0.610896
Novel:NC_037564.1_41452	0.578484	5.430806	1.373861	0.24115	0.610896
Novel:NC_037557.1_31142	0.578566	5.430806	1.371507	0.241553	0.610896
Novel:hsa-miR-2115-5p	-0.3337	13.68715	1.363853	0.242871	0.610896
bta-miR-425-5p	0.537479	5.948004	1.361568	0.243266	0.610896
Novel:hsa-miR-5589-5p	0.696298	4.411923	1.349679	0.245334	0.610896
Novel:NC_037554.1_25273	-1.79134	2.966248	1.342704	0.246558	0.610896
bta-miR-10182-5p	1.638627	2.751136	1.339057	0.2472	0.610896
bta-miR-20a	1.057754	4.830205	1.338969	0.247216	0.610896
bta-miR-181a	-0.31826	12.78448	1.334403	0.248023	0.610896
bta-miR-455-5p	-0.71487	4.715853	1.330392	0.248736	0.610896
bta-miR-1224	-1.14375	3.332992	1.324549	0.249777	0.610896
bta-miR-874	0.656764	5.007245	1.320675	0.250471	0.610896
Novel:NC_037557.1_30641	1.055956	4.857521	1.320552	0.250493	0.610896
bta-miR-125b	0.850852	6.755935	1.30927	0.252527	0.611087
bta-miR-320a	-0.35034	13.2646	1.305732	0.253169	0.611087
Novel:hsa-miR-4743-5p	-0.73321	4.79344	1.305219	0.253262	0.611087
bta-miR-126-5p	-0.66567	7.53012	1.301276	0.25398	0.611087
Novel:NC_037565.1_42130	1.301672	3.203034	1.285495	0.25688	0.613205
Novel:NC_037546.1_4218	-0.80826	4.277484	1.28144	0.257631	0.613205
Novel:NC_037558.1_32847	-0.64298	7.047405	1.269786	0.259807	0.613205
Novel:NC_037549.1_15660	-0.32329	11.78109	1.265713	0.260572	0.613205
Novel:NC_037566.1_43625	0.600735	5.288046	1.264818	0.260741	0.613205
Novel:NC_037560.1_35933	-0.68889	8.812223	1.264668	0.260769	0.613205
Novel:NC_037556.1_30106	-0.32329	11.78109	1.264254	0.260847	0.613205
bta-miR-12057	1.432424	2.859697	1.238774	0.265707	0.621126
bta-miR-484	0.423799	8.231201	1.237521	0.265949	0.621126
Novel:NC_037545.1_698	1.77174	2.877101	1.22884	0.267633	0.621216
Novel:NC_037564.1_40358	-0.48908	6.0126	1.224124	0.268553	0.621216
bta-miR-222	-0.65616	5.537571	1.223949	0.268587	0.621216
Novel:NC_037558.1_31842	-0.86415	3.599185	1.216393	0.27007	0.622636
Novel:chi-miR-671-5p	1.146411	3.135856	1.20536	0.272253	0.624198
Novel:hsa-miR-3185	-0.68237	8.752637	1.204178	0.272488	0.624198
bta-miR-30c	0.532814	5.95773	1.192408	0.274844	0.62571
Novel:NC_037549.1_16300	0.426551	12.47617	1.192163	0.274894	0.62571
Novel:hsa-miR-6781-5p	1.350161	2.899772	1.186	0.276138	0.626247
Novel:hsa-miR-545-3p	0.624822	6.342661	1.18198	0.276953	0.626247
bta-miR-365-3p	1.217746	3.961288	1.178068	0.27775	0.626247
Novel:hsa-miR-6888-5p	-1.38164	3.010306	1.16048	0.281366	0.630668
Novel:NC_037545.1_434	-0.63248	4.966308	1.156462	0.282201	0.630668

Novel:NC_037556.1_30288	-0.63272	7.595963	1.155709	0.282357	0.630668
bta-miR-10a	0.437327	9.901907	1.151531	0.283229	0.630668
bta-miR-450b	-0.55222	8.355127	1.14609	0.284369	0.631247
Novel:NC_037568.1_45446	1.726404	3.020534	1.136533	0.286386	0.633762
Novel:chi-miR-7-5p	-1.68023	2.738142	1.094289	0.295523	0.651969
Novel:NC_037546.1_4225	1.194347	3.253556	1.07952	0.298805	0.656768
Novel:hsa-miR-146b-3p	1.412025	2.986322	1.076285	0.29953	0.656768
bta-miR-541	-0.68016	4.746796	1.066121	0.301823	0.659778
Novel:hsa-miR-4723-5p	-0.51765	13.4365	1.045868	0.306461	0.665174
bta-miR-92a	0.29569	13.31417	1.045452	0.306557	0.665174
Novel:NC_037560.1_35426	0.493172	10.75793	1.043215	0.307075	0.665174
bta-let-7a-3p	0.706113	5.089677	1.034411	0.309125	0.667598
Novel:NC_037554.1_25895	-1.52848	2.889572	1.018022	0.312989	0.671423
Novel:NC_037557.1_30447	-1.49991	2.691066	1.007619	0.315474	0.671423
Novel:NC_037557.1_31157	-1.49991	2.691066	1.007619	0.315474	0.671423
Novel:NC_037569.1_47087	-0.63325	6.011097	1.005972	0.31587	0.671423
bta-miR-6123	0.541499	5.425748	1.003907	0.316367	0.671423
Novel:hsa-miR-3909	-1.17159	3.445665	1.003294	0.316515	0.671423
bta-miR-370	-1.60455	3.272466	0.961255	0.326871	0.691346
Novel:NC_037550.1_18131	-1.5209	2.79259	0.948683	0.330055	0.696027
bta-miR-677	0.888435	4.224178	0.942804	0.331558	0.697147
bta-miR-3578	-1.14207	3.675918	0.935445	0.333452	0.699079
Novel:hsa-miR-6849-3p	0.609373	4.652628	0.926454	0.335786	0.69991
bta-miR-19a	0.431657	6.68016	0.92343	0.336576	0.69991
bta-miR-18a	0.993742	3.277427	0.920327	0.337389	0.69991
Novel:NC_037547.1_9455	-1.05345	3.819945	0.917879	0.338032	0.69991
bta-miR-532	-0.3693	9.336	0.91038	0.340014	0.69991
bta-miR-301a	0.434717	7.122704	0.909097	0.340354	0.69991
Novel:NC_037549.1_16296	-1.3346	2.97083	0.907023	0.340906	0.69991
bta-miR-154c	-0.78647	4.576042	0.904107	0.341683	0.69991
Novel:NC_037552.1_21521	-1.537	3.060476	0.900552	0.342634	0.69991
bta-miR-424-5p	-0.59874	5.868586	0.894245	0.34433	0.701169
bta-miR-22-5p	0.584112	5.422575	0.889838	0.345521	0.701169
bta-miR-1468	0.543428	6.339336	0.887355	0.346195	0.701169
bta-miR-503-3p	-1.31516	3.332337	0.883372	0.34728	0.701169
Novel:NC_037545.1_431	-0.37294	7.787857	0.872058	0.350386	0.701169
bta-miR-126-3p	-1.3223	3.151022	0.86774	0.351582	0.701169
bta-let-7c	-0.37523	8.718593	0.866014	0.352061	0.701169
Novel:NC_037547.1_8573	-0.37304	7.787857	0.864575	0.352462	0.701169
Novel:hsa-miR-4479	1.333189	2.680233	0.86356	0.352744	0.701169
Novel:NC_037569.1_46653	-1.40808	2.852513	0.858371	0.354195	0.701169
Novel:chi-miR-485-5p	1.73629	3.028459	0.857034	0.35457	0.701169
bta-miR-326	-0.65309	3.875876	0.855557	0.354985	0.701169
Novel:NC_037545.1_503	0.476457	8.361795	0.846522	0.357538	0.702617
bta-miR-151-5p	0.416723	6.923533	0.845718	0.357767	0.702617
Novel:NC_037569.1_46468	-0.57814	5.367898	0.836794	0.360315	0.702617
Novel:NC_037550.1_18986	1.321791	2.705405	0.835885	0.360576	0.702617
Novel:NC_037549.1_16794	1.381325	2.776346	0.835741	0.360618	0.702617
Novel:NC_037550.1_17830	1.320847	2.705405	0.831549	0.361825	0.703058

Novel:NC_037569.1_46559	-0.34927	9.401025	0.817034	0.366049	0.709343
Novel:hsa-miR-548v	-0.56591	6.152542	0.807796	0.368773	0.712695
bta-miR-128	0.338879	8.90898	0.799646	0.371199	0.715125
Novel:chi-miR-1307-3p	0.401144	6.015258	0.794254	0.372817	0.715125
Novel:hsa-miR-10522-5p	-1.42543	3.023632	0.793466	0.373054	0.715125
Novel:NC_037555.1_27452	-1.03693	2.806417	0.78887	0.374442	0.715125
bta-miR-708	0.7066	5.180221	0.786974	0.375017	0.715125
bta-miR-382	-1.04086	3.698059	0.771054	0.379891	0.722498
Novel:hsa-miR-185-5p	-1.19315	3.080256	0.763348	0.382283	0.723557
bta-miR-202	-0.38181	9.63111	0.762761	0.382466	0.723557
bta-miR-411c-3p	-1.26251	3.282267	0.7552	0.384835	0.726123
Novel:NC_037547.1_9951	0.396588	7.126427	0.728993	0.39321	0.739978
bta-miR-424-3p	-0.40879	5.724875	0.717606	0.39693	0.743623
Novel:NC_037547.1_8180	-1.22089	2.656488	0.716722	0.397221	0.743623
bta-miR-215	0.338224	12.1944	0.712977	0.398457	0.743995
bta-miR-181c	0.611171	4.620899	0.705211	0.401038	0.746869
Novel:NC_037546.1_4277	-1.20414	2.653946	0.696588	0.403933	0.749469
bta-miR-2285y	-1.21363	2.654092	0.694833	0.404525	0.749469
Novel:oar-miR-10b	-0.72299	3.62691	0.689782	0.406238	0.750704
bta-miR-331-5p	0.42154	5.535788	0.682597	0.408694	0.7533
bta-miR-1343-3p	0.392663	5.731226	0.669248	0.413314	0.759861
bta-miR-495	1.125427	2.63225	0.658071	0.417242	0.765121
bta-miR-3956	-1.15416	2.647477	0.649268	0.420375	0.768899
Novel:hsa-miR-8077	-0.37	5.620783	0.641023	0.42334	0.770589
bta-miR-2285by	0.773027	3.443102	0.638869	0.42412	0.770589
Novel:NC_037564.1_40741	-1.24038	2.878341	0.636761	0.424886	0.770589
bta-miR-106b	0.371265	5.781991	0.634808	0.425597	0.770589
bta-miR-4449	-1.18655	2.897111	0.630053	0.427336	0.771164
bta-miR-92b	0.280983	12.78135	0.628066	0.428066	0.771164
bta-miR-485	-1.11921	2.661182	0.613772	0.433371	0.774821
bta-miR-19b	0.385862	8.985106	0.610759	0.434502	0.774821
bta-miR-331-3p	-1.16947	2.899163	0.609997	0.434789	0.774821
Novel:NC_037568.1_44803	0.602015	4.582841	0.608628	0.435305	0.774821
bta-miR-421	-0.32886	6.294756	0.601558	0.437984	0.774821
Novel:hsa-miR-505-3p	1.071871	2.70879	0.599644	0.438714	0.774821
Novel:NC_037553.1_23200	-0.63977	4.25309	0.597366	0.439585	0.774821
bta-miR-147	-0.98526	3.021056	0.597248	0.43963	0.774821
Novel:NC_037565.1_42322	-0.76514	3.687943	0.596747	0.439822	0.774821
bta-miR-30b-5p	-0.36537	5.52824	0.590445	0.442247	0.777184
bta-miR-504	0.866235	3.097007	0.573022	0.44906	0.780603
Novel:NC_037545.1_1554	-0.79659	3.307767	0.571713	0.449579	0.780603
bta-miR-502b	0.466196	4.186362	0.57065	0.450001	0.780603
Novel:hsa-miR-136-3p	-1.08809	2.724257	0.570379	0.450108	0.780603
bta-miR-12023	-0.92631	2.993229	0.568876	0.450706	0.780603
Novel:hsa-miR-6765-3p	0.927639	3.658717	0.565069	0.452225	0.780603
Novel:NC_037565.1_42254	0.654018	3.739128	0.563463	0.452868	0.780603
Novel:NC_037557.1_30978	-1.05716	2.653141	0.55877	0.454756	0.780603
bta-miR-151-3p	0.216823	12.14417	0.558172	0.454998	0.780603
bta-miR-2403	0.683683	3.454793	0.55289	0.457139	0.780603

Novel:NC_037569.1_46552	0.339937	7.033008	0.551913	0.457537	0.780603
Novel:NC_037565.1_42436	-1.04512	2.651569	0.548328	0.459002	0.780603
Novel:NC_037549.1_16548	0.933787	2.799875	0.547272	0.459435	0.780603
Novel:NC_037549.1_15024	0.933787	2.799875	0.547272	0.459435	0.780603
bta-miR-452	1.003966	3.633848	0.542396	0.461441	0.78216
bta-miR-129-5p	-0.36988	7.799369	0.52426	0.46903	0.785535
bta-miR-129	-0.36987	7.799369	0.523883	0.46919	0.785535
Novel:NC_037569.1_47783	-0.6925	3.299279	0.521137	0.470357	0.785535
Novel:NC_037552.1_22370	-0.35917	6.900179	0.520087	0.470805	0.785535
Novel:NC_037560.1_34765	-0.35916	6.900179	0.519825	0.470917	0.785535
Novel:NC_037558.1_31888	-0.76017	3.234877	0.51961	0.471008	0.785535
bta-miR-2285k	0.9735	2.646073	0.517717	0.471817	0.785535
Novel:NC_037567.1_43945	-1.16604	3.003445	0.516828	0.472198	0.785535
bta-miR-542-5p	-0.48886	4.458028	0.508969	0.475585	0.786791
bta-miR-2285u	-0.27869	6.998415	0.508619	0.475737	0.786791
Novel:hsa-miR-5580-5p	0.470529	5.041443	0.506084	0.476839	0.786791
Novel:NC_037553.1_24444	-1.02057	3.016135	0.504872	0.477367	0.786791
bta-miR-1296	-0.40795	5.214549	0.501854	0.478687	0.786791
Novel:NC_037548.1_12259	-0.4103	5.193753	0.499006	0.479937	0.786791
Novel:hsa-miR-4450	0.979454	2.922569	0.497425	0.480634	0.786791
Novel:NC_037549.1_15077	-0.27843	7.65831	0.490083	0.48389	0.790317
Novel:hsa-miR-4761-5p	-1.01757	2.717075	0.487475	0.485056	0.790421
Novel:NC_037546.1_3986	-0.81603	3.183823	0.48035	0.488264	0.791942
Novel:NC_037561.1_36931	-0.28496	6.56281	0.479177	0.488795	0.791942
bta-miR-182	0.431672	4.594835	0.475845	0.490311	0.791942
Novel:chi-miR-125a-5p	0.280779	6.096096	0.475631	0.490408	0.791942
bta-miR-6528	-0.86012	3.213869	0.466682	0.494517	0.795308
bta-miR-3601	-0.46414	4.523991	0.464464	0.495545	0.795308
bta-miR-12034	0.325598	5.655316	0.463311	0.496081	0.795308
bta-miR-2285s	-0.82184	3.37706	0.461489	0.496929	0.795308
bta-miR-148a	-0.19784	13.13645	0.443818	0.505285	0.806881
Novel:NC_037567.1_44073	0.230259	9.646417	0.436914	0.508615	0.808956
Novel:NC_037560.1_35724	0.730497	3.194144	0.433719	0.510169	0.808956
Novel:NC_037564.1_40393	-0.85558	3.085132	0.433184	0.510431	0.808956
Novel:hsa-miR-591	0.223281	9.89603	0.424707	0.514598	0.808956
bta-miR-425-3p	-0.36623	4.72322	0.424416	0.514742	0.808956
bta-miR-193b	-0.229	11.3466	0.42359	0.515151	0.808956
bta-miR-16a	0.394104	5.588834	0.422871	0.515508	0.808956
Novel:hsa-miR-339-5p	0.431186	4.181315	0.422665	0.51561	0.808956
Novel:NC_037561.1_37534	-0.38335	5.268922	0.41397	0.519961	0.809698
bta-miR-760-3p	0.265885	7.740336	0.412577	0.520664	0.809698
Novel:NC_037548.1_13397	-0.35521	4.544597	0.408123	0.522924	0.809698
Novel:hsa-miR-5193	-0.87263	2.667414	0.40743	0.523277	0.809698
bta-miR-11987	-0.40537	5.105488	0.40699	0.523501	0.809698
bta-miR-32	0.247556	6.79949	0.406572	0.523714	0.809698
Novel:hsa-miR-3672	0.260686	10.34134	0.406036	0.523989	0.809698
Novel:NC_037550.1_17628	0.264404	7.896838	0.403418	0.525329	0.810024
Novel:NC_037549.1_15917	0.380905	5.344783	0.396109	0.529106	0.814096
bta-miR-15a	0.302953	7.012604	0.389896	0.532354	0.81734

Novel:hsa-miR-3173-3p	0.913081	3.382596	0.386991	0.533885	0.81794
Novel:NC_037547.1_9101	0.36987	5.386131	0.38479	0.535051	0.817977
bta-miR-378b	-0.3124	5.471144	0.377859	0.538751	0.820205
bta-miR-493	0.493673	3.754044	0.377776	0.538796	0.820205
Novel:NC_037548.1_12165	-0.54283	3.729869	0.375268	0.540147	0.820519
bta-miR-29a	0.222018	9.323549	0.367197	0.544536	0.823951
bta-miR-545-5p	-0.82557	2.910358	0.3667	0.544808	0.823951
Novel:NC_037556.1_30323	0.677622	3.496247	0.364799	0.545853	0.823951
Novel:chi-miR-125a-3p	-0.92821	2.61332	0.360747	0.548091	0.824416
Novel:hsa-miR-765	-0.94846	2.880327	0.360081	0.548461	0.824416
Novel:chi-miR-491-5p	0.606775	3.447103	0.35686	0.550255	0.825383
bta-miR-33b	-0.8209	2.774505	0.35359	0.552088	0.826327
bta-miR-654	0.846382	2.94621	0.351634	0.55319	0.826327
bta-miR-193a	0.558958	3.829756	0.341673	0.558865	0.833069
Novel:hsa-miR-4663	0.807529	2.830914	0.338706	0.560577	0.833888
bta-miR-592	-0.76784	2.664632	0.332459	0.564215	0.834814
Novel:NC_037564.1_41171	-0.45881	4.773407	0.33225	0.564337	0.834814
Novel:hsa-miR-572	-0.90857	2.872808	0.331643	0.564693	0.834814
Novel:hsa-miR-3689d	-0.31879	11.39427	0.328423	0.566589	0.835835
bta-miR-132	0.179559	10.40544	0.32652	0.567715	0.835835
Novel:NC_037553.1_24219	0.901072	2.855474	0.321232	0.570868	0.83741
bta-miR-199a-3p	0.294017	6.959926	0.320811	0.571121	0.83741
bta-miR-103	-0.17551	12.80744	0.317589	0.57306	0.83773
Novel:NC_037550.1_18158	-0.26775	6.754273	0.315525	0.574309	0.83773
Novel:NC_037568.1_45248	-0.21089	10.95672	0.311863	0.576539	0.83773
Novel:NC_037558.1_32071	0.585426	3.813298	0.311683	0.576649	0.83773
bta-miR-26b	0.22618	7.307899	0.309964	0.577702	0.83773
bta-miR-362-3p	-0.62	3.54198	0.305182	0.580652	0.83773
bta-miR-148d	0.22391	8.809143	0.303447	0.58173	0.83773
Novel:NC_037558.1_32782	-0.26973	8.829166	0.300207	0.583753	0.83773
Novel:NC_037558.1_32781	-0.26973	8.829166	0.3	0.583883	0.83773
bta-miR-432	-0.29568	5.849581	0.299522	0.584182	0.83773
bta-miR-214	0.291116	5.195416	0.295538	0.586693	0.83773
Novel:NC_037547.1_7908	-0.82785	2.619139	0.295502	0.586716	0.83773
Novel:NC_037547.1_9916	-0.82785	2.619139	0.295502	0.586716	0.83773
bta-miR-409a	0.892146	3.112858	0.293121	0.588227	0.83773
Novel:NC_037556.1_29299	0.605865	3.469821	0.292121	0.588864	0.83773
Novel:hsa-miR-4691-3p	0.441184	4.541502	0.284949	0.593475	0.83932
bta-miR-2284ab	-0.27729	5.145855	0.284401	0.593831	0.83932
bta-miR-107	-0.17222	11.80272	0.284085	0.594036	0.83932
bta-miR-129-3p	0.664163	3.077191	0.283118	0.594665	0.83932
bta-miR-3596	-0.16225	10.86347	0.277045	0.598645	0.843277
bta-miR-2285e	-0.39994	3.673506	0.271646	0.602229	0.84578
Novel:NC_037563.1_39396	-0.20269	13.38846	0.270823	0.602781	0.84578
bta-miR-10164-3p	0.306365	5.48267	0.265969	0.606049	0.847132
Novel:NC_037564.1_40336	0.724961	2.580948	0.265026	0.606688	0.847132
Novel:hsa-miR-4675	0.416787	3.893227	0.262968	0.608088	0.847132
bta-miR-2285bz	0.263686	5.76973	0.259215	0.61066	0.847132
bta-miR-2483-5p	-0.48623	3.85615	0.257608	0.611768	0.847132

bta-miR-30d	0.15442	11.25577	0.251102	0.6163	0.847132
Novel:hsa-miR-1252-5p	0.704008	2.602588	0.250034	0.617051	0.847132
Novel:NC_037564.1_40376	0.247556	7.709525	0.24944	0.61747	0.847132
Novel:hsa-miR-208a-5p	0.472859	4.228427	0.249328	0.617549	0.847132
bta-miR-2285cr	-0.64792	2.97342	0.247935	0.618533	0.847132
Novel:NC_037568.1_46017	-0.43526	3.217451	0.247439	0.618884	0.847132
Novel:NC_037560.1_35659	-0.43525	3.217451	0.247024	0.619178	0.847132
bta-miR-345-3p	0.249133	7.621091	0.246001	0.619905	0.847132
bta-miR-330	-0.5278	3.631077	0.243488	0.621698	0.847132
Novel:NC_037569.1_47154	-0.25503	5.739431	0.242763	0.622217	0.847132
Novel:hsa-miR-6755-5p	-0.58237	2.956355	0.241415	0.623186	0.847132
Novel:NC_037555.1_27844	-0.43167	4.213968	0.240522	0.62383	0.847132
Novel:NC_037557.1_31699	0.311016	7.324913	0.232759	0.629486	0.849369
bta-miR-411a	-0.46631	4.649815	0.232057	0.630003	0.849369
bta-let-7i	-0.1664	10.62762	0.231971	0.630066	0.849369
Novel:hsa-miR-629-3p	-0.56418	3.176198	0.231769	0.630215	0.849369
bta-miR-21-3p	0.209119	10.98829	0.228942	0.632309	0.850592
Novel:NC_037546.1_6567	0.808117	3.014879	0.221939	0.637567	0.855942
bta-miR-1247-3p	-0.49534	3.669305	0.219976	0.639058	0.855942
Novel:hsa-miR-4753-5p	0.429948	3.371266	0.218916	0.639867	0.855942
bta-miR-27b	0.135441	14.39063	0.213042	0.644393	0.860391
Novel:hsa-miR-4442	0.696868	2.790918	0.208742	0.647755	0.862377
bta-miR-2285aw	-0.66094	2.583419	0.208067	0.648286	0.862377
bta-miR-3604	0.235273	7.617297	0.205482	0.650332	0.863496
bta-miR-2483-3p	0.195578	6.38314	0.203406	0.651986	0.864092
Novel:NC_037552.1_22398	0.587515	3.306044	0.19512	0.658689	0.868793
bta-miR-6517	-0.18019	6.186806	0.194543	0.659162	0.868793
Novel:NC_037557.1_30644	-0.26026	5.841146	0.194536	0.659168	0.868793
Novel:hsa-miR-6499-5p	0.366638	4.47455	0.191735	0.661477	0.869258
bta-miR-181d	-0.25717	5.374818	0.189509	0.663325	0.869258
Novel:NC_037569.1_46992	0.202737	6.29243	0.189344	0.663463	0.869258
bta-miR-27a-3p	-0.16028	8.383598	0.188258	0.66437	0.869258
bta-miR-3431	-0.37286	3.499521	0.18654	0.665812	0.869558
Novel:hsa-miR-6068	-0.22034	5.558004	0.182936	0.668862	0.871953
bta-miR-190b	-0.3447	4.016609	0.179008	0.672227	0.872386
Novel:NC_037553.1_24652	-0.27183	4.310882	0.178791	0.672414	0.872386
bta-miR-30e-5p	0.130717	11.36788	0.178292	0.672845	0.872386
Novel:hsa-miR-4746-5p	0.376887	4.056621	0.176045	0.674794	0.873334
Novel:NC_037562.1_38928	-0.51727	3.108416	0.172369	0.678015	0.874788
Novel:chi-miR-103-3p	0.383457	3.318532	0.171981	0.678357	0.874788
bta-miR-345-5p	-0.70627	2.862632	0.167616	0.682239	0.878214
Novel:hsa-miR-4687-3p	0.719403	2.937796	0.160983	0.688253	0.883049
Novel:hsa-miR-3919	-0.52386	2.993145	0.160037	0.689123	0.883049
bta-miR-2285ad	-0.57058	2.626072	0.159422	0.689689	0.883049
bta-miR-423-5p	0.115273	14.64461	0.153574	0.695143	0.887707
Novel:NC_037568.1_44905	-0.14448	9.246466	0.152122	0.696515	0.887707
bta-miR-2285dk	-0.56923	2.834518	0.149775	0.698751	0.887707
bta-miR-29b	0.572648	3.106137	0.149484	0.699029	0.887707
Novel:NC_037556.1_29371	0.439768	3.163377	0.148974	0.699518	0.887707

Novel:NC_037546.1_4856	-0.56184	2.839165	0.146548	0.701856	0.888609
Novel:NC_037555.1_28111	-0.32141	4.043133	0.14567	0.702708	0.888609
Novel:NC_037547.1_8396	-0.24724	5.624064	0.141747	0.70655	0.890321
bta-miR-16b	-0.23075	7.426045	0.140124	0.708158	0.890321
bta-miR-374a	-0.51575	3.183035	0.139437	0.708841	0.890321
Novel:NC_037563.1_39878	-0.14611	13.28083	0.138449	0.709828	0.890321
Novel:NC_037569.1_46554	-0.38699	3.526234	0.138008	0.71027	0.890321
bta-miR-335	0.493162	3.276377	0.136708	0.711576	0.890401
Novel:hsa-miR-149-5p	0.41053	2.843226	0.13271	0.715638	0.893925
bta-miR-93	-0.13307	9.256668	0.129346	0.719111	0.895376
bta-miR-2415-3p	-0.58146	2.796919	0.129166	0.719298	0.895376
Novel:NC_037548.1_13020	-0.56904	2.727169	0.127327	0.72122	0.896213
Novel:NC_037557.1_30645	-0.49199	2.979443	0.125054	0.723617	0.897635
Novel:NC_037564.1_40345	-0.33699	4.788002	0.122821	0.725995	0.89806
bta-miR-98	-0.28334	4.58166	0.119482	0.729597	0.89806
Novel:hsa-miR-6867-5p	0.208071	5.07842	0.119176	0.72993	0.89806
Novel:NC_037561.1_36929	0.544962	2.874724	0.119153	0.729955	0.89806
bta-miR-301b	0.544877	2.874724	0.118908	0.730222	0.89806
bta-miR-378d	0.380396	2.845031	0.11753	0.731729	0.898373
Novel:NC_037547.1_10635	0.106897	14.13686	0.114115	0.735507	0.901467
bta-miR-100	0.144938	9.924507	0.11085	0.739179	0.902353
bta-miR-2285dh	-0.27521	3.977307	0.108485	0.741875	0.902353
Novel:NC_037558.1_33220	-0.53619	2.894172	0.106448	0.744225	0.902353
Novel:NC_037558.1_32366	-0.53664	2.894172	0.106286	0.744413	0.902353
bta-miR-423-3p	-0.09805	11.6572	0.106221	0.744489	0.902353
bta-miR-1306	0.349368	3.520878	0.104414	0.746596	0.902353
bta-miR-502a	0.312009	3.381061	0.103798	0.747318	0.902353
Novel:hsa-miR-10399-3p	-0.34366	3.370617	0.102279	0.749112	0.902353
Novel:NC_037551.1_19470	-0.46069	3.044838	0.101696	0.749804	0.902353
bta-miR-184	-0.41381	2.608548	0.101464	0.75008	0.902353
bta-miR-1307	-0.1392	5.999873	0.09983	0.752034	0.902353
Novel:hsa-miR-4447	-0.20857	4.624777	0.099608	0.752301	0.902353
Novel:hsa-miR-1252-3p	0.47265	2.70974	0.099368	0.75259	0.902353
bta-let-7d	0.210393	4.922754	0.09472	0.758261	0.906549
bta-miR-15b	0.278629	3.405362	0.094431	0.758618	0.906549
Novel:NC_037556.1_29844	-0.18149	6.393095	0.092874	0.760555	0.907351
Novel:NC_037547.1_10096	0.43057	3.032015	0.089976	0.764207	0.908954
Novel:NC_037569.1_47131	0.324897	3.477358	0.088392	0.766232	0.908954
Novel:NC_037559.1_33968	0.090358	11.0665	0.088235	0.766433	0.908954
bta-miR-2285bn	-0.2084	3.788313	0.087818	0.766969	0.908954
Novel:hsa-miR-5002-5p	0.443409	2.785373	0.085132	0.77046	0.911503
Novel:NC_037569.1_47935	0.12406	5.657332	0.083556	0.772535	0.911503
bta-miR-7863	0.383896	2.749587	0.082378	0.7741	0.911503
bta-miR-149-5p	0.15639	4.862216	0.081416	0.775387	0.911503
Novel:NC_037553.1_23542	-0.1851	4.340908	0.081146	0.775751	0.911503
bta-miR-2285z	0.287217	3.813856	0.080371	0.776795	0.911503
Novel:NC_037567.1_44260	-0.12648	5.626546	0.079468	0.778019	0.911503
bta-miR-505	0.122947	5.640489	0.076328	0.782336	0.914422
Novel:hsa-miR-4795-5p	0.499024	2.83945	0.075807	0.783062	0.914422

Novel:hsa-miR-4277	0.353909	3.006983	0.074575	0.784788	0.914948
Novel:hsa-miR-4520-3p	0.145534	5.265897	0.072636	0.787537	0.916663
Novel:NC_037560.1_35884	0.1171	9.914386	0.071171	0.789639	0.916766
bta-miR-664b	-0.36882	2.964716	0.070795	0.790183	0.916766
Novel:NC_037557.1_30640	0.137759	8.609826	0.067176	0.795493	0.919648
bta-let-7b	-0.0839	10.53395	0.067009	0.795743	0.919648
Novel:hsa-miR-1251-3p	-0.15985	10.0836	0.066492	0.796515	0.919648
Novel:chi-miR-323b	-0.20169	4.589769	0.065087	0.798629	0.920606
Novel:NC_037569.1_48167	-0.26868	3.442445	0.06273	0.802232	0.923275
Novel:hsa-miR-1199-3p	0.184825	4.934689	0.06158	0.804016	0.923845
bta-miR-204	0.155615	6.370856	0.060342	0.805957	0.924593
Novel:NC_037558.1_32842	0.230341	4.193668	0.059346	0.807532	0.924921
bta-miR-193a-3p	0.172118	4.975719	0.056758	0.811695	0.928206
Novel:NC_037547.1_7559	-0.09947	7.456096	0.055766	0.813318	0.928267
Novel:NC_037545.1_498	-0.09609	7.809538	0.055147	0.814337	0.928267
Novel:NC_037551.1_20226	-0.31635	3.006439	0.050252	0.822625	0.936225
Novel:NC_037551.1_19485	-0.25459	3.085413	0.049257	0.824361	0.936715
Novel:NC_037563.1_39275	0.290357	2.688529	0.045888	0.830379	0.937663
Novel:NC_037556.1_30278	-0.35531	2.926606	0.04575	0.830631	0.937663
Novel:NC_037551.1_19947	0.28985	2.688529	0.04563	0.83085	0.937663
Novel:hsa-miR-943	0.267288	3.056188	0.04536	0.831344	0.937663
bta-miR-11971	0.112422	5.355816	0.045147	0.831734	0.937663
bta-miR-2285v	0.296491	2.834914	0.042929	0.835858	0.940274
Novel:NC_037546.1_5605	-0.30905	2.835686	0.042498	0.836674	0.940274
bta-miR-133a	-0.29103	3.357347	0.039581	0.842301	0.945117
Novel:NC_037546.1_5830	0.118471	5.000357	0.037171	0.847118	0.946379
bta-miR-25	-0.05219	14.39303	0.037137	0.847186	0.946379
Novel:hsa-miR-6771-5p	-0.13331	6.094075	0.037039	0.847386	0.946379
Novel:hsa-miR-3670	-0.21241	3.106179	0.036252	0.848997	0.946525
bta-miR-193a-5p	0.138831	3.965931	0.03569	0.850157	0.946525
bta-miR-127	-0.07363	10.66286	0.034987	0.851622	0.946687
Novel:hsa-miR-372-5p	0.155171	4.282333	0.033242	0.855329	0.947515
Novel:NC_037560.1_35002	-0.23586	2.792635	0.032538	0.856852	0.947515
Novel:hsa-miR-4514	0.135304	4.648796	0.029819	0.862902	0.947515
Novel:hsa-miR-4687-5p	0.214614	3.183461	0.029647	0.863293	0.947515
Novel:NC_037564.1_40236	0.129556	4.386462	0.029367	0.863933	0.947515
bta-miR-2389	0.155634	3.607879	0.029296	0.864098	0.947515
Novel:chi-miR-3432-5p	-0.29052	3.377342	0.028751	0.865355	0.947515
bta-miR-296-3p	-0.05927	9.493276	0.028366	0.866252	0.947515
bta-miR-99a-3p	0.172071	3.958825	0.027981	0.867155	0.947515
bta-miR-224	0.171736	3.514637	0.027582	0.868096	0.947515
bta-miR-503-5p	0.232356	2.778508	0.0264	0.870928	0.947515
bta-miR-2285cj	0.187906	3.22108	0.026327	0.871104	0.947515
bta-miR-494	-0.22431	3.453922	0.025213	0.873839	0.947515
Novel:NC_037548.1_13083	0.066848	6.061161	0.025168	0.873949	0.947515
Novel:NC_037550.1_19237	-0.28977	2.906945	0.024746	0.875003	0.947515
Novel:NC_037551.1_19765	0.095348	4.990051	0.024166	0.876464	0.947515
bta-miR-2285cs	-0.18181	2.71417	0.024112	0.8766	0.947515
Novel:NC_037553.1_22953	-0.13894	3.107605	0.024088	0.876662	0.947515

bta-miR-2285ba	-0.20012	2.957849	0.023584	0.877948	0.947515
bta-miR-744	-0.06432	7.496368	0.023254	0.878797	0.947515
Novel:hsa-miR-1301-3p	0.227025	3.09717	0.022458	0.880875	0.947709
bta-miR-323b-3p	0.136283	3.476705	0.022176	0.881621	0.947709
bta-miR-2285q	0.148392	3.74781	0.021558	0.88327	0.948061
Novel:chi-miR-202-3p	-0.04275	11.66276	0.017517	0.894706	0.957626
bta-miR-34a	-0.14285	3.638497	0.017282	0.895411	0.957626
Novel:NC_037569.1_47143	-0.13733	3.403257	0.017024	0.896188	0.957626
Novel:NC_037558.1_32026	-0.04558	9.097687	0.016102	0.899024	0.957794
bta-miR-6518	-0.06559	6.983553	0.016043	0.899207	0.957794
bta-miR-218	-0.20735	2.838594	0.015679	0.900353	0.957794
bta-miR-551b	0.109957	3.26456	0.013783	0.906544	0.962951
bta-miR-2478	-0.12038	3.637872	0.013019	0.909157	0.964298
Novel:hsa-miR-5195-5p	-0.04582	8.990694	0.012097	0.912421	0.965205
bta-miR-146b	-0.08203	4.585762	0.011974	0.912865	0.965205
bta-miR-2435	0.059323	5.485583	0.011649	0.914051	0.965205
bta-miR-199b	0.143622	2.913307	0.011238	0.915576	0.965394
bta-miR-210	-0.04285	8.751115	0.008456	0.926732	0.975589
Novel:hsa-miR-4720-5p	-0.12638	3.159891	0.008173	0.927966	0.975589
Novel:NC_037551.1_19861	0.068792	4.247531	0.006802	0.934268	0.980776
bta-miR-194	-0.04068	7.204873	0.006029	0.938107	0.982918
Novel:hsa-miR-4308	0.046764	4.185915	0.005787	0.93936	0.982918
Novel:NC_037560.1_35881	0.108205	2.668769	0.005586	0.940421	0.982918
Novel:hsa-miR-6747-3p	0.071264	3.570306	0.005041	0.943399	0.984206
Novel:hsa-miR-12136	0.041205	4.95628	0.004744	0.94509	0.984206
Novel:hsa-miR-6131	-0.03124	7.158174	0.004583	0.946025	0.984206
Novel:hsa-miR-6834-5p	0.081773	3.126574	0.004196	0.948349	0.984206
bta-miR-1388-5p	-0.02862	7.500498	0.004087	0.949024	0.984206
Novel:hsa-miR-4659a-3p	0.081745	3.589928	0.00381	0.95078	0.984206
Novel:hsa-miR-4689	0.059369	4.38055	0.003736	0.951262	0.984206
bta-miR-142-5p	-0.05454	3.240189	0.003248	0.954554	0.986189
bta-miR-2284j	0.076931	2.677837	0.002593	0.959392	0.989761
Novel:NC_037550.1_17420	0.053579	2.967709	0.002289	0.961845	0.990866
Novel:hsa-miR-4800-5p	0.016515	7.874658	0.00144	0.969732	0.995833
bta-miR-199c	-0.0544	2.861818	0.001382	0.970347	0.995833
Novel:NC_037566.1_42967	0.032607	3.562453	0.000971	0.975138	0.995833
bta-miR-2299-5p	-0.032	4.125175	0.000966	0.975211	0.995833
bta-miR-212	-0.02722	3.156252	0.000898	0.976093	0.995833
Novel:hsa-miR-3153	-0.02612	3.55733	0.000878	0.976357	0.995833
Novel:chi-miR-221-5p	-0.0235	3.379459	0.000675	0.979279	0.995833
Novel:chi-miR-378-3p	0.025833	2.641913	0.000612	0.98027	0.995833
Novel:chi-miR-500-5p	0.016771	3.285474	0.000579	0.980796	0.995833
bta-miR-155	-0.00898	8.919882	0.000456	0.982958	0.995833
bta-miR-378c	-0.0094	6.093591	0.000451	0.983058	0.995833
bta-miR-130b	-0.00806	7.656772	0.000436	0.983333	0.995833
Novel:NC_037553.1_22911	0.010485	4.646069	0.000239	0.987658	0.996375
bta-miR-769	-0.00603	7.285294	0.000206	0.988551	0.996375
Novel:hsa-miR-8057	-0.00511	8.404239	0.000176	0.989421	0.996375
Novel:NC_037567.1_44693	0.005406	5.570249	0.000129	0.990954	0.996375

Novel:NC_037564.1_41155	0.005349	5.758547	0.000108	0.991718	0.996375
Novel:NC_037561.1_36492	-0.01498	3.246286	5.96E-05	0.993839	0.996375
Novel:NC_037561.1_37162	-0.01475	3.246286	5.68E-05	0.993989	0.996375
Novel:hsa-miR-203a-3p	-0.00344	4.316646	3.95E-05	0.994985	0.996375
Novel:hsa-miR-6089	0.00129	3.66162	1.49E-05	0.996923	0.996923

Supplementary file S4.

List of the target genes for the most significant DE-miRNAs between extracellular vesicle (EVs) isolated from follicular fluid (FF) collected from antral (An) and preovulatory (pO) follicles (FDR < 10exp-6, LogFC>|2 and homologous with human miRNAs, n=14) with P-value<0.001.

hsa miRNA	Gene	RefseqID	Seed Start	Seed End	Pvalue
miR-1246	LZTFL1	NM_001276378	2897	2883	0
miR-1246	LZTFL1	NM_020347	2616	2602	0
miR-1246	LZTFL1	NM_001276379	2668	2654	0
miR-1246	ZFP69B	XM_005271136	2067	2056	0
miR-1246	ZFP69B	XM_005271137	2064	2053	0
miR-1246	ZFP69B	XM_005271139	1968	1957	0
miR-1246	ZFP69B	XM_005271138	1971	1960	0
miR-1246	ZFP69B	NM_023070	1981	1970	0
miR-148a-3p	TMEM246	XM_005252275	2181	2167	0
miR-148a-3p	TMEM246	XM_005252276	1995	1981	0
miR-193b-3p	DYM	XM_005258289	3625	3613	0
miR-194-5p	INTS10	XM_005273555	2492	2481	0
miR-194-5p	INTS10	XM_005273558	2286	2275	0
miR-194-5p	INTS10	NM_018142	2369	2358	0
miR-194-5p	INTS10	XM_005273557	2414	2403	0
miR-194-5p	INTS10	XM_005273556	2489	2478	0
miR-215-5p	ERCC3	XM_005263619	2785	2774	0
miR-215-5p	ERCC3	NM_000122	2608	2597	0
miR-215-5p	ERCC3	XM_005263618	2774	2763	0
miR-29c-3p	EIF4E2	NM_004846	1064	1054	0
miR-29c-3p	EIF4E2	XM_005246975	1464	1454	0
miR-29c-3p	TMEM236	XM_005252650	1256	1245	0
miR-29c-3p	TMEM236	XM_005275683	1256	1245	0
miR-378a-3p	GSPT2	NM_018094	2806	2795	0
miR-708-5p	ASPA	NM_000049	1163	1152	0
miR-708-5p	ASPA	NM_001128085	1096	1085	0
miR-708-5p	C10orf71	XM_005269476	5120	5108	0
miR-708-5p	C10orf71	XM_005269479	2755	2743	0
miR-708-5p	C10orf71	XM_005269477	5036	5024	0
miR-708-5p	C10orf71	XM_005269478	5055	5043	0
miR-708-5p	C10orf71	NM_001135196	5066	5054	0
miR-708-5p	KPNA4	NM_002268	8878	8861	0
miR-708-5p	MORF4L2	NM_001142418	1543	1532	0
miR-708-5p	MORF4L2	NM_001142420	1323	1312	0
miR-708-5p	MORF4L2	NM_001142426	1341	1330	0
miR-708-5p	MORF4L2	NM_001142431	1372	1361	0
miR-708-5p	MORF4L2	NM_001142430	1390	1379	0

miR-708-5p	MORF4L2	NM_001142421	1425	1414	0
miR-708-5p	MORF4L2	NM_001142423	1443	1432	0
miR-708-5p	MORF4L2	NM_001142427	1445	1434	0
miR-708-5p	MORF4L2	NM_001142425	1463	1452	0
miR-708-5p	MORF4L2	NM_001142428	1474	1463	0
miR-708-5p	MORF4L2	NM_001142419	1476	1465	0
miR-708-5p	MORF4L2	NM_001142424	1492	1481	0
miR-708-5p	MORF4L2	NM_001142432	1494	1483	0
miR-708-5p	MORF4L2	NM_012286	1494	1483	0
miR-708-5p	MORF4L2	NM_001142429	1512	1501	0
miR-708-5p	MORF4L2	NM_001142422	1525	1514	0
miR-708-5p	RAB2B	NM_032846	2394	2382	0
miR-708-5p	RAB2B	NM_001163380	2321	2309	0
miR-708-5p	RCVRN	NM_002903	1127	1116	0
miR-708-5p	ST6GALNAC5	XM_005271238	5372	5356	0
miR-708-5p	TEX14	NM_001201457	4674	4662	0
miR-708-5p	TEX14	XM_005257521	4374	4362	0
miR-708-5p	TEX14	XM_005257520	4477	4465	0
miR-708-5p	TEX14	XM_005257519	4529	4517	0
miR-708-5p	TEX14	NM_031272	4536	4524	0
miR-708-5p	TEX14	XM_005257518	4649	4637	0
miR-708-5p	TEX14	NM_198393	4656	4644	0
miR-708-5p	ZKSCAN8	NM_001278119	7384	7371	0
miR-708-5p	ZKSCAN8	NM_006298	7290	7277	0
miR-708-5p	ZKSCAN8	NM_001278122	7304	7291	0
miR-708-5p	ZKSCAN8	XM_005249382	7322	7309	0
miR-708-5p	ZKSCAN8	XM_005249383	7335	7322	0
miR-708-5p	ZKSCAN8	NM_001278121	7331	7318	0
miR-101-3p	CD163	NM_004244	3965	3955	0.0001
miR-101-3p	CD163	NM_203416	3882	3872	0.0001
miR-1246	E2F8	NM_024680	3412	3402	0.0001
miR-1246	E2F8	NM_001256372	3158	3148	0.0001
miR-1246	E2F8	NM_001256371	3213	3203	0.0001
miR-1246	FIGF	NM_004469	1634	1624	0.0001
miR-1246	MRC1	XM_005276375	3450	3440	0.0001
miR-1246	PHYHIPL	NM_032439	2947	2936	0.0001
miR-1246	PHYHIPL	NM_001143774	2727	2716	0.0001
miR-1246	PHYHIPL	XM_005270227	2583	2572	0.0001
miR-1246	PHYHIPL	XM_005270226	2596	2585	0.0001
miR-1246	PHYHIPL	XM_005270228	2660	2649	0.0001
miR-1246	ROPN1	NM_017578	1067	1058	0.0001
miR-1246	SLC24A5	XM_005254308	1639	1629	0.0001
miR-1246	SLC24A5	XM_005254309	1401	1391	0.0001
miR-1246	TPM4	XM_005260044	979	969	0.0001
miR-1246	VPS45	NM_007259	2798	2788	0.0001
miR-1246	VPS45	NM_001279355	2054	2044	0.0001
miR-1246	VPS45	NM_001279353	2341	2331	0.0001
miR-1246	VPS45	NM_001279354	2443	2433	0.0001
miR-130a-3p	A4GNT	NM_016161	1556	1546	0.0001

miR-130a-3p	CAST	NM_001042440	3629	3618	0.0001
miR-130a-3p	CAST	NM_173060	3337	3326	0.0001
miR-130a-3p	CAST	NM_001190442	3501	3490	0.0001
miR-130a-3p	DTD1	XM_005260871	2555	2544	0.0001
miR-132-3p	AFF2	NM_002025	7519	7507	0.0001
miR-132-3p	AFF2	NM_001170628	6053	6041	0.0001
miR-132-3p	AFF2	NM_001169125	7402	7390	0.0001
miR-132-3p	AFF2	NM_001169122	7414	7402	0.0001
miR-132-3p	AFF2	NM_001169124	7414	7402	0.0001
miR-132-3p	AFF2	NM_001169123	7489	7477	0.0001
miR-191-5p	FAM227A	XM_005261703	2502	2492	0.0001
miR-193b-3p	MCTS1	NM_014060	5162	5150	0.0001
miR-193b-3p	MCTS1	NM_001137554	4974	4962	0.0001
miR-193b-3p	WDR77	XM_005271191	797	788	0.0001
miR-194-5p	C9orf24	XM_005251617	1228	1219	0.0001
miR-194-5p	CPOX	XM_005247125	1703	1693	0.0001
miR-215-5p	EXOC4	NM_021807	4005	3994	0.0001
miR-215-5p	EXOC4	XM_005250521	2855	2844	0.0001
miR-215-5p	EXOC4	XM_005250522	2858	2847	0.0001
miR-215-5p	EXOC4	XM_005250520	4013	4002	0.0001
miR-215-5p	WWP2	NM_007014	4064	4053	0.0001
miR-215-5p	WWP2	NM_199424	3156	3145	0.0001
miR-215-5p	WWP2	NM_001270453	3701	3690	0.0001
miR-215-5p	WWP2	XM_005255778	3944	3933	0.0001
miR-215-5p	WWP2	NM_001270454	4011	4000	0.0001
miR-29c-3p	CDH11	XM_005255767	2261	2252	0.0001
miR-29c-3p	LOC101930547	XM_005276045	422	413	0.0001
miR-29c-3p	SERPINB9	XM_005249184	1662	1652	0.0001
miR-335-5p	BCAS3	XM_005257476	2970	2960	0.0001
miR-708-5p	GRIA4	XM_005271518	5400	5389	0.0001
miR-708-5p	GRIA4	NM_000829	5406	5395	0.0001
miR-708-5p	METTL21A	XM_005246340	2034	2023	0.0001
miR-708-5p	METTL21A	XM_005246337	2076	2065	0.0001
miR-708-5p	METTL21A	XM_005246338	2096	2085	0.0001
miR-708-5p	METTL21A	XM_005246339	2129	2118	0.0001
miR-708-5p	METTL21A	XM_005246341	2155	2144	0.0001
miR-708-5p	METTL21A	XM_005246336	2158	2147	0.0001
miR-708-5p	ODF2L	XM_005271055	3526	3515	0.0001
miR-708-5p	ODF2L	XM_005271058	3580	3569	0.0001
miR-708-5p	ODF2L	NM_001184766	3577	3566	0.0001
miR-708-5p	ODF2L	XM_005271056	3617	3606	0.0001
miR-708-5p	ODF2L	NM_001184765	3664	3653	0.0001
miR-708-5p	ODF2L	XM_005271054	3734	3723	0.0001
miR-708-5p	OR51I1	NM_001005288	1012	1004	0.0001
miR-708-5p	TYMP	NM_001113756	1833	1825	0.0001
miR-708-5p	TYMP	NM_001953	1649	1641	0.0001
miR-708-5p	TYMP	NM_001113755	1661	1653	0.0001
miR-708-5p	TYMP	NM_001257989	1664	1656	0.0001
miR-101-3p	LOC100996761	XM_005276163	917	909	0.0002

miR-101-3p	LOC100996761	XM_005277468	917	909	0.0002
miR-101-3p	LOC100996761	XM_003960065	1370	1362	0.0002
miR-101-3p	LOC100996761	XM_005276160	1416	1408	0.0002
miR-101-3p	MPP7	NM_173496	2348	2337	0.0002
miR-101-3p	MPP7	XM_005252367	2239	2228	0.0002
miR-101-3p	NDC1	NM_018087	3554	3543	0.0002
miR-101-3p	NDC1	NM_001168551	3434	3423	0.0002
miR-101-3p	SORL1	XM_005271652	5550	5539	0.0002
miR-101-3p	TNRC6B	XM_005261393	16016	16004	0.0002
miR-101-3p	TNRC6B	XM_005261394	12654	12642	0.0002
miR-101-3p	TNRC6B	NM_001024843	12721	12709	0.0002
miR-101-3p	TNRC6B	NM_015088	14712	14700	0.0002
miR-101-3p	TNRC6B	NM_001162501	15042	15030	0.0002
miR-101-3p	ZNF143	XM_005253127	2834	2824	0.0002
miR-101-3p	ZNF143	XM_005253126	2576	2566	0.0002
miR-101-3p	ZNF143	XM_005253125	2695	2685	0.0002
miR-101-3p	ZNF143	XM_005253121	2756	2746	0.0002
miR-101-3p	ZNF143	XM_005253122	2781	2771	0.0002
miR-101-3p	ZNF143	NM_003442	2808	2798	0.0002
miR-1246	BEGAIN	NM_001159531	2492	2482	0.0002
miR-1246	BEGAIN	XM_005267919	2393	2383	0.0002
miR-1246	BEGAIN	NM_020836	2418	2408	0.0002
miR-1246	BEGAIN	XM_005267920	2435	2425	0.0002
miR-1246	BEGAIN	XM_005267921	2464	2454	0.0002
miR-1246	BEGAIN	XM_005267922	2467	2457	0.0002
miR-1246	GDF11	NM_005811	1756	1745	0.0002
miR-1246	MARCH8	XM_005271806	3214	3203	0.0002
miR-1246	MARCH8	NM_001002266	1850	1839	0.0002
miR-1246	MARCH8	NM_145021	2338	2327	0.0002
miR-1246	MARCH8	XM_005271804	3121	3110	0.0002
miR-1246	PTPLAD2	NM_001010915	858	848	0.0002
miR-1246	TCN1	NM_001062	1436	1427	0.0002
miR-1246	TUBGCP3	NM_006322	3244	3234	0.0002
miR-1246	TUBGCP3	XM_005268291	3216	3206	0.0002
miR-1246	ZNF155	XM_005259215	2060	2051	0.0002
miR-130a-3p	PHF20	NM_016436	5757	5746	0.0002
miR-130a-3p	PHF20	XM_005260421	5367	5356	0.0002
miR-130a-3p	PHF20	XM_005260420	5626	5615	0.0002
miR-132-3p	PDE3A	NM_001244683	6366	6355	0.0002
miR-132-3p	PDE3A	NM_000921	7109	7098	0.0002
miR-132-3p	SPAST	XM_005264516	2899	2888	0.0002
miR-132-3p	SPAST	NM_199436	2786	2775	0.0002
miR-132-3p	SPAST	NM_014946	2882	2871	0.0002
miR-148a-3p	SF3A2	NM_007165	1642	1633	0.0002
miR-194-5p	FAM208B	NM_017782	8363	8353	0.0002
miR-194-5p	KCTD17	XM_005261746	728	719	0.0002
miR-194-5p	KCTD17	XM_005261741	1109	1100	0.0002
miR-194-5p	KLK13	XM_005258723	1423	1413	0.0002
miR-194-5p	KLK13	XM_005258724	1186	1176	0.0002

miR-194-5p	SRGN	NM_002727	1117	1107	0.0002
miR-215-5p	GRIA1	NM_001258020	3945	3934	0.0002
miR-215-5p	GRIA1	NM_001258023	3477	3466	0.0002
miR-215-5p	GRIA1	NM_001258021	3580	3569	0.0002
miR-215-5p	GRIA1	NM_001258022	3580	3569	0.0002
miR-215-5p	GRIA1	NM_001258019	3648	3637	0.0002
miR-215-5p	GRIA1	NM_000827	3888	3877	0.0002
miR-215-5p	GRIA1	NM_001114183	3888	3877	0.0002
miR-29c-3p	CHRNB2	NM_000748	4172	4161	0.0002
miR-29c-3p	HAPLN3	XM_005254871	2069	2059	0.0002
miR-29c-3p	HAPLN3	NM_178232	1871	1861	0.0002
miR-29c-3p	IFI30	NM_006332	999	990	0.0002
miR-29c-3p	ISG20L2	NM_030980	1581	1571	0.0002
miR-335-5p	GPT	NM_005309	1802	1793	0.0002
miR-335-5p	NAA30	NM_001011713	2974	2963	0.0002
miR-378a-3p	CADM4	NM_145296	1468	1458	0.0002
miR-378a-3p	CADM4	XM_005258620	1321	1311	0.0002
miR-708-5p	GRIA4	NM_001077243	5519	5508	0.0002
miR-708-5p	METTL21A	NM_001127395	4393	4382	0.0002
miR-708-5p	METTL21A	NM_145280	4336	4325	0.0002
miR-708-5p	MTHFD1L	XM_005266911	1939	1931	0.0002
miR-708-5p	SLC35C1	XM_005253003	1064	1056	0.0002
miR-708-5p	TEX261	NM_144582	3270	3259	0.0002
miR-708-5p	TEX261	XM_005264121	3150	3139	0.0002
miR-708-5p	TEX261	XM_005278320	3150	3139	0.0002
miR-708-5p	TLN2	XM_005254709	8974	8963	0.0002
miR-708-5p	TLN2	NM_015059	8226	8215	0.0002
miR-708-5p	TLN2	XM_005254717	8328	8317	0.0002
miR-708-5p	TLN2	XM_005254716	8403	8392	0.0002
miR-708-5p	TLN2	XM_005254707	8448	8437	0.0002
miR-708-5p	TLN2	XM_005254714	8499	8488	0.0002
miR-708-5p	TLN2	XM_005254715	8501	8490	0.0002
miR-708-5p	TLN2	XM_005254708	8600	8589	0.0002
miR-708-5p	TLN2	XM_005254712	8622	8611	0.0002
miR-708-5p	TLN2	XM_005254710	8743	8732	0.0002
miR-708-5p	TLN2	XM_005254713	8775	8764	0.0002
miR-708-5p	TLN2	XM_005254711	8831	8820	0.0002
miR-708-5p	TYMP	NM_001257988	1684	1676	0.0002
miR-101-3p	CPM	NM_001874	6045	6034	0.0003
miR-101-3p	CPM	NM_001005502	6013	6002	0.0003
miR-101-3p	CPM	NM_198320	6031	6020	0.0003
miR-101-3p	PROK2	NM_001126128	1571	1561	0.0003
miR-101-3p	PROK2	NM_021935	1508	1498	0.0003
miR-101-3p	SORL1	NM_003105	8943	8932	0.0003
miR-101-3p	ZBTB34	XM_005251989	4511	4500	0.0003
miR-101-3p	ZBTB34	NM_001099270	4419	4408	0.0003
miR-1246	ATF1	NM_005171	1464	1454	0.0003
miR-1246	C11orf54	XM_005273935	2191	2181	0.0003
miR-1246	C11orf54	XM_005273937	1680	1670	0.0003

miR-1246	C11orf54	NM_014039	1684	1674	0.0003
miR-1246	C11orf54	XM_005273936	1853	1843	0.0003
miR-1246	C11orf54	XM_005273934	1858	1848	0.0003
miR-1246	CLINT1	XM_005266013	2843	2833	0.0003
miR-1246	DYNLL1	NM_001037494	777	768	0.0003
miR-1246	DYNLL1	NM_003746	690	681	0.0003
miR-1246	DYNLL1	NM_001037495	704	695	0.0003
miR-1246	KDM5A	NM_001042603	6724	6713	0.0003
miR-1246	PGM5	NM_021965	2096	2086	0.0003
miR-1246	RANBP1	NM_001278639	1284	1275	0.0003
miR-1246	RANBP1	NM_001278640	1029	1020	0.0003
miR-1246	RANBP1	NM_002882	1032	1023	0.0003
miR-1246	RANBP1	XM_005261266	1147	1138	0.0003
miR-1246	RANBP1	NM_001278641	1149	1140	0.0003
miR-130a-3p	MPL	XM_005270874	2037	2028	0.0003
miR-130a-3p	SESTD1	XM_005246944	2332	2321	0.0003
miR-130a-3p	TCF4	NM_001083962	7955	7944	0.0003
miR-130a-3p	TCF4	NM_001243235	6972	6961	0.0003
miR-130a-3p	TCF4	NM_001243234	6984	6973	0.0003
miR-130a-3p	TCF4	NM_001243236	6988	6977	0.0003
miR-130a-3p	TCF4	NM_001243233	7340	7329	0.0003
miR-130a-3p	TCF4	NM_001243231	7377	7366	0.0003
miR-130a-3p	TCF4	NM_001243232	7411	7400	0.0003
miR-130a-3p	TCF4	NM_001243230	7416	7405	0.0003
miR-130a-3p	TCF4	NM_001243226	7709	7698	0.0003
miR-130a-3p	TCF4	NM_001243227	7789	7778	0.0003
miR-130a-3p	TCF4	NM_001243228	7899	7888	0.0003
miR-130a-3p	TCF4	NM_003199	7943	7932	0.0003
miR-132-3p	FAM184A	NM_024581	3807	3798	0.0003
miR-132-3p	FAM184A	NM_001100411	3269	3260	0.0003
miR-132-3p	FAM184A	XM_005267143	3356	3347	0.0003
miR-132-3p	FAM184A	XM_005267145	3358	3349	0.0003
miR-132-3p	FAM184A	XM_005267142	3461	3452	0.0003
miR-132-3p	FAM184A	XM_005267144	3610	3601	0.0003
miR-132-3p	FAM184A	XM_005267141	3798	3789	0.0003
miR-132-3p	MIER1	XM_005271077	602	593	0.0003
miR-132-3p	SLC25A28	XM_005270209	5004	4995	0.0003
miR-132-3p	SLC25A28	XM_005270211	1094	1085	0.0003
miR-132-3p	SLC25A28	XM_005270210	1194	1185	0.0003
miR-132-3p	SLC25A28	XM_005270208	1486	1477	0.0003
miR-132-3p	SLC25A28	NM_031212	1496	1487	0.0003
miR-132-3p	TMEM106B	NM_018374	5873	5862	0.0003
miR-132-3p	TMEM106B	NM_001134232	5679	5668	0.0003
miR-132-3p	TMEM106B	XM_005249789	5729	5718	0.0003
miR-148a-3p	KRTAP2-1	NM_001123387	538	529	0.0003
miR-191-5p	AJAP1	NM_018836	1686	1676	0.0003
miR-193b-3p	LYPD1	NM_144586	1813	1803	0.0003
miR-193b-3p	LYPD1	XM_005263583	999	989	0.0003
miR-193b-3p	LYPD1	NM_001077427	1104	1094	0.0003

miR-193b-3p	STX18	NM_016930	1825	1815	0.0003
miR-193b-3p	USP54	XM_005269583	2582	2574	0.0003
miR-194-5p	ATP6V1F	NM_001198909	766	757	0.0003
miR-194-5p	ATP6V1F	NM_004231	682	673	0.0003
miR-194-5p	EOMES	NM_001278182	3000	2990	0.0003
miR-194-5p	EOMES	NM_001278183	1984	1974	0.0003
miR-194-5p	EOMES	XM_005265509	2086	2076	0.0003
miR-194-5p	EOMES	NM_005442	2943	2933	0.0003
miR-194-5p	IP6K2	NM_001190316	1146	1136	0.0003
miR-194-5p	IP6K2	NM_001190317	1069	1059	0.0003
miR-194-5p	RAD51B	NM_133509	1940	1930	0.0003
miR-194-5p	SUMO3	XM_005261165	1030	1020	0.0003
miR-194-5p	SUMO3	XM_005261166	844	834	0.0003
miR-194-5p	SUMO3	NM_006936	954	944	0.0003
miR-215-5p	MYO6	NM_004999	6796	6785	0.0003
miR-29c-3p	ADAMTS7	NM_014272	5362	5353	0.0003
miR-29c-3p	HMCN1	NM_031935	17254	17244	0.0003
miR-29c-3p	ISG20L2	XM_005245516	1808	1798	0.0003
miR-29c-3p	ISG20L2	XM_005245518	830	820	0.0003
miR-29c-3p	ISG20L2	XM_005245517	1493	1483	0.0003
miR-29c-3p	PMP22	NM_000304	1405	1395	0.0003
miR-29c-3p	PMP22	NM_153322	1284	1274	0.0003
miR-29c-3p	PMP22	NM_153321	1362	1352	0.0003
miR-29c-3p	PMP22	NM_001281455	1366	1356	0.0003
miR-29c-3p	PMP22	NM_001281456	1401	1391	0.0003
miR-29c-3p	TMEM236	NM_001098844	1271	1260	0.0003
miR-335-5p	KIAA1841	XM_005264605	2500	2492	0.0003
miR-335-5p	MUC13	NM_033049	1970	1960	0.0003
miR-335-5p	ZBTB12	NM_181842	1733	1724	0.0003
miR-378a-3p	RAPH1	NM_213589	5312	5301	0.0003
miR-378a-3p	RAPH1	XM_005246780	5337	5326	0.0003
miR-378a-3p	RAPH1	XM_005246778	5365	5354	0.0003
miR-378a-3p	RAPH1	XM_005246777	5412	5401	0.0003
miR-378a-3p	RAPH1	XM_005246779	5453	5442	0.0003
miR-378a-3p	ZDHHC12	XM_005252278	1346	1337	0.0003
miR-378a-3p	ZDHHC12	XM_005252280	977	968	0.0003
miR-378a-3p	ZDHHC12	NM_032799	1138	1129	0.0003
miR-101-3p	CBWD1	XM_005251515	1309	1300	0.0004
miR-101-3p	CBWD1	XM_005251514	1366	1357	0.0004
miR-101-3p	CBWD1	XM_005251513	1395	1386	0.0004
miR-101-3p	CBWD1	NM_001145356	1396	1387	0.0004
miR-101-3p	CBWD1	NM_018491	1453	1444	0.0004
miR-101-3p	CBWD1	NM_001145355	1551	1542	0.0004
miR-101-3p	CBWD3	NM_201453	1456	1447	0.0004
miR-101-3p	CBWD3	XM_005251993	845	836	0.0004
miR-101-3p	CBWD3	XM_005277643	845	836	0.0004
miR-101-3p	CBWD3	XM_005251992	1306	1297	0.0004
miR-101-3p	CBWD3	XM_005277640	1306	1297	0.0004
miR-101-3p	CBWD3	XM_005277639	1363	1354	0.0004

miR-101-3p	CBWD3	XM_005251991	1390	1381	0.0004
miR-101-3p	CBWD3	XM_005277638	1390	1381	0.0004
miR-101-3p	CBWD3	XM_005251990	1393	1384	0.0004
miR-101-3p	CBWD3	XM_005277637	1393	1384	0.0004
miR-101-3p	CBWD5	XM_005272752	1294	1285	0.0004
miR-101-3p	CBWD5	NM_001024916	1306	1297	0.0004
miR-101-3p	CBWD5	XM_005272751	1363	1354	0.0004
miR-101-3p	CBWD5	XM_005272750	1390	1381	0.0004
miR-101-3p	CBWD5	XM_005272749	1393	1384	0.0004
miR-101-3p	CBWD5	XM_005272748	1450	1441	0.0004
miR-101-3p	CBWD6	NM_001085457	1453	1444	0.0004
miR-101-3p	CBWD6	XM_005272536	1303	1294	0.0004
miR-101-3p	CBWD6	XM_005272535	1306	1297	0.0004
miR-101-3p	CBWD6	XM_005272534	1390	1381	0.0004
miR-101-3p	CBWD6	XM_005272533	1393	1384	0.0004
miR-101-3p	CBWD7	XM_001717050	852	843	0.0004
miR-101-3p	LOC101060578	XM_003960454	825	816	0.0004
miR-101-3p	PRRC2C	XM_005245020	10122	10112	0.0004
miR-101-3p	PRRC2C	XM_005245029	9713	9703	0.0004
miR-101-3p	PRRC2C	XM_005245027	9728	9718	0.0004
miR-101-3p	PRRC2C	NM_015172	9797	9787	0.0004
miR-101-3p	PRRC2C	XM_005245024	9817	9807	0.0004
miR-101-3p	PRRC2C	XM_005245023	9832	9822	0.0004
miR-101-3p	PRRC2C	XM_005245026	9870	9860	0.0004
miR-101-3p	PRRC2C	XM_005245022	9874	9864	0.0004
miR-101-3p	PRRC2C	XM_005245025	9885	9875	0.0004
miR-101-3p	PRRC2C	XM_005245021	9913	9903	0.0004
miR-101-3p	PRRC2C	XM_005245019	9967	9957	0.0004
miR-101-3p	PRRC2C	XM_005245016	10048	10038	0.0004
miR-101-3p	PRRC2C	XM_005245017	10054	10044	0.0004
miR-101-3p	PRRC2C	XM_005245018	10054	10044	0.0004
miR-101-3p	PRRC2C	XM_005245015	10069	10059	0.0004
miR-1246	CYSTM1	NM_032412	734	725	0.0004
miR-1246	MSR1	NM_002445	1873	1863	0.0004
miR-1246	ZNF254	XM_005260167	3398	3388	0.0004
miR-1246	ZNF254	XM_005260169	2021	2011	0.0004
miR-1246	ZNF254	XM_005260168	2071	2061	0.0004
miR-1246	ZNF254	XM_005260166	2441	2431	0.0004
miR-132-3p	AHCY	XM_005260315	1629	1620	0.0004
miR-132-3p	C11orf87	XM_005271544	2428	2418	0.0004
miR-132-3p	EFCAB9	NM_001171183	668	660	0.0004
miR-132-3p	FREM2	NM_207361	11864	11853	0.0004
miR-132-3p	PDE3A	XM_005253389	6230	6219	0.0004
miR-148a-3p	MCMDC2	NM_001136161	2202	2193	0.0004
miR-191-5p	TOR1AIP1	NM_001267578	2880	2870	0.0004
miR-191-5p	TOR1AIP1	NM_015602	2877	2867	0.0004
miR-193b-3p	KCNJ11	XM_005252910	2621	2611	0.0004
miR-193b-3p	KCNJ11	NM_001166290	1661	1651	0.0004
miR-193b-3p	KCNJ11	NM_000525	2316	2306	0.0004

miR-193b-3p	NCOR1	XM_005256876	8690	8680	0.0004
miR-193b-3p	NCOR1	XM_005256875	8819	8809	0.0004
miR-193b-3p	NCOR1	XM_005256874	8873	8863	0.0004
miR-193b-3p	NCOR1	XM_005256867	8884	8874	0.0004
miR-193b-3p	NCOR1	XM_005256873	8999	8989	0.0004
miR-193b-3p	NCOR1	XM_005256872	9017	9007	0.0004
miR-193b-3p	NCOR1	XM_005256871	9020	9010	0.0004
miR-193b-3p	NCOR1	XM_005256868	9044	9034	0.0004
miR-193b-3p	NCOR1	XM_005256869	9044	9034	0.0004
miR-193b-3p	NCOR1	XM_005256870	9044	9034	0.0004
miR-193b-3p	NCOR1	XM_005256866	9047	9037	0.0004
miR-193b-3p	TNFRSF10C	NM_003841	1383	1374	0.0004
miR-194-5p	ARHGAP6	NM_006125	3430	3421	0.0004
miR-194-5p	CHP1	XM_005254140	721	713	0.0004
miR-215-5p	B3GALNT1	NM_001038628	2458	2448	0.0004
miR-215-5p	B3GALNT1	NM_033168	2040	2030	0.0004
miR-215-5p	B3GALNT1	XM_005247863	2082	2072	0.0004
miR-215-5p	B3GALNT1	XM_005247860	2084	2074	0.0004
miR-215-5p	B3GALNT1	XM_005247864	2085	2075	0.0004
miR-215-5p	B3GALNT1	NM_033169	2108	2098	0.0004
miR-215-5p	B3GALNT1	NM_003781	2135	2125	0.0004
miR-215-5p	B3GALNT1	XM_005247862	2159	2149	0.0004
miR-215-5p	B3GALNT1	XM_005247859	2177	2167	0.0004
miR-215-5p	B3GALNT1	XM_005247858	2182	2172	0.0004
miR-215-5p	B3GALNT1	NM_033167	2254	2244	0.0004
miR-215-5p	B3GALNT1	XM_005247861	2318	2308	0.0004
miR-29c-3p	COL2A1	NM_001844	5013	5004	0.0004
miR-29c-3p	COL2A1	NM_033150	4806	4797	0.0004
miR-335-5p	NXPH2	NM_007226	1145	1135	0.0004
miR-378a-3p	KPNA4	NM_002268	7097	7086	0.0004
miR-378a-3p	ZDHHC12	XM_005252279	1111	1102	0.0004
miR-708-5p	DCAF10	NM_024345	7816	7805	0.0004
miR-708-5p	ZNF157	NM_003446	1621	1613	0.0004
miR-101-3p	MGAT4A	XM_005263866	3744	3734	0.0005
miR-101-3p	PRRC2C	XM_005245028	9781	9771	0.0005
miR-1246	CLINT1	NM_001195555	3042	3032	0.0005
miR-1246	CLINT1	NM_014666	2988	2978	0.0005
miR-1246	CLINT1	NM_001195556	3019	3009	0.0005
miR-1246	GRAMD2	NM_001012642	2875	2865	0.0005
miR-1246	KIAA1522	NM_020888	5331	5321	0.0005
miR-1246	KIAA1522	NM_001198973	2570	2560	0.0005
miR-1246	KIAA1522	XM_005271073	5126	5116	0.0005
miR-1246	KIAA1522	NM_001198972	5246	5236	0.0005
miR-1246	PTPRA	NM_002836	3398	3389	0.0005
miR-1246	PTPRA	XM_005260781	2603	2594	0.0005
miR-1246	PTPRA	XM_005260779	2748	2739	0.0005
miR-1246	PTPRA	NM_080841	2914	2905	0.0005
miR-1246	PTPRA	XM_005260777	2999	2990	0.0005
miR-1246	PTPRA	XM_005260776	3026	3017	0.0005

miR-1246	PTPRA	XM_005260778	3069	3060	0.0005
miR-1246	PTPRA	NM_080840	3089	3080	0.0005
miR-1246	PTPRA	XM_005260780	3142	3133	0.0005
miR-1246	SGOL1	NM_001199257	1444	1435	0.0005
miR-1246	SGOL1	NM_138484	1538	1529	0.0005
miR-1246	SGOL1	NM_001012412	1589	1580	0.0005
miR-1246	SGOL1	NM_001199256	1675	1666	0.0005
miR-1246	SGOL1	NM_001199254	1726	1717	0.0005
miR-1246	SGOL1	NM_001012410	2345	2336	0.0005
miR-1246	SGOL1	NM_001199252	2482	2473	0.0005
miR-1246	ZNF254	NM_001278678	2243	2233	0.0005
miR-1246	ZNF254	NM_001278664	2339	2329	0.0005
miR-1246	ZNF254	NM_001278663	2378	2368	0.0005
miR-1246	ZNF254	NM_203282	2466	2456	0.0005
miR-1246	ZNF254	NM_001278662	2581	2571	0.0005
miR-1246	ZNF254	NM_001278661	2601	2591	0.0005
miR-1246	ZNF254	NM_001278677	2673	2663	0.0005
miR-130a-3p	ARL16	NM_001040025	1150	1141	0.0005
miR-130a-3p	SESTD1	NM_178123	2478	2467	0.0005
miR-132-3p	GDF5	NM_000557	2340	2331	0.0005
miR-132-3p	GRM3	NM_000840	3779	3770	0.0005
miR-132-3p	GRM3	XM_005250289	2282	2273	0.0005
miR-132-3p	GRM3	XM_005250287	3145	3136	0.0005
miR-132-3p	POLG2	XM_005276869	1658	1649	0.0005
miR-148a-3p	MAP3K4	NM_005922	5360	5351	0.0005
miR-148a-3p	MAP3K4	XM_005266989	5204	5195	0.0005
miR-148a-3p	MAP3K4	NM_006724	5210	5201	0.0005
miR-148a-3p	MAP3K4	XM_005266988	5354	5345	0.0005
miR-193b-3p	NCOR1	NM_006311	8812	8802	0.0005
miR-193b-3p	NCOR1	NM_001190440	8304	8294	0.0005
miR-193b-3p	SLC35D2	NM_007001	1490	1481	0.0005
miR-193b-3p	SLC35D2	XM_005251678	1119	1110	0.0005
miR-193b-3p	SLC35D2	XM_005251677	1218	1209	0.0005
miR-193b-3p	SLC35D2	XM_005251675	1333	1324	0.0005
miR-215-5p	BTN2A2	NM_001197237	2831	2821	0.0005
miR-215-5p	BTN2A2	NM_001197239	2179	2169	0.0005
miR-215-5p	BTN2A2	NM_181531	2461	2451	0.0005
miR-215-5p	BTN2A2	XM_005248798	2797	2787	0.0005
miR-215-5p	BTN2A2	NM_006995	2809	2799	0.0005
miR-29c-3p	HPGD	NM_001256305	930	920	0.0005
miR-29c-3p	HPGD	NM_001145816	1007	997	0.0005
miR-29c-3p	RHOBTB1	NM_001242359	3734	3724	0.0005
miR-29c-3p	RHOBTB1	NM_014836	3662	3652	0.0005
miR-29c-3p	SCML2	NM_006089	3838	3828	0.0005
miR-335-5p	PRDM2	NM_012231	6676	6666	0.0005
miR-335-5p	PRDM2	NM_001135610	1421	1411	0.0005
miR-335-5p	PRDM2	XM_005245997	5645	5635	0.0005
miR-335-5p	PRDM2	XM_005245994	5817	5807	0.0005
miR-335-5p	PRDM2	XM_005245996	5866	5856	0.0005

miR-335-5p	PRDM2	XM_005245992	5904	5894	0.0005
miR-335-5p	PRDM2	XM_005245993	5967	5957	0.0005
miR-378a-3p	FBXO9	XM_005248995	1363	1354	0.0005
miR-378a-3p	FBXO9	XM_005248996	1403	1394	0.0005
miR-378a-3p	PRPSAP2	XM_005256724	1910	1901	0.0005
miR-378a-3p	PRPSAP2	XM_005256729	1586	1577	0.0005
miR-378a-3p	PRPSAP2	XM_005256725	1654	1645	0.0005
miR-378a-3p	PRPSAP2	XM_005256728	1664	1655	0.0005
miR-378a-3p	PRPSAP2	XM_005256727	1718	1709	0.0005
miR-378a-3p	PRPSAP2	XM_005256726	1725	1716	0.0005
miR-378a-3p	SYN2	NM_003178	1882	1872	0.0005
miR-708-5p	FCAMR	NM_001170631	2693	2684	0.0005
miR-101-3p	KTI12	NM_138417	1209	1200	0.0006
miR-101-3p	MGAT4A	NM_001160154	3196	3186	0.0006
miR-1246	CALM2	XM_005264576	1708	1699	0.0006
miR-1246	CCDC132	NM_001257998	4137	4127	0.0006
miR-1246	CCDC132	XM_005250488	3230	3220	0.0006
miR-1246	CCDC132	XM_005250487	3929	3919	0.0006
miR-1246	CCDC132	XM_005250486	4082	4072	0.0006
miR-1246	CCDC132	NM_017667	4109	4099	0.0006
miR-1246	EXOSC9	NM_005033	1520	1512	0.0006
miR-1246	EXOSC9	NM_001034194	1571	1563	0.0006
miR-1246	KLC1	XM_005267631	1917	1908	0.0006
miR-1246	KLC1	XM_005267629	1944	1935	0.0006
miR-1246	KLC1	XM_005267630	1944	1935	0.0006
miR-1246	KLC1	XM_005267628	1968	1959	0.0006
miR-1246	KLC1	XM_005267626	1971	1962	0.0006
miR-1246	KLC1	XM_005267623	1992	1983	0.0006
miR-1246	KLC1	XM_005267622	2043	2034	0.0006
miR-1246	KLC1	XM_005267620	2070	2061	0.0006
miR-1246	KLC1	NM_005552	2258	2249	0.0006
miR-1246	MBNL2	NM_207304	2900	2890	0.0006
miR-1246	MBNL2	XM_005254020	2937	2927	0.0006
miR-1246	MBNL2	XM_005254019	2955	2945	0.0006
miR-1246	PPTC7	NM_139283	1761	1751	0.0006
miR-1246	PYGO1	XM_005254300	1677	1668	0.0006
miR-1246	THADA	NM_001271643	3203	3195	0.0006
miR-1246	TM2D3	NM_078474	1039	1030	0.0006
miR-1246	TM2D3	NM_025141	961	952	0.0006
miR-130a-3p	MRPL42	NM_172177	2828	2818	0.0006
miR-130a-3p	MRPL42	NM_014050	2825	2815	0.0006
miR-130a-3p	RERE	NM_012102	6825	6815	0.0006
miR-130a-3p	RERE	NM_001042682	4939	4929	0.0006
miR-130a-3p	RERE	XM_005263466	5462	5452	0.0006
miR-130a-3p	RERE	XM_005263465	6193	6183	0.0006
miR-130a-3p	RERE	XM_005263464	6319	6309	0.0006
miR-130a-3p	RERE	NM_001042681	6640	6630	0.0006
miR-130a-3p	SH2D3A	XM_005259466	2290	2281	0.0006
miR-130a-3p	SH2D3A	XM_005259473	1826	1817	0.0006

miR-130a-3p	SH2D3A	XM_005259470	1930	1921	0.0006
miR-130a-3p	SH2D3A	XM_005259471	1998	1989	0.0006
miR-130a-3p	SH2D3A	XM_005259469	2029	2020	0.0006
miR-130a-3p	SH2D3A	XM_005259468	2203	2194	0.0006
miR-148a-3p	TRPM5	XM_005252882	3567	3559	0.0006
miR-148a-3p	TRPM5	XM_005252881	3588	3580	0.0006
miR-191-5p	SSX4	NM_005636	1178	1169	0.0006
miR-191-5p	SSX4	NM_175729	1042	1033	0.0006
miR-191-5p	SSX4B	NM_001034832	1189	1180	0.0006
miR-191-5p	SSX4B	NM_001040612	1053	1044	0.0006
miR-193b-3p	SEPN1	NM_020451	4134	4124	0.0006
miR-193b-3p	SEPN1	NM_206926	4032	4022	0.0006
miR-193b-3p	SLC35D2	XM_005251676	1314	1305	0.0006
miR-193b-3p	SLC35D2	XM_005251674	1382	1373	0.0006
miR-193b-3p	SLC35D2	XM_005251673	1397	1388	0.0006
miR-193b-3p	STK11	XM_005259618	2620	2610	0.0006
miR-194-5p	ATE1	NM_001001976	2129	2120	0.0006
miR-194-5p	ATE1	NM_007041	2129	2120	0.0006
miR-194-5p	TRIM23	NM_033228	1784	1775	0.0006
miR-194-5p	TRIM23	NM_033227	1813	1804	0.0006
miR-215-5p	BTN2A2	NM_001197240	2680	2670	0.0006
miR-29c-3p	FOXJ2	NM_018416	3354	3344	0.0006
miR-29c-3p	FOXJ2	XM_005253430	2401	2391	0.0006
miR-29c-3p	SLC16A14	XM_005246352	4115	4105	0.0006
miR-29c-3p	SLC16A14	XM_005246354	3169	3159	0.0006
miR-29c-3p	SLC16A14	XM_005246353	3838	3828	0.0006
miR-29c-3p	SLC16A14	NM_152527	3982	3972	0.0006
miR-335-5p	GPN1	NM_007266	1602	1593	0.0006
miR-335-5p	GPN1	NM_001145049	1388	1379	0.0006
miR-335-5p	GPN1	NM_001145048	1495	1486	0.0006
miR-335-5p	GPN1	NM_001145047	1563	1554	0.0006
miR-335-5p	NAA25	XM_005253938	4002	3992	0.0006
miR-335-5p	SRSF4	NM_005626	2223	2214	0.0006
miR-378a-3p	PRPSAP2	NM_001243940	1496	1487	0.0006
miR-378a-3p	PRPSAP2	NM_001243936	1654	1645	0.0006
miR-378a-3p	PRPSAP2	NM_001243941	1688	1679	0.0006
miR-378a-3p	PRPSAP2	NM_001243942	1721	1712	0.0006
miR-378a-3p	PRPSAP2	NM_002767	1837	1828	0.0006
miR-378a-3p	TUFM	NM_003321	1821	1812	0.0006
miR-1246	C11orf30	XM_005274106	6677	6667	0.0007
miR-1246	C11orf30	XM_005274113	6233	6223	0.0007
miR-1246	C11orf30	XM_005274112	6482	6472	0.0007
miR-1246	C11orf30	XM_005274111	6512	6502	0.0007
miR-1246	C11orf30	XM_005274110	6524	6514	0.0007
miR-1246	C11orf30	XM_005274108	6530	6520	0.0007
miR-1246	C11orf30	XM_005274109	6635	6625	0.0007
miR-1246	C11orf30	XM_005274107	6674	6664	0.0007
miR-1246	C3orf36	NM_025041	1622	1614	0.0007
miR-1246	CALM2	NM_001743	1173	1164	0.0007

miR-1246	DCC	XM_005258204	5425	5415	0.0007
miR-1246	GNRH1	NM_000825	1996	1988	0.0007
miR-1246	GNRH1	NM_001083111	1127	1119	0.0007
miR-1246	MBNL2	XM_005254018	3086	3076	0.0007
miR-1246	MBNL2	XM_005254022	2996	2986	0.0007
miR-1246	MBNL2	XM_005254021	3050	3040	0.0007
miR-1246	MBNL2	NM_144778	3031	3021	0.0007
miR-132-3p	AHCY	XM_005260317	2221	2212	0.0007
miR-132-3p	AHCY	NM_000687	1997	1988	0.0007
miR-132-3p	AHCY	XM_005260316	2060	2051	0.0007
miR-132-3p	AHCY	NM_001161766	2161	2152	0.0007
miR-132-3p	C18orf63	NM_001174123	3780	3770	0.0007
miR-132-3p	C22orf39	NM_001166242	977	968	0.0007
miR-132-3p	DYNC1LI2	NM_006141	3825	3815	0.0007
miR-132-3p	DYNC1LI2	XM_005255831	3595	3585	0.0007
miR-132-3p	MYCBP2	XM_005266299	14991	14982	0.0007
miR-132-3p	MYCBP2	XM_005266300	14526	14517	0.0007
miR-132-3p	MYCBP2	NM_015057	14763	14754	0.0007
miR-132-3p	PBDC1	XM_005262274	805	797	0.0007
miR-132-3p	PBDC1	XM_005277805	805	797	0.0007
miR-132-3p	TJAP1	NM_001146018	2708	2699	0.0007
miR-132-3p	TJAP1	NM_001146020	2502	2493	0.0007
miR-132-3p	TJAP1	NM_001146017	2646	2637	0.0007
miR-132-3p	TJAP1	NM_001146019	2651	2642	0.0007
miR-132-3p	TJAP1	NM_001146016	2681	2672	0.0007
miR-132-3p	TJAP1	NM_080604	2703	2694	0.0007
miR-148a-3p	UGT1A1	NM_000463	2150	2141	0.0007
miR-148a-3p	UGT1A10	NM_019075	2172	2163	0.0007
miR-148a-3p	UGT1A3	NM_019093	2138	2129	0.0007
miR-148a-3p	UGT1A4	NM_007120	2167	2158	0.0007
miR-148a-3p	UGT1A5	NM_019078	2138	2129	0.0007
miR-148a-3p	UGT1A6	NM_001072	2271	2262	0.0007
miR-148a-3p	UGT1A6	NM_205862	1484	1475	0.0007
miR-148a-3p	UGT1A7	NM_019077	2126	2117	0.0007
miR-148a-3p	UGT1A8	NM_019076	2189	2180	0.0007
miR-148a-3p	UGT1A9	NM_021027	2163	2154	0.0007
miR-193b-3p	GART	XM_005260941	3473	3465	0.0007
miR-193b-3p	SPERT	XM_005266285	1835	1827	0.0007
miR-193b-3p	SPERT	XM_005266284	1517	1509	0.0007
miR-193b-3p	SPERT	NM_152719	1607	1599	0.0007
miR-194-5p	FAM208B	XM_005252475	8828	8818	0.0007
miR-194-5p	FAM208B	XM_005252483	8069	8059	0.0007
miR-194-5p	FAM208B	XM_005252481	8166	8156	0.0007
miR-194-5p	FAM208B	XM_005252482	8268	8258	0.0007
miR-194-5p	FAM208B	XM_005252480	8335	8325	0.0007
miR-194-5p	FAM208B	XM_005252477	8373	8363	0.0007
miR-194-5p	FAM208B	XM_005252479	8427	8417	0.0007
miR-194-5p	FAM208B	XM_005252474	8459	8449	0.0007
miR-194-5p	FAM208B	XM_005252478	8459	8449	0.0007

miR-194-5p	FAM208B	XM_005252476	8618	8608	0.0007
miR-194-5p	LPCAT2	XM_005256006	1120	1113	0.0007
miR-194-5p	NAA50	XM_005247794	3544	3534	0.0007
miR-29c-3p	ABCB6	NM_005689	2947	2939	0.0007
miR-29c-3p	ABCB6	XM_005246215	2777	2769	0.0007
miR-29c-3p	NASP	NM_002482	2822	2813	0.0007
miR-29c-3p	NASP	XM_005270889	1825	1816	0.0007
miR-29c-3p	NASP	NM_152298	1805	1796	0.0007
miR-29c-3p	NASP	NM_001195193	2630	2621	0.0007
miR-29c-3p	NASP	XM_005270888	2842	2833	0.0007
miR-29c-3p	RFX7	XM_005254603	6037	6027	0.0007
miR-29c-3p	RFX7	NM_022841	5949	5939	0.0007
miR-29c-3p	SERPINB9	NM_004155	1621	1611	0.0007
miR-335-5p	ADAMDEC1	NM_001145271	2229	2220	0.0007
miR-335-5p	ADAMDEC1	NM_001145272	2020	2011	0.0007
miR-335-5p	ADAMDEC1	NM_014479	2139	2130	0.0007
miR-335-5p	ARGLU1	NM_018011	1570	1561	0.0007
miR-335-5p	G2E3	XM_005267835	3277	3267	0.0007
miR-335-5p	NAA25	NM_024953	4156	4146	0.0007
miR-378a-3p	UTP18	NM_016001	1741	1733	0.0007
miR-708-5p	ABCA5	XM_005257177	5325	5316	0.0007
miR-708-5p	FCAMR	NM_001122979	1808	1799	0.0007
miR-708-5p	FCAMR	NM_032029	1891	1882	0.0007
miR-708-5p	MAPK1	NM_138957	1441	1433	0.0007
miR-101-3p	DDIT4	NM_019058	1116	1107	0.0008
miR-1246	COG6	NM_001145079	2392	2382	0.0008
miR-1246	CYP39A1	XM_005249171	1676	1668	0.0008
miR-1246	FHL5	NM_001170807	1070	1061	0.0008
miR-1246	FHL5	NM_020482	1301	1292	0.0008
miR-1246	PTPLAD2	XM_005251459	834	824	0.0008
miR-1246	ZNF155	NM_001260486	2016	2007	0.0008
miR-1246	ZNF155	NM_001260487	1899	1890	0.0008
miR-1246	ZNF155	NM_198089	1913	1904	0.0008
miR-1246	ZNF155	NM_003445	1990	1981	0.0008
miR-1246	ZNF155	NM_001260488	2002	1993	0.0008
miR-130a-3p	CBR4	XM_005263315	870	863	0.0008
miR-132-3p	C19orf47	XM_005258521	1221	1213	0.0008
miR-132-3p	FTSJ2	NM_013393	1140	1131	0.0008
miR-132-3p	SLC25A20	NM_000387	1655	1646	0.0008
miR-148a-3p	ATP6AP2	XM_005272573	1542	1533	0.0008
miR-148a-3p	ATP6AP2	XM_005272576	1239	1230	0.0008
miR-148a-3p	ATP6AP2	XM_005272575	1394	1385	0.0008
miR-148a-3p	ATP6AP2	XM_005272574	1470	1461	0.0008
miR-148a-3p	ATP6AP2	NM_005765	1491	1482	0.0008
miR-148a-3p	DOCK6	XM_005260000	6548	6540	0.0008
miR-148a-3p	DOCK6	NM_020812	6356	6348	0.0008
miR-148a-3p	DOCK6	XM_005260001	6455	6447	0.0008
miR-148a-3p	SIRT4	XM_005253864	1827	1819	0.0008
miR-148a-3p	SIRT4	XM_005253865	935	927	0.0008

miR-148a-3p	SIRT4	NM_012240	1123	1115	0.0008
miR-193b-3p	GART	NM_000819	3197	3189	0.0008
miR-193b-3p	GART	NM_001136006	3336	3328	0.0008
miR-193b-3p	GART	NM_001136005	3373	3365	0.0008
miR-193b-3p	TP53AIP1	NM_001195194	532	524	0.0008
miR-193b-3p	TP53AIP1	NM_022112	1210	1202	0.0008
miR-193b-3p	ZIC1	NM_003412	2362	2352	0.0008
miR-194-5p	CEP63	NM_025180	5748	5738	0.0008
miR-194-5p	CEP63	NM_001042400	4986	4976	0.0008
miR-194-5p	CEP63	NM_001042383	5020	5010	0.0008
miR-194-5p	CEP63	XM_005247798	5236	5226	0.0008
miR-194-5p	CEP63	XM_005247795	5374	5364	0.0008
miR-194-5p	CEP63	XM_005247796	5470	5460	0.0008
miR-194-5p	CEP63	XM_005247799	5489	5479	0.0008
miR-194-5p	CEP63	XM_005247797	5676	5666	0.0008
miR-194-5p	DOLK	NM_014908	2055	2047	0.0008
miR-194-5p	LYSMD3	XM_005248421	2050	2040	0.0008
miR-194-5p	LYSMD3	NM_198273	1924	1914	0.0008
miR-194-5p	TRAPPC8	XM_005258235	3800	3791	0.0008
miR-194-5p	TRAPPC8	XM_005258234	4972	4963	0.0008
miR-194-5p	TRAPPC8	XM_005258233	5062	5053	0.0008
miR-29c-3p	ARSB	NM_000046	4971	4961	0.0008
miR-29c-3p	ARSB	XM_005248505	4184	4174	0.0008
miR-29c-3p	ING3	NM_198267	820	811	0.0008
miR-29c-3p	LAMA2	XM_005266981	9877	9869	0.0008
miR-29c-3p	LAMA2	XM_005266982	9865	9857	0.0008
miR-29c-3p	RNF39	NM_025236	1991	1982	0.0008
miR-29c-3p	RNF39	NM_170769	1791	1782	0.0008
miR-29c-3p	TET2	NM_001127208	7099	7089	0.0008
miR-29c-3p	TET2	XM_005263082	6756	6746	0.0008
miR-335-5p	ALPPL2	NM_031313	1851	1842	0.0008
miR-335-5p	CPNE1	NM_003915	2141	2133	0.0008
miR-335-5p	CPNE1	NM_001198863	1911	1903	0.0008
miR-335-5p	CPNE1	NM_152926	1914	1906	0.0008
miR-335-5p	CPNE1	NM_152925	1956	1948	0.0008
miR-335-5p	CPNE1	NM_152928	2009	2001	0.0008
miR-335-5p	CPNE1	NM_152927	2041	2033	0.0008
miR-335-5p	G2E3	NM_017769	3332	3322	0.0008
miR-335-5p	HIST1H4I	XM_005249436	1135	1126	0.0008
miR-335-5p	KRT17	XM_005257349	1639	1630	0.0008
miR-335-5p	MBD1	NM_001204139	2308	2299	0.0008
miR-335-5p	SMARCA2	XM_005251558	5720	5711	0.0008
miR-335-5p	SMARCA2	NM_139045	5446	5437	0.0008
miR-335-5p	SMARCA2	NM_003070	5500	5491	0.0008
miR-335-5p	SMARCA2	XM_005251557	5605	5596	0.0008
miR-378a-3p	OR6A2	NM_003696	1250	1242	0.0008
miR-708-5p	CCDC85A	XM_005264123	1894	1886	0.0008
miR-708-5p	CRTC3	NM_022769	3014	3004	0.0008
miR-708-5p	CRTC3	XM_005254968	2969	2959	0.0008

miR-708-5p	CRTC3	NM_001042574	3011	3001	0.0008
miR-101-3p	CSRP2	NM_001321	775	767	0.0009
miR-101-3p	CSRP2	XM_005268678	762	754	0.0009
miR-1246	ITGAV	XM_005246536	6465	6455	0.0009
miR-1246	ITGAV	NM_001144999	5452	5442	0.0009
miR-1246	ITGAV	NM_001145000	5628	5618	0.0009
miR-1246	ITGAV	NM_002210	5736	5726	0.0009
miR-1246	NCMAP	NM_001010980	2545	2535	0.0009
miR-1246	PRRG1	NM_001173489	1267	1257	0.0009
miR-1246	PRRG1	NM_001142395	1157	1147	0.0009
miR-1246	PRRG1	XM_005272627	1174	1164	0.0009
miR-1246	PRRG1	XM_005277804	1174	1164	0.0009
miR-1246	PRRG1	NM_001173490	1176	1166	0.0009
miR-1246	PRRG1	NM_000950	1248	1238	0.0009
miR-1246	UNC13D	XM_005257137	3675	3667	0.0009
miR-1246	ZNF33A	XM_005252580	1341	1332	0.0009
miR-129-5p	CCDC169	XM_005266513	562	555	0.0009
miR-130a-3p	NOTCH2NL	XM_005277392	3167	3157	0.0009
miR-148a-3p	OPHN1	XM_005262270	4131	4121	0.0009
miR-193b-3p	NXF3	NM_022052	1814	1806	0.0009
miR-193b-3p	RPS6KB2	XM_005274165	1434	1426	0.0009
miR-193b-3p	RPS6KB2	XM_005274164	1555	1547	0.0009
miR-193b-3p	SLC25A45	XM_005273922	1074	1066	0.0009
miR-194-5p	BIRC6	XM_005264449	16046	16037	0.0009
miR-194-5p	BIRC6	XM_005264455	15914	15905	0.0009
miR-194-5p	BIRC6	XM_005264454	15935	15926	0.0009
miR-194-5p	BIRC6	XM_005264453	15977	15968	0.0009
miR-194-5p	BIRC6	XM_005264452	16004	15995	0.0009
miR-194-5p	BIRC6	XM_005264451	16019	16010	0.0009
miR-194-5p	BIRC6	XM_005264450	16034	16025	0.0009
miR-194-5p	C17orf112	XM_005256919	516	508	0.0009
miR-194-5p	CD44	NM_001202557	1969	1960	0.0009
miR-194-5p	CHMP4C	NM_152284	1714	1705	0.0009
miR-194-5p	LYSMD3	XM_005248422	1859	1849	0.0009
miR-194-5p	SEC31B	XM_005269676	4050	4041	0.0009
miR-194-5p	SEC31B	XM_005269680	2079	2070	0.0009
miR-194-5p	SEC31B	XM_005269679	3356	3347	0.0009
miR-194-5p	SEC31B	NM_015490	3685	3676	0.0009
miR-194-5p	SEC31B	XM_005269678	3764	3755	0.0009
miR-194-5p	SEC31B	XM_005269677	3916	3907	0.0009
miR-194-5p	YIF1B	XM_005259385	1423	1415	0.0009
miR-29c-3p	ADAMTS12	XM_005248381	6461	6451	0.0009
miR-29c-3p	AUNIP	NM_024037	1876	1867	0.0009
miR-29c-3p	COL3A1	NM_000090	4767	4758	0.0009
miR-29c-3p	LAMA2	NM_001079823	9601	9593	0.0009
miR-29c-3p	LAMA2	NM_000426	9613	9605	0.0009
miR-378a-3p	LPCAT4	NM_153613	1889	1881	0.0009

Supplementary file S5. Box Plot of normalizaed miRNAs count (Log) for DE-miRNAs (FDR < 10exp-6, LogFC>|2|) with homologies with human miRNA for different experimental group: preovulatory in non breeding season (pO-NBS), preovulatory in breeding season (pO-BS), antral in non breeding season (An-NBS), antral in breeding season (An-BS).

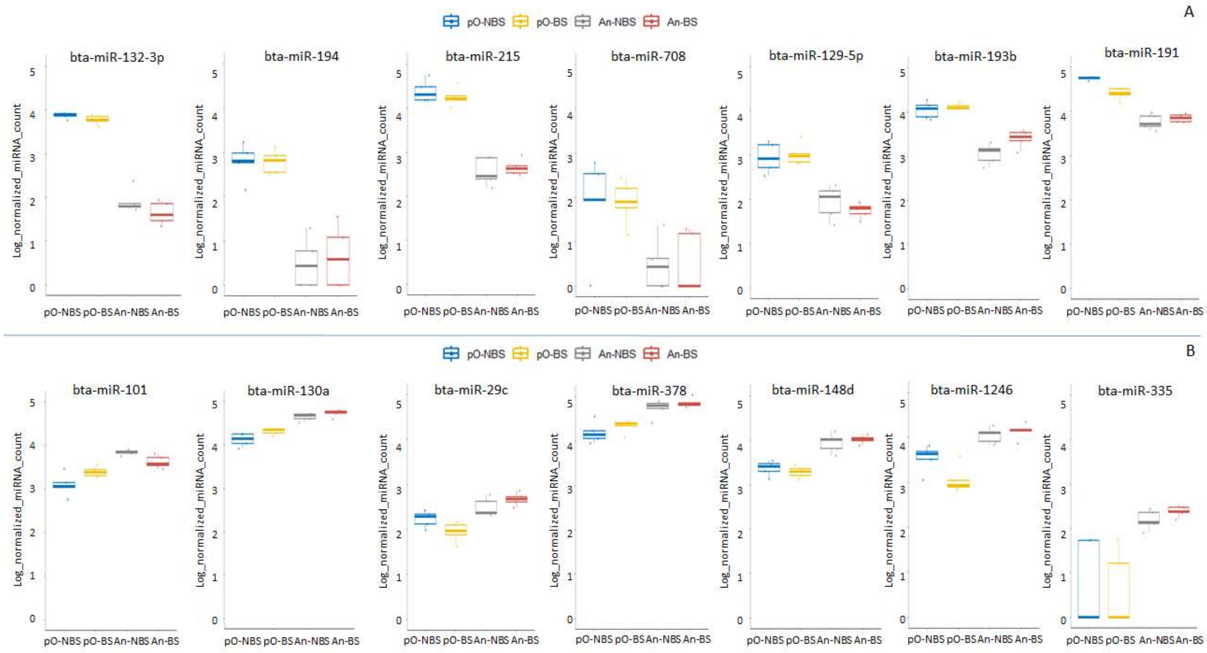

Supplementary file S6.
List of novel miRNA precursors

>Novel:hsa-miR-5193
GCCUCCUCCGUCGGCCUCUACACUCCGAGUGUGAGCGUCGGAGAUGCUC
>Novel:NC_037563.1_39396
ACACGGACAGGAUUGACAGAUUGAGAGCUCUUUCUUGAUUCUGUGGGUGGUGGUGC
>Novel:NC_037563.1_39878
CACAGAAUCAAGAAAGAGCUCUCAAUCUGUCAAUCCUGUCCGUGU
>Novel:NC_037547.1_10635
CAGAACUUAGCCACUGUGAACAAAGCGGAAACCAGUCACUGUUCACCAAUCAGCUAAGCUC
U
>Novel:hsa-miR-1237-3p
UGGCUUGCUCCGGGGUCACCCAGCCAGAGAGUGGGGAAGGAGACUCCACUCAGGCCUUCUG
ACUCCAAGUCCAGU
>Novel:hsa-miR-6832-3p
GCCCUUUUCCCCGCCCCUCCCAGCGCGGGGCGUGGGCGU
>Novel:chi-miR-202-3p
AGAGGUGUAGGGCAUGGGAAAAUGGGGCCCCGAGGUUCCCCUGCACCAGAGCCU
>Novel:NC_037556.1_30106
ACAGUCAACGGUCGGUGGUUUUUUCAAAUCCCAAACUCACCGACAGCGUUGAAUGUU
>Novel:NC_037549.1_15660
ACAAUCAACGGUCGAUGGUUUUGAUUUUUAAUUCCAAACUCACCGACAGCGUUGAAUGUU
>Novel:hsa-miR-2115-5p
GCAAACAUCUGACUGAAAGCUUAGCUGUGUGGUACAGCUUCCAGUCGGGGAUGUUUACA
>Novel:NC_037559.1_33968

CCUCAAGGAGCUUCAGUCUAGUAGGGGAUGAGACGUACUAGACUGUGAGCUCCUCGA
>Novel:NC_037549.1_16300
UGGCCUACAGAAAUGACAGACAGACUUAGUCACAUUGUCUGUCAAUUCAUAGGUCAU
>Novel:hsa-miR-3672
GGAGUGAUUUGUCAGGUUAAUUCUGAUAACAAAUGAGACUCUGGCAUGCUAAC
>Novel:NC_037568.1_45248
AGCGGGACUUUGUGGGCCAGUUGGAUAAAACAUAAACUCAUCUCGCCCUCAAAACCCCG
>Novel:NC_037567.1_44073
CGACCGACGCCACGCCGAGUCGAUUGGCAAUAUAGACGAGCCGGUCGAGGUCCGGUCGA
>Novel:chi-miR-2284a
GAAAAGUUUGUUCGGGUUAAUCUGUACCAUCUUAUGGAAAAAUCUGAACAAACUUUAU
>Novel:NC_037569.1_46559
AGCCUUGGGUGUGGGAGGGUAAUUAAGAUGCCAACGGUCCUACACUCAAGGCAUG
>Novel:NC_037568.1_44905
AGUGCUGGCUCAGCAGUAGGUCAGGUGAUCACACUACCUGCACGAACAGCACUUUG
>Novel:NC_037560.1_35426
UCAGUUCAGUCGCUCAGUCGUGUCCAACCCUAGACUGCGCGCCCUCAGAGCAGAC
>Novel:NC_037569.1_46961
AAGAGUUUGUUUGGGUUUCUCCAUAAGCUGUAUAGAAAAACCUGAACGAACUCUUU
>Novel:NC_037565.1_42672
AGGCAGUGGCCUGUACAGUUAUCUCCUGUACCGGGUGGUAUCAUAGACCCUCAAACUGUAC
AAACUACUACCUCA
>Novel:NC_037547.1_10214
GGCAAUAUGUUGGCAUAGCAGGUUCGCAGUUCAACAGCUAUGCCAGCAUCUUGCCU
>Novel:NC_037547.1_8560
AGAGUUCAUUCAGGUUUUUUGGUAAGAUCUUACAGAAAAACCUGAAUGAACUCUUU
>Novel:NC_037554.1_24951
AAAGAGUUCAUUCAGGUUUUUCCAUAAGAUGUUGUGGGAAAAAAAUGGAAUGAACUUUUU
GG
>Novel:NC_037550.1_17628
GCUCCCCACAGACCCAGAGCCGAAUUUCUAAGGUAUCCGGGCUCUGGUGGACUGAGGGGGG
CGACGC
>Novel:NC_037555.1_27844
GGCAUAGCGCACUGCAGCCCAGAACUCCUGAGCUCAAGCGAUCCUCCAGCCUCAGCCUCCCG
AGUAGCUGGGACUACAGGCGCGCGCCACC
>Novel:NC_037547.1_7559
UUGAGGUUAGUGGCAACAGCAUGUGCGUGGUUUCCGGGAAACCUUCAGUGAGCCUGCUGUU
AGCCCUAGCCCCGCA
>Novel:hsa-miR-6741-5p
AUGGGUGCGAUUUCUGUGUGAGACAGUUCCAUUUACCGUGUCCUCAACCACAGAUAGCACC
CCU
>Novel:NC_037563.1_39679
AAUCAGGAAAUGGAUGGCGUUUUUCAAAUCACAUGAAUCAGCGCCCCAGAAUUCAUCAUUU
UC
>Novel:NC_037569.1_47087
AGCAGCGCCUCACGUUUUGAACCGUUUAAAGCACUUCAAAACAUGAAUUGCUGCUG
>Novel:hsa-miR-548a-3p
AGAGAGUUCAUUGGGUUUUUCCAUAAGAUGGUACAGAAAAACUGAAUGAACUUUCUGG
>Novel:NC_037567.1_44260
CCAAGUCCAUCCUUCAGCUCUACAGUCCUCACCAACUUGGAGAGCUGAAGGAUGGACUUGG
>Novel:NC_037567.1_44693
CCAAGUCCAUCCUUCAGCUCUCCAAGUUGGUGAGGACUGUAGAGCUGAAGGAUGGACUUGG
>Novel:hsa-miR-652-5p
CAACCCUAGUGGCGCCAUUCAAUUCUAGUCUAUGUGAAUGGCACCCUCUCCUAGGGUUGU
>Novel:NC_037569.1_46992
UCUCUCAACCAACCAGAUGUUUGUCUGCAUCACCUCAAACAGCUGGAUGGUUGACG

>Novel:NC_037548.1_12197
AAAGAGUUCAUUCAGGUUUUCCCAUCUAUAUGAUAUGUCCUAAAAACCUGGAAUGAACUUU
UUGG
>Novel:NC_037556.1_29844
GUGCUGUGAUGAUGCCUUAAAAUUGUGGUUUCGACUCACUGAGAGUAACAUGAAGACCUAC
AAUUCCUUGGCUGUGUCUGAGCACC
>Novel:hsa-miR-12136
CAAAAAGUUCGUCCAGAUUUUUGUAAUAUUUUAUGGAAAAACCUGAACAAAUUUUUUGGC
C
>Novel:NC_037560.1_35403
GACUCAGAUCGAAGCGUGCCUGCAGAGUUGGGAUUGGAUACUCAGGCCUCGUCUUGUCUGA
GGAA
>Novel:NC_037546.1_5830
AAAGUUCGUUCCAGUUUUUCUGUAACAUCUUAUGGAAAAAUCUGGACGAACUUUUUG
>Novel:NC_037564.1_41423
AGAGUUCAUUCAGGUUUUUCCACAAAAUGUUACAGAAAAACCCAAACAAACUUUUU
>Novel:NC_037547.1_8583
AAAGUUCAUUUAGGUUUUUCUGUAGGAUGCUCUGGAAAAACUCAAACCAACUUUU
>Novel:NC_037551.1_19765
AAAAAAUUUGUUCAGGUUUUUCCAUAAAAUAUUACAAAAAUCUGGACGAACUUUUUG
>Novel:NC_037548.1_12259
AGGGUCAAAGCCCCACUCCCCACAGAAGGUUUUCCUAAAGGAGAUGGAGCCAGGGCCCUAA
>Novel:NC_037552.1_22398
AGGAGCAAUAAUGAAAAACGGUUUAUGACAAACAUUUUUCGUUAUUGCUCUUGA
>Novel:NC_037545.1_1192
AGAGUUCAUUCAGGUUUUUCUGUAACAUCUUACAGGAAAACCCAAACAAACUUUUU
>Novel:hsa-miR-6776-5p
UCUGGGUGAGCAGUCGGAGACUCCGCAGUGCUCUUCCUGUCGGGACGGGAUCGCUUUCUCU
GGCCAUCAACACCCCUGGAG
>Novel:hsa-miR-5190
ACAGUGACCAGGUGACGACGGAUUUCUCAAGUGACAACCUCGUGGCUUGGUCACAGUGU
>Novel:NC_037557.1_30647
CAAGUGCCUUCACUGCAGUAGAUGCACAUAUCACUACCUGCACUGUAAGCACUUUG
>Novel:hsa-miR-6125
ACGGAAGGGCAGAGAGGGCCAGGGGCAGCACACACUAUCUCCCUGAGCCCCUCCUGCCCCCC
U
>Novel:NC_037565.1_42254
GGACCCGGGAGCACAGCCCAGGGCAGCACUCCCCACCCGGGCAUCUGCGCCCGAGUCU
>Novel:hsa-miR-4691-3p
GGGCUCCCUCUGGGGGCUGCCGUCCCGGCUCAGAGCAGCCACACAGAGCGCCCGG
>Novel:hsa-miR-6499-5p
CCGGGCGCUCUGUGUGGCUGCUCUGAGCCGGGACGGCAGCCCCCAGAGGAGCCCUGGCCC
>Novel:hsa-miR-6777-5p
CCGGGGAGCCCGGCUGGCCCUUCUGGCCUGGCAUCCCUGGCA
>Novel:NC_037555.1_28111
CCAAAAAGUUCAUUUGGGUCCUUCCAUCUGAUGUUAUAGGAAAAGCCCAAGCGAACUUUUU
>Novel:chi-miR-3432-5p
UGCGGGAUCUUUAGUUGUGGCGUGUAAAUUCUUAGUUGCAGCAACUAAAGAUCCCUCAGG
>Novel:hsa-miR-1290
AGGAUUUUUAGGGGCAUUAUGAUAGUGGAAUGCAAACACAUCUGCCCCCAAAAGUCCCU
>Novel:NC_037545.1_169
AAAAGUUUGUUUGGGUUUUCCCAUAUGACGUUAUGAGAAAACCCGAACGAACUUUUU
>Novel:NC_037567.1_44300
AAAAGUUGGUUUGAGUUUUUCUGUAACAUCUUAUCAGAAAAAUCCAAGCGAACUU
>Novel:NC_037567.1_44728
AAGUUCGCUUGGAUUUUUCUGAUAAGAUGUUACAGAAAAACUCAAACCAACUUUU

>Novel:NC_037553.1_23200
GACCUGGCUCCAGCCCAGCCCAGCAGCUUCAGAGAUGGACCUGGACAGGAGCCUUCA
>Novel:hsa-miR-762
UGAGGAUGCCGGCCAGCCUUGCCUCCUCAGCCUCGCUGGGCUGGCCUGCCUCCAAGC
>Novel:NC_037549.1_15917
CAUCGUUACCAGACAGUGUUAGAGUCGAGCCGAGAAAUCCAGCACUGUCCGGUAAGAUG
>Novel:NC_037553.1_24045
AAGUUCCUUUAGGUUUUUCCAUAAGAGCAUAUGAAAACCCAAACAAACUUUUU
>Novel:hsa-miR-760
GUGGGCAGAGGGGCAUCUGUCUUUGGAAUUCUAGGCUCUGUCCCUCUGUCCAAUU
>Novel:hsa-miR-1470
GCCCUCCGCUUCUGGUCCUACCGGGCGCAGAAAGCCCGGAGGUAGC
>Novel:hsa-miR-3919
UCAGAGAACCGUGCCGAGGAGGCUCAGCGCGGUUCUUUA
>Novel:NC_037549.1_16296
AGGCUGGGAGAUAAAUGAAUAGAAUAAAAUUUUCUAUCCAAUCAUUUCUCCUAGCCUG
>Novel:NC_037557.1_31157
ACUUUCCCGGGACUUGGAGCGCUUCCGCACGCGUUCCUUGUCCCGAGAGCGCGA
>Novel:NC_037552.1_22185
AUCGCAUCAACACUCGUCCGUUCUUAAAUAACAUUGAUAACAGGCGGGUGCUGAUACGAUC
A
>Novel:hsa-miR-3670
AGGGGCAGGAUGUCUGAGCUCACUCAGCAAGAGGAAGGAGAGCUCAGUGCCUACCCUGCA
>Novel:NC_037556.1_29648
GAGGACUCAGCAGUAGGCUCUGUGAGAGACCCUCCAGAGCAGGCUGUGGAGACCAAGA
>Novel:NC_037550.1_17830
AAAGUUCAUUUGGGUCCUUCCGUUAAGAUGUAUGGAAAACCUGAAUGAACCUUUU
>Novel:NC_037550.1_18986
AGGUUCAUUCAGGUUUUCCAUACAUCUUAACGGAAGGACCCAAAUGAACUUU
>Novel:NC_037561.1_36221
AGCCUGUCAUGGCCCUGGCCUUGGACCCGCGGAGGGCCAGGGGUGUGUCGGGCUCU
>Novel:NC_037549.1_14209
CACUGCUUGGACCCGACUCUGAUCGCUUCUACAGAGAAGGGCUGCUAGCAGGAAG
>Novel:NC_037556.1_29299
ACCAGCCCGGAGCCUCUGGGGCAGCGGUGACGGCCACAGCCACCACUGCCGAGUGCUCCGUG
GGCUCCGGGCUGGGGU
>Novel:chi-miR-214-3p
UCUGUGUUCUUGGCCUGUGUGUGUCUUUGAACCACUCCACAGCAGACUCAGGACACAGA
>Novel:hsa-miR-548c-3p
AGCUCGUUUGGGCUUUUCCGUAGGAUCUCCUGGAAAAAUCCGAACGAACUUUC
>Novel:chi-miR-499-3p
ACAGACUUGCUGUGAUGUUCACGUGGAGAGGAGUUAAACAUCACUGCAAGUCUUAA
>Novel:NC_037564.1_41137
AAGAUGAAGGAACUCAAGAGAGCAACUUUCCUCUCUUGAGUUUCUUCAUCUGU
>Novel:NC_037552.1_21285
UCUCCUGGGGUCUCACCUUAGCAUGUCCUCAUUAAUGGUGUUAAGGUUUCUCCAGGUGAC
>Novel:NC_037546.1_4277
UCUCCAAACAGCAAGGACAAUGCCACACAAUUAUAAUAGCAUAUUGUCCAUUGUAUCUGGG
GAU
>Novel:chi-miR-2284d
AAGUUUGUUCCGCUUUUCCCAUAAGAUAGUACAGAAAAACCUGAACAAACUUUU
>Novel:NC_037550.1_18493
UCCGGGAGUGUAGGGCGCGCUCAAGACGGGAGGACCUUAGAGCGCGCAAAGCUCUGGACUC
CC
>Novel:NC_037546.1_6178
AGAAGGUUCAUUUGGGUUUUUCCAUAACAUUUUAAAGAGAAACCCAAACGAACUUCUU

>Novel:NC_037569.1_48167
UCGCCCCGCCUCUCUGCACGACACGCACGGGGCGGCGCACAGGUGGGCGGGGCUU
>Novel:chi-miR-326-3p
UCUCUGGGUCUGCUCAGCCCACUGAGGCACACCGAGUCCAGUGAGCCCUGGAGGCCCAGCC
>Novel:hsa-miR-3620-5p
GGCUGGGCCUCCAGGGCUCACUGGACUCGGUGUGCCUCAGUGGGCUGAGCAGACCCAGAGA
>Novel:NC_037564.1_40643
CCCCCGCCCCGCCCCCUCCGGUGGGCUGCGGGGCGCGGCGCGGCGCGGC
>Novel:NC_037550.1_17382
UCCGGGAGUGUAGGGCGCGCUCAAGACGGGAGGACCUUAGAGCGCGCAAAGCUCUGGACUC
CC
>Novel:NC_037549.1_16572
AGAGACGUGAUCUUGUUUUGGAGUCUGGGUUGAACAAAACAAGGUCACGUCUCUCA
>Novel:NC_037549.1_15054
UGAGAGACGUGACCUUGUUUUGUUCAACCCAGACUCCAAAACAAGAUCACGUCUCUUAG
>Novel:NC_037553.1_24733
UGGGCGGAGGUGGGGCGGGGGGCCCCCAAAUGGGCUUGCCCCCCCCUCCCCCCGCC
>Novel:NC_037555.1_27691
CCUCCGCCCCGCGCUCGCCUCGCGCACAGGCACCGGCCGGCGGGCGCGGGGAGCGGAGAGC
>Novel:hsa-miR-4695-5p
AAGGAGGCUGGGUUCCUGCUCAGUGGUGCCUGCAGGAACCCAGGCUCUCUUCAGU
>Novel:NC_037547.1_8809
GUCUUUUUUUUUUUUUUUUUUCCCAGCUUCUACUCUUCUAUAAGCUUGGGGAGUAAGAAGA
GGCUGUGUCAAUCU
>Novel:NC_037554.1_25908
AAAGAGUUCGUUUUGGUUUUUCUGUACCAUCUUAUGGAAAACCCAAACGAACUUUC
>Novel:NC_037564.1_40769
UUUCGGUUAUCUAGCUUUAUGACGGCUCUGUGGCACUCAUACAGCUAGAUAACCAAAGA
>Novel:NC_037553.1_23966
UUUCGGUUAUCUAGCUUUAUGAAGACCAAUACACUCAUACAGCUAGAUAACCAAAGA
>Novel:hsa-miR-4779
CAGGAGGGCAACACGGAGCUCUGACCUUGAGGGGCUUCUGCUCUGCCAGCC
>Novel:NC_037555.1_27132
GCUUCUGCAGACUGGCUCCAAUUACAGAUAUGUAAUUGGAUAUGUAAUUGGGGCUUAUCUA
CAGAAGUC
>Novel:NC_037550.1_18370
UUUCGGUUAUCUAGCUUUAUGAAGACUCCACACCACUCAUACAGCUAGAUAACCAAAGA
>Novel:NC_037560.1_35002
UUCCGGGCAGCGGGCUGAGGCGGAAAUGCACUCACGUCUCAGGAGCUCGCCUGGACGGG
>Novel:NC_037558.1_31888
GACUGGGCUGAAGGAGCCCCCCUGUCUGAUCCUCACCCAGGAGCUCCUGGAGCCCAGUCCGC
>Novel:NC_037567.1_44673
CACGUCCUCUGACCACAGAGGCUAUGGCUAGAGAUCUCUUAGGUCCUAAGGACGCC
>Novel:NC_037555.1_26952
UAGGGGCAGUCAGGGACCUGGAUUUCUAUCCCUAUCUUAAUCCCCGACUGUCCCCUCC
>Novel:hsa-miR-6071
UUCUGCUGUACAUUCUGCCAUCCUGAUAAGAUGAUAAGAUGACGGAAUAUACACCAGAAA
>Novel:NC_037556.1_30278
CCCUCAGAGGGCGACGGCCCUGGCAUUCUGAGCAGCUCAUGGCAGUCGCCCGCUGUGGCCA
>Novel:NC_037547.1_10894
AGCCCAGGUCCCCGGCUCUGGGGCGCUUAGAACUCUCAGAGCCCAGGUCCCCGGCUCU
>Novel:hsa-miR-629-3p
ACAGGGCGCCCGGCGCGCCUCGGAGCGCACGUUCCGCGCUUUCUCCCGCCGGCUCCCUGGAC
>Novel:NC_037545.1_1343
UCAGUAACAAAGAUUCAUCCUUGUGUCCAUCAAGCAACAAGGAGAAUCUUUGUCACU
>Novel:NC_037546.1_4436

AAAGUUCGCUUGGAUUUUUCCAUAAGAUGAUAUGGAAACCCCAAACGAAUUUUUU
>Novel:hsa-miR-4663
GGUGUUGUUUCUUUCAGCUUUUAGCUGAGCGAUCUCCUCU
>Novel:NC_037558.1_33220
GAGACUCAGAGGUGCCAAAAGCCAAUCGCCAGCUCUUGGCCUCCUGCGUCUUU
>Novel:NC_037549.1_14776
GGCGCCAGCCGAGGGGGGCCUCUCCAUUCAUUCCCUCAUUAGGGAUGCGGGGCCGCCUCCUC
CUCCUCCU
>Novel:NC_037569.1_46509
CCUGGGGCUCUGUACUCCCAGAUGUCAGGUAGAUGCUCAAACAGAGUGCUGACGGGUCACU
CAGAACCCCGAGU
>Novel:hsa-miR-4270
AGGGGGUGGGAGGUUUUGGGCAGGGGGUGAUAAGUCCCCUGGGCAGGGAGCAGUAAUUCCC
GGG
>Novel:hsa-miR-4707-5p
GCGAGAGGCCGCGGCCCUGAGCGGCUGUGUGACCGCACCCCACUCCCACCUUGCAGGUUCAC
CCUCUGCUCCCCGGCACCUCCACCA
>Novel:hsa-miR-1199-3p
CAGGCGGGCUUCCCAGCCGCGUCCCACCUGCGCGGCCGCCCCCGCGGGC
>Novel:hsa-miR-4778-3p
GCCCCGCCCGCGGCUGGAGGAAGCGGCCGCCGCGGCUCACGCUUCUUCAGCCCGGGCGGCCU
>Novel:hsa-miR-4707-3p
AGCCCGCCGCAGCCCGUCGUCAAUGGCAGGCCAGAAUCUGGGGGCAGGGUACGGAGGGAGG
C
>Novel:hsa-miR-10226
UUGGGGAUUGAGGCCACAGGUCCCGGUUCCCCAGUUGGUCAUGUGUGAUCUGUCCCCAGG
>Novel:NC_037561.1_37446
AGUAUCGCAUCGACUUGGAGCAGUUUAAGGGCAUGUUUGCUCUGCUCUUUCCCUGGGCCUG
CGGAACU
>Novel:hsa-miR-765
AGGAGGAGCUGGAGCGGCUGGAGGAGGCCUGCGACAUGGCGCUAGAGCUCAACGCCUCC
>Novel:chi-miR-2331
GUCAGCCCCGGGGCAGCCCUCAGGCUGCCCUGCAGUCCUGGCCU
>Novel:hsa-miR-1253
AGAGAAGAAUGUUGAGCUAGUGAACACAGGUUGUGUUUACACACACACAUACAUAUUUAU
>Novel:hsa-miR-6834-5p
GUGAGGGACUGGGAGAGGUGGCUCUGUCCCUCGAGU
>Novel:chi-miR-500-5p
GAAUCCUUGCCCAGGUGCAUUGCAUGGAGACAGCACUAGCACCCAGAGAGCAAGGAUUAG
>Novel:hsa-miR-7704
CCCCAUCCUGGCCUCGCCCCUUGUUGGCUCUGAUCUCUCUGAGCAGGAUGUAGGGGUCGGG
GGUCGGGGGGCGGGC
>Novel:hsa-miR-4691-5p
GUCCUCCAAGGUGCCCUUUUGUCAUCAAAGAGCAAAACAAAAGGGCACCUUGGAGGAC
>Novel:hsa-miR-1827
CACUACACCCCGCUGCCUCUCGUGGAGAAGAGGCAGGGUAGUGUAAUGGA
>Novel:hsa-miR-619-3p
GACCUGGAGCCCGACCGGGGUUGCAGAGCAAACAGCCAGGUCUGGGCUCCAGCCCCUUG
>Novel:hsa-miR-4514
UCAGGCAGCUAGAGCCGGCGCCAUGGUGGAGAAGGAGGAGGCGGGCGGCGGUAUCAGCGAG
GAGGAGG
>Novel:chi-miR-1307-3p
ACUCGGCGUGGAACCCGGCGGUGGCCGGGCCCUCGCCCUUGUCC
>Novel:hsa-miR-5004-5p
CCCAGGGCCUGUCCCCCAGAGCCAGCUCCAUGCUGUGGUGUGAGAGGACAGGACUGGGAC
>Novel:hsa-miR-591

CGACCAUGGCUGUAGACUGUUACCUCCGGUUCCCACAGUAACAAUCGAAAGCCACGGU
>Novel:hsa-miR-4675
AGACUCGGGCGCAGAUGCCCGGGUGGGGAGUGCUGCCCUGGGCUGUGCUCCCGGGUCC
>Novel:hsa-miR-3074-5p
GUUCCUGCUGAACUGAGCCAGUGUGUAAAAUGAGAACUGAUAUCAGCUCAGUAGGCACCG
>Novel:hsa-miR-4447
GGUGGGGGGAGGCGGGGGAGACAGGCCACGCACAGUCACUACAAAGAAUAUGGCCUCCUGU
CUGUUCACUUCCCAUCGUGAUUUU
>Novel:hsa-miR-6825-5p
UUAGUGCUGUGAUGACCUAAAGAGGGGAGGACGGAGGAGG
>Novel:chi-miR-125a-5p
GCCCUGAGUUCAGGCAGCAGUUCAAUGUUCAGCAAUGUCCUCUGAACUGCUGCCCAAACUC
GGGACUU
>Novel:oar-miR-134-3p
GCCAGGCCCCAGCCCUGCCUUGCUGGGCUGGGCUCUGGCCU
>Novel:hsa-miR-6728-3p
GACCUCGCUGCGGGCUCACCUUGAAGUACACAGUGCCUCGCUCCUUCACUAUGGUGCUCUG
CUCCAGCUUCUCCU
>Novel:hsa-miR-6755-5p
CAGGGUAGGCACUGAGCUCUCCUUCCUCUUGCUGAGUGAGCUCAGACAUCCUGCCCCUAG
>Novel:hsa-miR-4520-3p
AUGGACAGAGGAGCCUGGUGGACUACAGUCUCAGUUCACUUGCUCAGUUGUGUCCGACUUU
UUGCAACCCCAUGGACUGCAGCACACUAGGCUUCCCUGUCCGUCA
>Novel:hsa-miR-3654
GAAAAUGCAGACUACGAUGAGGGCCUCACUGGACUGCAAGAAUAC
>Novel:hsa-miR-4720-5p
ACUGGCAUCGUGAUGGACUCCGGUGACGGGGUCACCCACACGGUGCCCAUCU
>Novel:hsa-miR-6715a-3p
ACACCACAGUGUGGUUUGGACGUGGCCACAGUGCCGUACAAACCACAGUGUGCUGCUG
>Novel:chi-miR-130a-3p
CAGUGCAACUUCCGGGCCUCUGGCUUCGCCCAGUCCCAGGCGCUACGACUUCCGGCCCCGAA
GGUCCCCUGCU
>Novel:hsa-miR-2110
CUGGGGAAAGCAGGUGAGGGUCUGCACCCCAGUAAGCCUCGUUCUGCUUUCCCCACAGGCA
>Novel:hsa-miR-203a-3p
GGUUCUUAACAGUUCAACAGUUCUGUAGCGCAAUUGUGAAAUGUUUAGGACCAC
>Novel:hsa-miR-4746-5p
UCGGUCCCUCGGGCCAGGGCAGGAAGCGAGUCUCUCUUACCCUGGUGCGUGGGGCCG
>Novel:hsa-miR-548v
AUGGACAGAGGAGCCUGGGGGAUGGGCUACAGUCCAUGGGGU
>Novel:hsa-miR-759
ACCCUGCCCUGCGCUCCCUUCAGUAAGCAUACAGACAGAGUGCAGACCAGGGUCU
>Novel:hsa-miR-372-5p
GCUCAAAUAUACAGCUUGUUCUUGUCAGUUCUAGCUGACAAGAACAAGCUGUAUAUUUGAA
GUU
>Novel:hsa-miR-3064-5p
UCUGGCUGGCUGGCGCGGGCUCCGCUGUCUUCUGGGUCCUGGGUGAGUACAGGGUCUGGGC
AGCGCUGUGGGCGGUGGGGGCCAGGUG
>Novel:chi-miR-103-5p
AGCUUCUUUGGCUGCAGGGUCUCAGCCUUGAGGAAUUCACAGACAUCUGCAGGCACAGAAG
CCAC
>Novel:hsa-miR-486-3p
AGGGGCAGAGGUGGACUGACAGUCUGUCAAAUACUAAGACCAGAUACCAGGCCACCCUCUG
CUCCUCC
>Novel:hsa-miR-6077
AACCAAGCUUGCUUUUCUUUUUGUUCCGAGUAGGUAAAGGAAGAGACCUGUGGACCU

>Novel:hsa-miR-12135
GAAAGGUUCGUUUGGGUUUUUUGUAAGAUAUUAUAGCAAAACCUGAGUGAAUUUUAGGC
>Novel:hsa-miR-198
GGUCCAGAGUUGCCAGAGGAGUGGCGUCUGGACCAG
>Novel:hsa-miR-5681a
CCUUGGUUGUUUCAAUUUUGGUAUGGGAGAAAUGUCUCUAUUUCUUACUAAGAAAGGGCU
GCUGUGCC
>Novel:hsa-miR-6770-5p
GGAGAAGGGCUGGGGCCUUGGGGGGUCUUGUCCACCCCA
>Novel:hsa-miR-9900
UCGAAAAAGAGCUUUGAGUGCUACUUGCACCCUCAGGUCCCUGUUCGGGC
>Novel:hsa-miR-548as-3p
AAAAAGUUUGUUCAGGUUUUCCAUACCACUUAAAGGAAAAACCCAAACGAACUUCUU
>Novel:hsa-miR-4434
AGGAGAAGCUGUGGGUGCUGAGUAAGGCAGGAAUGUCAGGGGCCCGCUCUCGCAACAUCGU
CUACUUCUCCGUC
>Novel:hsa-miR-5088-5p
AAGGGCUCGGCGGCGGUUGCCGCUGCUGCCGCCGCCGCCGCCCACCA
>Novel:hsa-miR-4442
UCCGGACAGCAGAGGCCCCGCCUGCAUGUGAUCUCUCCGUGAGCAAGCAGGCCACUGCUGU
CCACA
>Novel:hsa-miR-3909
GGGGAUGAGAAUGGAGAGGUAUCUAGUUGCGCUCAAAGGUCCUCUCCAUCCUUACCCCAU
>Novel:hsa-miR-7106-5p
AGGGAGGAACUGGACUCCCUAAGGAAAUUCUCAUUUGUUACUAAGGGAAAGUGAGGGAAA
AGGUUCCUCUGUCAACUCC
>Novel:chi-miR-874-5p
UGCAGGGCAUUGGUUGUCUCAUUGGAGGCCCCACUGUCACUGAUAU
>Novel:hsa-miR-6868-3p
UUCCUUCUCCCCCUCUCCCCUCCACAGUGCAGAGAAGGGAGUGCAAAGG
>Novel:chi-miR-127-3p
CCACCUCGGGGUUCCUUGCUGCCCGGAUCUGCCCGGAUCCCCGAGUCGGGC
>Novel:chi-miR-758
AUUGUGACCACGCUGCAGGACAGUAUCCGCAAAGUGGUCAGUGG
>Novel:hsa-miR-4677-3p
UCUGUGAGCUGUGCUUGCCGGGUCAAUCAGCCAAUAAGCCCGACAGUCAUUGCUUACACCC
C
>Novel:hsa-miR-21-3p
UUUAGUGUGAUGAUGGCGUUUGAUAGUUUGGACACAAACACCAUUGUCACACUCCA
>Novel:hsa-miR-3974
AAAGGUCAUUCUGAAGCCUUUGGAAAACAAGGCACACUGAAGUGACCCAGCCGGUGGGCUC
AGAAUGGCCAGCAU
>Novel:hsa-miR-10522-5p
AGAAGAAUGAGGACAGACUGAGGCCGCCAGCACUGCCGCGUCUGUCUCUCAUUCCUUUCGA
C
>Novel:hsa-miR-548d-3p
AAAGGGCCCUCAGGUUUUUCUGUAAAACGUUAAUGGAAAAACCCGAACGAACUCUUU
>Novel:chi-miR-411b-5p
UGGUCGACCAGUUGGAAAGUAAUUGUUUCUAAUGUACUUCACCUGGUCCACUAGC
>Novel:hsa-miR-3202
AGGAAGGGGAGCCAAGGGACCAGCCUGCCUCAGAACGGACCCCUCUUCUGGGCUCCAGCCCC
ACACCUCCCUCCU
>Novel:hsa-miR-7845-5p
AAGGGACAGAGCCACUCUAUAGUGGGUUUUCUCUUCU
>Novel:chi-miR-490
AUGUUCAUUCGGGUUUUUCUAAAUCAUCUUACAGAAAACCUGGAAUGAACUUUU

>Novel:chi-miR-34a
GACUCACCUCCACUGUUACCAGGCUGCGGGCGAGGAGGCAGUGGCUUCGUCCU
>Novel:hsa-miR-766-5p
GACUUGCAGCAGUGACCUGGGUGACAGAGGAGGAAGCUGAGGCCU
>Novel:hsa-miR-6814-5p
ACCCAAGGACUGUAGCCCGUCUGGCUCUCAGUCCAUGGGUUU
>Novel:hsa-miR-4491
UAUGUGGAAGCUGACCAGAGAUGAGAUGAACUAUGUUUUCCCACAGUGUGCUCUUUGGGGU
CACUCUCACUUUGU
>Novel:hsa-miR-4753-5p
CGGCGAGGGUAGCUGAGGCCAGGCCAUCCAGUAUGCGAUUUAAGGGCCAACAAGGCCAGCU
AGCUUCCCGAG
>Novel:hsa-miR-6501-3p
GCAGAGCAGGGAAGGUGUGUUUAGCGCUCACUACCCAGCACCUGCCCAGCCCACUC
>Novel:hsa-miR-10399-3p
CUUAUCUCUCUGCUUCAGCGGACACCCCUGUGAUCUCGGAAGAGAAAGGGC
>Novel:chi-miR-432-5p
GCUUGGAGGCAGGCCAGCCCAGCGAGGCUGAGGAGGCAAGGCUGGCCGGCAUCCUCA
>Novel:hsa-miR-5002-5p
CAUUUGGUAGAGCAUCAGAUUUUUAAUCUGAGUGCCUAGGGUUCAAGUCCC
>Novel:hsa-miR-4482-3p
GUUCUAUUUUGUUGGUUUUUGAAACUGAGACCAUAAUUAAGAAGGA
>Novel:hsa-miR-4641
AAAGAGGCCAUGCCAGGGCAGCAGGAACUCUGGCAGCUGCCCAUGGACUGUCCUCUUCC
>Novel:hsa-miR-505-3p
GGUCAACAUUCCAACAGCCUGGGGUUUUCAUUCCAGCACCAAACUGUGAGGGAUGUGGACC
CG
>Novel:hsa-miR-3122
AUUGGGACUCCGAGCCCGGGCUAUUUCUGGCCCUGGCGCGGCUCCAAGAA
>Novel:hsa-miR-149-3p
AGGGAGGGAGGAGUCAGAGCUGAGGGAGAGGAAGUCUCUUUGGGAGGUGGGAUUGCUGGG
CUCUCUUU
>Novel:chi-miR-22-3p
AAGCUGCCAGUUGCAGAAAUCCUCCAAAGCCACUGAGACGUGGCUCAAAUUUUAGCCCCAC
UUCCACAGUCAUU
>Novel:hsa-miR-136-3p
GCGUCACCACGUGAUGACGCUGUGCCGUCACACACAUCAUCGCAUAUUGACACAA
>Novel:hsa-miR-6765-3p
UGGACUUUGAUCACCCCAGGACCUUUGACACCUGGGGGUCUGAGGGGC
>Novel:chi-miR-320-3p
CAAAGCUGGGUUGAGAGGUGGGUUGGCAACAAUGAUGAAAGCAUCUUUCCAAUGCCAACUG
UUACUCAGCCCCUCGCUGCACAGAG
>Novel:chi-miR-145-3p
AUUCCUGGGAAAACUGGACUGGCUCAGGGGCAAACAGCCUUAACCAGGCAAAUCUAGUUUC
UCCUAGAUCCCU
>Novel:hsa-miR-208a-5p
AGAAGGAGCACGAGGCCCCACUUUCACAGUCUGUAUUCAUACUGAAAAUCAAGAUUAAGUG
AGCUUUUGCCCUUCUGC
>Novel:hsa-miR-10524-5p
UCUGUAUCCGCAGUCUCACGUCCACGGAUUUGGUUGAACUGAGGACUGAAGAGGAUGCGGA
UGCAGAAC
>Novel:hsa-miR-6808-3p
UUGUGACCGGAAUUGAGGGCUCCAACCCUCAGCCCUGGUCCAGAC
>Novel:hsa-miR-3169
UCUGGGGUCAGGAUGGGAGAGAGAAUCAGGGUGAGGUGCUUUGGAAUAAAGGACUGAUCC
CAGAGG

>Novel:hsa-miR-4485-5p
GCCGCCUGCUCUGCUGCAGAACAGCUUCUGCCAAUGAACACCCAG
>Novel:hsa-miR-6780b-3p
AUAGCACUGCUUACAAGGAACAAGCCCUUGUAAGCCUUUUU
>Novel:hsa-miR-4795-5p
GUCCCGAGGCAGCCAGGAUCCCCGGUGAGCUGGGGCUCUGUGGGGAGUCACUUGAAGUGGU
GCCUGGGGACUC
>Novel:chi-miR-106b-3p
GAGAAGCCCCAUGUGGAGCACGGCUGAUGUCUGCAGGAGAGAGAGGAGACAGAGCAGUGAA
CUCCGCACUGGCUUCCUCCU
>Novel:chi-miR-3958-5p
GGCCUCCAUCGGAUAUGCUCUUUCCCCACGUCUGAGCUGAGGUCAC
>Novel:oar-miR-544-3p
AUAUGGGUCUGCAGAAUGCGACUCCCAUGCCUGCAGGUACCUCAAGCACUAGGAGUAGUUC
UGCAGGCCCCUUCU
>Novel:hsa-miR-4802-5p
AGAUCAAUGCCAAGGUCAGGUCAUGUGCCCAGGGCACAUGGAGGACUGGGUGCUGGGCAUC
>Novel:oar-miR-10b
UAGUAUGUACAAUGUUACCAUUUUUGCCCUGUACAAUGCUGAA
>Novel:hsa-miR-7113-3p
CAAUGGGUGGAGUGGACGGAGUAAGGUUCUAUUCCAACUCCCUGCUCCAAACAUCCAU
>Novel:hsa-miR-1321
UCAGCGCCCGUGGGCUCCAUGGGAGUCUCGACCCCCAGGGAGGGAGCCGCGCGGGGC
>Novel:hsa-miR-4659a-3p
AUUCUUCUUCUCUUUCCUUCUUACUCUCCUUUUCUUCUCGAGAACUAGGAGAAGAAGGUGA
UGCUGAAGAGGAUGAA
>Novel:hsa-miR-6830-3p
GAAUCUGGAGACCAAAUGCUGGUUCAAAACCUGUUCUGACCAUGGACAGGUUAUGCAGUCU
UUCUGGAUCUCCU
>Novel:hsa-miR-8077
AGUAAACAUCCACCUCCCAGCCGACUCGUGUGUUACAGCUGAGUGUAGGAUGUUUACA
>Novel:hsa-miR-545-3p
CCAUUUGCUUAUUUUGCUGAGAAUAAGUCAAAUGAACAUCAGCAAAACAGGCAAACGGAG
>Novel:hsa-miR-5195-5p
AAUGCUAACAUGUAGGAGUCAGUCCGAGGUAAAAACCCCUAUCACGAUUAGCAUUAA
>Novel:hsa-miR-4267
AGCUAGGGCGGGAUGGGCGGUCGUGAGCCUGGGGGCUCUACGCCCAGCUCACCAGAGCCCA
CCCUGA
>Novel:hsa-miR-4721
AGAGGGCUGAGGACCAGGGAAGCAGAGAGAGAGCAAGUCCCGCCUGUGAAUUUCCACUUGU
CUCUGCCCUCUGU
>Novel:hsa-miR-3126-5p
GCCCCAUGACCAUCCUUUAAAUCAAACCCAGAGGGACCUUGGUCAGGGGACA
>Novel:chi-miR-323b
AAGUAGUAAGUUGUAUAGUUAUCUUCAAAUUGGGGCCUAAAAUCCCUACCCCACAACAAUA
CAACUUACUACCUCA
>Novel:hsa-miR-1252-5p
CUGCCCAGAUGCGGUCCACUCUGAAGCCAAGAAGGAAUCUGGGCAGGC
>Novel:hsa-miR-148b-5p
CCCAUCAAACUUACGGAACUUGGAUGGAAACUUAACAAGUUCUGCAGUGGAUGGGCA
>Novel:hsa-miR-4668-3p
AAAAUUUGUUCUGGUUUUUCAUAAGCUAUUAUUGAAAAUCCCGAACAAACUUUCA
>Novel:hsa-miR-6886-3p
GGGGGCGGGCGGGCGGCCGUCUCGGCGGGAGCCGGCGGCCCUUCAGCGUGUCUUU
>Novel:chi-miR-485-5p

GGAAGUCACCCCAGCCUCCCGUAAAAUGAAGACAUUCAGCUUGCACCCAGAGAGGCUGUGA
GGUUCUGUCCU
>Novel:chi-miR-9-5p
CUUGUUUAGAAGCUAAGUAGGGGCAGUAAGUCAGUUAAGAUUUGGUUUACCUUUGGUUUG
GACUUAGGG
>Novel:hsa-miR-12117
GCUGACGUGCAGGGCGCUGAAGUUAAAGUGGACUGCGGCGAGCCG
>Novel:hsa-miR-4665-3p
CUCACUGUGGAGGCUGGCAAGCAGCUUCUGCUUUCGGCCGACAGCGGGGC
>Novel:hsa-miR-7160-3p
AAGGGCCCUGACUCAGAGCCCCACAGGGGGGCCCACAUGAGGAGGGGCUCACAC
>Novel:hsa-miR-6780a-3p
GUCCUCUGCCAGCCUCGGCACCUCGCGCUUUAUUUCUGGUGCCUAAGCAGGAGCGGAGGAC
AA
>Novel:hsa-miR-4689
AUGAGGAGGACGUAGGAGAGGGACAUCCUUAUGUACUCGUGUAUGUGUAGUGUCCUUAUU
ACUGUCUACAUCUUUAAAG
>Novel:NC_037554.1_25895
AGAAGAGGAGGAAGAGCUGAACAAUGUUCUUCACCUCUGUUCUCCCUUCCCCU
>Novel:hsa-miR-6839-3p
UCCGAGGACAAUAUAUUAAAUGGGUUUUUGGAGCAGGGAG
>Novel:hsa-miR-8070
UUGUGAUUAAAGGAUCAGAGCUCCAGCUCCCCUUCCAGGCUUGCCUCAAGGUGGAGAAGUU
UGGGGAUCUCAUCCUUAAAGCCACUGAG
>Novel:hsa-miR-6779-3p
GCUACCCCCAGCAGGGCUGUGAGGUGGGAGAGCCAGCCCUGAGUGGGGCCAGUGG
>Novel:hsa-miR-3529-3p
GAAGCUGUGACUUGUUGUCAUAGUACAUCAGAACAACAAAAUCACUAGUCUUCCA
>Novel:hsa-miR-4476
GAGGAAGGCAGGAGACAGGGUCAGAGCUAGACCGUAGCUCUUGGCUUCCCCUA
>Novel:hsa-miR-3918
UGGGGAAGGGUGGGGAUGAGGCCCACAGCCGCAGGCUCAGGGCCUUGUCGUCUCUCCCAGG
>Novel:hsa-miR-1252-3p
UAAAUGAGAUGUUCUGGCUUUACCAGAGCACGCUCAUUUACA
>Novel:hsa-miR-6892-3p
GCCCUCUCCAUCCGGCCCUCGACGCUCAGCUCUCUCGCCGGAGCUGGGGGGAGGGAG
>Novel:hsa-miR-4640-5p
UGGGCCAGUGGUGCUGUGCUUGUGGUUGACCGUGCCCCCACCACAGGUACACGCUGCUGC
CCAAG
>Novel:chi-miR-450-3p
CUUGGGAAUUAGGACUUGGGACUUGGGAUUCUCUAGAACAGGCUUUAAGUC
>Novel:hsa-miR-7162-3p
AGCUCACCAGGCCAAGGGCCUCUGUGAGCCCAGAGGACCUGAGGUGCUGGGAGCUCC
>Novel:hsa-miR-3150b-3p
AGAGGAGAGAAGAGCUGGGUGUCGGAUUCAGUCACUUCCCUUUGUGUGAAGUUUAGAGUA
ACAGCCUCUCCCUCG
>Novel:hsa-miR-4448
GGCUCCUUCUUCGCCCCUCGCUUUUUCUUCUCUGAACGGUAAGAGGAGGGGAGGGAGGACG
GGGUGGGAAAACG
>Novel:hsa-miR-4271
UUGGGGCCUUCCAGGAUGGUGGAGCCGUGACUGGCUUAAACCAAACAAGGGGAAGAGCCCA
GGGC
>Novel:hsa-miR-1304-3p
CCCGGUUGUCAAUGAGUCUGUUUUCACUCAGCUCACUGACAACCUCUCU
>Novel:hsa-miR-448
AGUUGGUCCUCCGUAUCCAUGGGUUCCUCAUCCUUGCAUAUGGAGGACUGUCUGU

>Novel:hsa-miR-4687-3p
AGGCUGUUCAACUCCUUCUUUAUUUGGGGUUGAAAAGCCCUGA
>Novel:NC_037568.1_45559
CCCGCCCGGCCCAGCCCGCCCCCUCAGGCCGUGGGCCGGGUCCGGGGUGG
>Novel:NC_037555.1_26459
AUAGGAGGAAGAACAAAGAAGACACAAAAGGAUGUGUCUUCUUUGUUCUUCCUCCUAU
>Novel:NC_037559.1_34348
CAGGUGGCGGCGCGCGGGCCCGGCGCGGCGCGGAGGGAGCCGGCGGCGGGCGCGCGCGGCCC
CGCGG
>Novel:hsa-miR-3179
AGAAGGGGCUCCUGAGCACUCUGGAGGAUGCGCGGAUGCAGACCAUGCUUGCUGUUGCGUU
UGGUGCAGUAUGUCUCUUCAAUUG
>Novel:NC_037561.1_36289
GUCCGGGGCUCCGCGCGCCUGGGCCCGCGGGGAGAGAAGGGGCGCGGGCGCGCUGAGCCCC
GGG
>Novel:NC_037569.1_47154
CCCGGGAGGGGGCGGGGCCGAGGCGGCAGAGGGCGGGGCCCGGGCGCGCUCGCCCCGCCCCC
CGGGCG
>Novel:NC_037546.1_4552
UAACCGGCCACCGGCCUCGCGGCGCCCUCCCGAGGGCGCCGCGGGGGCGGGGCCGGCGCGU
>Novel:NC_037555.1_27029
UGAUGCAGGGCAGGUUCCACAUGUAUGAAGGCUACCCGCUCUGGAAGGUA
>Novel:hsa-miR-3185
UUCCGGCUUUUCUCCUACAGCCGCCCAAGGACGACAAGAAGAAGAAAGAUGCCGGAAAG
>Novel:hsa-miR-1324
ACAGACAGUCCUCCAUAUGCAAGGAUGAGGAACCCAUGGAUACGGAGGACCAACUGUAC
>Novel:chi-miR-671-5p
AGGAAGCCAGUUGAAGAAGCAGCAUGCUCACAACUGCCAUCCUAG
>Novel:hsa-miR-185-3p
CUGGUGCCCAGCUACUCGGAGGCAGUGCUCAUGGACCUGGCGGGGCUGGGCGCGCGCU
>Novel:NC_037546.1_6618
GCCGGCCCCGCCCCCGCGGCGCCCUCGGGAGGGCGCCGCGAGGCCGGUGGCCGGUUA
>Novel:NC_037551.1_20226
CGCAGUAGGCUGGCUGCCUUGCUAGCGUGGAGGCGGCGAGGAGCCGAGCCCUGCGCA
>Novel:NC_037566.1_43625
GCCGGGCCCGCCGCCGGGCCCGGGAGUGCGGGGCCGGCGGGUCGGUGGGCCUCCC
>Novel:NC_037564.1_40975
GCCGCCGCCCGGGGCUGACUCAGGCCUAAUUGCUGCAUUUAUGAGUCAUCGCCGCGGCGGG
GC
>Novel:NC_037569.1_47143
AAGCCCCGCCCACCUGUGCGCCGCCCCGUGCGUGUCGUGCAGAGAGGCGGGGCGA
>Novel:hsa-miR-6852-5p
ACCUGGGGAGUGCAGGGCCUGGCCUGAGAACACUUGGAGCCGGCCCUGCAGUUCCCAGGGG
A
>Novel:hsa-miR-3944-5p
UGGCUCGGCCCCAGCCCCUCCCUGGGUGCAGCCCAGAGCCUCU
>Novel:NC_037561.1_36492
GAUGAGGCUCAGCGAGCCCUGGGAAUCAGACACAGGGACCAGGGCUCGCUGAGCCUCAUC
>Novel:NC_037556.1_30323
ACCCCAGCCCGGAGCCCACGGAGCACUCGGCAGUGGUGGCUGUGGCCGUCACCGCUGCCCCA
GAGGCUCCGGGCUGGUGGCUG
>Novel:hsa-miR-1908-3p
GGCGGGGAGGGGGCUUGCGGAUGCGGCCGCCCCCGGGGAG
>Novel:NC_037551.1_19470
UGCGCAGGGCUCGGCUCCUCGCCGCCUCCACGCUAGCAAGGCAGCCAGCCUACUGCGCUCC
>Novel:NC_037559.1_33762

CCGCGGGGCCGCGCGCGCCCGCCGCCGGCUCCCUCCGCGCCGCGCCGGGCCCGCGCGCCGCCA
CCUGACG
>Novel:NC_037561.1_37162
GAUGAGGCUCAGCGAGCCCUGGUCCCUGUGUCUGAUUCCCAGGGCUCGCUGAGCCUCAUC
>Novel:hsa-miR-6817-3p
UCUCUCUGUUCCACCGCUCGAGCCAGUACUGGAUACAGCAGGAGCAGAGAAACA
>Novel:NC_037546.1_4218
GGCUGCAGCCGCCGGCUUUCGGAGCAGCCGGCGGCCGCACACACUC
>Novel:NC_037546.1_5605
CUGCCCGGAUCCCAGUAGCCAGUAUCUCCCACCAUCCAGGCUACUGGGAUCCGGGCAGU
>Novel:hsa-miR-4446-3p
AAUUCUUGUCCCCAUGCCUCAGCUACAAGAGGGAUGAGGCAAGGGCUGCAGGGAGGGG
>Novel:NC_037562.1_38088
ACUGGACCACCAGCGAAUUCUCAGAUGUAUGAGAAUUCGCUGGUGGUCCAGU
>Novel:NC_037545.1_2589
GGGCGGGCGGGGCCGGGGCCUGGGGGCGGGGACCGGACUCGCCCGAGGGG
>Novel:NC_037545.1_1635
GCCCGCCUCCGCCCGGAGUUGACCAGAUGGAGGCUAGAGGGCAC
>Novel:hsa-miR-7110-3p
GCUCUCUCAGCGUCUCGGAGAAGAGAAGGGGGGGAAUCACACACUCAUCGUCAUGCUUGGA
GAAUACUGAAGGGAGAAG
>Novel:NC_037559.1_34015
GAGACCAGCAGGGUGAGGCCUGUGGCGGCCAGGACCUGCCUACCCCUGCCGUCCACCACCCU
CACACCUGCUGCCCGCUCAC
>Novel:NC_037566.1_43137
CCACCGACCCGCCGGCCCCGCACUCCCGGGCCCGGCGGCGGGCCCGGC
>Novel:hsa-miR-3124-3p
UGGGAAAUAUCUAGGAAACAGUCCACUUUCCUAGAUAUUUCCCACU
>Novel:hsa-miR-4700-3p
GACAGGACAGGGCAUGGGGGAUGCUCCAGAAAGAAGGGGCGUGCUCAGGGGGCCUGUCUGC
AU
>Novel:NC_037553.1_23622
UCAGGGAUGGGUGUCGGAGGAGGGUGGGGGCCACAGGCCCCAUCCUGAUGCCCCUCGCCA
CCUCCAUCCCUCAGAGU
>Novel:NC_037567.1_44553
CCAGUUAGGUGACUCAGAGUAACCAGUUAGUUACUGAUACUUACUCUGAGUGACCUAACUG
GUG
>Novel:NC_037547.1_9757
GGCCGUGACUGGAGACUGUUACUGAGGGCGGCCCGGGCAGUAAGCAGUCUAGAGCCAAGGU
>Novel:NC_037556.1_28345
AGGAUGCUCUGGCUGCCUGGCAGCCAGAGCAUCCGUC
>Novel:NC_037564.1_40897
GGCCCGGCCCCUGGCCAGGCAGGCCGAGCCUGCCAGAGGCGUCGGGCCGC
>Novel:hsa-miR-658
GUCUGGGGCGCCGGCGCCUCGCCCGCGCUCUGAGAGGCGGGCGCGGAGGGAGAACCCGGCCC
U
>Novel:NC_037566.1_43159
CGAGCAGGCGGCGCCCGGCCGAGAUCUGCCGGCCGGAGGCAGCGUGCUCGUU
>Novel:NC_037549.1_17067
CUCUCCCCGGGGCUCCCGGCGCAGGAGGCAAGCGCUUUCUUCCGUCCUGCCCCAGCCCCGGG
GGAGCG
>Novel:NC_037549.1_15945
UUCGGGUCCACAGAGCUCUCCGCUCGGUGCUGGGGCAUUGAGGAGGGGGCCCUGGGCCCGC
UCU
>Novel:NC_037545.1_698

CACCUGUGAGCUGAAACGCCCGCUCUCCGCACGCCUCCGCGACCGCCGGGGACCGAAGGCGG
GCGUCCUCCUCGCAGGCAGC
>Novel:NC_037548.1_12165
AGGAGGGGACUGCGGCCCUGGGGGACGGACCCUGGGUCGCGACCUCUCCCCGG
>Novel:chi-let-7i-3p
GGCGGCCGGCGCGGCCGCGGCCUGCGGGGCUGGCUGCGCAAAGCGUCGGGCCCCGG
>Novel:NC_037550.1_18158
GCCCCGCCCCCUCGCGGGGAGGCCACCUCUCCUGGUGGGGAGGCUUGGACU
>Novel:NC_037565.1_42675
GCCCCCCGUCCUCCUCCUGGUCCACCUGGCCUCUGGGGCUCAGGUGAGGAGCCAGGAGGCUG
GGCAGGGUGAUU
>Novel:NC_037549.1_15024
GAAGUCCCUGAACACUCUUAUAGAGUGGUUUUGUCCGAGUGUUCAGGGACUUCCC
>Novel:NC_037555.1_26499
UCUUUCCUCCUGUGGGGCAGGACUUCCCUGGCUCCACCCCAGGUCAGAAAGAGC
>Novel:NC_037549.1_16548
AGUCCCUGAACACUCGGACAAAACCACUCUAUAAGAGUGUUCAGGGACUUC
>Novel:NC_037561.1_36265
GGAGGGGGUGGGGUGAAGCACCCCGGGGCUCCCGCCGG
>Novel:NC_037553.1_23471
CCCGGCUUCCUGAGCGCGGCCGAGGAGGAGACACUGAGCCGCGAGCUGGAACCCGAG
>Novel:oar-miR-1197-5p
GCCUUCCUCCCAGGGCCCUGAGCCUGAGCUCCUGCCUCCAGGGUGCGGCAGCCAGUGGCCAU
CAGUGGGGGAAGGGGG
>Novel:NC_037568.1_45446
AGCCCGAGUCGCCCGGGUCUCUGGGGUGAAAAGAGUCUCCAGAGGGCCCUAAGGGACAGGG
CCCG
>Novel:hsa-miR-583
GAAAGAGGAUCCUUAGGAGAAGCAGUGUGAAGGAGAUGGAACUGAACUUGGGGGUGUUUG
UUGAG
>Novel:hsa-miR-3605-3p
ACUCCGUGGAAGCUGUCCUCUCAGAAGCUGGGCUUCUUCCUCGUGACUUU
>Novel:NC_037565.1_42436
CUGGUGUUGUGAAGUAGCCGUUCUCUGAUUGGCGACGGCCUGAUUCACAACACCAGCU
>Novel:hsa-miR-4683
GGGAGAUCCUCAGGAAAAGUCUCUCUGAGGACCCUGGGUAUCUCUGAU
>Novel:NC_037547.1_10895
CAGAGCCCAGGUCCCCGGCUCUGGGGCGCUUAGGACUCUCAGAGCCCAGGCCCCCGGCUCUG
GGGCGCUUAGGACUCUCAG
>Novel:hsa-miR-3180-3p
GCGCUGCUCGGGGCCGAAGAGGUGGCCGGGCCCGCGGCGACCGGCUCCUCAGGGGCGGCGG
CGGCGGGG
>Novel:NC_037561.1_36258
AGGCCUCAGACCGAGCGUGGACCCUCAGGCCUGAGGCCAAG
>Novel:NC_037549.1_14540
GAACCCCGCCAAUCCUCCGGUAGGUGUCCGGGAGACCGUGAGGCCAAUACUUGCGGGCAGG
CCGUGUGGGA
>Novel:NC_037547.1_10896
CUCUGGGGCGCUUAGGACUCUCAGAGCCCAGGUCCCCAACUCUGGGGCGCUUAGGACUCUC
AGAGCCCAGGUCCCCGGCUCU
>Novel:NC_037567.1_44107
CCAGUUAGGUCACUCAGAGUAAGUAUCAGUAACUAACUGGUUACUCUGAGUCACCUAACUG
G
>Novel:NC_037546.1_4225
GAGGGCGAGGGGCCUCAGAGGGCCUCCCACUUAUUCU
>Novel:hsa-miR-5586-3p

AGAAACUGGUUUCCCUCUUAUACAGACGGGAUAACUGAGGCCCCAAGAGGGGCCGCAGCAU
CCCAGAGAGUGAGAGAUGCCAGUCCCGAG
>Novel:NC_037566.1_43651
AACGAGCACGCUGCCUCCGGCCGGCAGAUCUCGGCCGGGCGCCGCCUGCUCGACCC
>Novel:NC_037569.1_48203
UGAGGGUCAAGGGUCAGAGAGCUGGUGCAGCUUGGGCUCCUGACCCGCCCCUCCUC
>Novel:NC_037560.1_35659
GCUGGAGGAAGGGCCCAGAGGCGAUCUGAGCACAACCCGCCUUCACAAAGGCCCUGCCUCC
AGCCC
>Novel:NC_037545.1_137
UGCUCCAACUCACAGCUCCCAGCAGGCGAAGCGCCCUGGGGGCGGGGGGCGGAGCGGG
>Novel:hsa-miR-572
GUCCGCUCGCAUCCGCCCUCCGGCAGCGCCGCGGGCGGCAGGAGUGGCGCGAUCG
>Novel:NC_037546.1_6567
UCCCGGGGGCCCAGGUUCUCCUUGCAUCCCUGGUUCUCCAGCUGAACCAGGUGGGCCUGGG
CUUCCUGGAAG
>Novel:NC_037553.1_23515
GGGGAGGGGAGGGGAGGGCCGGCUUUAGGAAGAGCCCGGCCAUCCUCCAGCCCCCA
>Novel:NC_037552.1_21136
UCAAUGGAUACGAAUCUGGGCUUCACUAUCUCCUGGAAUUGGCUCAGAUUUGUAUCCAGUG
AGU
>Novel:NC_037566.1_43276
GCUCCGAGUCCUCCGCUGCAGCUCACGGGUGAGAAGCAGCCAGGGGCUCGAUAAGA
>Novel:NC_037546.1_5469
AGGGUCAGAGUCAAGGGAGGGGAUGCAGUCUCUUGGCCCUGACCAAUG
>Novel:hsa-miR-1293
GAGGCUAUACUCAACACUGGGGCAGGGCUGUGAAUUGGCUUCCCUGCCUGGGUGGUAAUAG
AAUGGGCUCCA
>Novel:NC_037567.1_44729
UGGAUGCGUUCUUGCCACUGCUCUGUACUUGGAUCAUUUCACCUACAAGCCAGUGGUAAGA
ACGCAUCCAAA
>Novel:NC_037558.1_31787
GGGCUGGGCAGGUGCUGGGUAGUGAGCGCUAAACACACCUUCCCUGCUCUGC
>Novel:NC_037558.1_32253
UCCACCGCACCCGCCGAUGCCUUCUAUGGCAGGAGGAAGUGCGGCUGGACC
>Novel:NC_037568.1_44803
GAGCUGCUGGGCCCUGGGGAGGCAAAGGGAGGGCGGAGCCUCAGGGGCUGGACCCGGGGAA
GCCGGCGGGAGCCU
>Novel:NC_037565.1_41731
CCCAGGCUUCUGCUUCCGAGCGUUUGCAUACGCGGGCUCGGAGCCUGGAGCCUGGA
>Novel:NC_037556.1_29371
AGGGUGAGGAAGGCAGGAGGCCAGAGGCCAGGGCCCUUUCUUCAUGCCCC
>Novel:NC_037547.1_8540
UCGCCCUCUCAACCCAGCUUUUCCCGACUCCGGGAAGAACCGGGAAGAGAAGGCGGAG
>Novel:hsa-miR-7108-3p
GCCCGCCCGGCUCCCCGGGGUGCGCCCUCCUCGGCCCCGCGCCCUCGGGGCUCGCCGGCACA
GCCU
>Novel:hsa-miR-4783-5p
GGCGCGCCCCCGCCGCGCCGGGGCCCCUGCUGGCCUGGUGGCCGGGACGGCAGCGCCUG
>Novel:NC_037558.1_32412
CCAGAAAGUUCAUUCAGUUUUUCUGUACCAUCUUAUGGAAAAACCCAAUGAACUCUCU
>Novel:NC_037548.1_11146
AGUCCUAAGCCAAAAUCCUGGACCACCUAAUGGUCCAGGAUUUUGGCUUAGGACU
>Novel:NC_037548.1_12742
AGUCCUAAGCCAAAAUCCUGGACCAUUAGGUGGUCCAGGAUUUUGGCUUAGGACU
>Novel:NC_037546.1_6394

GGUGGCAGCCUCAGAGCUCAGGCCAAACCCCAGCCACUUCGGGCCUGGGACUGUGGGCGCCC
CCU
>Novel:hsa-miR-4515
AAUGUCAAAACGCUCUCUCCGAGCGGGAUCACUGGGAAAAUGAAGGUGGGACAGCGGCAAA
AGGACUGGAGAGCGCCCGCGGGUC
>Novel:NC_037568.1_45672
GCCAAGGCCGGGUCUGCAGGGUCCAGUCCACGCUGCAAAGCCAGGCCUUCCA
>Novel:NC_037561.1_36931
GCCCUUUCACCAUUGCACUGCUUGCCGGGGGCGCACAGUAGUGCAACAGGGAAAGAGUGU
>Novel:NC_037569.1_46587
AGGAGGGGAGGGAGGAACAGCUCUGAGGGCUUCUCCUCCUGCCCCUCCAGA
>Novel:NC_037550.1_18976
GUUGGCCAAAAAGUUCAUUCAGGGUUGGCCAUAUGAUCCGAACGAACUUUUUGGCCAA
>Novel:hsa-miR-4711-5p
GGCAUCAGUCCACUGGCACAGUUUACCUACAGGAAAGAUAACAGAAGUAAGCACUGCCCAC
AGAAAUGGGUGUCAG
>Novel:NC_037568.1_45368
ACUCGCCGUCGGAGGGGAGGCUCUGGUUACCAUUCAAGAUCUCCUGCUCGGCAGGAGAGUU
CCAGAACCUCCUCUCGACGUCGCAGAGG
>Novel:hsa-miR-6784-5p
CCCGGGGCUCAGCGCGCCCGCGCCCCUUCUCUCCCCGCGGGCCCAGGCGCGCGGAGCCCCGG
AC
>Novel:NC_037564.1_40367
CGGGGGAGCUGGGGCGUCUCCGGACGGGGUUUGAGGGAGAGUAAGCAGCCGCCUGUGAGGG
GCCUCGGCCUACUGGU
>Novel:NC_037548.1_12436
AGGUUCCAGGCGUUUGGCUGAGUGGCACAGGGUUACCAGACGGCCUCCCGCCUGGGUCCAG
A
>Novel:hsa-miR-6827-5p
CUCAAAGGCAAUGAAGGGGCUCCAAGAAAAUGCCUUUGAUGAAAGGCUAAGGGAGCCAGUU
UAUGCCGACAGAA
>Novel:NC_037552.1_21299
CGUAUCAGCACCCGCCUGUUAUCAAUGUUAUUUAAGAACGGACGAGUGUUGAUGCGAU
>Novel:NC_037547.1_7786
GUCGAGAGCUUGGUCCAGACCGUUUCUUUGAGGCAGGACACCAUGAAUGUGGUGGAGCGGU
CUGGCCCCUGCUCUUCCAU
>Novel:hsa-miR-744-5p
CCGGCGCCUUCCGCAGAGGCCAGGCAGGGCGGGGCGGCGGCGGGC
>Novel:hsa-miR-1203
UCCGGAGCCUAGGGCAAGAUUUUAGACUCUGAAUCGACUUGUUCUUUGGCCUCGGGUG
>Novel:hsa-miR-3960
GGCGGCGGUCGCUUCGGCUCGCCCAGUGGCCCGCGCCGAUGGCGGCCGCCUCCUU
>Novel:NC_037554.1_25410
CCAAAAGUUCACUCAAGUUUUUCCAUUACAUCGUAUGGAAAAACCUAAGCAAACUUUUGGG
C
>Novel:NC_037558.1_33125
GGUCCAGCCGCACUUCCUCCUGCCAUAGAAGGCAUCGGCGGGUGCGGUGGACUCA
>Novel:NC_037558.1_32071
GAGGGUGGGGAGAGGCACAUCAGGGUGGGAGUUCCCAGAUGCCCCCGCUACCUCCCACCCC
GUGUCCCCUCUCCCAGUCCCUCAC
>Novel:NC_037558.1_33041
GAGGGGAGAGCACCGAGGACCCCGGAACCCAGCUGCAGAAGCUCAGGGAGGGGGUAUGCUG
UCUCCCCUCCA
>Novel:hsa-miR-6781-5p
GCCCCGGCGUUGCAGGCCUCCGGCCGGCAGUUGCGCCUUUAAGCCGGGCCGGUAACGCCGGG
CACU

>Novel:NC_037567.1_44155
GGGGAGAGGAGCCGGUCGCGCGUACCCUGCAGGCUCUGAUACAUGCUCCAGUAGAUGCGCA
GACAGUUCUUCUCCUUCU
>Novel:NC_037562.1_39076
GCCUCUGGGACUACAGAUCCUUCUUCUCUCUGGUUCUCAGCCCCAGGGGUUC
>Novel:NC_037568.1_46017
GCUGGAGGAAGGGCCCAGAGGCGGCACCAAGGGGCUUGUGUGCGCACCCAGCCCAGCGGCA
GGCGCUUCUUCUAAAGC
>Novel:NC_037548.1_11079
AUUCUCCAGUCCUGCCUUCACCUGCCCAUGAAGGAAGUGGACGGAGGAGGC
>Novel:NC_037557.1_30447
CUCGGGACAAGGAACGCGUGCGGAAGCGCUCCAAGUCCCGGGAAAGU
>Novel:NC_037556.1_28346
AGGAUGCUCUGGCUGCCUGGCAGCCAGAGCAUCCGUC
>Novel:hsa-miR-6849-3p
CCCAGCCUCCACUCCGGCUCGCCUACCCGGGCUCCGCCCCCACCGCUUGAGGCGUCUCUCUC
CGGGGAGGCCCGGAGGGCACCGGACUCCGGC
>Novel:NC_037560.1_35933
CUUUCCGGCAUCUUUCUUCUUCUUGUCGUCCUUGGGCGGCUGUAGGAGAAAAGCCGGAAUG
CG
>Novel:hsa-miR-3181
CUCGGGCCUUAGAGCUCUUGGAAUGAGCUGGAGUGGGGCUUCCAAC
>Novel:NC_037552.1_21749
GGGGAAAGCAGAACGAGGCUUACUGGGGUGCAGACCCUCACCUGCUUUCCCCAG
>Novel:NC_037546.1_2774
ACGCAGCGGCCCGCUCUCUGAGGAGGCUGCUGGGUUUCGCUGCCUCAGCAAGCAGGCAGCA
CUGUGUCC
>Novel:NC_037561.1_36929
GCUUUGACAAUAUCAUUGCACUGCUCCUCAAAGCACAGUAGUGCAAUCUCGUCAGAGCGC
>Novel:NC_037564.1_40877
AAGCGGGGGUGGGGAAGCCAGGCUGGGCUUCUCCCCAUCCUCACUGAA
>Novel:chi-miR-491-5p
UGUGGGGAGGAUGGACCCUGGCUGAGCGUGAUGGGCCAGGCCUCCCGCCUCUCCAGGCU
>Novel:NC_037545.1_583
AAAUCCAGUGUGUAUUAAUAUUCUUUAUUAGAUGAUUAUUAAUAUUAAUACACAUUGGAU
UUCU
>Novel:NC_037546.1_3432
CUCGGGGACUGGGCCAGGCUUGUGCAAGGCCCGGGCCCCGCCCC
>Novel:hsa-miR-548u
UCCUAGCCAGAAUUUUCAAGGAGAGUUAAGAUAAAAAGACUGGCUAGAAAG
>Novel:hsa-miR-6771-5p
UUCGGGAGGCUAGGGACGGGCUGGGGUGCGGGGGCGCCGGCCGCACUUCUGUUCGUCGGUG
AUCAGAACC
>Novel:NC_037555.1_27170
AGGCAGAGCAGGGGCUUUUCCUUUGAGGGGAGGGAGAUGUUUACUCUCACUGAGGAGUUCG
GCUCCCCUGCCUGCCACU
>Novel:NC_037545.1_2765
UGCCAGAGGCCCAGGUCCCUCUUCGGGGAACACCUGAGCCACCGGCAGC
>Novel:hsa-miR-4467
CGGCGGCGGCGGCGACUCGCGGGCUCGGGGUUCGGGCUCCGUGGACUGCCCGGCGCGACGC
GGGA
>Novel:NC_037552.1_22028
ACUGGAUACAAAUCUGAGCCAAUUCCAGGAGAUAGUGAAGCCCAGAUUCGUAUCCAUUGA
>Novel:NC_037552.1_22617
CUUCCGAUGCGUCUCCUCUCCUGCAGGUUGUAGCUGCGGUGCCCGGGCUGCGCCUUGGUCA
GGAGGCGCCCGGAGGCA

>Novel:hsa-miR-6727-5p
CUCGGGGCAGCUCAGUACAGGACCCGUCAGGGAGGACGCAGGUCCAAGCUGGCCUCGAAGC
>Novel:NC_037563.1_39275
GUGCUUGACAGAACCAUGUUCCGUUUCCAUCGUUCCACCACAUGGUUAGAUCAAGCACAA
>Novel:NC_037549.1_15915
GUCAUCAUUACCAGGCAGUAUUAGAGACCAGACACCAUCCAAUGCUGCCCAGUAAGAUG
>Novel:NC_037568.1_44799
UUCACAGGUAGGAGCCCGAGGCCACAGGGGCCUCGGUCCUACAGCCGAAGG
>Novel:NC_037548.1_11583
UUCCCUGGCAGUCCAGUGGUUAAGACUCCACACUUCUAAUGCAGGGGGUGUGGGUUUGAUC
ACUGGUCAGGGAACUCAGAU
>Novel:hsa-miR-6085
UAGGGGCUGUCACAUAUGGGCCAUCAGAACCCUCAGGCCCAUUUAGUGAAGCCCCUGUU
>Novel:NC_037547.1_9583
AGAAGAAAGAGCGUGCUGGGGACUCCUGGCACCCUCUUGUUCCAA
>Novel:NC_037557.1_31142
AAAGUUUGUUUGGGUGUUUCUGUAAGAUGGUAUGAAAAACCUGAACGAACUCUUU
>Novel:hsa-miR-3144-5p
CCUUUCUCCCUCCCUCAGCCAAGAUCUGUCGGACCGCUGGGGACCCAGGGAGCCGGGGGG
>Novel:hsa-miR-943
UGGGCGGGCACCGGUGGCAGACAGGCCUGUGCUGGGCGCUUGACUGUCAGGAGCCCCUCAC
A
>Novel:hsa-miR-3908
GAGCAAUGAUGAAAAGGUUUGACUGCAGACCUUUUUAACUAUGAAAGU
>Novel:NC_037569.1_47983
AAAGAGUUCGUUCAGGUUUUUCUAUACAGCUUAUGGAGAAACCCAAACAAACUCUU
>Novel:NC_037562.1_38773
UGUCCGUGGUUCUACCCUGUGGUAGAACAGCAUGACGUAACCUACCAUAGGGUAAAACCAC
>Novel:hsa-miR-185-5p
UCCGGCCCGGUAUCCUGCCUGUCCACUGCCACCAAGGAGAGACCGGACGGAGC
>Novel:NC_037564.1_41171
GGCCCAGGAUGAGAACCCAGGUGUCCGAGUCACAUCCUUGCCUU
>Novel:NC_037545.1_1965
AGAAAUCCAAUGUGUAUUAAUAUUAAUAAUCAUCUAAUAAAGAAUAUUAAUACACACUGG
AUUUCUCU
>Novel:hsa-miR-10392-3p
GUCGGCGGCCGCGGCGGCGGCGCCCAGGGGCAACAGGGCGGCGGCCCCCGCCUGCUCGGCGG
>Novel:NC_037564.1_40336
AGAAGUGCACCAUGUUUGUUUCGUCACAUAUCAGCGAAAAGAACAUGGGCGACUUCUUU
>Novel:hsa-miR-6763-3p
GCGGGGCCCAGGUGGCCAGAGAAGGGUCCCCGGGUCUCCCGCCG
>Novel:NC_037552.1_21735
AAAAAGGAAGCAGGCAGCUGGGGAGGAGGCAGGGGAUGCGGGAGUUGGGGUUCUGCACAA
ACCCUGCGGCUUCAACCUCUUCCCAGGCUGUGCUGGCUGGAGCU
>Novel:NC_037545.1_315
UGGCUCAUUUAGAAGCAGCCAUAUGUCGUGUGGGUGGCUGCCUGUGAAUGGGCUG
>Novel:NC_037545.1_824
CCAGGAGCUCUCAAUCUAGUGGGAAAGUCAGAAAGGCAACUCAAUAGACUGUGAGCUCCUU
>Novel:NC_037546.1_2911
GACAGACGUAGACCUGGAUGUUCUGGCAAGCAAGCAAGCAAACCAGCUCUACCUCUGUCUU
>Novel:NC_037547.1_7477
AGCCUUGGACACAGGCUCCGUUCUUCUCACAUGAGCGGGGCUGUGGGCUGGCAGG
>Novel:NC_037560.1_34765
UUUUGGGGUAAGGGCUUCCGGCUAUUGAGUUAUGUACAGCAAGCCCAGACCGCAAAAAG
>Novel:NC_037557.1_30978
AAAACGUUCGUUUGGGUUUUUCGUACCGUCUCAUGAAAUACCCAGAUGAGCCUUUUGG

>Novel:NC_037553.1_24652
AGGCCUUGUGCUCCCUUCAGAAGUGAACAUACAGCCAGGCUUCUGAGUGGCCCACGGCUCC
U
>Novel:NC_037547.1_9951
GCUUUGACAAUACUAUUGCACUGCUAGCUGUACAGUACAGUAGUGCAAUAAAGUCAGAGCG
U
>Novel:NC_037551.1_19861
UCAAAUAUACAGCUUGUUCUUGUCAGCUAGAACUGACAAGAACAAGCUGUAUAUUUGAGC
>Novel:NC_037548.1_13083
AGAGGAGUAGGGCAUGGGACGUGACUGGUCCAACUGUGCUCCUGCCGACCUC
>Novel:hsa-miR-4479
UGCGCGGCUCUCUGCUCAGGAGAGCGCGGCCUCUUGCGCUUGUGCCCUCGCC
>Novel:NC_037556.1_29821
AGCCCCAGCCACAGCCGCAGAAUCGAGCACGCGAGCCCAGGAGCUGCAGGUUCCAUGGCUCU
CCAAGCGCUGCGGUCGGACUGGAGCGCC
>Novel:NC_037561.1_36340
GUCCCUCCUGCAGGUCCAGUUCCCACACAGAGCAGCAGGCACUCUGCUGGACUCACAGGGAC
UGUACCUGUGGAAGGUUCU
>Novel:NC_037555.1_28140
CAGGCAGGGGAGCCGAACUCCUCAGUGAGAGUAAACAUCUCCCUCCCCUCAAAGGAAAAGC
CCCUGCUCUGCC
>Novel:NC_037547.1_10096
GCCUUUCUCUCCUCCUUUCAUCUUGAGGGGAGAGGCUC
>Novel:hsa-miR-1184
AUCGAGCCCCUGGCUGCUUCUCACCCGUGAGCUGCAGCGGAGGACUCGGAGC
>Novel:NC_037547.1_10102
AGGAACAUGGAAAAUUGGCAGAAAUAGAUGAUGACUGACAAUCUUCCAUGUUCCUAU
>Novel:NC_037548.1_14012
AAGUCCGUUCGGGUUUUUCUAUAACAUCUUAAGGAAACCCUGAACGAACUAAAACCCAAAC
GAACUUUC
>Novel:NC_037565.1_42179
UGUCCCCUUUCAUUUCAUUCACCAGAGUAUUUGAAGGAAGCCUCAAGUGAACUUGAAGGAA
CCUGAGUGAACUCCCGGUGUUGGUGAUGGACAGGGAGGCC
>Novel:chi-miR-345-5p
UCUGACUCCUCCCUCCCUCCCACCUCCUAAAACAGCCCCUGGGAGCAGGCAGGGUGAGGCAG
CGGUGGGGGU
>Novel:NC_037552.1_21699
GCCGGCAGUGCGAGGCUGGAGGUCCGCGACCUCGGGACCCGCUCUGCCGGAGA
>Novel:NC_037547.1_7908
AUUUCUUAAGUUGAUGAGGACUAAAUUAGUCCUCAUCAACUUAAGAAAU
>Novel:NC_037547.1_10537
AAAGAGUUCAUUCAGGUUUUUCUGUAAGAUCUUACCAAAAAACCUGAAUGAACUCU
>Novel:NC_037569.1_47783
UUCAGUACUUGCAGUUCAUGGACUCAGUACUUGCAGCUCAUGGACUCAGUACUUGCAGCUC
GUGGACUCAGUACUUGCAGC
>Novel:hsa-miR-1249-5p
UGGAGGGACUGUGGCAGCGAGCCCGGCUCAGAAGUGCUAAGAGCUGCUACAGCCCCUUACG
>Novel:hsa-miR-570-3p
GGAAAGAUGUUGUCCACGUGACAGAUGAGGGUGUUGGGCUGACCCAGCAUCACAGGAGACU
UGGAAAACACAGUCACCUC
>Novel:NC_037549.1_15077
AAAUUUGUCCAGGUUUUUCUGGAAGAUGAUAUGGAAAACCCGAACGAACUUUUC
>Novel:NC_037547.1_7667
UGGGCGUGAGGCCGGCCAGGUAGGAGUUCUCCACAAAGCCUCGGCUCUCAGGCCCAUA
>Novel:NC_037558.1_32026
GGAAAGCCUCCGCCCAACCCUCUGAACCAAGCACAACAGGAUUGGGGGGGCCCUC

>Novel:NC_037547.1_7828
AUGGGGUAAGGAUGGAGAGGACCUUUGAGCGCAACUAGAUACCUCUCCAUUCUCAUCCCCA
GAG
>Novel:NC_037555.1_27093
AGGAAGACAGGUCUGAGGUUCAGCUCUGUCCCUGGAACCUCAGACCUUCUUCUCU
>Novel:NC_037545.1_1896
AGUUCCUCCCUCUCCGCGUUCGCCUACGGGAGAGUGAGGACUGU
>Novel:NC_037569.1_46554
UGAAUCCUUGAAUAGGUGUGUUGCACUAGAGCAGCACUCACACCUAGGUUCCAAGGAUU
>Novel:NC_037545.1_498
GUCCCAAGAGCCUGACUUGUGAUGUUACUAAAAUACCUCACAAGUUAGGGUCUCAGGGA
>Novel:hsa-miR-8057
UUGGCUCUGCGAGGUCGGCUCAAGGUGGGUCUGGAUGUUGAGCAGGCCUGUCAGGGCGUUG
>Novel:NC_037564.1_40376
CCUCCAGACCCCUAGUUCAGGGCCCACCAGCCAUCACAAGCACUGGACUAGGAGUCAGCAG
>Novel:NC_037569.1_46552
AGAAUCCUUGCCCAGGUGCAUUGCAUUCAGAAAGCACUCUCACCCAGGUAGCAAGGAUUA
>Novel:hsa-miR-4767
UGCGGGCGGACGAGGGGCCAGGCCGGCGCAGGGGCUGAGUUUCUGCAAAGUUUGACCUGGU
UGCCUUUCCGAUGCUGCAGC
>Novel:NC_037546.1_3187
CACAGUAGGUCUUGCCACAGAGCGAGCAACGGUAGGGCCUCUCCCUGAGUUGCUUGCGCCG
GCCAGAGCUGAGGUUCU
>Novel:NC_037550.1_19237
UGCAGCGCCCACUGUGGAAGAUGCCCUACAUGCUGCAGUGCCUGUCCCAGAGGGAACUGCG
AU
>Novel:hsa-miR-2355-5p
GUCCCCAGCCCGUCCACCCAGCCCCACAGCUCCUGGGGUGUCUUCCCUGCCCUGGGCUGAGA
CGCCAGGGGGCCA
>Novel:NC_037556.1_30288
CAUUAUUACUCACGGUACGAGUUUGAAGCGUCACAGCGCGUACCAAAAGUAAUAAUG
>Novel:NC_037552.1_21521
AGGAAUGAGAGACAGACGCGGCAGUGCUGGCGGCCUCAGUCUGUCCUCAUUCUUCU
>Novel:NC_037562.1_38928
UGGGAAGUGGGGUGGGCCAGGGAGGCUAGGCCCAGCAUGCACCCCCACUUCUCCUUU
>Novel:NC_037553.1_24219
GUCCUGGCUCCCCUCUUCCCUUCCCUGGAGUUCAUGAUUCAACCACAGGGGAGAAGGGUGG
CAGGCAGAGCCAGGAUAA
>Novel:NC_037546.1_4148
GGAAGAGGACAGUCCAUGGGCAGCUGCCAGAGUUCCUGCUGCCCUGGCAUGGCCUCUUUCC
C
>Novel:NC_037558.1_32366
AAAGACGCAGGAGGCCAAGAGCUGGCGAUUGGCUUUUGGCACCUCUGAGUCUCU
>Novel:hsa-miR-5580-5p
CGUUGUGGGAGAACCCUGAAGAGCGCCACGUCCUCCGGGCUGGCUCCCGCGGCCCC
>Novel:NC_037569.1_46557
AACCCUGCAUGUGGGAGGGGUUUUCAGAAAGCUCACCCUCCACCAUGCAAGGGAUG
>Novel:hsa-miR-2276-5p
UCCACAGCUCACAGGUGAUGGGCCAGGUACGGGAACGUGGACCUGGGAGCUCCCUCUGGGU
CUGUGGGGA
>Novel:NC_037547.1_9251
UCCAGCCCCUUCUCCUUCCUGGACCCUGAGCUCCUCUCUGUUCUCUGGGAAUGGAGCCAGGC
UGAGACC
>Novel:NC_037549.1_14559
GCCCCUGUGCCUUCCUGGUCCUUUGUAGACUUGCCUUAAUGAUAUACACAAAAGACUGGGA
GCAGGGGAAG

>Novel:NC_037566.1_43265
AAAGUUCAUUUGGGUGUUUCCAUAAGAUGUUAGGGAAAAGCCUGAAUGAACUCUUU
>Novel:NC_037564.1_40744
AAAGAGUUCGUUCAGGUUUUUCUGUAAGAUGUUACAGUAAAACCUGAAGGGAAAUUUUGG
>Novel:hsa-miR-6068
UCCAGGCCCCACCCGGUAGUUCAAGAACCUGCGAGGGGGCGGGGC
>Novel:NC_037555.1_28194
UUUGCUCAGGUUUUUCUGUACCAUCUUAUUGUACAGAAAAACCCGAACGAAUU
>Novel:NC_037549.1_14328
AGUUUUACUACACUCCUUCUCUGGAGGAGUGUAGUAAAACUAU
>Novel:NC_037547.1_6928
CCAAGGGAAGUCGGGCCUUUGCUGGAAUUAUCGGGAGCUCUCAGAGCAUCCCCGUCAGGAA
GGCCUGCUCUUCCCAGAGAA
>Novel:NC_037564.1_40741
UUCUUCCCAGAACUCCCACAUGUACUCCCUCUGGAGAAGAGUCCAGCCCUUU
>Novel:NC_037560.1_35724
AGGAGCCCUCUGGAGGGAGGCUUUCCCUCAGUUACAGUCUGUCCCUGGAGAGCAUCCUCU
>Novel:hsa-miR-6747-3p
GGAACAGGAGACGGAAGGACAGGGCCAGAGGCUGUCCUUCCUGCCUUCCUCACCCUC
>Novel:NC_037551.1_20840
GAGGGGUGGACUGAGAGCCAGGAUUCCAAGGCUGGAUGACUCUCAGUCCCUUCUCCU
>Novel:NC_037564.1_40932
GUCCUCCUACUCCUCUGCUAUCUUGUUAGGGAGUCAAGACAGCUGAGAAGUAGGACAAUGU
>Novel:NC_037553.1_23542
AGGAGCCGUGGGCCACUCAGAAGCCUGGCUGUAUGUUCACUUCUGAAGGGAGCACAAGGCC
UCUCC
>Novel:NC_037547.1_9916
AUUUCUUAAGUUGAUGAGGACUAAUUUAGUCCUCAUCAACUUAAGAAAU
>Novel:NC_037568.1_45118
AUGGCAACUGUAACGGCGCUGCGCCGCACAGUGCCCCCC
>Novel:NC_037569.1_46660
AGAAGCAGCUGCAGAAGGACAAGCAGGUCUACCGGACCAUGCACCACCAUCUGCUGCUGCU
GCUGA
>Novel:NC_037560.1_35631
AAAGAGAGGCUUUGAACCUGGGACCCACACCCAGGAGCAAAGUUUCGCCCUUUUG
>Novel:NC_037564.1_40236
GUGGUCCUAAACAUUUCACAAUUGCGCUACAGAACUGUUGAACUGUUAAGAACCACUG
>Novel:NC_037547.1_9353
GCUCCUGGAGGACAGGGACUUUAUAUUCAUUGUUUGUGACACCAGUUGCCC
>Novel:NC_037553.1_24557
AGCAUAGGUGGGGCUGGGACUUCUGAAAUGUAAUCUGACGAGGCUCUAGUUUUUCCACCUG
GCCAG
>Novel:hsa-miR-6513-5p
UUUGGGAUAAGGGAAGGACCAAGGGCAUGGCAGCUGGUGUGACUUUCCUGAGGCUAACCUA
UUGGUCCUCCUUCCCGAACC
>Novel:NC_037565.1_42130
AGGAGAAAGCUGUGCACUCCUGGUCACGGAUCUCCACGGCGGGGAAGAAGGCCUUGGAGGA
AGCGCUGCUUGUCUUCA
>Novel:NC_037546.1_4816
AUCUGCAGUUUGACGGGCUUAACGUUUAACAGGAUGUGGGCCUGUUUGCUGCAGGUAU
>Novel:NC_037545.1_1554
UGUCUUCUUGGCAUCUCUGCAAGGCUCCACAAAGCAGGUGGUGCAGGACGGAGGAGGU
>Novel:hsa-miR-4537
CGGCCGCCAUCGGCGCGGGCCACUGGGCGAGCCGAAGCGACCGCCGCC
>Novel:NC_037547.1_7372
AAGAAGUUCGUUUGGGUUUUUCCAUAAGAUAUUAUGGAAAAUGAACUUUUUGGCCAAC

>Novel:hsa-miR-6846-5p
UGGGGGCUGGAGGAUGGCCGGGCUCUUCCUAAAGCCGGCCCUCCCCUCCCCUCCCCCAGG
>Novel:NC_037546.1_4402
ACUUUUGUGACUAUGCAACUGGAGUGAGAAUACUAGCUGCAGAUCACAAAAAUGA
>Novel:NC_037550.1_17666
CCAAAAAGUUCAUUCCAGUUUUUCAGUAAGCUGUUAAAGAAAAACCUGAAUGAACUUUCUG
G
>Novel:NC_037550.1_17420
AUUGUUGUCGAGGCGGCCGGCUUGUGCGUCGUCUUAGCAGCAAACA
>Novel:hsa-miR-4450
GGGGGAUUAGCUCAGCUGGGCUGCUAAUUGCCUCCGA
>Novel:NC_037569.1_47073
AAGUGCUUGCAUUGCAGUAGAUCUCAAAAAGCUACCUGCACUGUAAGCACUUU
>Novel:NC_037551.1_20645
AUCUAGGAGAUCCGGCUGAGGUGGAGAUCCGGCUGAGGUGGAAUCCUGGGGGC
>Novel:NC_037552.1_22370
UUUUGGGGUAAGGGCUUCCUGACUACUGUAGAGAGGAACAGCAAGCCCAGACCGCAAAAAG
>Novel:NC_037564.1_41393
UGGCCAAGUGGACUCUUCUCCUGUGUCUCCUCCCAGGAGGCGGCUUCCUUGGUCACC
>Novel:NC_037546.1_3818
AGUUCUGACUCCAAGUCCAGGGAACUUUCUGGAGUUGGACACUGA
>Novel:NC_037553.1_23265
CCUGGCACAUAGCAGGCACUCAACAAGUAUAUACUGAGUACUUACUAGGUGCCAAG
>Novel:NC_037560.1_35884
CCUAUAGACACAAGCUUGUGUGCGCUACCACCACAAGUUCGGAUCUACGGGUU
>Novel:hsa-miR-663a
CCGAACUCUGCGCCCGAGUUCCCCGGACGUUUCGAUUGUGUGGGAAUCGUCUGGAGAGGCG
GGGGACGGAGGAGGC
>Novel:hsa-miR-4800-5p
CCCUGAGCUCAACGGGUAAGACCCUUGGCUGUUUUCUGCCAUGUGGACCCAGGGAGCCGGG
AGG
>Novel:hsa-miR-5739
GGGCCCAGGGCCCCCUCCUCAAUGCCCCAGCACCGAGCGGAGAGCUCUGUGGACCCGA
>Novel:NC_037569.1_48052
UUCAGUAAGUUUUUCAGAUGAUUAGUUUGUGUAAGAAUCAUCUGAAAAACUUAUUGAAAC
>Novel:NC_037547.1_9101
GCAGCCAAGCCUCCUAACAGUUGGGUUUCAGUUUCCAUUCCUGUUAGGAGAGGCUUGGCUG
GGU
>Novel:NC_037564.1_40695
CCAGGGCUGUCCCUGUCCCACAGGGACAGGGUCGGGGGAC
>Novel:hsa-miR-4428
AAAGGAGAGGAGGAAGAGGCUGUGCUGUUUGCCAAUCUUUCGUUUGCCUCAGACAGGUUUU
UCUUCAUUAUCA
>Novel:NC_037569.1_46549
UCCAUGCACAUAGGAGGUGCUUUGAGAAUUCACAGCUCCGAUAUGCAAUGGGUA
>Novel:NC_037546.1_3986
AGGACACAAGUUGGGAAAGCAGCCUGGUGUCUUAGCGGGAAUUCUUUUCUCUUGUGGCCUU
A
>Novel:NC_037547.1_9992
UCCAUAAAGUAGGAAACACUACACCCUCCAGUGCUGUUAGUAGUGCUUUCUACUUUAUGGG
>Novel:NC_037552.1_21762
CUCUCCACAGCCUCCUCUUUAGAGCUGGAAGGGGCUGUGGGGACUA
>Novel:NC_037558.1_32847
GCUGGGCCCAGAGUCCAUGUUCCUGGACUGAGCAGCUUCCACCAGCACUAUGGGUCCUGGA
CCCAGCCC
>Novel:NC_037564.1_40946

AGAGCCACUCCAACAUCUGUACAUGGCCCACCCUCAGAUGGGAGCCUCGCAAAUGGUGGUG
ACCUGGCUCUUC
>Novel:NC_037558.1_31842
AGGCCAGGGCCUUCUCCUCCCAGUUGGGCCCUGCUGAGCCCUA
>Novel:NC_037564.1_40778
GAAAGUUCGUUUGGGUUUUUCCGUAACAGCUUAUGGAAAAACUUGAUCAAACUUUUCG
>Novel:NC_037564.1_40307
GUGGACCAGGUGAAGUACAUUAGAAACAAUUACUUUCCAACUGGUCGACCA
>Novel:NC_037547.1_10878
CGGGAAGAAGGUGACCUUGGUAUCCUUACAGGUGAACCUCCUGGGAGGUUCCUUUUUUCCU
GAG
>Novel:NC_037561.1_37123
AAACAGCCCGAGUCUCUCGAUUCUCAAAUUUGUUAGAAUCACAGGGAAAUGGGGCUGGGAC
U
>Novel:NC_037553.1_24410
CCCGGAACACGUGUCUAAAGCAUUUGGUGCGUUGGCCGGGAA
>Novel:NC_037552.1_22436
AGUGCCUCUUUGAGGAAGGAGCUCUGUCUAGAGUUUCCUCUCCCGGUCCAAA
>Novel:NC_037560.1_35256
GAUGGCAGUGGAACUAGUGAUUGUAAGCAUGAGAGUACAAUCAGCUAAUUACACUGCCUAC
AA
>Novel:hsa-miR-6515-3p
AAGAAAGAGACUGGAGAAGUUUGCCCCAUGGUCCCUCAGUGUAGCCAGCGAUAUCUCUUCA
GCUCUUCCCU
>Novel:NC_037569.1_47107
GGAGAGUAAAAAAUGAUUUGCUAGAUGUGUUAAAGAACUAGUCAAAUCAAUUUUUUACCU
CU
>Novel:NC_037552.1_21636
CUGGUCCUCAGCUUCUCUCAUGAACUUGAACGACCUUUCCAGCUGCAGGGAGAAGGGUGAG
AGGAGGCU
>Novel:NC_037548.1_11963
UUUCAGUUGGGGAGGACAGAGGCUCACUUGGUCUUGAAGUCCUCCACCAGCCCCUGCA
>Novel:NC_037560.1_34839
CCUGAUGGCUCAGUGGUAGAAGAAUCCACUUGCAAUAUAGGAGUUGCAGGAGAUGUGGGU
UCAAUCGCUGGGUUGAGAGG
>Novel:NC_037564.1_40358
AGACUCAUUUGAGACGAUGAUGGAGCGUAAGAAUCCAUCAUCAAAACAAAUGGAGU
>Novel:NC_037547.1_8172
CUUCUGUGUUCUCUUCCUCAGAGGCAGAUCAGCAAGUC
>Novel:NC_037551.1_19485
GGAGGUGGAGAAGUGAAUCAGCGUAGCAUCGAUUUCCAGCUCUCGCUCUCUGCCUCUCU
>Novel:hsa-miR-4723-5p
UGCUCAGGGCGGCCCUGCCUGCGGCCUGGAAGGGGGAGGACGGAGGAGG
>Novel:NC_037562.1_38494
UUUCCCGGAAUGCGUGGCUAUAACAUUUGGUGCGUUGGCCGGGAAACG
>Novel:NC_037564.1_40345
AGUGGACCGUGUUACAUACGUCACAGAUAAAGCGUACGCUAUACGGUCUACUAU
>Novel:NC_037557.1_30428
AAAGAGUUCGUUCAGGUUUUUCAUACCAUCUUACAGAAACACCCAAACAAACUUUUUG
>Novel:NC_037548.1_12074
GCCACUAGACCACCAGGGAAGUCCCUAGAGUCUGUGUGUUCU
>Novel:hsa-miR-6778-3p
AGCCUCCCAUCCAGCGGCUGGGAGAUGCUGACCGGACCCACCGGCAGAGGAGAGGCUGCUG
GAGCGACAGGAUCA
>Novel:NC_037547.1_10839
AGCACCCACAGCUUCUCCUUGAGUUUCUUGGUGGACGAGCUGAAGGUGCUGC

>Novel:NC_037557.1_31269
CAAAAAAUUUCAUUUGGGCUUUUCAUAAGAUGUUGUAGAAAAACCCAAAUGAAAUUUUUU
GGC
>Novel:hsa-miR-6784-3p
CCUCACCCCGCCCGCCCGCCUCGGUCUGGCUAGGUCCGGGGAG
>Novel:NC_037563.1_40142
ACUUCGUUGGGGUUUUUCCAUUCCAUCUUAGGGAAGAACCUGAACGAACUUU
>Novel:hsa-miR-6888-5p
CGCCCCAGGAGUGACGCCUCCCGGGAAGAGCUGGCUGAACUGGCCCAGCAGGCCAACCCGGA
GGAGAUUGAGCUGGGCGAG
>Novel:NC_037548.1_13020
GGCCAAAAAGUUUGUUUGCAUUUACCCAUAAGAUAUUACGGAAAGAUCCGAACGAACUUUU
UGGCCAA
>Novel:NC_037552.1_21563
AGUUUCCUUUCUCCUCCACAGGAAGCUAAUGGCAGCUUUGGACCGGGAGAGGAAACUCU
>Novel:hsa-miR-4733-5p
GGCGGGGAGCCCAAGCGGCAGCCUUUUGACCACAGAUCCCAAGGCCGAGUUCUCCUCCU
>Novel:NC_037564.1_41452
AAUUUCCCUUCAGGUUUUACUGUAACAUCUUACAGAAAAACCUGAACGAACUCUUU
>Novel:NC_037553.1_24374
GCCUGGAGAAUUCCAUAGAGUAUAUAGUCCAUGGGGUCACAAAGAGUCA
>Novel:NC_037546.1_6204
AACUUCCAGAUCUUUGACUUUGAACUGAACAAGGAGGAUAUGACCACCUUGCUCAGCUACA
ACAGGGACUGGAGGGCCU
>Novel:NC_037569.1_47131
GUAGUCACUAGGGUACCAUUUUGAAACAAUGUACAACCAUCUACUAAACGGAACCACUAGU
GACUUG
>Novel:NC_037545.1_1001
AAGCAGCACAAUAAUAUUGGUGUUUAAUAUUUAUUUUACUACGCCAAUAUUUACGUGCUGC
UA
>Novel:NC_037551.1_20679
AGGAAAGGGCUGGGGAGUCUUUGUCCUGAGCCUGCCUCA
>Novel:NC_037560.1_35881
AAAAGUUGGUUUGAGUUUUUCCAGAAGAUGGUAUAGAAAAUCACAAACUUUUUG
>Novel:NC_037565.1_41728
UUUUCUCAGUUACUGUGAACUGGAGGCUAGAGUUCGUUCACAUUAACUGAGAAACGGCCU
>Novel:NC_037566.1_42927
AAGUUUGCUCAGGUGCUUCCGUGCCAGCUUGCAGAUAAACCCAAACGAACUUUU
>Novel:NC_037569.1_47135
AACCAUGGGGUAUGAGCAGACAAGCAUGCUGCAUGCUCAGACAACCAUGGUGCA
>Novel:oar-miR-299-3p
CAUGUGGGCACUGAACGCUCUGGAAUCGAGGAGGGUGGAAUUUGCUCCUGUG
>Novel:NC_037564.1_40326
ACAGAGAGCUUGCCCUUGUAUAUUCUACAUCAAAAAACCGAAUAUACAAAGGGCAACCUCG
CUCUAA
>Novel:NC_037546.1_4997
AAGACAGAGGUAGAGCUGGUUUGCUUGCUUGCUUGCCAGAACAUCCAGGUCUACGUCUGUC
UUCU
>Novel:NC_037557.1_30641
AAGUGCUCAUAAUGCAGUAGAUAACUAAACACUACCUGCACUAUAAGCACUUUA
>Novel:NC_037553.1_22994
GAGCUACAGUGCUUCAUCUCAGACUCCCAACUGACCAGAGAUGCAGCACUGCACC
>Novel:hsa-miR-149-5p
GGCGGGGGGGGGGUGGUAUGUGGAAAUCUGUGCUGGCUCCCUCGGCCCA
>Novel:hsa-miR-146b-3p
UCCCUGUGGUCUAGUGUUUAAGAAUCCAUCUGUCAACACAGGGGAU

>Novel:NC_037545.1_431
AAAAAGUUUGUUUGAGUUCCUAUGGAAAAACCCAAACGAACUUUUC
>Novel:NC_037566.1_42967
GCAUGCUGGACCUUUUGAUGUUUGCUGUGGCCUGAGGGAUCUUUAGUUGCUG
>Novel:hsa-miR-3917
GUCCACUCCCUUCCCUGUGUGGGCGGUAUGCGCACGUUGAAGCCACCUCGGACUGGAGGAC
UGUGGCCG
>Novel:NC_037564.1_41155
GGGCAUCACAGACCUGUUCUUGCAGCGGGCUGACUUGUCUGGCAUCAUGUAAGAGCUAAAC
CUGCGGGUGUCCAA
>Novel:NC_037565.1_42630
GUCCUGGGACCUGGGCCUGGAUGACUUCUGAUUAUCUAGAGAUCAAUGUCCCUCAGAGGCC
CAGGCCUCAGUCCUC
>Novel:hsa-miR-5587-3p
CCGGGGAGCUCACAGAGGGGACCCUGUGCCCCGGGGGCACCCGGCU
>Novel:NC_037569.1_46468
GAGACCCAGUAGCCAGAUGUAGCUACUGAUUACAAAAGACAGGAUCUACACUGGCUACUGA
GCCAUUG
>Novel:hsa-miR-1301-3p
CCCCACCUGCCCCAUCACCCCCAGCCUGGCAGGCUCGGGCUCUGGGCACUGCUCGGUUGCAG
CUGGUGGAGUUCUCGGC
>Novel:NC_037558.1_32842
CUUCUCCAGGGGAUCUUCCCAGCUCAGGAAUUGAAUCCAGGUCUCCAACAUUGCAGGCAGA
UGGUUUACCGACUGAGCUAUGCGGGAAUCCCCCAUGGUCUAGUA
>Novel:NC_037546.1_6872
GGGUGGCUCUGGGCCCGGACGCCACCUCUACAGACCCUCCCAGAACCCCAGGGGCGGCUCUA
GGCCCAGAUUCCACCUCU
>Novel:NC_037555.1_26737
CCUCCCCGCCCCCUCCGCUGGCCCCACUUCCUCGGCGCAGGCGGCGGGAGCGGGAC
>Novel:NC_037556.1_28514
AGGUUGGAGGUGUGGGCUAGAGUAAUGUUUUUUUGUCAGCUGGCCCCUCCCUCCACCACC
>Novel:NC_037555.1_27452
ACUUGGGUAUCAAUCCCAAAGUUUUUAAUUAGAGAAGAGUUCUUUGGAAUUGGUACUGGG
ACG
>Novel:hsa-miR-1915-3p
UGGGAAACUCGCCUGGAGGAAGACCCCAGGGCCUUUCCUUCU
>Novel:NC_037561.1_36813
AGCACGGGGAGGAGGUCAAAUUACUUUUGCCCUUCUGCUCC
>Novel:NC_037546.1_3761
GGAGAAAGGGAAGGUCUGUCACCUGUGAACAGGAUCUCUGAGAACUAGUUUCUUUUCCUCC
G
>Novel:NC_037554.1_24998
ACCCAUGGACUGAGAGCCAGACGGGCUACAGUCCUUGGGU
>Novel:NC_037545.1_1689
CAGCCCAUUCACAGGCAGCCACCCACACGACAUAUGGCUGCUUCUAAAUGAGCCA
>Novel:NC_037565.1_42629
AGGCCCAGGCCUCAGUCCUCCAAAAGACUCCUGCCCCUGUAUGACUGGGCUGGGCCCAU
>Novel:NC_037545.1_558
CAGAGUACUAACCACUAAAAGAUCAUGGCCAUGGUAUGUUAGAUUUAAAAAGUGGGUGGA
UUCAC
>Novel:NC_037547.1_9455
AGAGGAAGAGAGAAGAGCAGGAGUCUAGAGUCAGACUGAGUUUAGAUUCUGACUCUACCA
UUUCCUGUAU
>Novel:hsa-miR-6801-5p
AGGUCAGAGUGUGGAACCGGGAGUUCGCUUUACAGUCAACCAGU
>Novel:NC_037553.1_22953

CAACUGUUGAGGCUUCUAUGAUUCAGGGAGAGCAAAAUAUGAUUCCUGCCCUCUAGGAGCU
CACAGUCUGGU
>Novel:hsa-miR-6089
AGAGGCCGACCGUGUAUUGUGCGUCAUGAAGAAUGCGAACGGGCCACGGGCAGCCUCUCC
>Novel:NC_037554.1_25983
ACAGGACUUGGAGUCAGACAGUCCUGGCACUGAGUCAUGUCU
>Novel:NC_037545.1_1362
UUCUCCCGGGAGCUCGGCCUGCGAGGUGUCGGUCGGCCGGCCGCCGCGGGAGA
>Novel:NC_037548.1_11601
UAGAGAGAAACAGUGUGAUGAGCUUUUACUAUCUCAUCACACUGUUUCUCUCUA
>Novel:NC_037558.1_33240
CCCGCCCCUUCGCUCUCCUGAUGGAGGACCCUCUAGCUGGAGAGCUGGAGGGGCGGCCA
>Novel:hsa-miR-4706
UGCGGGGAGUGCACGGCCUGCCAGGUAAAGGAAGACUGUGGGGCCUGCUCCACCUGCCUC
>Novel:NC_037557.1_31699
CCACUACACUAAGGAAACCACAACUCUGUGGUUUCCCCAGUUUAGUGGUU
>Novel:hsa-miR-635
CCUUGGGCUGGCCUCUGACCUCGGUGGAAUUGUGGGGACAGGGGAAGGAGGGAGAUGGUGA
GGGGUG
>Novel:NC_037561.1_36598
AAGGACCUGGAGAAAGCCUAGCUUGAUCCAGGCACUGGA
>Novel:NC_037568.1_44853
CCCAGGCCUGUCGGUGGCCUCCCCUUGCGACCGGACAGGUCAGGGC
>Novel:NC_037569.1_47030
GUUUCAAUAAGUUUUUCAGAUGAUUCUUACACAAACUAAUCAUCUGAAAAACUUACUGAAA
UUG
>Novel:NC_037548.1_13192
UAGAGAGAAACAGUGUGAUGAGAUAGUAAAAGCUCAUCACACUGUUUCUCUCUA
>Novel:NC_037550.1_19148
GGCCCCAGGUGGGCCUGUCCAUGGGUUGGGCACAGCAGUUUCCUGAGUAAGAGCCUGCCCC
ACCCUCAGGGCAGCACCCCAGUCCA
>Novel:NC_037554.1_25287
AAGUUGCUUUGAGUUUUUCUGUAAUAUCUUACGUGAAAACCCAAACGAACUUUU
>Novel:hsa-miR-3125
GAGAGGAAGCGGGAGAGGAGCCCACGUCUCCUGUCACCCAAGUCCUCCAGCUGCG
>Novel:NC_037558.1_32697
CUUGCCUGUGGUCUCAUCACCUGCCCUCAGCAGAGUGAGAAACAAAAGGCAGGAG
>Novel:hsa-miR-1199-5p
AGGCAGAGCUUUGAGCCAGACCCCUGGAGGAGCACAGCUUCCCUACCUCUGAGCCCAGGGC
CCUCCCCCU
>Novel:hsa-miR-4277
ACAGUUCUCCAACUGGGAGCUGAGGAAGAGAGGUGGGAGCCCUGAGUUCUUGGCUGAACCA
GUCUGGAGUAGAGUCU
>Novel:NC_037558.1_33211
CAAAAAAUUUCAUUUGGGCUUUUCAUAAGAUGUUAUGGAAAAACCGUAAUGAACUUUUUG
GCCAACCC
>Novel:hsa-miR-6124
AGGAAAAGAUUUCGGACUCUGAGGUGAGUGUUAACCUGAGCUACGCGUCGGUUUUCCUAA
>Novel:hsa-miR-5589-5p
GCUGGCUCAGUCUUUCCAUGUUGUCUUGUUACCUGAGAGCUGGGUCUGAGGCCCCU
>Novel:NC_037546.1_2906
UCCUCCAGAGGUUUCCUUCUUUCAAUUAAAAUGACAAGACACCAAAGAAGUGGAACCCCUG
GAGGAAUG
>Novel:NC_037547.1_8774
UUCUGAACCUGCGGUUCCUGCCCUUUGUGGACCCUCAAAUUCUGGGCUCACAUAAGGGCCC
UGGAACACUGGUGGGAGGAAAA

>Novel:NC_037558.1_32244
CCAAAAGUUCACUCAAGUUUUUCCAUAAGAUGUUACGGAAAAACCCAAAUGAACAAUUUGG
C
>Novel:hsa-miR-1205
GGCCUCUGUUUGAAACCAACAGCUAUAGUGUAACUGCAGGUCUUCAGUCAGGGCCAU
>Novel:hsa-miR-3683
CGCGACAUGAUCAUUCUGCCCGAGAUGGUGGGCAGCAUGGUUGGCGUCUACAA
>Novel:NC_037549.1_14335
GGACGGAGUCCCGGGAAGCAGGGAGGGGGUCCACUCCAGUCCCGGGGCCCCUCCUC
>Novel:chi-miR-103-3p
AGCAGCAUUGUCCAGGGCAGCCAGGGCUUGGAAGUGUUGAGGA
>Novel:chi-miR-493-3p
GGAAGGUCACAGCCCUGGGGAGGCAGUGAUUUCUCUCCCUGUUUGGAUACUCUUG
>Novel:NC_037561.1_37534
AGGAGUUGGAAUAGGAGCUUGCUCUGUCCACUCCAUG
>Novel:chi-miR-449b-3p
GCAUCCGGCGAUCGUUGCUGCAGUUUUUACCCCUCAUUCCAUACAAAGGCAGCAACGCCAC
AAUUGCAGGACACUG
>Novel:NC_037546.1_4856
AGAAAGACUUGGACAGCUUCCGGCUGUCCAAGUAGCUUG
>Novel:hsa-miR-4761-5p
GACAGGUCGGCCGAGGAUGACUCAUCGAGUGACGAAGGGUCCCAGGAACUGGAAGAGUCCA
UCAAGGUGGACUCUGCCC
>Novel:NC_037550.1_19123
AAAAGUUCAUUCCAGUUUUCCCAUUUAUGGAAAAACCCAAAUGGACUUUUUG
>Novel:hsa-miR-339-5p
UCCCUGUCCUCCAGGAGCCCCCGGGCAUAUGGGAGACAGGAUAG
>Novel:NC_037547.1_7586
AGAAAGGAAGGAAUCUGGGCCACCCAGAUUUUUUCCACAGCUUG
>Novel:NC_037563.1_39389
AGAUUUUGAAGAAUUGCGUUGAUCUACAAAUUCAAAGCAAUCUUUCAAAAUCCUA
>Novel:NC_037569.1_46466
AAACCCAGCAGACAAUGUAGCUGUUGCUCAACAAACACAGAAAUCUACAUUGUAUGCCAGG
UUCA
>Novel:hsa-miR-219a-2-3p
GGAAUUGUACAGUCCAUGGGGUUGGACAUGGCUGAGCGACUUUCCCUUUCCCUAUGGACUG
AAACAGAGAAUG
>Novel:hsa-miR-4475
CAACUGGACUGUCCUCUACAGAAGAAAACACAGAAAGGGACAGUCGGAAGAA
>Novel:NC_037549.1_16477
GCAGAAGGGCAAAAGCUCACUUAAUCUUGAUUUUCAGUAUGAAUACAGACUGUGAAAGUG
GGGCCUCGUGCUCCUUCUGACC
>Novel:hsa-miR-4276
UUCAGUGAGGAUGGGGAGAAGCCCAGCCUGGCUUCCCCACCCCCGCUUUCUG
>Novel:NC_037569.1_46653
AAUAAAUGUUUGUUGAUGAGCCAUUCAUUUAUUCAUCCAAUAAACAUUUACUGA
>Novel:chi-miR-125a-3p
UCAGGUGAAGCACUGUAGGGAGAGGCGGCAGGGCACCUCUGGGACCUGCGUGUGUCCGCAU
GUGCCCGUGCGGAGC
>Novel:NC_037546.1_4613
AAAAAGCUCAUUUGGAAGUUUCCAUAAGAUCUGCUGGAAACACCCGAACGAACUUUUUGG
>Novel:NC_037564.1_40415
GAUGAAGAAACUCAAGAGAGGAAAGUUGCUCUCUUGAGUUCCUUCAUCUU
>Novel:NC_037550.1_18169
GUUCUCCAGGCUUAGGAGCGUGGGGAGUGUGUGGUGGAGGCUUAGGCAAAGACAGGAAUG
CAAAAGGAAGCCUGGGGAGCCC

>Novel:NC_037546.1_5554
AAAGUGUGUUUGGGUUUUUUUCUUAACAUCUUAUGGUAAAACCCGAACAAACUUUUU
>Novel:hsa-miR-4743-5p
GGGGAGCAGACGAUGUAUGCCACAAGCAGAGGCCGGAUUCUGUGCCCACCA
>Novel:hsa-miR-1265
GAGGAUGUAGCUCAGUGGUAAAGCACAUGCUCAGCAUGCAUGAGGUUCUGGGUUCAGUCCC
CAGCAU
>Novel:hsa-miR-3157-5p
CCUGCUUUCCUUCCAGAGCCCAACGUCUUCGUCAUCUUCAGCCAUGGACUGCAGGGC
>Novel:chi-miR-378-3p
ACUGGACUUGGGGUCAGAAGGCCUGGGCAUAGUUCAAGUUCUGGGUGAAUUGCCUACCUCC
UCGGACCGCCACGUUUUAUCU
>Novel:NC_037569.1_47935
AGAGGAAACCAGCAAGUGUUGACGCUAAACUGGCAGAAACAUCAAUACUUCCUGGCUCCCC
CACU
>Novel:NC_037569.1_46177
CCAUGCUCCAAGUGUCGCACAUCUAUGCUUCCACCAAGGAGAUGUACGGCAUUUAGGGGCA
GAACU
>Novel:hsa-miR-1289
GCGAGCGCAGGACGCCGCGCGCGGGAGGCACGGAGUCCGGCUCUCGGCU
>Novel:NC_037569.1_47148
AACGCGCCGCUGGGCUGCUAGGCUCGGCAGUGCGCUAGCUGCUCCGUGGCGCUUC
>Novel:NC_037547.1_8396
AGACGGAGGACUGAGGAGUAUAAUGUCCUCAAUAAGUCACCUAUCUAU
>Novel:NC_037565.1_42321
UUCGGCCUUCAAGGUUCCUGCACCUAAAACAUCCGGAAAUCCUGUCAACAGUGCAAGGAAG
CCUGGUUCAGCA
>Novel:NC_037548.1_11059
GCUGGGCAGAUGGCCUCAGCAGAGAGCCAGGCUCCCCAGUCA
>Novel:NC_037558.1_32782
CUGAGUAUCCAUUAUGUGCCAGGUACUGGUUAUGACCCUAGCACUUAUGGAUAAGGCACUG
>Novel:hsa-miR-4687-5p
GGACUCAGCAGGUCAGAAAGGAGCCCUCUGAGCUUCAG
>Novel:NC_037548.1_12213
AAAAUGUUCGUUUGGGUUUUUCUGUAAUAUCGUGUAUAAAAACUCGAACGAACUUUUU
>Novel:NC_037548.1_11028
AGAAGGAGGACAUUAAGGCCAAAAAGUUGGUGAACAAACGUUUCCCUCUGGCUUCCGUCUC
GCUCCCUCUCU
>Novel:NC_037562.1_38320
UAGGGGGGACCCUGGAUCCUCCAGUUUGGAAUGUUCUUAGGAUCUUCUCUCCCCUCCCCC
>Novel:oar-miR-3957-3p
GCGCACAGCCUCCUCUUCCUUCAUGUCAGGUUUCUGGAUUCAGGAUUGUUCUGUCGAUGGU
GGUGGUGGUGGUGGUGUGGUGG
>Novel:NC_037545.1_503
GACCCAUAGAAGCGAGCUUGUGCGGUCCACUUCACCACAAGAUCGGAUCUACGGGUU
>Novel:NC_037565.1_42764
AGGCAGGUGGGUUCUGCUCCCAGAACCAUGAAGCACGAGGCCAGCAGAGCCCAGCUGCCAA
A
>Novel:NC_037564.1_40258
CCAUGGGGUGGAGGACUGCGUCUGCCUUCCCCGUGGGCAGGGGUCUUCCCGUGGGGUGGAG
GACUGCAUCUGCCUUCCCCGUGGGC
>Novel:NC_037545.1_2499
GCAUUGGUGGUACAGUGGUGAGAAUAACUGCCUUCCAGAGAGA
>Novel:NC_037547.1_8180
GCCAGUCAUUCUCUCUCUCUCUCAAUGGUUUUAAGUUGAAAACAUUUGGUGGGUGAGGAAG
GGUGAAGAGGUCA

>Novel:NC_037557.1_31285
UUUUCAUUAAUAGUUUUGGGAGCCUUGUAUAAUAAUACCCAAACUGUUAGUGAAGAACU
>Novel:NC_037548.1_13397
AAAACGUUCGUUCGGGUUUUUCCAUACCAUCUUACGAGAAAAACCCGAAGAACUUUUUGC
>Novel:NC_037550.1_18832
CCCGGAACGCGUGGCUACAACAUAUGGUGCAUUGGCCGGGAA
>Novel:NC_037551.1_19555
AUGCAGGGCAAACUUCCUGGGUUUAAAAAAUCCUCUGAAUCAGGAAGGUUCUUUGUCCUGC
UCUGA
>Novel:NC_037553.1_24444
UCCUCAUCUUUCUCUUAACCACAGGAAGAUGAGCUGGACA
>Novel:NC_037551.1_19947
GCUUGACGGAACCAUGUUUUACUCAUACCUGGCAACCACAUGGUUAGAUCAAGCACAA
>Novel:NC_037549.1_14837
GUAUUGAGUUGGCCAAAAAAUUUGUUGAGGUUUUUCUGUAAGAUGUUAUGGAAAAACUUG
AGUGAACUUUUCUGGCCAACUCAAUACUU
>Novel:NC_037546.1_4132
AGGCCCUCCAGUCCCUGUUGUAGCUGAGCAAGGUGGUCAUAUCCUCCUUGUUCAGUUCAAA
GUCAAAGAUCUGGAAGUUCUCA
>Novel:hsa-miR-6514-3p
CUGCCUGUCUGUGCCUGCUGCCCAGACUUAUCUGGGAGCAAUCUAGACUUCGAUG
>Novel:hsa-miR-4667-5p
CCUGUAACACUCCCUUCAAAGGCCUGGGAGCACCGACCACGGGUCUGCUGGUCUGGGGAGG
GGUGGAAGGAU
>Novel:NC_037549.1_14266
AGGCGCUUUCGGUUGGGCCGAUUCCCGCCCGCUUCCUCCUAC
>Novel:hsa-miR-6748-3p
GUCUCGACAGCUGGACAGCCAAGUAAGAAGCCUUCGACACUCCUGUCCUGCAGUCGGAAUC
U
>Novel:hsa-miR-6885-3p
CUUUGCUUUCUGAUUGUAGGGCCCUGGAAUUCCUGGGUCUAGUGCCUGUGCAGUGCUGUUU
GGA
>Novel:NC_037558.1_32528
CAGUACCCCUGACUUCUCAGAGGCGCGAUGAAAAGCUCCCUGAGGUCACGGGCACAAGU
>Novel:hsa-miR-3714
GUCUAGUUUGCCCUUUUGAAAGCCAGGAGGAAGGCAGGCGGGGAAGC
>Novel:hsa-miR-3173-3p
UGUCUCUUCUUCCAGAUGUGGCCAAAAGGAGGAAGCUGAACUCAGG
>Novel:hsa-miR-152-5p
GCCUGAGUUCACAGCCAGAACUUGCCCGCAGCUAGGUGAAGACCAGUGAGCCAGGUUCUGA
GCCUCAGUCUCCU
>Novel:NC_037550.1_18353
CCAAAAAGUUCUUUUGGGUUUAUAAGACUAUAAGGAAAAACUGGAAUGACCUUUUUGG
>Novel:hsa-miR-4524a-3p
UCUUGGGUCUGUCCCACCCAACACUGCUGAGGCAAGAGACAGAUCCAAGGAC
>Novel:NC_037550.1_18908
AAAAAUUCACCUUUGGGCUUAAAAUAAAAUCAGAAAGCCCAAAAGGAGAAUUCUUUG
>Novel:NC_037557.1_30571
AAAAAUUUCAUUUGGGUUUUUCUACAACAUCUUAUGAAAAGCCCAAAUGAAAUUUUUUG
>Novel:NC_037545.1_1619
UGCGGACCGGCUGCGUGGCGAGAUCCCCGCGGGAAUAGCUCGCGCGGGGGCUGCUUCGUGA
CGCUGGUCGCUCC
>Novel:NC_037550.1_18703
GAUUAGGUCUAGAAGAGAGGAUGAAACAUCCUAAAUUCUAGACACUUUACAU
>Novel:NC_037555.1_26610

UCGUCAUGACCCUUUCUCUCUCUCAUGAAAUCUCAACCAGCCAUGGUCCAAUCAUUUCAAG
UGUUGCCAGGGUCAUGACUUUG
>Novel:NC_037562.1_38970
GGUGGAGACCUGGGGGGAGGGUGGGGGGCUGAGGGGGUGGAAGGUCCUCAUGCCCCCCAUC
CCCUGUCCUUCCUUCUCUUCCCUC
>Novel:NC_037562.1_38317
GGUGGAGACCUGGGGGGAGGGUGGGGGGCUGAGGGGGUGGAAGGUCCUCAUGCCCCCCAUC
CCCUGUCCUUCCUUCUCUUCCCUC
>Novel:NC_037557.1_30645
UGCUUGGCUGGCUCACUGGAUGGAAGCAUAACUUCUUAUGCCAGAAGGAGCACUUAGGGCA
GU
>Novel:NC_037557.1_30644
CAGUUUUGCAUAGAUUUGCACAACUACAUUCUUCUUGUAGUGCAACUAUGCAAAGCU
>Novel:NC_037550.1_19285
UGGCUCCAGGCAGCCCGGCCCUUCUUGCCGUGGCCUGGCUGCCAGGAGCCCAGC
>Novel:NC_037568.1_45170
AGUCUCGAGACCCUCUCUCUCAGUUUUCUGAAAAGGCUGACUGAAACAGAGCUGAGGGCAG
GGAUACCUCGUCACUAC
>Novel:NC_037557.1_30640
CAGUUUUGCAUGGAUUUGCACAGCAGAAUAUCACACAGCUGGAUGCAAACCUGCAAAACUA
AC
>Novel:NC_037566.1_43556
GGCGCUGGACGAUGAGGGCAGGUGCACAUGAAUAGCCAUGGGCAGAGCACCUGAGUCCUCA
CUGUCCCCUU
>Novel:NC_037553.1_23290
UUCCCGGCCAACGCACCAAAUGCUUUAGACACGUGUUCCGGGAAAC
>Novel:hsa-miR-4765
AGAGUGAUCAGUUCCUGCAAUUCCUGGGCCACCUAGGACAUAAACAUAAAAUCAGGGACAU
AUCAAAUGUCAAG
>Novel:NC_037558.1_31855
UUUCUGGUCCUGUUUCUUGGUGCCUUCUUCCGACGGGGCUAGAAACC
>Novel:NC_037546.1_4112
AAGAAGUUCGUUUGGGUUUUUCCUAACAUCUUAUGGAAAAACUUGAACAAACUUUUGAG
>Novel:NC_037547.1_8573
AAAAUUCCUUUAGAUUUUUCCUUAAGAUGUUCUGGAAAAACCCAAACGAACUUUUC
>Novel:NC_037555.1_28238
ACUCAAGGCAAACAGCUAGUGAGUCCUGGAUCUGGGAUGCAGUCCAGCUGGUUGAACUUGA
GACC
>Novel:NC_037564.1_40338
GAGGUUUCCCGUGUAUGUUUCAUCAUAAAUAAAGAGAAGACAACACGGAUAACCUCUC
>Novel:NC_037546.1_4370
AAGUCUUCAUCUUUGGCUCAUCAUAGGAGUCUAGCAUUUGAUUUUACUAUGCUAAGAUGAG
CAGCAAGGUGAAGCUCCU
>Novel:NC_037557.1_30688
AGAAGAGAAGAGGACAUGGAAAGCAAAGGUAGCUGAGAAGUCACAGUGAUUAUCAAUCAG
CCAUGUCCUCAUUAACUGUCCUUG
>Novel:NC_037552.1_21756
UGCCCAUCCACUGCAGAACUUGUUAAGUUUCCAUCCAAGUUCCGUAAGUUUGAUGGGAAGA
>Novel:NC_037564.1_40573
UCAUGUGUACAAUGACAGUAGAAUCUACUUGUGGUUUGGUGUCGGGAAUAAAAGAGAUUG
UACACAUGAAU
>Novel:NC_037547.1_10260
CUACCUGAAGCAGAAGCAGUUUUACCAGGGACCUAAAGAGGCUUCAGCUUCAGGUCUCU
>Novel:hsa-miR-3921
GAAAGGGCGUGGAGUUAUAAGACCUGGUGAAAACCCAGCUCUGAGCCUCAGUUCCCUCCU
>Novel:NC_037549.1_15761

GUCUUCUCCGAAGCAACGAUUUUCCACUCAUGCUUAGGGCUUUGUAUUGUAGCAUUUGGAG
AAGACAG
>Novel:NC_037567.1_43945
AGAUCACAACACUGUCCAGCAAGUUCCCCACUCCUCCCCGUGAGAGGGUGAUGCAUGCAUG
UCCCAGUGUGGCUGAUCAUG
>Novel:hsa-miR-6131
GGCUGGUCCAAUGGUAGUGGGUUGACAGAACUUACUAGACUAGGACUUAGAAGA
>Novel:hsa-miR-10398-5p
GAGGCGGGGGCACCAGGAACAGUGUGACUGUGGGGCUCCCCAGGCUUCCU
>Novel:hsa-miR-6770-3p
CCGUUGUCCGCGGCGCCCGCCUGCCCGCGAGGCCCCCGGGGGCGGCGGGAGGGCGUUGGCGG
CGCGGCACGGCCUGG
>Novel:chi-miR-24-3p
AGGCUCAGCAUUUUCUUCCUUUGGCUCAGGAGGCAAGGCCUGCUCUAAGGAGAGCCUGAUG
GCCUGA
>Novel:NC_037547.1_7205
GAAGGGGAAGCGGGAGAGGAGGAUCCGUGGGGGUCGAGGAGGACCCCGGCGUGCACUCUUU
UCCUCUCGCUCUCCGCUUCCCCUCUCUC
>Novel:hsa-miR-6734-3p
GGGUCGCAGGGCAGAGAGAGACCGCCCUUCCCCGCAGGCCUUC
>Novel:NC_037550.1_18131
CUCUUUCUCAAUGCAACCUGGAGAAGAGAGGAAGAGGGC
>Novel:chi-miR-7-5p
AGGAAGACGCGGUCGCGGCUGCUGCAGACUUUUGUGCCGCCCGCUCCUGCUCCUGACUCA
>Novel:NC_037564.1_40430
UGCUGCCAGGAGCUCAGGGCCUUGCGCUGUGUCUAAUGCACUAAUCCCAGUGUUGAGGCGU
CCUCUGGCUCCUGGCACCAAA
>Novel:chi-miR-24-5p
UUGGAGAAGGAGGUGGAGAGCAUGGGGGCCCAUCUUAAUGCCUACAGCACCCGGG
>Novel:NC_037547.1_8021
AAAGUUCAGCACGGCGGGAACCGUCUCCUCAGCUCCUU
>Novel:NC_037545.1_548
GAGUUCCUUCUCCAGAGUUUGUGACCUGGGAUAGGGCUCAA
>Novel:NC_037545.1_416
AGGGAUCUUUAGUUGCUGCAUGUGGGAUCUAGUUCCCGGAUCAGGGAUUGAUCCUCC
>Novel:NC_037557.1_31280
AUUCAACCAACCAUGGAUUCAGUCAACCGCAGAAGGUAUAUUAAGUUUGGAAUUCGUGGUU
GGUUGAAUCU
>Novel:hsa-miR-4800-3p
UGUGACCAAGCCACGAGGUUGUCACUUGAGAAAUCCGUCGUCACCUGGUCACUGU
>Novel:hsa-miR-3689d
AGGAGGUGGACGGAGGAGAUUAUGGGCCGGGUAUGGUCAUUGCCCAGCAAAGGCAUCCAGA
CCUCUCUUGG
>Novel:NC_037547.1_9224
CCAGGGCAGUUCAUGAUGUCAGUCCAAUUAAGAAAGAACUGCUCUGGCA
>Novel:NC_037554.1_25273
AAGGUCAUUUGGGUUUUUCCAUAAGAUGUUACCAAAAAACCCAAACGUACUUUU
>Novel:NC_037562.1_38718
GGAAGAGUUUGGGAAAGGUCAAAAGGAGACACUACGUGUCCGACCACACCCCAGAAUCCUU
CUCU
>Novel:hsa-miR-3148
CUAGCUCUGGUUCCCCUUUGAUAACCAAAUCAUUGAUAAGGACAAGAGAGAGAGACAGGGA
GCAAGGGAAAAAGGAACCAGGGCAGAA
>Novel:NC_037548.1_12850
GAGGGGCUUUCCGGUGUAAACCGGAAAGCCUGCUAGACAAAU
>Novel:NC_037555.1_27478

CCAGGCACACUCAGCCCUGGGUCUGCCCAGGAAGAGUGUGUCUGGGC
>Novel:hsa-miR-203a-5p
AAUGACCAGCCUGGCCACGGGGUUCAAAAGUGAAAUUCAACAAGUGGUUCAGGUUGAUCUU
CUU
>Novel:NC_037547.1_8898
AGGCCAGAGAUGCUUCCUUUGCUCUAACGGGUUACCUUGGGUUGCCUUUUAGUUUUCGGUA
AUUUUUGUGGGAGCUUUCUUUCCU
>Novel:NC_037546.1_2960
UUCUAGCGACUGAAGGAAUCGUCUAUUCCUCUCUGAUCGCUCCCUCGGUGUUUUGGGG
>Novel:NC_037553.1_23729
UUUGGGGUCUGGGAACCUUGACCUGCAGGUGCCCUGAGGCCUGGCUCUCUUCCCCAGA
>Novel:NC_037569.1_48122
AAAAUCUGUGUUGAUCGUUUUUUGAUUUUGCCAUUUAUGGGGGUAAAAUCAUGACAGAUU
GACAUGAACAGUU
>Novel:NC_037547.1_8993
AGCACCUUCCCCCCAAUCCUGCUCUGAGCCUGGGUGUCGGCAAGAGGGGAGGAAGGGCUUG
>Novel:hsa-miR-6890-3p
GGCGGGGCUUCCAGGCCGUUGGUGUUGGGGACUUCCGGACUCACUGCCUGGCUCCUCUUUC
C
>Novel:NC_037553.1_23923
GCCAUUGAUGAUCGUUCUUCUCUUCCUUUUGGAGAGUAAGAGGGAGAGAGUGUAGUCUGA
GUGGUUA
>Novel:hsa-miR-4740-3p
GCCCGAGACAACAAGAAGACGCGCAUCAUCCCUCGCCAUUUGCAACUGGCCGUGAGAAAUG
AUGAAGAGCUCAACAAGUUACUCGGGGGU
>Novel:hsa-miR-3129-3p
AAACUAAUUUUUUGAGGCCUUGCUUCGGCAAGGCUAACUUAUAUUGGUUAAU
>Novel:NC_037564.1_40729
UUGGUAGAUAAGUAGGUCUUGAAUUCAUUUUGCACACAAGACCUGUUCUAUCUACCUU
>Novel:NC_037568.1_45619
ACUCGCGGGGAUCUCGGGGCCAACACGGGCGGACGGGUAGUUUGUGCGAGUCCCUGCCCGC
CACCCCCCCGACUCCCCAUGCGGGACC
>Novel:NC_037555.1_26458
GCCCAGACACACUCUUCCUGGGCAGACCCAGGGCUGAGUGUGCCUGGAGGG
>Novel:NC_037562.1_38368
GCGGGGGGCCAGCUUCUCUCAGGCCUCACUGGCCGUCAUGCCACAGCGGCUGGCCUGGGAC
GCUGCUCCCUGCUG
>Novel:NC_037565.1_41975
UCAAAAGCCCUAUAAGUAAGCUAAUGUAUUUACUCAUAGCUUACUUAUAGGGCUUUUGAUU
>Novel:chi-miR-221-5p
CAAGGACUGCCAGGUGUGACAGGUUAGCCUGGCAACGUUCUACUC
>Novel:NC_037558.1_33194
CUCAGCAGAUAGCCUCCUGGGGGUCUAUUUGCAGGUUGAUUCUUGGGACUGUCUGCCGAGC
U
>Novel:hsa-miR-1251-3p
GUCAUCCGUGGGGGAAGCGGGUGCGUGCUCACGUUCUCCAGCUUUGCGCAGUGGCAG
>Novel:NC_037558.1_32328
AGCUCGGCAGACAGUCCCAAGAAUCAACCUGCAAAUAGACCCCCAGGAGGCUAUCUGCUGA
GUCCC
>Novel:NC_037547.1_8236
UGAGAGUUGAAAGUUCAGAUGAGAGCUGAACUUUUUUUUCUUGCU
>Novel:NC_037553.1_24363
CCUUCCAGGGAUCUUCUCCUUGCUGCAACAGGGAGAUCACUUUGGGUUUGG
>Novel:NC_037553.1_23783
AUAAAGUAACUGAGACGGAUCCCACAAACGACACAUGACAAUCAGUCUCAUUGCUUUAUAA
>Novel:hsa-miR-7113-5p

AUUGUUUCCAGCCCAGGCACUCUCAGGCUCCGCGCGGCCCCAGGGAGAUGCUGGACUGUGU
GC
>Novel:NC_037561.1_36447
CCAGCCCCAUUUCCCUGUGAUUCUAACAAAUUUGAGAAUCGAGAGACUCGGGCUGUUU
>Novel:chi-miR-22-5p
AGUUCUUCAGUGGCAAGCAACAAAAACCGAUAGGCCACUGUUGUUCGGCCAGCGAAAAGGA
AAUG
>Novel:NC_037549.1_15080
UGGGCAGAGAGCACAGUCCAUGCCAAAGCACUGAAACCUGAAAGAGGAGUGAUUGGAGAGA
AAGGAUCAUUGUGCUUUGAGCAAAGG
>Novel:NC_037553.1_22911
AGAACUAAACUUCACAGGUCUGACACUGACACAACCUAUGGAAUUCAGUUCUCA
>Novel:NC_037548.1_11085
ACUAGUGCUUUUUUCUUAGGACAGAUUAAGAGAAAGGGCUCUGGAGC
>Novel:NC_037567.1_44437
AGGUACUCUGUGGAUAUCCCCUUGGACAAAACUGUUGUCAACAGGAUGUCUUCAGAGACCC
UG
>Novel:chi-miR-151-5p
UCGAGGAGCUCACAGUCCUGGGGCAGAAGAGAAUGUACUCCUGGCUGUGAUGGAGG
>Novel:hsa-miR-135a-3p
AAUAGGGAACGUGAGCUGAGCCCCGCAUGAGUGGAGGAACUUCAUCUUCAGUUUCUAUUUG
>Novel:hsa-miR-4501
UCCCAGAGCCUUGUUGCCAGAUGGUCCCAUGUGACUGAACUCUGGCUGG
>Novel:hsa-miR-4443
AUGGAGGCGUCCCUGCAGGAGCUCAAAGCCAGCCCGCGCAUGGUGCCCCGCGCCGUGU
>Novel:hsa-miR-6787-3p
GCUCAGCUCUCCAAGGUUGCCUUGCUGGGAGGGCAGACUCUG
>Novel:hsa-miR-6818-3p
CUGUCUCUACAACUCUGCCACACUUCUCAAACCAGAGGUGUUUUGAGGAUUUUUGGAAUUG
GGU
>Novel:hsa-miR-4318
CAUCUCUGGCCACGUCAACAACCCCUGCACUGUGGAGGAGGAGAUGUC
>Novel:NC_037565.1_42848
ACGAGUGCUUCAAGUGCGGACGGUCUGGCCACUGGGCCCGGGAGUGCCCCACUGGUGGAGG
CCGCGGUCGUGGAAUGAGAAGCCGUGGC
>Novel:NC_037567.1_44092
ACCCAAUGAUGAAGACUUAGGUUGCUUCUAUGUCUUGGUCAUUGUAAAU
>Novel:NC_037565.1_42322
AGCUUUGAUGAUGAAGAAUCAGUGGAUGGAAAUAGGCCAUCAUCAGCUGCUUCGGCCUUCA
AGGUUCC
>Novel:hsa-miR-3153
AGGGAAAGCCGAUCCGGAUCCUGUGGUCUCAGAGGGAUCCGUCUCUGAGAAAAUCUGGUGU
GGGAAACGUCUUUG
>Novel:NC_037549.1_16021
CCUCCUGACGACCUUCUCUCUGUCAGUAUCCCUCUACACCCCAGGGUAGGUCAUCAGGGAG
AC
>Novel:hsa-miR-4308
GAGCCCACUGUCUAGCCUAAGGAGCCCUGGACAAGGCUGCU
>Novel:NC_037558.1_32790
UCAGCAGCAGUCUCCUUCCUUCCAGAAGAGAAGCUGUUGAGC
>Novel:chi-miR-23b-5p
UGGGUUCCUCCUUCUUCUAGGAGAUUCUUUUUCAGCAGGUUUAAGUGGGAUCUAGG
>Novel:NC_037562.1_38969
GUCCUUCCUUCUCUUCCCUCCCCACAACCAGUUAAAAUCCUCUUAAAAAGCCUACCACAGGG
GUGAGGCUGGUGAGGGAGGGACUA
>Novel:hsa-miR-6828-3p

CUCUGCUCUCCGACCCUGGUUUUAUCUUGAUCUGGCUGAGGGGCCACAGGAGGAGUAGAGG
G
>Novel:NC_037551.1_19577
UGGAAAAAGGAGGAAAGCUGUCUUUCCUCUGUCAUUUUGUAAC
>Novel:NC_037556.1_28412
AUUGGUGGUUCAGUGGUAGAAUUCUUACCUGCCACGCAGGAAGCCCAAAUUCAACUUAGCG
ACUGAACAACA
>Novel:NC_037562.1_38318
GUCCUUCCUUCUCUUCCCUCCCCACAACCAGUUAAAAUCCUCUUAAAAAGCCUACCACAGGG
GUGAGGCUGGUGAGGGAGGGACUA
>Novel:NC_037564.1_40393
CAGUCAGAUUGUAGCCAGGGGACAAAGGCUCUGCAGAAAGGC
>Novel:hsa-miR-4468
GGAGCAGAAGGGCAAAAGUAAUUUGACCUCCUCCCC
>Novel:NC_037548.1_11604
GCCUUCAGGACCUCAGACCAAGUUCUGCUCAGAAAUCUCUUUGGUCGUGUCUCCAAGGCGA
>Novel:chi-miR-1197-3p
GAGGACACGGUGGCCAUGAUGGGGGUCUUCAUGCGGGCAUGGGUCCGGGCAAGUCCUCGGA
AUAGGAGCCCGUCCUCUG
>Novel:hsa-miR-4632-3p
AGACCGGAGGGGCCUCUCACCGAGCCGCCCUUCUUUCUCU
>Novel:NC_037563.1_39591
GGGGAGCUGGGGAUAGGCCUGGUACCCGGAGGAGGCUGGGUGGCUUCCCCAGAGUCCUCUU
>Novel:NC_037550.1_18584
ACGAGUGCUUCAAGUGCGGACGGUCUGGCCACUGGGCCCGGGAGUGCCCCACUGGUGGAGG
CCAGGGUCGUGGAAUGAGAAGCCGUGGC
>Novel:NC_037562.1_38397
AUACUGCAAGCCAUAAAUUAUGGAGUUUAUCGUAAGGACUGGCUUCCAGUCCUCU
>Novel:NC_037549.1_15051
GAGCGAGAUCGACUGACUUCCGGCCAGGCCGUUGUCUGGGUGGCGCGGUCGAGUCAUCGUU
GGGCCU
>Novel:hsa-miR-4768-5p
GUGGGUGAGAGAGCUGUAUCCAUUUUGCUGUAUCCAUUAUCCAAUCACAAUUAAUUCUCUC
UCCCUUUUCCU
>Novel:NC_037545.1_1494
AAUUGCUGCCAUCACUGGGCAUCAGCCCUCCAGUGAUCAGCAAAAUA
>Novel:NC_037545.1_1780
CCGGCGCCGGCGGUCCCUGCGGAAGGGGCCAACAGCAGCCCGCAUCGGGACCGCGAGCGCCG
AGC
>Novel:NC_037563.1_40056
GGUAAUCAAGCUGUGUCUACAGGAGUAUUUUUUCCUAAGAAAUAGCAGUGAGGAUACAGU
GGAAAAG
>Novel:NC_037551.1_20562
CUGCAAAGACUGAGUUUGCUCUCUCUGGGAGGAACCCAAUACAUCCCAGGGACAGAGUCCU
CUACAGAUUGAA
>Novel:NC_037562.1_39081
ACACUUCCGGGAUUCUGGGCGGAAUAAGGGUGCCGCACAGCCUCUCGGGAGUCGUAG
>Novel:hsa-miR-4508
GCAGGUCAGCCCGGUCCUCCCUGGGCUCCGCCCCUCUGUGACGGGGCUGCACCUGGAG
>Novel:NC_037546.1_4475
GGGGAGGAGGAGUGACUUAUUAGGCAGAGGAACAAGGCUGUAUAGCUAGUCCCUUCCUCCU
CUCUCU
>Novel:hsa-miR-4283
CACUCAUUCGCGAGACCGCCGAUCCCGCCUCCACGGUGAUCAGGUUAGUGUGCGCCGCGGG
UGCUGGGGGCUCGAGAACCGAGCGGA
>Novel:NC_037569.1_47777

AAAGUUCGCUUGGAUUUUUCCAUACCAUUUUACAGAAAAGCCCAAACGAUCUUUUU
>Novel:NC_037553.1_23738
CCUUCCAGGGAUCUUCUCCUUGCUGCAACAGGGAGAUCACUUCAGGUU
>Novel:NC_037564.1_41300
GUUUCAUUCAGGUUUUCCAGUAAGAUGGUAUAGAAAACCCAAACGAACUUUU
>Novel:hsa-miR-6516-5p
UUUGCAGUGAUUGGAGCCCUGCUACAGAGCAGCGGACCCAAGAGGACUGCACUCU
>Novel:NC_037549.1_16794
AACCUUGCUUAUUGUUGCUUAAUUCCAGUACCAAUGAGGAAAGCAGAC
>Novel:NC_037545.1_434
GGCCAGGAUUCCACCCCUGGUCAGGGGAAUAAGAUCCCAUAUGCCUC
>Novel:NC_037553.1_24731
CCACUGGCCCCACUCAGGGCUGGCUCUCCCACCUCACAGCCCUGCUGGGGGUAGCGGCA
>Novel:NC_037558.1_32781
CUUAUGGAUAAGGCACUGGGGCUGGCACUGUGGACACAAACAGUGAACCUUCCUCAGAAAA
AUCCAGAAGAC
>Novel:hsa-miR-6867-5p
UCAAAAACAGCCGCCACCACCACCAUCAUCUCGGAGCAGUGUGUGUGUGUGUGUGUGUG
UGUGUGU
>Novel:NC_037558.1_32628
CUUUUCGCUGGCCGAACAACAGUGGCCUAUCGGUUUUUGUUGCUUGCCACUGAAGAACU
>Novel:NC_037546.1_2789
GGCUCCAAGGAGCAGAGUGAGAGCUGAGCACCAGGUCAUGACUCUGCAGUCCAGCCUU

Supplementary file S7.
List of novel miRNA mature sequences
>Novel:hsa-miR-5193
GCCUCCUCCGUCGGCCUC
>Novel:NC_037563.1_39396
ACACGGACAGGAUUGACAGAU
>Novel:NC_037563.1_39878
AUCUGUCAAUCCUGUCCGUGU
>Novel:NC_037547.1_10635
CAGAACUUAGCCACUGUGAA
>Novel:hsa-miR-1237-3p
GCCUUCUGACUCCAAGUCCAGU
>Novel:hsa-miR-6832-3p
GCCCUUUUCCCCGCCCCU
>Novel:chi-miR-202-3p
AGAGGUGUAGGGCAUGGGAA
>Novel:NC_037556.1_30106
ACUCACCGACAGCGUUGAAUGUU
>Novel:NC_037549.1_15660
ACUCACCGACAGCGUUGAAUGUU
>Novel:hsa-miR-2115-5p
AGCUUCCAGUCGGGGAUGUUUACA
>Novel:NC_037559.1_33968
CCUCAAGGAGCUUCAGUCUAG
>Novel:NC_037549.1_16300
UGUCUGUCAAUUCAUAGGUCAU
>Novel:hsa-miR-3672
AUGAGACUCUGGCAUGCUAAC
>Novel:NC_037568.1_45248
AGCGGGACUUUGUGGGCCAGUU
>Novel:NC_037567.1_44073
AGCCGGUCGAGGUCCGGUCGA

>Novel:chi-miR-2284a
GAAAAGUUUGUUCGGGUU
>Novel:NC_037569.1_46559
ACGGUCCUACACUCAAGGCAUG
>Novel:NC_037568.1_44905
CUACCUGCACGAACAGCACUUUG
>Novel:NC_037560.1_35426
CGCGCCCUCAGAGCAGAC
>Novel:NC_037569.1_46961
AAAAACCUGAACGAACUCUUU
>Novel:NC_037565.1_42672
AACUGUACAAACUACUACCUCA
>Novel:NC_037547.1_10214
CAGCUAUGCCAGCAUCUUGCCU
>Novel:NC_037547.1_8560
AAAAACCUGAAUGAACUCUUU
>Novel:NC_037554.1_24951
AAAGAGUUCAUUCAGGUUUUU
>Novel:NC_037550.1_17628
GCUCCCCACAGACCCAGAGCCG
>Novel:NC_037555.1_27844
GGCAUAGCGCACUGCAGCCCA
>Novel:NC_037547.1_7559
GCUGUUAGCCCUAGCCCCGCA
>Novel:hsa-miR-6741-5p
AUGGGUGCGAUUUCUGUGUGAGA
>Novel:NC_037563.1_39679
GCGCCCCAGAAUUCAUCAUUUUC
>Novel:NC_037569.1_47087
UCAAAACAUGAAUUGCUGCUG
>Novel:hsa-miR-548a-3p
AAAAACUGAAUGAACUUUCUGG
>Novel:NC_037567.1_44260
CCAAGUCCAUCCUUCAGCUCUA
>Novel:NC_037567.1_44693
UAGAGCUGAAGGAUGGACUUGG
>Novel:hsa-miR-652-5p
CAACCCUAGUGGCGCCAUU
>Novel:NC_037569.1_46992
UCUCUCAACCAACCAGAUGUU
>Novel:NC_037548.1_12197
AAAGAGUUCAUUCAGGUUUU
>Novel:NC_037556.1_29844
AUUCCUUGGCUGUGUCUGAGCACC
>Novel:hsa-miR-12136
CAAAAAGUUCGUCCAGAUUUUU
>Novel:NC_037560.1_35403
CUCGUCUUGUCUGAGGAA
>Novel:NC_037546.1_5830
AAAAAUCUGGACGAACUUUUUG
>Novel:NC_037564.1_41423
AGAGUUCAUUCAGGUUUUU
>Novel:NC_037547.1_8583
AAAGUUCAUUUAGGUUUUUCUG
>Novel:NC_037551.1_19765
AAAAAUCUGGACGAACUUUUUG

>Novel:NC_037548.1_12259
GGAGAUGGAGCCAGGGCCCUAA
>Novel:NC_037552.1_22398
AUUUUUCGUUAUUGCUCUUGA
>Novel:NC_037545.1_1192
AGAGUUCAUUCAGGUUUUU
>Novel:hsa-miR-6776-5p
UCUGGGUGAGCAGUCGGAGA
>Novel:hsa-miR-5190
ACAGUGACCAGGUGACGACGGAUU
>Novel:NC_037557.1_30647
CUACCUGCACUGUAAGCACUUUG
>Novel:hsa-miR-6125
ACGGAAGGGCAGAGAGGGCCAG
>Novel:NC_037565.1_42254
GGACCCGGGAGCACAGCCCA
>Novel:hsa-miR-4691-3p
GCAGCCACACAGAGCGCCCGG
>Novel:hsa-miR-6499-5p
CCGGGCGCUCUGUGUGGCUGC
>Novel:hsa-miR-6777-5p
CCGGGGAGCCCGGCUGGC
>Novel:NC_037555.1_28111
CCAAAAAGUUCAUUUGGGUCCUU
>Novel:chi-miR-3432-5p
UGCGGGAUCUUUAGUUGUGGCG
>Novel:hsa-miR-1290
AGGAUUUUUAGGGGCAUUA
>Novel:NC_037545.1_169
AGAAAACCCGAACGAACUUUUU
>Novel:NC_037567.1_44300
AAAAGUUGGUUUGAGUUUUUCU
>Novel:NC_037567.1_44728
AGAAAAACUCAAACCAACUUUU
>Novel:NC_037553.1_23200
GACCUGGCUCCAGCCCAGCCCA
>Novel:hsa-miR-762
UGGGCUGGCCUGCCUCCAAGC
>Novel:NC_037549.1_15917
CAUCGUUACCAGACAGUGUUA
>Novel:NC_037553.1_24045
AACCCAAACAAACUUUUU
>Novel:hsa-miR-760
AGGCUCUGUCCCUCUGUCCAAUU
>Novel:hsa-miR-1470
GCCCUCCGCUUCUGGUCC
>Novel:hsa-miR-3919
UCAGAGAACCGUGCCGAGGA
>Novel:NC_037549.1_16296
AUCCAAUCAUUUCUCCUAGCCUG
>Novel:NC_037557.1_31157
ACUUUCCCGGGACUUGGAGCGC
>Novel:NC_037552.1_22185
AUCGCAUCAACACUCGUCCGUU
>Novel:hsa-miR-3670
AGAGCUCAGUGCCUACCCUGCA

>Novel:NC_037556.1_29648
GAGGACUCAGCAGUAGGCUCU
>Novel:NC_037550.1_17830
AAAGUUCAUUUGGGUCCUU
>Novel:NC_037550.1_18986
AAGGACCCAAAUGAACUUU
>Novel:NC_037561.1_36221
GGCCAGGGGUGUGUCGGGCUCU
>Novel:NC_037549.1_14209
AGAAGGGCUGCUAGCAGGAAG
>Novel:NC_037556.1_29299
UCCGUGGGCUCCGGGCUGGGGU
>Novel:chi-miR-214-3p
CACAGCAGACUCAGGACACAGA
>Novel:hsa-miR-548c-3p
GAAAAAUCCGAACGAACUUUC
>Novel:chi-miR-499-3p
AAACAUCACUGCAAGUCUUAA
>Novel:NC_037564.1_41137
AAGAUGAAGGAACUCAAGAGAGC
>Novel:NC_037552.1_21285
UUAAGGUUUCUCCAGGUGAC
>Novel:NC_037546.1_4277
UGUCCAUUGUAUCUGGGGAU
>Novel:chi-miR-2284d
AAAACCUGAACAAACUUUU
>Novel:NC_037550.1_18493
UCCGGGAGUGUAGGGCGCGCUC
>Novel:NC_037546.1_6178
AGAAGGUUCAUUUGGGUUUUUC
>Novel:NC_037569.1_48167
UCGCCCCGCCUCUCUGCACGA
>Novel:chi-miR-326-3p
UCUCUGGGUCUGCUCAGCCCAC
>Novel:hsa-miR-3620-5p
GUGGGCUGAGCAGACCCAGAGA
>Novel:NC_037564.1_40643
CGGGGCGCGGCGCGGCGCGGC
>Novel:NC_037550.1_17382
UCCGGGAGUGUAGGGCGCGCUC
>Novel:NC_037549.1_16572
AAAACAAGGUCACGUCUCUCA
>Novel:NC_037549.1_15054
UGAGAGACGUGACCUUGUUUUG
>Novel:NC_037553.1_24733
UGGGCGGAGGUGGGGCGGGGGGCC
>Novel:NC_037555.1_27691
CCUCCGCCCCGCGCUCGCCUCG
>Novel:hsa-miR-4695-5p
AAGGAGGCUGGGUUCCUGCU
>Novel:NC_037547.1_8809
GUCUUUUUUUUUUUUUUU
>Novel:NC_037554.1_25908
AAAGAGUUCGUUUUGGUUUUUC
>Novel:NC_037564.1_40769
UCAUACAGCUAGAUAACCAAAGA

>Novel:NC_037553.1_23966
UCAUACAGCUAGAUAACCAAAGA
>Novel:hsa-miR-4779
CAGGAGGGCAACACGGAGCUCU
>Novel:NC_037555.1_27132
GCUUCUGCAGACUGGCUCCAAU
>Novel:NC_037550.1_18370
UCAUACAGCUAGAUAACCAAAGA
>Novel:NC_037560.1_35002
UUCCGGGCAGCGGGCUGAGGCG
>Novel:NC_037558.1_31888
GACUGGGCUGAAGGAGCCC
>Novel:NC_037567.1_44673
CACGUCCUCUGACCACAGAGG
>Novel:NC_037555.1_26952
UUAAUCCCCGACUGUCCCCUCC
>Novel:hsa-miR-6071
UUCUGCUGUACAUUCUGCCAUC
>Novel:NC_037556.1_30278
UGGCAGUCGCCCGCUGUGGCCA
>Novel:NC_037547.1_10894
CAGAGCCCAGGUCCCCGGCUCU
>Novel:hsa-miR-629-3p
UUUCUCCCGCCGGCUCCCUGGAC
>Novel:NC_037545.1_1343
UCAGUAACAAAGAUUCAUCCUUG
>Novel:NC_037546.1_4436
AAAGUUCGCUUGGAUUUUU
>Novel:hsa-miR-4663
AGCUGAGCGAUCUCCUCU
>Novel:NC_037558.1_33220
UCUUGGCCUCCUGCGUCUUU
>Novel:NC_037549.1_14776
GGCGCCAGCCGAGGGGGGCCUCUC
>Novel:NC_037569.1_46509
ACGGGUCACUCAGAACCCCGAGU
>Novel:hsa-miR-4270
GCAGGGAGCAGUAAUUCCCGGG
>Novel:hsa-miR-4707-5p
UCCCCGGCACCUCCACCA
>Novel:hsa-miR-1199-3p
CGCGGCCGCCCCCGCGGGC
>Novel:hsa-miR-4778-3p
GCUUCUUCAGCCCGGGCGGCCU
>Novel:hsa-miR-4707-3p
AGCCCGCCGCAGCCCGUCGUCA
>Novel:hsa-miR-10226
UCAUGUGUGAUCUGUCCCCAGG
>Novel:NC_037561.1_37446
AGUAUCGCAUCGACUUGGAGCAG
>Novel:hsa-miR-765
AGGAGGAGCUGGAGCGGC
>Novel:chi-miR-2331
GCCCUGCAGUCCUGGCCU
>Novel:hsa-miR-1253
AGAGAAGAAUGUUGAGCU

>Novel:hsa-miR-6834-5p
GUGAGGGACUGGGAGAGG
>Novel:chi-miR-500-5p
GAAUCCUUGCCCAGGUGCAUU
>Novel:hsa-miR-7704
GGGGGUCGGGGGGCGGGC
>Novel:hsa-miR-4691-5p
GUCCUCCAAGGUGCCCUUUUG
>Novel:hsa-miR-1827
AGAGGCAGGGUAGUGUAAUGGA
>Novel:hsa-miR-619-3p
GACCUGGAGCCCGACCGGGGU
>Novel:hsa-miR-4514
UCAGGCAGCUAGAGCCGGCGCCA
>Novel:chi-miR-1307-3p
ACUCGGCGUGGAACCCGGCGG
>Novel:hsa-miR-5004-5p
AGAGGACAGGACUGGGAC
>Novel:hsa-miR-591
CGACCAUGGCUGUAGACUGUUA
>Novel:hsa-miR-4675
UGGGCUGUGCUCCCGGGUCC
>Novel:hsa-miR-3074-5p
GUUCCUGCUGAACUGAGCCA
>Novel:hsa-miR-4447
GGUGGGGGGAGGCGGGGGAGA
>Novel:hsa-miR-6825-5p
AGGGGAGGACGGAGGAGG
>Novel:chi-miR-125a-5p
GCCCUGAGUUCAGGCAGCAGUU
>Novel:oar-miR-134-3p
GCUGGGCUGGGCUCUGGCCU
>Novel:hsa-miR-6728-3p
GCUCUGCUCCAGCUUCUCCU
>Novel:hsa-miR-6755-5p
CAGGGUAGGCACUGAGCUCU
>Novel:hsa-miR-4520-3p
AUGGACAGAGGAGCCUGGUGGA
>Novel:hsa-miR-3654
CACUGGACUGCAAGAAUAC
>Novel:hsa-miR-4720-5p
ACUGGCAUCGUGAUGGACU
>Novel:hsa-miR-6715a-3p
ACAAACCACAGUGUGCUGCUG
>Novel:chi-miR-130a-3p
CAGUGCAACUUCCGGGCCU
>Novel:hsa-miR-2110
CUGGGGAAAGCAGGUGAGGGU
>Novel:hsa-miR-203a-3p
GUGAAAUGUUUAGGACCAC
>Novel:hsa-miR-4746-5p
UCGGUCCCUCGGGCCAGGGCAG
>Novel:hsa-miR-548v
GGCUACAGUCCAUGGGGU
>Novel:hsa-miR-759
ACAGAGUGCAGACCAGGUCU

>Novel:hsa-miR-372-5p
GCUCAAAUAUACAGCUUGUUC
>Novel:hsa-miR-3064-5p
UCUGGCUGGCUGGCGCGGGCU
>Novel:chi-miR-103-5p
AGCUUCUUUGGCUGCAGGGU
>Novel:hsa-miR-486-3p
AGGGGCAGAGGUGGACUGACAGU
>Novel:hsa-miR-6077
AGGAAGAGACCUGUGGACCU
>Novel:hsa-miR-12135
GAAAGGUUCGUUUGGGUUUU
>Novel:hsa-miR-198
GGUCCAGAGUUGCCAGAG
>Novel:hsa-miR-5681a
AGAAAGGGCUGCUGUGCC
>Novel:hsa-miR-6770-5p
GGAGAAGGGCUGGGGCCU
>Novel:hsa-miR-9900
UCAGGUCCCUGUUCGGGC
>Novel:hsa-miR-548as-3p
AAAAACCCAAACGAACUUCUU
>Novel:hsa-miR-4434
AGGAGAAGCUGUGGGUGCU
>Novel:hsa-miR-5088-5p
AAGGGCUCGGCGGCGGUU
>Novel:hsa-miR-4442
UCCGGACAGCAGAGGCCCCGCCU
>Novel:hsa-miR-3909
GGUCCUCUCCAUCCUUACCCCAU
>Novel:hsa-miR-7106-5p
AGGGAGGAACUGGACUCC
>Novel:chi-miR-874-5p
AGGCCCCACUGUCACUGAUAU
>Novel:hsa-miR-6868-3p
UUCCUUCUCCCCCUCUCC
>Novel:chi-miR-127-3p
CCGGAUCCCCGAGUCGGGC
>Novel:chi-miR-758
AUUGUGACCACGCUGCAGGACA
>Novel:hsa-miR-4677-3p
UCUGUGAGCUGUGCUUGCCGGGU
>Novel:hsa-miR-21-3p
AAACACCAUUGUCACACUCCA
>Novel:hsa-miR-3974
AAAGGUCAUUCUGAAGCCU
>Novel:hsa-miR-10522-5p
AGAAGAAUGAGGACAGACU
>Novel:hsa-miR-548d-3p
GAAAAACCCGAACGAACUCUUU
>Novel:chi-miR-411b-5p
UGGUCGACCAGUUGGAAAGU
>Novel:hsa-miR-3202
AGGAAGGGGAGCCAAGGGAC
>Novel:hsa-miR-7845-5p
AAGGGACAGAGCCACUCU

>Novel:chi-miR-490
AAACCUGGAAUGAACUUUU
>Novel:chi-miR-34a
AGGCAGUGGCUUCGUCCU
>Novel:hsa-miR-766-5p
AGGAGGAAGCUGAGGCCU
>Novel:hsa-miR-6814-5p
ACCCAAGGACUGUAGCCC
>Novel:hsa-miR-4491
UAUGUGGAAGCUGACCAGAGA
>Novel:hsa-miR-4753-5p
CAAGGCCAGCUAGCUUCCCGAG
>Novel:hsa-miR-6501-3p
GCAGAGCAGGGAAGGUGUGUUU
>Novel:hsa-miR-10399-3p
AUCUCGGAAGAGAAAGGGC
>Novel:chi-miR-432-5p
GCUUGGAGGCAGGCCAGCCCA
>Novel:hsa-miR-5002-5p
CAUUUGGUAGAGCAUCAGA
>Novel:hsa-miR-4482-3p
GUUCUAUUUUGUUGGUUUUU
>Novel:hsa-miR-4641
UGCCCAUGGACUGUCCUCUUCC
>Novel:hsa-miR-505-3p
GGUCAACAUUCCAACAGCCUGG
>Novel:hsa-miR-3122
AUUGGGACUCCGAGCCCGGGC
>Novel:hsa-miR-149-3p
AGGGAGGGAGGAGUCAGA
>Novel:chi-miR-22-3p
AAGCUGCCAGUUGCAGAAA
>Novel:hsa-miR-136-3p
CAUCAUCGCAUAUUGACACAA
>Novel:hsa-miR-6765-3p
ACACCUGGGGGUCUGAGGGGC
>Novel:chi-miR-320-3p
CAAAGCUGGGUUGAGAGGU
>Novel:chi-miR-145-3p
AUUCCUGGGAAAACUGGAC
>Novel:hsa-miR-208a-5p
GAGCUUUUGCCCUUCUGC
>Novel:hsa-miR-10524-5p
GAGGAUGCGGAUGCAGAAC
>Novel:hsa-miR-6808-3p
UUGUGACCGGAAUUGAGGGCU
>Novel:hsa-miR-3169
AAGGACUGAUCCCAGAGG
>Novel:hsa-miR-4485-5p
GCCGCCUGCUCUGCUGCAGAA
>Novel:hsa-miR-6780b-3p
GCCCUUGUAAGCCUUUUU
>Novel:hsa-miR-4795-5p
UGAAGUGGUGCCUGGGGACUC
>Novel:chi-miR-106b-3p
CCGCACUGGCUUCCUCCU

>Novel:chi-miR-3958-5p
CGUCUGAGCUGAGGUCAC
>Novel:oar-miR-544-3p
GUUCUGCAGGCCCCUUCU
>Novel:hsa-miR-4802-5p
CAUGGAGGACUGGGUGCUGGGCAUC
>Novel:oar-miR-10b
GCCCUGUACAAUGCUGAA
>Novel:hsa-miR-7113-3p
ACUCCCUGCUCCAAACAUCCAU
>Novel:hsa-miR-1321
CAGGGAGGGAGCCGCGCGGGGC
>Novel:hsa-miR-4659a-3p
AUUCUUCUUCUCUUUCCUUCU
>Novel:hsa-miR-6830-3p
AGUCUUUCUGGAUCUCCU
>Novel:hsa-miR-8077
AGCUGAGUGUAGGAUGUUUACA
>Novel:hsa-miR-545-3p
UCAGCAAAACAGGCAAACGGAG
>Novel:hsa-miR-5195-5p
AACCCCUAUCACGAUUAGCAUUAA
>Novel:hsa-miR-4267
CCCAGCUCACCAGAGCCCACCCUGA
>Novel:hsa-miR-4721
AGAGGGCUGAGGACCAGGG
>Novel:hsa-miR-3126-5p
AGAGGGACCUUGGUCAGGGGACA
>Novel:chi-miR-323b
AACAAUACAACUUACUACCUCA
>Novel:hsa-miR-1252-5p
AGAAGGAAUCUGGGCAGGC
>Novel:hsa-miR-148b-5p
AAGUUCUGCAGUGGAUGGGCA
>Novel:hsa-miR-4668-3p
GAAAAUCCCGAACAAACUUUCA
>Novel:hsa-miR-6886-3p
GGCCCUUCAGCGUGUCUUU
>Novel:chi-miR-485-5p
AGAGGCUGUGAGGUUCUGUCCU
>Novel:chi-miR-9-5p
CCUUUGGUUUGGACUUAGGG
>Novel:hsa-miR-12117
AAAGUGGACUGCGGCGAGCCG
>Novel:hsa-miR-4665-3p
UUCGGCCGACAGCGGGGC
>Novel:hsa-miR-7160-3p
AAGGGCCCUGACUCAGAG
>Novel:hsa-miR-6780a-3p
GUCCUCUGCCAGCCUCGGCA
>Novel:hsa-miR-4689
AUGAGGAGGACGUAGGAG
>Novel:NC_037554.1_25895
AGAAGAGGAGGAAGAGCU
>Novel:hsa-miR-6839-3p
UGGGUUUUUGGAGCAGGGAG

>Novel:hsa-miR-8070
UUGUGAUUAAAGGAUCAGAGC
>Novel:hsa-miR-6779-3p
CAGCCCUGAGUGGGGCCAGUGG
>Novel:hsa-miR-3529-3p
AACAACAAAAUCACUAGUCUUCCA
>Novel:hsa-miR-4476
GAGGAAGGCAGGAGACAG
>Novel:hsa-miR-3918
UCAGGGCCUUGUCGUCUCUCCCAGG
>Novel:hsa-miR-1252-3p
UAAAUGAGAUGUUCUGGCUU
>Novel:hsa-miR-6892-3p
GCCCUCUCCAUCCGGCCCUCGA
>Novel:hsa-miR-4640-5p
UGGGCCAGUGGUGCUGUG
>Novel:chi-miR-450-3p
CUUGGGAAUUAGGACUUGGGACU
>Novel:hsa-miR-7162-3p
CCUGAGGUGCUGGGAGCUCC
>Novel:hsa-miR-3150b-3p
AGAGGAGAGAAGAGCUGGGU
>Novel:hsa-miR-4448
GGCUCCUUCUUCGCCCCU
>Novel:hsa-miR-4271
AGGGGAAGAGCCCAGGGC
>Novel:hsa-miR-1304-3p
GCUCACUGACAACCUCUCU
>Novel:hsa-miR-448
UUGCAUAUGGAGGACUGUCUGU
>Novel:hsa-miR-4687-3p
AGGCUGUUCAACUCCUUCUU
>Novel:NC_037568.1_45559
CCCGCCCGGCCCAGCCCG
>Novel:NC_037555.1_26459
UCUUCUUUGUUCUUCCUCCUAU
>Novel:NC_037559.1_34348
GGCGCGCGCGGCCCCCGCGG
>Novel:hsa-miR-3179
AGAAGGGGCUCCUGAGCACU
>Novel:NC_037561.1_36289
GUCCGGGGCUCCGCGCGCCUGGG
>Novel:NC_037569.1_47154
CCCGGGAGGGGGCGGGGC
>Novel:NC_037546.1_4552
UAACCGGCCACCGGCCUCGCGGCG
>Novel:NC_037555.1_27029
AAGGCUACCCGCUCUGGAAGGUA
>Novel:hsa-miR-3185
AGAAGAAGAAAGAUGCCGGAAAG
>Novel:hsa-miR-1324
ACAGACAGUCCUCCAUAUGCAA
>Novel:chi-miR-671-5p
AGGAAGCCAGUUGAAGAAG
>Novel:hsa-miR-185-3p
CGGGGCUGGGCGCGCGCU

>Novel:NC_037546.1_6618
CGCCGCGAGGCCGGUGGCCGGUUA
>Novel:NC_037551.1_20226
CGAGGAGCCGAGCCCUGCGCA
>Novel:NC_037566.1_43625
GCCGGGCCCGCCGCCGGG
>Novel:NC_037564.1_40975
GCCGCCGCCCGGGGCUGACU
>Novel:NC_037569.1_47143
UCGUGCAGAGAGGCGGGGCGA
>Novel:hsa-miR-6852-5p
ACCUGGGGAGUGCAGGGCCUGGCCU
>Novel:hsa-miR-3944-5p
GGUGCAGCCCAGAGCCUCU
>Novel:NC_037561.1_36492
CCAGGGCUCGCUGAGCCUCAUC
>Novel:NC_037556.1_30323
ACCCCAGCCCGGAGCCCACGGA
>Novel:hsa-miR-1908-3p
GCGGCCGCCCCCGGGGAG
>Novel:NC_037551.1_19470
UGCGCAGGGCUCGGCUCCUCG
>Novel:NC_037559.1_33762
CCGCGGGGCCGCGCGCGCC
>Novel:NC_037561.1_37162
CCAGGGCUCGCUGAGCCUCAUC
>Novel:hsa-miR-6817-3p
UCUCUCUGUUCCACCGCUCGA
>Novel:NC_037546.1_4218
GGCUGCAGCCGCCGGCUUUCG
>Novel:NC_037546.1_5605
CUGCCCGGAUCCCAGUAGCCA
>Novel:hsa-miR-4446-3p
AAGGGCUGCAGGGAGGGG
>Novel:NC_037562.1_38088
ACUGGACCACCAGCGAAUUCUC
>Novel:NC_037545.1_2589
GGGCGGGCGGGGCCGGGGC
>Novel:NC_037545.1_1635
GCCCGCCUCCGCCCGGAGU
>Novel:hsa-miR-7110-3p
GCUCUCUCAGCGUCUCGGAGA
>Novel:NC_037559.1_34015
ACCCUCACACCUGCUGCCCGCUCAC
>Novel:NC_037566.1_43137
CCCGGCGGCGGGCCCGGC
>Novel:hsa-miR-3124-3p
ACUUUCCUAGAUAUUUCCCACU
>Novel:hsa-miR-4700-3p
GACAGGACAGGGCAUGGGGG
>Novel:NC_037553.1_23622
UCAGGGAUGGGUGUCGGAGGAG
>Novel:NC_037567.1_44553
CCAGUUAGGUGACUCAGAGUAA
>Novel:NC_037547.1_9757
GGCCGUGACUGGAGACUGUUA

>Novel:NC_037556.1_28345
AGGAUGCUCUGGCUGCCU
>Novel:NC_037564.1_40897
CUGCCAGAGGCGUCGGGCCGC
>Novel:hsa-miR-658
CGCGGAGGGAGAACCCGGCCCU
>Novel:NC_037566.1_43159
CCGGAGGCAGCGUGCUCGUU
>Novel:NC_037549.1_17067
CUCUCCCCGGGGCUCCCG
>Novel:NC_037549.1_15945
UUCGGGUCCACAGAGCUCUCCGC
>Novel:NC_037545.1_698
CACCUGUGAGCUGAAACGCCCG
>Novel:NC_037548.1_12165
UGGGUCGCGACCUCUCCCCGG
>Novel:chi-let-7i-3p
CUGCGCAAAGCGUCGGGCCCCGG
>Novel:NC_037550.1_18158
GCCCCGCCCCCUCGCGGG
>Novel:NC_037565.1_42675
GCCCCCCGUCCUCCUCCU
>Novel:NC_037549.1_15024
GAAGUCCCUGAACACUCUUAUA
>Novel:NC_037555.1_26499
CACCCCAGGUCAGAAAGAGC
>Novel:NC_037549.1_16548
UAUAAGAGUGUUCAGGGACUUC
>Novel:NC_037561.1_36265
CCCCGGGGCUCCCGCCGG
>Novel:NC_037553.1_23471
AGCCGCGAGCUGGAACCCGAG
>Novel:oar-miR-1197-5p
GCCUUCCUCCCAGGGCCCU
>Novel:NC_037568.1_45446
AGCCCGAGUCGCCCGGGUCUCU
>Novel:hsa-miR-583
GAAAGAGGAUCCUUAGGAG
>Novel:hsa-miR-3605-3p
ACUCCGUGGAAGCUGUCCUC
>Novel:NC_037565.1_42436
CGGCCUGAUUCACAACACCAGCU
>Novel:hsa-miR-4683
GGGAGAUCCUCAGGAAAAG
>Novel:NC_037547.1_10895
CAGAGCCCAGGUCCCCGGCUCU
>Novel:hsa-miR-3180-3p
AGGGGCGGCGGCGGCGGGG
>Novel:NC_037561.1_36258
AGGCCUCAGACCGAGCGU
>Novel:NC_037549.1_14540
GAACCCCGCCAAUCCUCCGGUAGG
>Novel:NC_037547.1_10896
CAGAGCCCAGGUCCCCGGCUCU
>Novel:NC_037567.1_44107
UUACUCUGAGUCACCUAACUGG

>Novel:NC_037546.1_4225
GGGCCUCCCACUUAUUCU
>Novel:hsa-miR-5586-3p
GAGAGUGAGAGAUGCCAGUCCCGAG
>Novel:NC_037566.1_43651
AACGAGCACGCUGCCUCCGG
>Novel:NC_037569.1_48203
UGAGGGUCAAGGGUCAGAGAGCU
>Novel:NC_037560.1_35659
GCUGGAGGAAGGGCCCAGAGG
>Novel:NC_037545.1_137
GGCGGGGGGCGGAGCGGG
>Novel:hsa-miR-572
GUCCGCUCGCAUCCGCCCU
>Novel:NC_037546.1_6567
UCCCGGGGGCCCAGGUUCUCC
>Novel:NC_037553.1_23515
CCGGCCAUCCUCCAGCCCCCA
>Novel:NC_037552.1_21136
UCAAUGGAUACGAAUCUGGGCU
>Novel:NC_037566.1_43276
GCUCCGAGUCCUCCGCUGCAGC
>Novel:NC_037546.1_5469
AGGGUCAGAGUCAAGGGAGG
>Novel:hsa-miR-1293
UGGGUGGUAAUAGAAUGGGCUCCA
>Novel:NC_037567.1_44729
UGGAUGCGUUCUUGCCACUGC
>Novel:NC_037558.1_31787
AAACACACCUUCCCUGCUCUGC
>Novel:NC_037558.1_32253
GGAGGAAGUGCGGCUGGACC
>Novel:NC_037568.1_44803
GGAAGCCGGCGGGAGCCU
>Novel:NC_037565.1_41731
UCGGAGCCUGGAGCCUGGA
>Novel:NC_037556.1_29371
AGGGUGAGGAAGGCAGGAG
>Novel:NC_037547.1_8540
UCGCCCUCUCAACCCAGCUUUU
>Novel:hsa-miR-7108-3p
GCCCGCCCGGCUCCCCGG
>Novel:hsa-miR-4783-5p
GGCGCGCCCCCGCCGCGC
>Novel:NC_037558.1_32412
CCAGAAAGUUCAUUCAGUUUUU
>Novel:NC_037548.1_11146
UCCAGGAUUUUGGCUUAGGACU
>Novel:NC_037548.1_12742
UCCAGGAUUUUGGCUUAGGACU
>Novel:NC_037546.1_6394
CCUGGGACUGUGGGCGCCCCCU
>Novel:hsa-miR-4515
AGGACUGGAGAGCGCCCGCGGGUC
>Novel:NC_037568.1_45672
ACGCUGCAAAGCCAGGCCUUCCA

>Novel:NC_037561.1_36931
GCCCUUUCACCAUUGCACUG
>Novel:NC_037569.1_46587
CUUCUCCUCCUGCCCCUCCAGA
>Novel:NC_037550.1_18976
GUUGGCCAAAAAGUUCAUUCAGG
>Novel:hsa-miR-4711-5p
GGCAUCAGUCCACUGGCAC
>Novel:NC_037568.1_45368
ACUCGCCGUCGGAGGGGAGGCU
>Novel:hsa-miR-6784-5p
CCCGGGGCUCAGCGCGCCCGCG
>Novel:NC_037564.1_40367
UGUGAGGGGCCUCGGCCUACUGGU
>Novel:NC_037548.1_12436
AGGUUCCAGGCGUUUGGCUGAG
>Novel:hsa-miR-6827-5p
AGGGAGCCAGUUUAUGCCGACAGAA
>Novel:NC_037552.1_21299
AACGGACGAGUGUUGAUGCGAU
>Novel:NC_037547.1_7786
GUCGAGAGCUUGGUCCAGACCG
>Novel:hsa-miR-744-5p
GGCGGGGCGGCGGCGGGC
>Novel:hsa-miR-1203
UCCGGAGCCUAGGGCAAGAUU
>Novel:hsa-miR-3960
GGCGGCGGUCGCUUCGGCUCGC
>Novel:NC_037554.1_25410
CCAAAAGUUCACUCAAGUUUUU
>Novel:NC_037558.1_33125
GGUCCAGCCGCACUUCCUCC
>Novel:NC_037558.1_32071
UCCCCUCUCCCAGUCCCUCAC
>Novel:NC_037558.1_33041
GAGGGGAGAGCACCGAGG
>Novel:hsa-miR-6781-5p
CGGGCCGGUAACGCCGGGCACU
>Novel:NC_037567.1_44155
AGACAGUUCUUCUCCUUCU
>Novel:NC_037562.1_39076
UUCUCAGCCCCAGGGGUUC
>Novel:NC_037568.1_46017
GCUGGAGGAAGGGCCCAGAGG
>Novel:NC_037548.1_11079
AGGAAGUGGACGGAGGAGGC
>Novel:NC_037557.1_30447
GCGCUCCAAGUCCCGGGAAAGU
>Novel:NC_037556.1_28346
AGGAUGCUCUGGCUGCCU
>Novel:hsa-miR-6849-3p
CCCAGCCUCCACUCCGGCUCGCC
>Novel:NC_037560.1_35933
CUUUCCGGCAUCUUUCUUCUUCU
>Novel:hsa-miR-3181
CUCGGGCCUUAGAGCUCUUGG

>Novel:NC_037552.1_21749
ACCCUCACCUGCUUUCCCCAG
>Novel:NC_037546.1_2774
ACGCAGCGGCCCGCUCUCUGA
>Novel:NC_037561.1_36929
GCUUUGACAAUAUCAUUGCACU
>Novel:NC_037564.1_40877
GCUUCUCCCCAUCCUCACUGAA
>Novel:chi-miR-491-5p
UGUGGGGAGGAUGGACCCUGGCU
>Novel:NC_037545.1_583
UAUUAAUACACAUUGGAUUUCU
>Novel:NC_037546.1_3432
CUCGGGGACUGGGCCAGGCU
>Novel:hsa-miR-548u
AAAAGACUGGCUAGAAAG
>Novel:hsa-miR-6771-5p
UUCGGGAGGCUAGGGACGGGCU
>Novel:NC_037555.1_27170
AGGCAGAGCAGGGGCUUUUCCU
>Novel:NC_037545.1_2765
UGCCAGAGGCCCAGGUCCCUCU
>Novel:hsa-miR-4467
CGGCGGCGGCGGCGACUC
>Novel:NC_037552.1_22028
AGCCCAGAUUCGUAUCCAUUGA
>Novel:NC_037552.1_22617
CUUCCGAUGCGUCUCCUCU
>Novel:hsa-miR-6727-5p
CUCGGGGCAGCUCAGUACAGGA
>Novel:NC_037563.1_39275
CAUGGUUAGAUCAAGCACAA
>Novel:NC_037549.1_15915
GUCAUCAUUACCAGGCAGUAUUA
>Novel:NC_037568.1_44799
GGGCCUCGGUCCUACAGCCGAAGG
>Novel:NC_037548.1_11583
UUCCCUGGCAGUCCAGUGGUUAA
>Novel:hsa-miR-6085
UAGGGGCUGUCACAUAUGGGC
>Novel:NC_037547.1_9583
AGAAGAAAGAGCGUGCUGGGG
>Novel:NC_037557.1_31142
AAAAACCUGAACGAACUCUUU
>Novel:hsa-miR-3144-5p
UGGGGACCCAGGGAGCCGGGGG
>Novel:hsa-miR-943
UUGACUGUCAGGAGCCCCUCACA
>Novel:hsa-miR-3908
GAGCAAUGAUGAAAAGGU
>Novel:NC_037569.1_47983
AAAGAGUUCGUUCAGGUUUUU
>Novel:NC_037562.1_38773
UGUCCGUGGUUCUACCCUGUGGU
>Novel:hsa-miR-185-5p
AGGAGAGACCGGACGGAGC

>Novel:NC_037564.1_41171
GGCCCAGGAUGAGAACCC
>Novel:NC_037545.1_1965
AGAAAUCCAAUGUGUAUUAAUA
>Novel:hsa-miR-10392-3p
GCGGCCCCCGCCUGCUCGGCGG
>Novel:NC_037564.1_40336
AGAAGUGCACCAUGUUUGUUU
>Novel:hsa-miR-6763-3p
GUCCCCGGGUCUCCCGCCG
>Novel:NC_037552.1_21735
CAGGCUGUGCUGGCUGGAGCU
>Novel:NC_037545.1_315
GCUGCCUGUGAAUGGGCUG
>Novel:NC_037545.1_824
CCAGGAGCUCUCAAUCUAGUG
>Novel:NC_037546.1_2911
AAACCAGCUCUACCUCUGUCUU
>Novel:NC_037547.1_7477
UGAGCGGGGCUGUGGGCUGGCAGG
>Novel:NC_037560.1_34765
GCAAGCCCAGACCGCAAAAAG
>Novel:NC_037557.1_30978
AAAACGUUCGUUUGGGUUUUU
>Novel:NC_037553.1_24652
UCUGAGUGGCCCACGGCUCCU
>Novel:NC_037547.1_9951
GCUUUGACAAUACUAUUGCACUG
>Novel:NC_037551.1_19861
GAACAAGCUGUAUAUUUGAGC
>Novel:NC_037548.1_13083
AGAGGAGUAGGGCAUGGGAC
>Novel:hsa-miR-4479
UGCGCGGCUCUCUGCUCAGG
>Novel:NC_037556.1_29821
AGCCCCAGCCACAGCCGCAGA
>Novel:NC_037561.1_36340
GUCCCUCCUGCAGGUCCAGUU
>Novel:NC_037555.1_28140
AAGGAAAAGCCCCUGCUCUGCC
>Novel:NC_037547.1_10096
GCCUUUCUCUCCUCCUUU
>Novel:hsa-miR-1184
GCUGCAGCGGAGGACUCGGAGC
>Novel:NC_037547.1_10102
ACAAUCUUCCAUGUUCCUAU
>Novel:NC_037548.1_14012
AAACCCAAACGAACUUUC
>Novel:NC_037565.1_42179
UUGGUGAUGGACAGGGAGGCC
>Novel:chi-miR-345-5p
UCUGACUCCUCCCUCCCU
>Novel:NC_037552.1_21699
UCGGGACCCGCUCUGCCGGAGA
>Novel:NC_037547.1_7908
AGUCCUCAUCAACUUAAGAAAU

>Novel:NC_037547.1_10537
AAAGAGUUCAUUCAGGUUUUU
>Novel:NC_037569.1_47783
UUCAGUACUUGCAGUUCAUGGACU
>Novel:hsa-miR-1249-5p
UGGAGGGACUGUGGCAGCGAGC
>Novel:hsa-miR-570-3p
GGAAAACACAGUCACCUC
>Novel:NC_037549.1_15077
AAAACCCGAACGAACUUUUC
>Novel:NC_037547.1_7667
AGCCUCGGCUCUCAGGCCCAUA
>Novel:NC_037558.1_32026
GGAAAGCCUCCGCCCAACCCU
>Novel:NC_037547.1_7828
AUGGGGUAAGGAUGGAGAGGACC
>Novel:NC_037555.1_27093
GAACCUCAGACCUUCUUCUCU
>Novel:NC_037545.1_1896
UACGGGAGAGUGAGGACUGU
>Novel:NC_037569.1_46554
UGAAUCCUUGAAUAGGUGUGUU
>Novel:NC_037545.1_498
GUCCCAAGAGCCUGACUUGU
>Novel:hsa-miR-8057
UUGGCUCUGCGAGGUCGGCU
>Novel:NC_037564.1_40376
CCUCCAGACCCCUAGUUCAGG
>Novel:NC_037569.1_46552
AGAAUCCUUGCCCAGGUGCAU
>Novel:hsa-miR-4767
UGCGGGCGGACGAGGGGC
>Novel:NC_037546.1_3187
GGCCAGAGCUGAGGUUCU
>Novel:NC_037550.1_19237
UGCAGCGCCCACUGUGGAAG
>Novel:hsa-miR-2355-5p
GUCCCCAGCCCGUCCACCCAG
>Novel:NC_037556.1_30288
CGCGUACCAAAAGUAAUAAUG
>Novel:NC_037552.1_21521
AGUCUGUCCUCAUUCUUCU
>Novel:NC_037562.1_38928
CACCCCCACUUCUCCUUU
>Novel:NC_037553.1_24219
GUCCUGGCUCCCCUCUUCCC
>Novel:NC_037546.1_4148
GGAAGAGGACAGUCCAUGGGCA
>Novel:NC_037558.1_32366
AAAGACGCAGGAGGCCAAGA
>Novel:hsa-miR-5580-5p
GGCUGGCUCCCGCGGCCCC
>Novel:NC_037569.1_46557
ACCCUCCACCAUGCAAGGGAUG
>Novel:hsa-miR-2276-5p
UCCCUCUGGGUCUGUGGGGA

>Novel:NC_037547.1_9251
UCCAGCCCCUUCUCCUUC
>Novel:NC_037549.1_14559
AAAGACUGGGAGCAGGGGAAG
>Novel:NC_037566.1_43265
AAAAGCCUGAAUGAACUCUUU
>Novel:NC_037564.1_40744
AAAGAGUUCGUUCAGGUUUUU
>Novel:hsa-miR-6068
CCUGCGAGGGGGCGGGGC
>Novel:NC_037555.1_28194
ACAGAAAAACCCGAACGAAUU
>Novel:NC_037549.1_14328
AGUUUUACUACACUCCUUCU
>Novel:NC_037547.1_6928
CCAAGGGAAGUCGGGCCUUUGC
>Novel:NC_037564.1_40741
UCUGGAGAAGAGUCCAGCCCUUU
>Novel:NC_037560.1_35724
UCUGUCCCUGGAGAGCAUCCUCU
>Novel:hsa-miR-6747-3p
UCCUGCCUUCCUCACCCUC
>Novel:NC_037551.1_20840
GACUCUCAGUCCCUUCUCCU
>Novel:NC_037564.1_40932
GUCCUCCUACUCCUCUGCUAU
>Novel:NC_037553.1_23542
AGGAGCCGUGGGCCACUCAGA
>Novel:NC_037547.1_9916
AGUCCUCAUCAACUUAAGAAAU
>Novel:NC_037568.1_45118
AUGGCAACUGUAACGGCGC
>Novel:NC_037569.1_46660
AGAAGCAGCUGCAGAAGGAC
>Novel:NC_037560.1_35631
AAAGAGAGGCUUUGAACCUGGGAC
>Novel:NC_037564.1_40236
GUGGUCCUAAACAUUUCAC
>Novel:NC_037547.1_9353
GCUCCUGGAGGACAGGGA
>Novel:NC_037553.1_24557
AGCAUAGGUGGGGCUGGGACU
>Novel:hsa-miR-6513-5p
UUUGGGAUAAGGGAAGGAC
>Novel:NC_037565.1_42130
AGGAGAAAGCUGUGCACU
>Novel:NC_037546.1_4816
AUCUGCAGUUUGACGGGCU
>Novel:NC_037545.1_1554
GGUGCAGGACGGAGGAGGU
>Novel:hsa-miR-4537
CGAGCCGAAGCGACCGCCGCC
>Novel:NC_037547.1_7372
AAGAAGUUCGUUUGGGUUUUU
>Novel:hsa-miR-6846-5p
UGGGGGCUGGAGGAUGGCCG

>Novel:NC_037546.1_4402
ACUUUUGUGACUAUGCAA
>Novel:NC_037550.1_17666
CCAAAAAGUUCAUUCCAGUUUU
>Novel:NC_037550.1_17420
AUUGUUGUCGAGGCGGCCGG
>Novel:hsa-miR-4450
GGGGGAUUAGCUCAGCUG
>Novel:NC_037569.1_47073
CUACCUGCACUGUAAGCACUUU
>Novel:NC_037551.1_20645
AUCUAGGAGAUCCGGCUGAGG
>Novel:NC_037552.1_22370
GCAAGCCCAGACCGCAAAAAG
>Novel:NC_037564.1_41393
UGGCCAAGUGGACUCUUCUCCU
>Novel:NC_037546.1_3818
AGUUCUGACUCCAAGUCCAGG
>Novel:NC_037553.1_23265
UGAGUACUUACUAGGUGCCAAG
>Novel:NC_037560.1_35884
ACAAGUUCGGAUCUACGGGUU
>Novel:hsa-miR-663a
AGGCGGGGGACGGAGGAGGC
>Novel:hsa-miR-4800-5p
UGUGGACCCAGGGAGCCGGGAGG
>Novel:hsa-miR-5739
GCGGAGAGCUCUGUGGACCCGA
>Novel:NC_037569.1_48052
AUCUGAAAAACUUAUUGAAAC
>Novel:NC_037547.1_9101
GCAGCCAAGCCUCCUAACAGUUG
>Novel:NC_037564.1_40695
GGGACAGGGUCGGGGGAC
>Novel:hsa-miR-4428
AAAGGAGAGGAGGAAGAGGCU
>Novel:NC_037569.1_46549
CAGCUCCGAUAUGCAAUGGGUA
>Novel:NC_037546.1_3986
AGGACACAAGUUGGGAAAGCAGCC
>Novel:NC_037547.1_9992
AGUGCUUUCUACUUUAUGGG
>Novel:NC_037552.1_21762
CUCUCCACAGCCUCCUCU
>Novel:NC_037558.1_32847
GCUGGGCCCAGAGUCCAUGUU
>Novel:NC_037564.1_40946
AGAGCCACUCCAACAUCUGU
>Novel:NC_037558.1_31842
AGGCCAGGGCCUUCUCCU
>Novel:NC_037564.1_40778
GAAAGUUCGUUUGGGUUUUUC
>Novel:NC_037564.1_40307
ACUUUCCAACUGGUCGACCA
>Novel:NC_037547.1_10878
GGUUCCUUUUUUCCUGAG

>Novel:NC_037561.1_37123
AAACAGCCCGAGUCUCUCGA
>Novel:NC_037553.1_24410
UUUGGUGCGUUGGCCGGGAA
>Novel:NC_037552.1_22436
AGAGUUUCCUCUCCCGGUCCAAA
>Novel:NC_037560.1_35256
GAUGGCAGUGGAACUAGUGAU
>Novel:hsa-miR-6515-3p
UCUCUUCAGCUCUUCCCU
>Novel:NC_037569.1_47107
AGUCAAAUCAAUUUUUUACCUCU
>Novel:NC_037552.1_21636
CUGGUCCUCAGCUUCUCUC
>Novel:NC_037548.1_11963
AGUCCUCCACCAGCCCCUGCA
>Novel:NC_037560.1_34839
AAUCGCUGGGUUGAGAGG
>Novel:NC_037564.1_40358
AGACUCAUUUGAGACGAUGAUG
>Novel:NC_037547.1_8172
CUUCUGUGUUCUCUUCCU
>Novel:NC_037551.1_19485
UCUCGCUCUCUGCCUCUCU
>Novel:hsa-miR-4723-5p
AGGGGGAGGACGGAGGAGG
>Novel:NC_037562.1_38494
UGGUGCGUUGGCCGGGAAACG
>Novel:NC_037564.1_40345
CGUACGCUAUACGGUCUACUAU
>Novel:NC_037557.1_30428
AAAGAGUUCGUUCAGGUUUUU
>Novel:NC_037548.1_12074
GCCACUAGACCACCAGGGA
>Novel:hsa-miR-6778-3p
AGCCUCCCAUCCAGCGGCUG
>Novel:NC_037547.1_10839
AGCACCCACAGCUUCUCCU
>Novel:NC_037557.1_31269
CAAAAAAUUUCAUUUGGGCUU
>Novel:hsa-miR-6784-3p
CCUCACCCCGCCCGCCCG
>Novel:NC_037563.1_40142
AAGAACCUGAACGAACUUU
>Novel:hsa-miR-6888-5p
GAGGAGAUUGAGCUGGGCGAG
>Novel:NC_037548.1_13020
GAUCCGAACGAACUUUUUGGCCAA
>Novel:NC_037552.1_21563
UUUGGACCGGGAGAGGAAACUCU
>Novel:hsa-miR-4733-5p
GAUCCCAAGGCCGAGUUCUCCUCCU
>Novel:NC_037564.1_41452
AAAAACCUGAACGAACUCUUU
>Novel:NC_037553.1_24374
GUCCAUGGGGUCACAAAGAGUCA

>Novel:NC_037546.1_6204
UACAACAGGGACUGGAGGGCCU
>Novel:NC_037569.1_47131
AACGGAACCACUAGUGACUUG
>Novel:NC_037545.1_1001
CGCCAAUAUUUACGUGCUGCUA
>Novel:NC_037551.1_20679
AGGAAAGGGCUGGGGAGU
>Novel:NC_037560.1_35881
AAAAGUUGGUUUGAGUUUUUC
>Novel:NC_037565.1_41728
UUUUCUCAGUUACUGUGAACU
>Novel:NC_037566.1_42927
AAACCCAAACGAACUUUU
>Novel:NC_037569.1_47135
AUGCUCAGACAACCAUGGUGCA
>Novel:oar-miR-299-3p
CAUGUGGGCACUGAACGCUC
>Novel:NC_037564.1_40326
ACAGAGAGCUUGCCCUUGUAUA
>Novel:NC_037546.1_4997
AAGACAGAGGUAGAGCUGGUUU
>Novel:NC_037557.1_30641
CUACCUGCACUAUAAGCACUUUA
>Novel:NC_037553.1_22994
GAGCUACAGUGCUUCAUCUCA
>Novel:hsa-miR-149-5p
GCUGGCUCCCUCGGCCCA
>Novel:hsa-miR-146b-3p
UCCCUGUGGUCUAGUGUU
>Novel:NC_037545.1_431
AAAACCCAAACGAACUUUUC
>Novel:NC_037566.1_42967
CCUGAGGGAUCUUUAGUUGCUG
>Novel:hsa-miR-3917
CCUCGGACUGGAGGACUGUGGCCG
>Novel:NC_037564.1_41155
GGGCAUCACAGACCUGUU
>Novel:NC_037565.1_42630
AGGCCCAGGCCUCAGUCCUC
>Novel:hsa-miR-5587-3p
GCCCCGGGGGCACCCGGCU
>Novel:NC_037569.1_46468
GAGACCCAGUAGCCAGAUGUAGCU
>Novel:hsa-miR-1301-3p
UUGCAGCUGGUGGAGUUCUCGGC
>Novel:NC_037558.1_32842
CUUCUCCAGGGGAUCUUC
>Novel:NC_037546.1_6872
AGGCCCAGAUUCCACCUCU
>Novel:NC_037555.1_26737
CGCAGGCGGCGGGAGCGGGAC
>Novel:NC_037556.1_28514
AGGUUGGAGGUGUGGGCUAGAGUA
>Novel:NC_037555.1_27452
UUUGGAAUUGGUACUGGGACG

>Novel:hsa-miR-1915-3p
CCCCAGGGCCUUUCCUUCU
>Novel:NC_037561.1_36813
CUUUUGCCCUUCUGCUCC
>Novel:NC_037546.1_3761
CUAGUUUCUUUUCCUCCG
>Novel:NC_037554.1_24998
GGGCUACAGUCCUUGGGU
>Novel:NC_037545.1_1689
CAGCCCAUUCACAGGCAGC
>Novel:NC_037565.1_42629
AGGCCCAGGCCUCAGUCCUC
>Novel:NC_037545.1_558
CAGAGUACUAACCACUAAAA
>Novel:NC_037547.1_9455
AGAGGAAGAGAGAAGAGC
>Novel:hsa-miR-6801-5p
AGGUCAGAGUGUGGAACCGGGA
>Novel:NC_037553.1_22953
UCUAGGAGCUCACAGUCUGGU
>Novel:hsa-miR-6089
AGAGGCCGACCGUGUAUUGUG
>Novel:NC_037554.1_25983
ACAGGACUUGGAGUCAGAC
>Novel:NC_037545.1_1362
UUCUCCCGGGAGCUCGGCCUG
>Novel:NC_037548.1_11601
CUCAUCACACUGUUUCUCUCUA
>Novel:NC_037558.1_33240
UGGAGAGCUGGAGGGGCGGCCA
>Novel:hsa-miR-4706
UGCGGGGAGUGCACGGCCUGC
>Novel:NC_037557.1_31699
CCACUACACUAAGGAAAC
>Novel:hsa-miR-635
CCUUGGGCUGGCCUCUGACCUCGG
>Novel:NC_037561.1_36598
AAGGACCUGGAGAAAGCC
>Novel:NC_037568.1_44853
UGCGACCGGACAGGUCAGGGC
>Novel:NC_037569.1_47030
GUUUCAAUAAGUUUUUCAGAU
>Novel:NC_037548.1_13192
CUCAUCACACUGUUUCUCUCUA
>Novel:NC_037550.1_19148
GGGCAGCACCCCAGUCCA
>Novel:NC_037554.1_25287
AAAACCCAAACGAACUUUU
>Novel:hsa-miR-3125
GAGAGGAAGCGGGAGAGGAGC
>Novel:NC_037558.1_32697
CUUGCCUGUGGUCUCAUC
>Novel:hsa-miR-1199-5p
UCUGAGCCCAGGGCCCUCCCCCU
>Novel:hsa-miR-4277
ACAGUUCUCCAACUGGGA

>Novel:NC_037558.1_33211
AAUGAACUUUUUGGCCAACCC
>Novel:hsa-miR-6124
AGGAAAAGAUUUCGGACU
>Novel:hsa-miR-5589-5p
AGCUGGGUCUGAGGCCCCU
>Novel:NC_037546.1_2906
UCCUCCAGAGGUUUCCUUCU
>Novel:NC_037547.1_8774
UUCUGAACCUGCGGUUCCU
>Novel:NC_037558.1_32244
CCAAAAGUUCACUCAAGUUUUU
>Novel:hsa-miR-1205
ACUGCAGGUCUUCAGUCAGGGCCAU
>Novel:hsa-miR-3683
CGCGACAUGAUCAUUCUGCCCG
>Novel:NC_037549.1_14335
GUCCCGGGGCCCCUCCUC
>Novel:chi-miR-103-3p
AGCAGCAUUGUCCAGGGC
>Novel:chi-miR-493-3p
GGAAGGUCACAGCCCUGGGGA
>Novel:NC_037561.1_37534
AGGAGUUGGAAUAGGAGCU
>Novel:chi-miR-449b-3p
CGCCACAAUUGCAGGACACUG
>Novel:NC_037546.1_4856
AGAAAGACUUGGACAGCU
>Novel:hsa-miR-4761-5p
UCAAGGUGGACUCUGCCC
>Novel:NC_037550.1_19123
AAAAGUUCAUUCCAGUUUU
>Novel:hsa-miR-339-5p
UCCCUGUCCUCCAGGAGC
>Novel:NC_037547.1_7586
AGAAAGGAAGGAAUCUGGGC
>Novel:NC_037563.1_39389
AAAGCAAUCUUUCAAAAUCCUA
>Novel:NC_037569.1_46466
AAACCCAGCAGACAAUGUAGCU
>Novel:hsa-miR-219a-2-3p
GGAAUUGUACAGUCCAUGGGGUUGG
>Novel:hsa-miR-4475
AAAGGGACAGUCGGAAGAA
>Novel:NC_037549.1_16477
GCAGAAGGGCAAAAGCUC
>Novel:hsa-miR-4276
UUCAGUGAGGAUGGGGAGAAGC
>Novel:NC_037569.1_46653
AUCCAAUAAACAUUUACUGA
>Novel:chi-miR-125a-3p
UCAGGUGAAGCACUGUAG
>Novel:NC_037546.1_4613
ACACCCGAACGAACUUUUUGG
>Novel:NC_037564.1_40415
GCUCUCUUGAGUUCCUUCAUCUU

>Novel:NC_037550.1_18169
AAAGGAAGCCUGGGGAGCCC
>Novel:NC_037546.1_5554
AAAACCCGAACAAACUUUUU
>Novel:hsa-miR-4743-5p
AGGCCGGAUUCUGUGCCCACCA
>Novel:hsa-miR-1265
GAGGAUGUAGCUCAGUGG
>Novel:hsa-miR-3157-5p
UUCAGCCAUGGACUGCAGGGC
>Novel:chi-miR-378-3p
ACUGGACUUGGGGUCAGAAGGC
>Novel:NC_037569.1_47935
AGAGGAAACCAGCAAGUGUUGA
>Novel:NC_037569.1_46177
UGUACGGCAUUUAGGGGCAGAACU
>Novel:hsa-miR-1289
CGGAGUCCGGCUCUCGGCU
>Novel:NC_037569.1_47148
AACGCGCCGCUGGGCUGCUAGGCU
>Novel:NC_037547.1_8396
AGACGGAGGACUGAGGAG
>Novel:NC_037565.1_42321
UUCGGCCUUCAAGGUUCC
>Novel:NC_037548.1_11059
AGAGCCAGGCUCCCCAGUCA
>Novel:NC_037558.1_32782
CUUAUGGAUAAGGCACUG
>Novel:hsa-miR-4687-5p
GAGCCCUCUGAGCUUCAG
>Novel:NC_037548.1_12213
AAAAUGUUCGUUUGGGUUUUU
>Novel:NC_037548.1_11028
AGAAGGAGGACAUUAAGGCC
>Novel:NC_037562.1_38320
UAGGGGGGACCCUGGAUCCU
>Novel:oar-miR-3957-3p
GCGCACAGCCUCCUCUUCCU
>Novel:NC_037545.1_503
ACAAGAUCGGAUCUACGGGUU
>Novel:NC_037565.1_42764
GGCCAGCAGAGCCCAGCUGCCAAA
>Novel:NC_037564.1_40258
CCAUGGGGUGGAGGACUGC
>Novel:NC_037545.1_2499
GCAUUGGUGGUACAGUGG
>Novel:NC_037547.1_8180
GCCAGUCAUUCUCUCUCUC
>Novel:NC_037557.1_31285
UUUUCAUUAAUAGUUUUGGGAGC
>Novel:NC_037548.1_13397
AAAACGUUCGUUCGGGUUUUU
>Novel:NC_037550.1_18832
UGGUGCAUUGGCCGGGAA
>Novel:NC_037551.1_19555
AAGGUUCUUUGUCCUGCUCUGA

>Novel:NC_037553.1_24444
AGGAAGAUGAGCUGGACA
>Novel:NC_037551.1_19947
CAUGGUUAGAUCAAGCACAA
>Novel:NC_037549.1_14837
GUAUUGAGUUGGCCAAAAAA
>Novel:NC_037546.1_4132
AGGCCCUCCAGUCCCUGUUGUA
>Novel:hsa-miR-6514-3p
CUGCCUGUCUGUGCCUGCUG
>Novel:hsa-miR-4667-5p
UCUGGGGAGGGGUGGAAGGAU
>Novel:NC_037549.1_14266
AGGCGCUUUCGGUUGGGC
>Novel:hsa-miR-6748-3p
UCCUGUCCUGCAGUCGGAAUCU
>Novel:hsa-miR-6885-3p
CUUUGCUUUCUGAUUGUAGGGCCC
>Novel:NC_037558.1_32528
CAGUACCCCUGACUUCUCAGAGG
>Novel:hsa-miR-3714
GAAGGCAGGCGGGGAAGC
>Novel:hsa-miR-3173-3p
AAAGGAGGAAGCUGAACUCAGG
>Novel:hsa-miR-152-5p
AGGUUCUGAGCCUCAGUCUCCU
>Novel:NC_037550.1_18353
AAAACUGGAAUGACCUUUUUGG
>Novel:hsa-miR-4524a-3p
AGAGACAGAUCCAAGGAC
>Novel:NC_037550.1_18908
AAGCCCAAAAGGAGAAUUCUUUG
>Novel:NC_037557.1_30571
AAGCCCAAAUGAAAUUUUUUG
>Novel:NC_037545.1_1619
UGCGGACCGGCUGCGUGGCGAGA
>Novel:NC_037550.1_18703
GAUUAGGUCUAGAAGAGAGGAUGA
>Novel:NC_037555.1_26610
UCGUCAUGACCCUUUCUCUCU
>Novel:NC_037562.1_38970
GUCCUUCCUUCUCUUCCCUC
>Novel:NC_037562.1_38317
GUCCUUCCUUCUCUUCCCUC
>Novel:NC_037557.1_30645
CCAGAAGGAGCACUUAGGGCAGU
>Novel:NC_037557.1_30644
CAGUUUUGCAUAGAUUUGCACA
>Novel:NC_037550.1_19285
UGGCUCCAGGCAGCCCGGCCCUUCU
>Novel:NC_037568.1_45170
AGUCUCGAGACCCUCUCUCU
>Novel:NC_037557.1_30640
CAGUUUUGCAUGGAUUUGCACA
>Novel:NC_037566.1_43556
AGUCCUCACUGUCCCCUU

>Novel:NC_037553.1_23290
UUCCCGGCCAACGCACCA
>Novel:hsa-miR-4765
AGAGUGAUCAGUUCCUGCAAU
>Novel:NC_037558.1_31855
UUCCGACGGGGCUAGAAACC
>Novel:NC_037546.1_4112
AAGAAGUUCGUUUGGGUUUUU
>Novel:NC_037547.1_8573
AAAACCCAAACGAACUUUUC
>Novel:NC_037555.1_28238
UCCAGCUGGUUGAACUUGAGACC
>Novel:NC_037564.1_40338
GAGGUUUCCCGUGUAUGUUUCA
>Novel:NC_037546.1_4370
AAGUCUUCAUCUUUGGCU
>Novel:NC_037557.1_30688
AGAAGAGAAGAGGACAUGGAAAG
>Novel:NC_037552.1_21756
UGCCCAUCCACUGCAGAACUU
>Novel:NC_037564.1_40573
AAGAGAUUGUACACAUGAAU
>Novel:NC_037547.1_10260
GGCUUCAGCUUCAGGUCUCU
>Novel:hsa-miR-3921
GCUCUGAGCCUCAGUUCCCUCCU
>Novel:NC_037549.1_15761
GUCUUCUCCGAAGCAACGAUU
>Novel:NC_037567.1_43945
GUCCCAGUGUGGCUGAUCAUG
>Novel:hsa-miR-6131
GGCUGGUCCAAUGGUAGUGGG
>Novel:hsa-miR-10398-5p
GGGCUCCCCAGGCUUCCU
>Novel:hsa-miR-6770-3p
UUGGCGGCGCGGCACGGCCUGG
>Novel:chi-miR-24-3p
AGGCUCAGCAUUUUCUUCCU
>Novel:NC_037547.1_7205
GCUCUCCGCUUCCCUCUCUC
>Novel:hsa-miR-6734-3p
CCCUUCCCCGCAGGCCUUC
>Novel:NC_037550.1_18131
AGAAGAGAGGAAGAGGGC
>Novel:chi-miR-7-5p
AGGAAGACGCGGUCGCGGCU
>Novel:NC_037564.1_40430
UGCUGCCAGGAGCUCAGGGCCU
>Novel:chi-miR-24-5p
AUGCCUACAGCACCCGGG
>Novel:NC_037547.1_8021
CCGUCUCCUCAGCUCCUU
>Novel:NC_037545.1_548
GAGUUCCUUCUCCAGAGU
>Novel:NC_037545.1_416
AGGGAUCUUUAGUUGCUG

>Novel:NC_037557.1_31280
AAUUCGUGGUUGGUUGAAUCU
>Novel:hsa-miR-4800-3p
AAUCCGUCGUCACCUGGUCACUGU
>Novel:hsa-miR-3689d
AGGAGGUGGACGGAGGAGA
>Novel:NC_037547.1_9224
AGAAAGAACUGCUCUGGCA
>Novel:NC_037554.1_25273
AAACCCAAACGUACUUUU
>Novel:NC_037562.1_38718
ACCCCAGAAUCCUUCUCU
>Novel:hsa-miR-3148
GGGAAAAAGGAACCAGGGCAGAA
>Novel:NC_037548.1_12850
CCGGAAAGCCUGCUAGACAAAU
>Novel:NC_037555.1_27478
GGAAGAGUGUGUCUGGGC
>Novel:hsa-miR-203a-5p
AGUGGUUCAGGUUGAUCUUCUU
>Novel:NC_037547.1_8898
AGGCCAGAGAUGCUUCCUU
>Novel:NC_037546.1_2960
UUCUAGCGACUGAAGGAAU
>Novel:NC_037553.1_23729
AGGCCUGGCUCUCUUCCCCAGA
>Novel:NC_037569.1_48122
AUGACAGAUUGACAUGAACAGUU
>Novel:NC_037547.1_8993
AGCACCUUCCCCCCAAUCCUGC
>Novel:hsa-miR-6890-3p
UCACUGCCUGGCUCCUCUUUCC
>Novel:NC_037553.1_23923
GCCAUUGAUGAUCGUUCUUCU
>Novel:hsa-miR-4740-3p
GCCCGAGACAACAAGAAGACG
>Novel:hsa-miR-3129-3p
AAACUAAUUUUUUGAGGCCU
>Novel:NC_037564.1_40729
AGACCUGUUCUAUCUACCUU
>Novel:NC_037568.1_45619
ACUCGCGGGGAUCUCGGGGCC
>Novel:NC_037555.1_26458
GCCCAGACACACUCUUCC
>Novel:NC_037562.1_38368
GCGGGGGGCCAGCUUCUCU
>Novel:NC_037565.1_41975
CUUACUUAUAGGGCUUUUGAUU
>Novel:chi-miR-221-5p
GCCUGGCAACGUUCUACUC
>Novel:NC_037558.1_33194
UCUUGGGACUGUCUGCCGAGCU
>Novel:hsa-miR-1251-3p
AGCUUUGCGCAGUGGCAG
>Novel:NC_037558.1_32328
AGCUCGGCAGACAGUCCCAAGA

>Novel:NC_037547.1_8236
UGAGAGUUGAAAGUUCAGA
>Novel:NC_037553.1_24363
CCUUCCAGGGAUCUUCUCCU
>Novel:NC_037553.1_23783
AAUCAGUCUCAUUGCUUUAUAA
>Novel:hsa-miR-7113-5p
CCCAGGGAGAUGCUGGACUGUGUGC
>Novel:NC_037561.1_36447
UCGAGAGACUCGGGCUGUUU
>Novel:chi-miR-22-5p
AGUUCUUCAGUGGCAAGC
>Novel:NC_037549.1_15080
UGGGCAGAGAGCACAGUCCAUG
>Novel:NC_037553.1_22911
AACCUAUGGAAUUCAGUUCUCA
>Novel:NC_037548.1_11085
AGAGAAAGGGCUCUGGAGC
>Novel:NC_037567.1_44437
AGGUACUCUGUGGAUAUCCCCUUGG
>Novel:chi-miR-151-5p
UCGAGGAGCUCACAGUCC
>Novel:hsa-miR-135a-3p
AAUAGGGAACGUGAGCUGA
>Novel:hsa-miR-4501
CAUGUGACUGAACUCUGGCUGG
>Novel:hsa-miR-4443
AUGGAGGCGUCCCUGCAGGAGC
>Novel:hsa-miR-6787-3p
GCUCAGCUCUCCAAGGUU
>Novel:hsa-miR-6818-3p
CUGUCUCUACAACUCUGCCACA
>Novel:hsa-miR-4318
CACUGUGGAGGAGGAGAUGUC
>Novel:NC_037565.1_42848
UCGUGGAAUGAGAAGCCGUGGC
>Novel:NC_037567.1_44092
ACCCAAUGAUGAAGACUU
>Novel:NC_037565.1_42322
UUCGGCCUUCAAGGUUCC
>Novel:hsa-miR-3153
AGGGAAAGCCGAUCCGGA
>Novel:NC_037549.1_16021
CCUCCUGACGACCUUCUCU
>Novel:hsa-miR-4308
GCCCUGGACAAGGCUGCU
>Novel:NC_037558.1_32790
GAAGAGAAGCUGUUGAGC
>Novel:chi-miR-23b-5p
UGGGUUCCUCCUUCUUCU
>Novel:NC_037562.1_38969
GUCCUUCCUUCUCUUCCCUC
>Novel:hsa-miR-6828-3p
CUCUGCUCUCCGACCCUG
>Novel:NC_037551.1_19577
CCUCUGUCAUUUUGUAAC

>Novel:NC_037556.1_28412
AUUGGUGGUUCAGUGGUAGAAUUCU
>Novel:NC_037562.1_38318
GUCCUUCCUUCUCUUCCCUC
>Novel:NC_037564.1_40393
AAGGCUCUGCAGAAAGGC
>Novel:hsa-miR-4468
GGAGCAGAAGGGCAAAAG
>Novel:NC_037548.1_11604
GCCUUCAGGACCUCAGAC
>Novel:chi-miR-1197-3p
GAGGACACGGUGGCCAUGAUGGG
>Novel:hsa-miR-4632-3p
AGCCGCCCUUCUUUCUCU
>Novel:NC_037563.1_39591
GCUUCCCCAGAGUCCUCUU
>Novel:NC_037550.1_18584
UCGUGGAAUGAGAAGCCGUGGC
>Novel:NC_037562.1_38397
ACUGGCUUCCAGUCCUCU
>Novel:NC_037549.1_15051
CGAGUCAUCGUUGGGCCU
>Novel:hsa-miR-4768-5p
AUUCUCUCUCCCUUUUCCU
>Novel:NC_037545.1_1494
GCCCUCCAGUGAUCAGCAAAAUA
>Novel:NC_037545.1_1780
GCAUCGGGACCGCGAGCGCCGAGC
>Novel:NC_037563.1_40056
GAGGAUACAGUGGAAAAG
>Novel:NC_037551.1_20562
GGGACAGAGUCCUCUACAGAUUGAA
>Novel:NC_037562.1_39081
ACACUUCCGGGAUUCUGGGCGG
>Novel:hsa-miR-4508
ACGGGGCUGCACCUGGAG
>Novel:NC_037546.1_4475
AGUCCCUUCCUCCUCUCUCU
>Novel:hsa-miR-4283
GGGGGCUCGAGAACCGAGCGGA
>Novel:NC_037569.1_47777
AAAGUUCGCUUGGAUUUUU
>Novel:NC_037553.1_23738
CCUUCCAGGGAUCUUCUCCU
>Novel:NC_037564.1_41300
AAAACCCAAACGAACUUUU
>Novel:hsa-miR-6516-5p
UUUGCAGUGAUUGGAGCCC
>Novel:NC_037549.1_16794
ACCAAUGAGGAAAGCAGAC
>Novel:NC_037545.1_434
GGCCAGGAUUCCACCCCU
>Novel:NC_037553.1_24731
CCACUGGCCCCACUCAGGGCUG
>Novel:NC_037558.1_32781
CUUAUGGAUAAGGCACUG

>Novel:hsa-miR-6867-5p
UGUGUGUGUGUGUGUGUGUGU
>Novel:NC_037558.1_32628
GCUUGCCACUGAAGAACU
>Novel:NC_037546.1_2789
GACUCUGCAGUCCAGCCUU
